# Crafting for Health: A Longitudinal Study of Job and Off-Job Crafting Changes during the COVID-19 Pandemic

**DOI:** 10.1007/s41542-025-00222-5

**Published:** 2025-02-26

**Authors:** Anja Isabel Morstatt, Georg F. Bauer, Jessica de Bloom, Zachary J. Roman, Martin Tušl, Philipp Kerksieck

**Affiliations:** 1 Industrial/Organizational and Social Psychology, Technische Universität Braunschweig, Brunswick, Germany; 2 Public and Organizational Health / Center of Salutogenesis, Institute of Epidemiology, Biostatistics, and Prevention, University of Zurich, Zurich, Switzerland; 3 Faculty of Social Sciences (Psychology), Tampere University, Tampere, Finland; 4 Department of HRM&OB, Faculty of Economics and Business, University of Groningen, Groningen, Netherlands; 5 Department of Informatics, Social Computing Group, University of Zurich, Zurich, Switzerland; 6 Department of Psychology, Psychological Methods, Evaluation, and Statistics, University of Zurich, Zurich, Switzerland

**Keywords:** Latent change score models, Temporal patterns, Proactive strategies, Individual job redesign, Leisure, DRAMMA needs

## Abstract

We examined the association between changes in employee job and off-job crafting and their self-rated health during the COVID-19 pandemic. Further, we investigated how these associations differed across sample subgroups, contrasting differences in work location, living situation, and contractual changes (short-time work). We used four waves of a longitudinal dataset surveying N_Total_ = 783 German-speaking employees from Germany, Switzerland, and Austria from 2019 to 2021. We applied latent change score modeling and multigroup analyses to investigate our research questions. Results indicated that the mean job and off-job crafting and self-rated health trajectories remained relatively stable. However, we observed significant interindividual variance in job and off-job crafting changes. We found a consistent small positive relationship between crafting changes in both life domains over time, indicating that employees tended to change their crafting efforts similarly across domains. Additionally, job crafting increases between Waves 1 and 2 were linked to higher subsequent self-rated health at Wave 2, and similarly, off-job crafting increases between Waves 3 and 4 were linked to higher self-rated health at Wave 4. We observed only minor differences in this pattern across subgroups. Our results show how adaptive changes in crafting are linked to broader interindividual health differences and help identify groups who are not able to increase crafting during crises and thus could benefit from targeted support. Crafting can be an effective individual strategy for maintaining health, complementing organizational and public health measures. We encourage future research to incorporate temporal and contextual phenomena into crafting research.

**Supplementary Information:**

The online version contains supplementary material available at 10.1007/s41542-025-00222-5.

## Introduction

In March 2020, the World Health Organization (WHO) declared that the COVID-19 pandemic is global (WHO, 2021). The COVID-19 pandemic has since been termed the “largest unplanned field experiments ever in the world of work” (Weigelt et al., 2021, p. 181), and various public health measures were introduced, removed, and even reintroduced to contain the spreading of the virus (Hale et al., 2021). These public health measures, such as forced teleworking, social isolation through contact restrictions, and school and recreational site closures (OECD, 2020a, 2020b; Cotofan et al., 2021; Rudolph et al., 2021), changed daily life in many ways. Some of the changes emerging during the pandemic may even be permanent (Rudolph et al., 2021; Weigelt et al., 2021), possibly expediting developments into the future of work (Ng et al., 2021; Sinclair et al., 2020). For employees, these developments imply more flexibility, autonomy, and the need for proactive actions, such as crafting, to navigate the complexity of their work/life situation (de Bloom et al., 2020). By crafting within the job and outside the job domain, employees proactively and on their own initiative address “certain [life domain characteristics] (…) to better align their [experiences] with their skills, abilities and preferences” (Tims et al., 2021). Within the job domain, job crafting refers to optimizing demands and resources as outlined within the Job Demands-Resources (JD-R) model (Bakker & Demerouti, 2007; Bakker et al., 2023). For example, employees might craft their job to reduce demands they perceive as hindering, e.g., breaking down complex tasks or they might leverage social resources by actively asking colleagues for feedback or structural resources by acquiring new relevant skills (Tims et al., 2012). Outside the job, employees may be proactive as well, which a recent stream of research describes as “needs-based off-job crafting” (Kujanpää et al., 2022) based on the identity-based integrative needs model of crafting (de Bloom et al., 2020). Off-job crafting refers to the proactive satisfaction of six psychological needs according to the DRAMMA model (detachment, relaxation, autonomy, mastery, meaning, and affiliation; D. B. Newman et al., 2014). An individual, therefore, might craft off the job by seeking detachment and relaxation through reading a novel after work, engaging in challenging hobbies to experience mastery, or increasing feelings of affiliation by initiating quality time with loved ones. We highlight two conceptualizations of crafting, noting that proactive efforts vary across life domains. However, we propose examining them together, as both share the same core idea, and off-job crafting can also be viewed through a JD-R perspective.^1^

Crafting strategies help employees stay healthy and well on and off the job (Kujanpää et al., 2022; Rudolph et al., 2017). Job crafting (Tims & Bakker, 2010; Wrzesniewski & Dutton, 2001) is associated with increased work engagement or reduced job strain (Rudolph et al., 2017), and off-job crafting is linked with better satisfaction of psychological needs, including recovery from work (Kujanpää et al., 2021, 2022) and mental well-being (Tušl et al., 2022). However, with one exception (Tušl et al., 2022), these studies predate the pandemic, and few examine changes in crafting (Farrell, 2019; van Wingerden et al., 2017). Crafting changes likely became more pronounced during the pandemic due to dramatic shifts in public health measures. These changes may have heightened the importance of situational factors over stable behavioral tendencies (see Situational Strength Theory; Meyer et al., 2010, 2020),, prompting employees to adjust their crafting efforts to face new challenges and prioritize their health across different life domains.

It remains largely unclear how crafting developed in the life domains throughout the pandemic and how these changes are related to health, which has been a general concern during the crisis. Most studies on job and off-job crafting conducted during the pandemic focused on beneficial outcomes of crafting not on dynamic changes in the crafting efforts themselves (Abdel Hadi et al., 2021; Behzadnia & FatahModares, 2020; Brauchli et al., 2022; Pijpker et al., 2022). However, it is likely that during different phases of the pandemic, individuals increased, decreased, or retained their level of crafting in both or one life domain. We still lack an understanding of how crafting changed during the pandemic, but also whether certain phases or contextual correlates have been associated with strong adaptive responses, emerging as sharp increases or decreases in crafting, and/or with risks for impaired health. We aim to bridge this gap by a) studying the changes in both job and off-job crafting throughout the pandemic, b) reviewing how changes in the extent of crafting are associated with employee health, and c) whether there are meaningful differences in these changes and associations with health between employee subgroups, characterized e.g. by their work location during the pandemic.

Investigating crafting during the COVID-19 pandemic is important for four reasons. First, this study expands the literature by examining crafting in both job and off-job domains, offering a comprehensive view of proactive behaviors. In the job domain, organizations helped implement public health measures like forced teleworking and counterbalancing efforts, for instance, enabling colleagues to maintain virtual contact (OECD, 2020b). Larger organizations may have been slow to identify and implement changes to help employees adapt, while smaller organizations often lacked the resources to provide necessary support during physical distancing (OECD, 2020b). With or without organizational support, employees likely engaged in both job and off-job crafting their experiences when believing this necessary and beneficial, supporting and maintaining their health. Understanding how crafting evolved in these interconnected domains over time enhances our insight into its impact on employee well-being.

Second, we know little about the changes in and patterns of crafting throughout the pandemic, and we believe our study is the first to address this gap. Changes in crafting might not have occurred linearly over the entire course of the pandemic but rather episodic, responding to significant events like lockdowns. A change in crafting might reflect an adaptive response of individuals to externally altered work and living situations due to the pandemic (see also propositions stated by Demerouti and Bakker (2022)). Our methodological approach of latent change score models captures these changes between time points and allows us to examine overall trends and variances (interpersonal differences), in our sample. Specifically, our study, assesses the directions and magnitudes of changes in regard to the frequency of crafting.

Third, we aim to investigate potential contextual correlates in addition to temporal patterns in crafting changes, based on various characteristics such as their living situation or working location (office vs. home office). For instance, of those moving to home offices at the beginning of the pandemic, earlier studies reported both positive and negative side effects of the pandemic (Kaltiainen & Hakanen, 2022; OECD, 2020a, 2020b; Tušl et al., 2021), e.g., more leisure time due to less commuting. Such developments may have enabled employees to leverage positive aspects, for instance, more leisure to invest in quality time with a partner/family. On the other hand, other employees may have needed to focus crafting efforts on a more severely affected life domain, for example, in case of increased workload. Either way, we suggest such differences potentially emerge as different patterns in crafting changes throughout the pandemic. This will advance our understanding of how a combination of temporal and contextual aspects are associated with crafting across life domains.

Lastly, we add to the research by investigating associations of changes in crafting with employee health, extending the findings by assessing multiple time points during the pandemic. By combining how intraindividual changes in crafting relate to subsequent interindividual differences in health, our study provides insights into how adaptive efforts during the pandemic might translate into broader differences in health across individuals. Further, we address potential temporal and contextual differences in the association between crafting and self-rated health. By this, we advance our understanding of whether adaptive efforts have been beneficial during certain periods of the pandemic and whether specific individuals benefitted more (or less) from such adjustments during this crisis. In conclusion, we provide insights for future guidelines and interventions to promote crafting and to ascertain which employee groups likely will experience less severe disruptions in future crisis situations.

## Background

### Changes in Crafting During the Pandemic

During the pandemic, employees needed to balance different aspects of their lives to ensure positive experiences. Challenges during the first and second lockdowns differed; the second lockdown let people apply proven strategies. A study in Austria found that during the second lockdown, people were better able to focus on work or daily activities, feeling less busy than during the first lockdown (Łaszewska et al., 2021). However, they also reported feeling less connected to others and experiencing more negative impacts on family life, leisure, and education (Łaszewska et al., 2021), hence a need to craft their off-job experiences. Therefore, one aim of this study is to investigate how employees’ job and off-job crafting changed considering the different phases of the pandemic.

Moreover, we investigate whether employees changed their crafting in both life domains similarly or focused – potentially temporarily – on one specific life domain. Employees may compensate for the lack of crafting opportunities in one domain by emphasizing crafting in the other (Petrou et al., 2017), but may also craft equally in both (Demerouti et al., 2020). Verelst et al. (2023) revealed associated potential risks, including depleted energy levels. We examine how crafting in the job and off-job domains changes over time based on contextual situations, possibly explaining when employees allocate energy evenly between two domains or focus on one. A (temporal) focus on one domain in crafting might, therefore, emerge as an increase in crafting during a certain period, potentially even accompanied by a decrease in crafting in the other life domain during the same period. Given the study's timeframe from June 2019 to December 2021, we can observe shifts in these foci, as declines in one domain may be followed by increases in another (and vice versa). However, crafting might also evolve jointly across life domains, following ideas often coined as congruency or spillover^2^ across life domains (Guest, 2001; Petrou & Bakker, 2016; Snir & Harpaz, 2002). As it remains unclear how crafting evolved during the pandemic, we combine these ideas into our first research question:

- ***RQ1:**** How did job and off-job crafting levels, and their interrelation, change over the course of the pandemic?*

### Crafting Changes and Health During the Pandemic

The pandemic significantly altered employees' lives and their perceptions of infection risk, both for themselves and others (Jimenez et al., 2023). Health and economic anxiety rose for many (LeNoble et al., 2023). A study found that those who perceived the pandemic impacts negatively reported poorer mental well-being (Tušl et al., 2021), while another study noted minor declines in occupational well-being, particularly among younger employees and those living alone (Kaltiainen & Hakanen, 2022).

We focus on self-rated health, thereby capturing holistically but parsimoniously how employees’ assessments of their health changed over time. Self-rated health combines individual assessments of body and mind within a single rating, and meta-analyses have shown its predictive value for mortality (DeSalvo et al., 2006; Idler & Benyamini, 1997; Jylhä, 2009). Individuals report poorer self-rated health when facing chronic conditions, including mental health problems, or low social support (Amstadter et al., 2010; French et al., 2012; Van Lente et al., 2012). Overall, self-rated health seems a relatively stable concept, as demonstrated before the pandemic (Perruccio et al., 2010) and when comparing pre-pandemic with pandemic health (Peters et al., 2020; Recchi et al., 2020; Szwarcwald et al., 2021; van de Weijer et al., 2022). However, self-rated health remains responsive to varying health conditions, e.g., during convalescence from complex surgeries (Perruccio et al., 2010). Based on pre-pandemic studies linking crafting to health-promoting characteristics (e.g., work engagement, Rudolph et al., 2017), we suggest that crafting during the COVID-19 pandemic will be positively associated with self-rated health.

Although not necessarily implicated in assessing the risks of physical infections, crafting is pivotal in other mental health perceptions, e.g., of social support systems (Holman et al., 2023; Tims et al., 2013). Pre-pandemic research indicates that job crafting interventions enhance self-efficacy (van den Heuvel et al., 2015), which is vital for health. Additionally, off-job crafting is beneficial for increasing sense of meaning in life (Kujanpää et al., 2022; Petrou et al., 2017) and has been associated with improved mental well-being during the pandemic (Tušl et al., 2022).

Studies during the pandemic have reiterated the protective role of crafting. For instance, a simple intervention study utilizing WhatsApp messages prompted Iranian students to increase their needs satisfaction, thereby reducing perceived stress during this critical time (Behzadnia & FatahModares, 2020). In Germany, researchers found that employees proactively crafting their leisure time experienced less emotional exhaustion, underscoring the importance of such proactive measures during initial strict public health measures (Abdel Hadi et al., 2021). Additionally, studies investigating changes from pre-pandemic to pandemic states have demonstrated the benefits of high levels of crafting for health. Employee groups identified as High Job Crafters and High Off-job Crafters reported positive changes in self-rated health before and after the pandemic (Brauchli et al., 2022). Similarly, those actively crafting their off-job domain before the pandemic reported less burnout during those challenging times (Pijpker et al., 2022). However, although emphasizing the positive impacts of crafting, these studies usually refer to crafting levels and interpersonal differences but fail to consider how adaptive efforts, e.g., increasing crafting, might also be related to subsequent higher levels of health.

Our study aims to elaborate on the findings related to crafting changes during the pandemic and their connection to self-rated health. In line with previous research findings, we expect positive associations between a crafting change in one life domain and the subsequent level of self-rated health. Job or off-job crafting increases may enhance personal or social resources, leading to better health outcomes compared to individuals who maintain or reduce their crafting efforts. Our study is the first to examine crafting in both life domains jointly and during the pandemic. It remains unclear whether crafting changes in both life domains are associated with subsequent health to a similar extent or whether crafting changes focused on one life domain played a more substantial role for maintaining health. Additionally, there might have been changes to these patterns throughout the pandemic, such as shifts in domain relevance for health from one pandemic phase to another. To allow for such an open investigation, we pose the following research question:

- ***RQ2:**** How are changes in job and off-job crafting associated with subsequent levels of self-rated health?*

### Interindividual Differences in Crafting Changes During the Pandemic

We explore interindividual differences by taking into account prior studies that suggested certain demographic groups to have faced adverse outcomes to a varying extent during the pandemic. For instance, younger individuals living alone, employees with reduced leisure time, or those with more caregiving duties reportedly perceived a negative impact (Tušl et al., 2021), while job uncertainty increased in the financial crisis following the pandemic (OECD, 2020a, 2020b). Positive side effects emerged concurrently, including perceived improvements in both work and private life (Tušl et al., 2021). Positive impacts on employees included increased leisure time, time spent with partners and family, and reduced commuting (Tušl et al., 2021). Moreover, in the early stages, teleworking employees experienced heightened work engagement – a notable positive side effect of the transformed work landscape (Kaltiainen & Hakanen, 2022).

Three major themes emerged and were further investigated: Firstly, employees’ physical work locations played a pivotal role, with widely differing experiences. Those transitioning to remote work faced unique challenges and opportunities compared to those continuing in physical workspaces. Secondly, living situations significantly influenced the pandemic’s impact. Individuals living alone might have faced reduced social interactions and their implications. Conversely, those living in families found solace in strengthened familial bonds but also incurred increased caregiving duties. Lastly, contractual changes introduced further complexity. Employees with changing job structures, e.g., a forced contractual reduction of the working hours, faced potential uncertainty in the job domain. In Germany, over eight million employees were in a short-time work scheme in April 2020, the highest since 2009 (Bundesagentur für Arbeit, 2024).

Further, the contextual variables mentioned above may well have occasioned changes in employees’ crafting throughout the pandemic to respond to specific needs and opportunities. Certain potentially more vulnerable groups may have intensified their crafting efforts (temporarily) to mitigate negative impacts. For instance, employees transitioning to home offices possibly increased efforts to connect with others virtually to combat social isolation, thereby increasing their job crafting in early phases of the pandemic. Some may have also found benefits through increasing crafting in either life domain, like mastering remote work or greater virtual closeness with loved ones. Additionally, specific groups may have focused on one domain, particularly those facing job insecurity, intensifying job crafting to secure enjoyment and stability amidst uncertainties. We formulate our third research question accordingly:

- ***RQ3:**** How are subgroup differences in work location, living situation, and contractual changes related to changes in job and off-job crafting and their interrelation?*

Finally, we assume in general that increases in crafting should be positively associated with subsequent levels of self-rated health. However, as outlined referring to RQ2, temporal or domain-specific deviations might form noteworthy patterns in the associations between crafting changes and health. Expanding this idea, we might also observe noteworthy patterns linked with contextual factors. For example, it is possible that certain groups' crafting changes were not beneficial in ensuring high levels of health during the pandemic. Identifying such groups might advance our understanding of potential risk groups in crises that cannot support their own health through increasing their crafting in the job and/or off-job domain. We investigate this along with our fourth research question:

- ***RQ4:**** How are subgroup differences in work location, living situation, and contractual changes related to the associations between crafting changes and subsequent self-rated health?*

## Methods

### Study Design, Sample, and Procedure

**Fig. 1:**
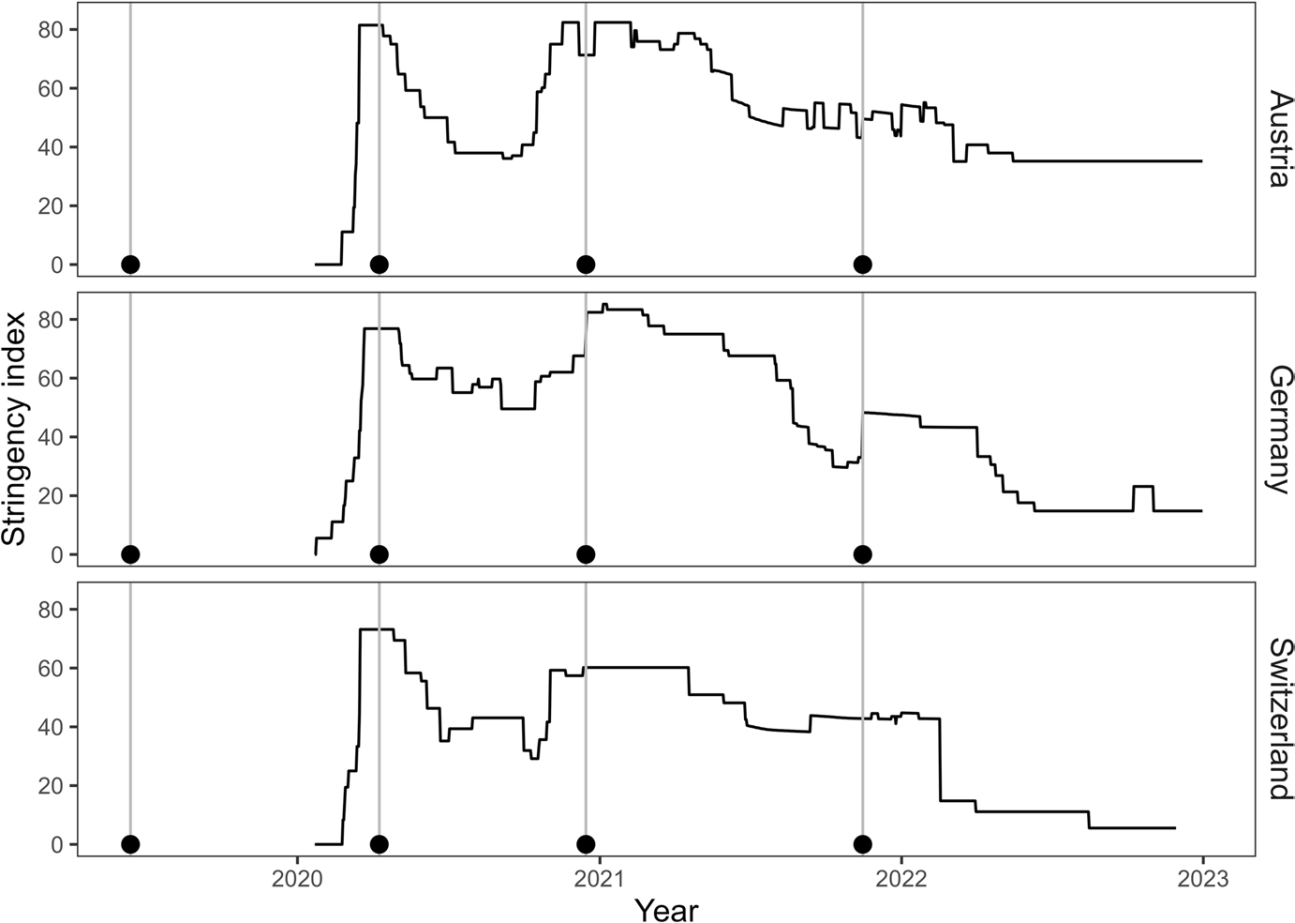
Average stringency index for Germany, Austria, and Switzerland based on the Oxford COVID-19 Government Response Tracker. Note. We plot the average stringency index (0–100) based on the Oxford COVID-19 Government Response Tracker (Hale et al., 2021). The stringency index indicates the severity of political closure and containment measures during the COVID-19 pandemic. After the COVID-19 pandemic was declared as such in March 2020 (WHO, 2021), governments all over the world placed first and stern measures to contain the virus spreading, e.g., nationwide lockdowns paired with school closures (Hale et al., 2021; Rudolph et al., 2021; Weigelt et al., 2021). Towards the summer of 2020, incidence rates of COVID-19 infections decreased (Bundesministerium für Gesundheit, n.d.) and many containment measures were then relaxed. However, in fall 2020, new virus variants increased the risks of infection and mortality again (RKI, 2023; WHO, 2023), so most governments again placed strict containment measures (Hale et al., 2021). Since then, and with the development of effective vaccinations against the virus (Mathieu et al., 2021), most political measures have been relaxed or even removed (Hale et al., 2021). Additionally, we plot our survey waves (Wave 1: June/July 2019, Wave 2: April 2020, Wave 3: December 2020, Wave 4: October/November 2021) within the graph to contextualize each survey timeframe. The starting dates of each survey wave are indicated by black dots at the bottom of each graph and a vertical gray line

We used four survey waves from a larger longitudinal online panel study inviting German-speaking employees from Germany, Switzerland, and Austria to participate repeatedly (panel provider: bilendi, formerly respondi). We chose these four survey waves to divide the pandemic into four phases: pre-pandemic (Wave 1, June/July 2019), post-onset and first lockdown (Wave 2, April 2020), second lockdown (Wave 3, December 2020), and incipient normalization phase (Wave 4, November/December 2021). To plot the data of the stringency index (scoring closure and containment measures) against our survey waves in Fig. 1, we referred to data from the Oxford COVID-19 Government Response Tracker (Hale et al., 2021).

The extensive longitudinal panel study, in which ours is embedded, includes two prior study waves in 2018 and 2019, not considered here. Generally, participants were invited to participate in the following survey waves. However, to expand the set, we invited all participants from previous waves to participate in our second survey wave. Since some participants decided to rejoin the panel study, our total *N* is highest for Wave 2 (*N* = 783); 573 participants completed our survey in Wave 1, 500 in Wave 3, and 399 in Wave 4. For all waves, we included participants currently working over 20 h per week and excluded self-employed individuals. We cleaned the data after collection to remove careless responses, mainly speeders and straightliners (Meade & Craig, 2012).

We present demographics from Wave 2, the key survey wave. Table 1 presents all demographics for the full sample and all group comparisons.

**Table 1:** Demographics of full sample and subgroups

	Full sample		Work location						Living situation				Contractual changes			
	(*N* = 783)		*FOW* *(n* = *407)*		*HO exp* *(n* = *187)*		*HO new* *(n* = *162)*		*Alone* *(n* = *213)*		*P/F* *(n* = *540)*		*CC* *(n* = *187)*		*NC* *(n* = *588)*	
Characteristic	*M*	*SE*	*M*	*SE*	*M*	*SE*	*M*	*SE*	*M*	*SE*	*M*	*SE*	*M*	*SE*	*M*	*SE*
Age	48.72	9.90	50.14	9.38	47.07	10.38	47.17	10.11	48.50	10.16	48.99	9.72	48.86	9.82	48.79	9.89
Gender (% female)	46.23%		51.35%		41.71%		37.04%		51.64%		43.34%		50.27%		44.39%	
Living situation																
Alone	27.20%		28.78%		23.50%		29.63%		100%		0%		24.06%		28.62%	
With partner or family	68.97%		67.99%		74.86%		67.90%		0%		100%		72.19%		68.79%	
With roommates	2.81%		3.22%		1.64%		2.47%		0%		0%		3.74%		2.59%	
No. of persons needing caregiving	1.20	1.33	1.18	1.28	1.4	1.52	1.04	1.21	0.38	0.55	1.54	1.4	1.39	1.33	1.15	1.33
Of that: No. of children under 11 years	0.19	0.51	0.14	0.44	0.25	0.57	0.27	0.60	0.02	0.17	0.26	0.57	0.25	0.55	0.17	0.49
Working hours per week^a^	38.14	7.02	37.13	7.44	39.26	6.67	39.07	6.11	39.19	6.33	37.74	7.21	37.39	7.2	38.34	6.97
Job function																
Apprentice, student, intern	0.38%		0.50%		0.54%		0%		0%		0.37%		0%		0.51%	
Employee without supervising function	70.37%		77.92%		56.99%		69.75%		77.14%		68.40%		65.95%		72.14%	
Employee with supervising function	24.14%		18.11%		36.02%		24.69%		20.95%		26.02%		28.65%		23.08%	
Employee in C-Level position	4.47%		3.47%		6.45%		5.56%		1.90%		5.20%		5.41%		4.27%	
Percentage home office																
Before pandemic	8.50%	21.32	0	0	32.67	29.66	0	0	6.60	18.80	9.29	22.39	8.18	21.07	8.53	21.21
Since pandemic	34.28%	42.98	0	0	73.69	33.55	78.33	27.69	34.62	43.87	34.42	42.77	22.73	35.75	38.12	44.43
Temporary contractual work reduction	23.88%		27.52%		20.86%		16.66%		21.13%		25%		100%		0%	
COVID-19 specific items (Wave 3)	*(n* = *470)*		*(n* = *233)*		*(n* = *122)*		*(n* = *98)*		*(n* = *131)*		*(n* = *321)*		*(n* = *106)*		*(n* = *363)*	
Belonging to high-risk group	28.51%		27.47%		25.41%		33.67%		22.90%		30.22%		28.30%		28.37%	
Quarantined (Self)	5.74%		4.72%		5.74%		8.16%		6.87%		5.61%		5.66%		5.79%	
Quarantined (Family)	14.26%		14.59%		11.48%		17.35%		5.34%		18.69%		15.09%		14.05%	
Diagnosed COVID-19 (Self)	1.70%		2.15%		0%		3.06%		0.76%		2.18%		1.89%		1.65%	
Diagnosed COVID-19 (Family)	6.60%		5.58%		6.56%		7.14%		2.29%		8.41%		8.49%		6.06%	

Demographics were assessed at Wave 2, except for COVID-19-specific items, which were assessed only at Wave 3 as a voluntary additional survey at the end of the main survey. We report the available sample sizes in this section. In Germany and Switzerland, parents with children under 12 years enjoyed special protection during the COVID-19 pandemic (CMS Law Tax Future, 2022; Deutscher Gewerkschaftsbund, 2022). For example, parents were entitled to additional sick days if needed (CMS Law Tax Future, 2022; Deutscher Gewerkschaftsbund, 2022). In our data collection, we asked the age of the person needing caregiving offering categories, with “7–10 years” being closest to the threshold presented by political support for parents. Therefore, we set the cut-off to define the special caregiving duties for children at 11 years. FOW = Full office workers, HO exp. = Home office experienced, HO new = Home office new, P/F = Living with partner or family, CC = Contractual changes, NC = No contractual changes

^a^ Weekly working hours were assessed using categories from 0–9 to 49 + . We report the mean via the midpoints of the categories and 54.5 as the last category midpoint

We investigated attrition in our sample, comparing those with complete participation (*n* = 300) against those with incomplete participation (*n* = 483, missing at least one wave) regarding demographics (age, gender, caregiving duties, working hours) and the variables in our model (job crafting, off-job crafting, self-rated health), all at Wave 2. We found mean differences for age *M*_Completes_ = 49.84 years vs. *M*_Incompletes_ = 48.02 years (*t*(697) = 2.592, *p* = 0.009), gender *M*_Completes_ = 1.42 vs. *M*_Incompletes_ = 1.49 (1 = “*male”*, 2 = “*female”*, 3 = “*other”*; *t*(643.89) = −1.986, *p* = 0.047), and caregiving duties (number of persons cared for) *M*_Completes_ = 1.05 vs. *M*_Incompletes_ = 1.30 years (*t*(681.37) = −2.582, *p* = 0.01). Effect sizes for these comparisons remained below small effects (Age: *d* = 0.20, Gender: *d* = 0.16, Caregiving duties: *d* = 0.20).

### Measures

For each wave, participants completed an online survey including the following measures. We assessed all variables in German, anchored using a 5-point Likert scale from 1 = “*strongly disagree*” to 5 = “*strongly agree*” unless otherwise stated. We present means, standard deviations, internal consistencies using Cronbach’s alpha, and intercorrelations in Table 2. All demographic variables (age, gender, weekly working hours, job function, sector, living situation, caregiving duties, contract changes, and percentage working in home office) presented in Table 1 and partly used to segment our sample for later group comparisons were used from the assessment at wave 2.

**Table 2:** Means, standard deviations, correlations, and internal consistencies for multiple indicator scales

Variable	*M*	*SD*	1	2	3	4	5	6	7	8	9	10	11	12	13	14
1. Gender^a^	1.46	0.50	–													
2. Age	48.72	9.90	.02	–												
3. JC W1	2.99	0.48	.00	-.17***	*.84*											
4. JC W2	2.97	0.48	-.01	-.20***	.74***	*.85*										
5. JC W3	2.94	0.51	-.01	-.15***	.74***	.79***	*.86*									
6. JC W4	2.81	0.50	.04	-.17***	.73***	.77***	.77***	*.86*								
7. OJC W1	3.78	0.60	.06	.11**	.24***	.19***	.17***	.16**	*.91*							
8. OJC W2	4.07	5.93	.03	-.00	-.08*	-.05	-.07	-.14**	-.03	*.92*						
9. OJC W3	3.57	0.63	-.02	.03	.23***	.27***	.33***	.30***	.59***	.01	*.93*					
10. OJC W4	3.61	0.63	.03	.09	.22***	.20***	.24***	.24***	.59***	-.02	.66***	*.93*				
11. SRH W1	3.59	0.79	.08	-.20***	.15***	.12**	.17**	.13*	.21***	.03	.25***	.23***	–			
12. SRH W2	3.67	0.77	.01	-.17***	.17***	.15***	.22***	.14**	.18***	.05	.32***	.21***	.73***	–		
13. SRH W3	3.71	0.76	.02	-.12**	.08	.09*	.20***	.12*	.25***	-.00	.34***	.21***	.69***	.71***	–	
14. SRH W4	3.67	0.78	.02	-.05	.07	.05	.12*	.05	.26***	-.00	.31***	.33***	.65***	.67***	.68***	
15. BRG W3	0.29	0.45	-.03	.40**	-.04	-.03	-.10*	-.04	.08	-.02	-.01	.09	-.30**	-.30**	-.30**	-.20**

We include gender and age assessed at W2. *M* = Mean, *SD* = Standard deviation, JC = Job crafting, OJC = Off-job crafting, SRH = Self-rated health, BRG = Belonging to risk group. Means and standard deviations were obtained using mean scores for the measures. Cronbach’s alpha is reported in italics along the diagonal for constructs with multiple indicators. * = *p* < 0.05, ** = *p* < 0.01, *** = *p* < 0.001

^a^ 1 = male, 2 = female

We assessed four facets of job crafting (increasing structural job resources, increasing social job resources, increasing challenging job demands, and reducing hindering job demands) using a total of 17 items (five, five, three, and four items for the facets) adapted from the Job Crafting Scale by Tims et al. (2012) and a version used by Petrou et al. (2012). We referred to the items by Petrou et al. (2012) for the facet “increasing challenging job demands” to address concerns regarding the complex wording of the version by Tims et al. (2012) (Nielsen & Abildgaard, 2012). Participants rated the items using a scale from 1 = “*never*” to 5 = “*very often*”. Example items for each facet are: “*I try to develop my capabilities*” (increasing structural job resources); “ *I ask others for feedback on my job performance.”* (increasing social job resources); *“I make sure that my work is mentally less intense.”* (reducing hindering job demands); *“I ask for more tasks when I have finished my work.”* (increasing challenging job demands).

The six facets of off-job crafting^3^ (detachment, relaxation, autonomy, mastery, meaning, and affiliation) were assessed using 18 items (three items per facet) from the Needs-based Off-job Crafting Scale developed by Kujanpää et al. (2022). Participants rated the items using a scale from 1 = “*never*” to 5 = “*very often*”. In the early stages of the data collection (Waves 1 & 2), an additional residual option, “I don’t know,” was presented to participants. We coded this residual option as a missing value. We introduced each item with the prefix “*Over the past month, …”.* Example items for each facet are: “*I’ve made sure to detach from work-related thoughts during off-job time.*” (Detachment); “*I’ve made sure to experience relaxation of my body and mind during off-job time.*” (Relaxation); “*I’ve organized my off-job activities so that I determine my own course of action.*” (Autonomy); “*I’ve organized my off-job activities so that I put my skills, knowledge or abilities into action.*” (Mastery); “*I’ve organized my off-job activities so that I achieve a sense of purpose in what I am doing.*” (Meaning); “*I’ve made sure to experience close connections to the people around me during off-job time.*” (Affiliation).

Self-rated health was measured using a single item as suggested by the WHO (1996). The item was “*How would you rate your health in general?*” with response options from 1 = *“very bad”* to 5 = *“very good”.*

Lastly, we controlled for belonging to a high-risk group for severe COVID-19 infections. At Wave 6, we asked participants: “Do you belong to a high-risk group for the coronavirus due to your age or an illness?” (0 = “*no”*, 1 = “*yes”*). We decided to include this measure as a control variable, as both COVID-19 infection rates (at Wave 6: 1.702%) and having been in quarantine (at Wave 6: 5.745%) only occurred with a very low base rate in our sample.

### Data Analysis Strategy

For data analysis, R (R Core Team, 2020) and the following packages were used: *tidyverse* (Wickham et al., 2019), *readr* (Wickham & Hester, 2021), and *lubridate* (Grolemund & Wickham, 2011) for data handling, *ggplot2* (Wickham, n.d.) and *apaTables* (Stanley, 2021) for plot and table creation, and *psych* (Revelle, 2021) and *lavaan* (Rosseel, 2012) for data analysis. We openly provide additional material in the Electronic Supplementary Materials 1–5 (e.g., overview of model fits and comparison tests for measurement invariance and latent growth curve testing, full model results of final models, R syntax regarding additional analyses regarding multigroup analyses based on caregiving duties).

First, we added categorical variables indicating our subgroups of interest to assess the third research question to our dataset.^4^ We added three categorical variables representing employees’ work location, living situation, and contractual changes based on information collected at the beginning of the COVID-19 pandemic (Wave 2). The first set of subgroup comparisons covered the work location. We defined three subgroups: Full office workers (*N* = 407), home office workers experienced (*N* = 187), and home office workers inexperienced (*N* = 162). The second set of subgroup comparisons covered the living situation. We formed two subgroups: Living alone (*N* = 213) and living with partner/family (*N* = 540). The third set of subgroup comparisons covered the contractual changes. We formed two subgroups: no contractual changes (*N* = 588) and contractual changes with temporary work reduction up to zero percent (*N* = 187).

Next, we noted missing data in our dataset due to participation dropout and the selection of residual options (in off-job crafting). While missing values are common, appropriate handling is required to prevent biased parameter estimates. We follow the suggestions by Newman (2014) and employ full information maximum likelihood methods for our analyses to handle both full missing survey participations and partial missings on selected scales.

Before seeking answers to our research questions, we assessed important assumptions. When exploring differences over time and across groups, the main assumption is measurement invariance (Putnick & Bornstein, 2016; Steinmetz et al., 2009; Vandenberg & Lance, 2000). Measurement invariance refers to the stability of constructs examined regarding their structure, factor loadings, and intercepts across time points and/or groups. With measurement invariance established, researchers can assume that the understanding of a construct remains unchanged between time points or groups, and comparisons between time points and groups become meaningful. Therefore, we started our analyses with elaborate measurement invariance testing for our multiple indicator constructs job and off-job crafting using item parcels along their respective facets. We referred to item parcels for job and off-job crafting to reduce the parameters in the models (Little et al., 2002; Orcan, 2013). We first assessed longitudinal measurement invariance for the full sample and all individual subgroups for later comparison (e.g., workers staying in their offices during the pandemic vs. workers moving to home offices for the first time). Next, we assessed intergroup measurement invariance referring to Wave 2. For all tests, measurement models using confirmatory factor analyses (CFA) were specified for the different stages of measurement invariance (configural, metric, scalar). To evaluate the models, we referred to recommended cut-off criteria: Root Mean Square Error of Approximation (RMSEA < 0.06), Comparative Fit-Index (CFI close to 0.95), Tucker-Lewis-Index (TLI close to 0.95), Standardized Root Mean Square Residual (SRMR < 0.08) (Hu & Bentler, 1999). Additionally, model comparison tests were performed to find the best-fitting model.

We specified a series of bivariate latent change score models (LCSM) to answer our first and second research questions. First, we specified an LCSM for the full sample, including self-rated health as an outcome of crafting changes. We followed, adapted, and extended the modeling approach for the LCSM by Geiser (2020) and Wiedemann et al. (2022).

To answer our third and fourth research questions, we again specified a series of LCSMs, but to compare subgroups, we nested those within three multigroup analyses (MGA). Per group comparisons of interest, we specified one MGA incorporating the same LCSM for each subgroup. For all models, we controlled for belonging to a high-risk group as a predictor of self-rated health. Additionally, we compared models with and without equality constraints (e.g., restraining covariances of job and off-job crafting changes to be equal over time and restraining change score intercepts to be equal across groups). We retained the equality constraint if the model comparison test indicated that the data could be equally well described by a more parsimonious (restrained) model. We next present the final models. Overviews of model fits and comparison tests are given in ESM 1 and ESM 2.

## Results

### Measurement Invariance, Equality Constraints, and Model Fit Indices

For the full sample and for the multigroup analyses splitting the sample by work location and living situation, we established partial scalar measurement invariance (invariant structure, same factor loadings, partially same item intercept) both over time and across groups for both job and off-job crafting. For the multigroup analysis splitting the sample by contractual changes, we could only establish partial metric and scalar measurement invariance for job crafting and full metric measurement invariance for off-job crafting. This suggests the job crafting measure is not exactly the same when comparing the group with contractual changes and the group without contractual changes at the beginning of the COVID-19 pandemic, as indicated by the need to release one factor loading from the parcel of “increasing social job resources” to the overall job crafting factor. In response, we fixed as much of the construct between groups as possible to ensure comparable results. However, allowing for partial measurement invariance yields only an approximation and will be discussed later (Putnick & Bornstein, 2016).

We implemented and tested several equality constraints within our models. We applied an equality constraint on the job and off-job crafting change score covariances across time for the full sample. Given the unequal time intervals between the measurements in our data, most parameters, e.g., regression parameters, are impacted by this. Therefore, an equality constraint on the covariances across time was the only comparison test that could be tested for the full sample. We applied equality constraints across groups for the multigroup analyses to ensure we interpret meaningful and not only spurious differences in parameters. Across groups, we tested for the equality of the auto-regressive effects of self-rated health, the regression from our control variable to self-rated health, the regressions from job and off-job crafting changes to self-rated health, the covariances between job and off-job crafting change scores, and the change score intercept of both job and off-job crafting. Our data indicated that the more parsimonious models with equality constraints could, in most cases, be retained. We present the final model fit indices, all of which indicate good model fit, in Table 3. We provide an overview of model fits and comparison tests for measurement invariance and equality constraints in our supplementary material ESM 1-4.

**Table 3:** Model fit indices for latent change score models

Modell	χ^2^	df	CFI	TLI	RMSEA	SRMR
*Full sample analyses*						
Baseline bivariate LCSM with SRH	2004.200***	872	0.928	0.918	0.041	0.076
*Multigroup analyses*						
Bivariate LCSM with SRH for group comparison of work location	4895.312***	2698	0.864	0.850	0.057	0.093
Bivariate LCSM with SRH for group comparison of living situation	3357.756***	1784	0.900	0.889	0.048	0.086
Bivariate LCSM with SRH for group comparison of contract changes	3226.126***	1764	0.909	0.898	0.046	0.085

*LCSM* Latent change score model, *SRH*  Self-rated health. For the full sample model, we retained the equality constraint on the covariances between job and off-job crafting change scores across time. For the model investigating the differences in work location, we only rejected equality constraints on the job crafting change score intercepts at Wave 4 and the off-job crafting change score intercepts at Waves 1, 2, and 4. For the model investigating the differences in living situation, we only rejected equality constraints on the regression from our control variable to self-rated health at Wave 4, the regression from off-job crafting change score to self-rated health at Wave 4, the job crafting change score intercepts at Wave 4, and the off-job crafting change score intercepts at Waves 3 and 4. For the model investigating the differences of contract changes, we only rejected equality constraints on the covariances of job and off-job crafting changes between Waves 1 and 2, the regression from off-job crafting change score to self-rated health at Wave 3, the job crafting change score intercepts at Wave 4, and the off-job crafting change score intercepts at Waves 3 and 4. * *p* < .05 ** *p* < .01 *** *p* < .001

### Latent Change Score Models

To answer our RQs 1–4, we next present the results from our LCSM analyses for the full sample and the multigroup analyses (Comparison by work location: HO new = new in home office, FOW = Full office workers, HO exp = experienced in home office; comparison by living situation: alone, P/F = Living with partner/family; comparison by contractual changes: NC = No contractual changes, CC = Contractual changes). RQ1 referred to the crafting changes over time and their interrelations; RQ2 considered temporal differences in the (positive) associations between crafting changes and subsequent levels of self-rated health. As RQ3 and RQ4 can be considered enhancements of RQ1 and RQ2 that explore differences in the patterns depending on contextual variables, we will present these results in combination with RQ1 and RQ2.

**Fig. 2:**
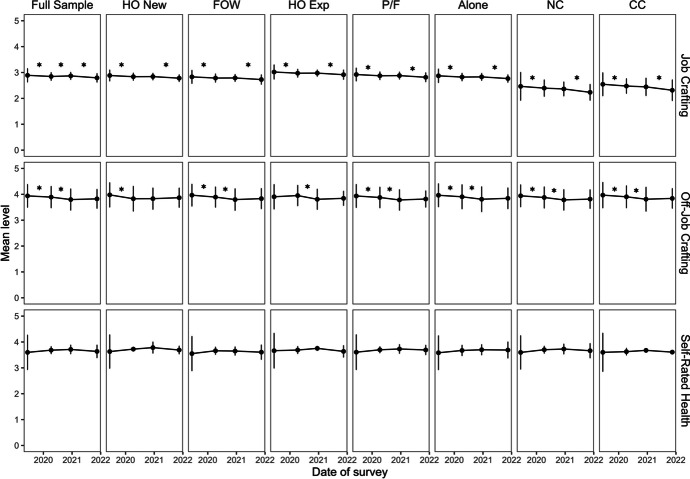
Job and off-job crafting and self-rated health trajectories. *Note*. HO new = new in home office, FOW = Full office workers, HO exp = experienced in home office, P/F = Living with partner/family, NC = No contractual changes, CC = Contractual changes. The sample was split according to reported demographics at Wave 2. Sample sizes: N _HO new/FOW/HO exp/HO new_ = 162 / 407 / 187, N _P/F/alone_ = 540 / 213, N _NC/CC_ = 588 / 187. Mean trajectories across time for job and off-job crafting as well as self-rated health. The values are generated from the latent change score models. Stars between two survey time points represent a significant mean change as indicated by a significant intercept of the respective change score. Self-rated health was modeled as an autoregressive model; no information about significant changes is available

**Fig. 3:**
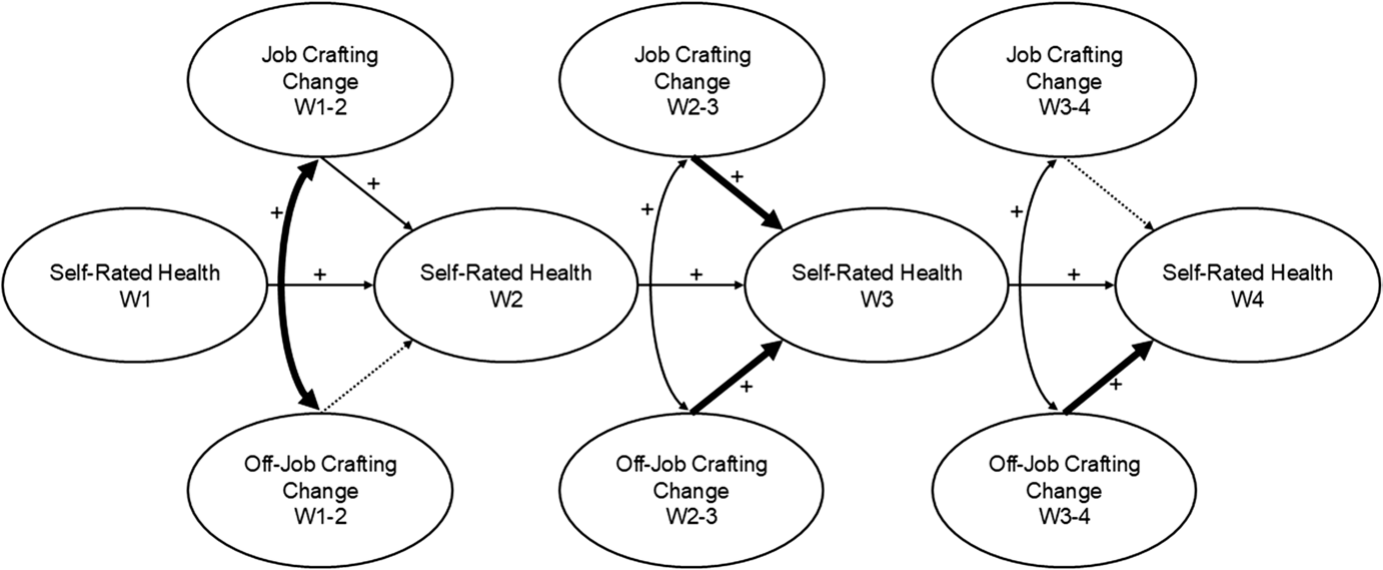
Graphical Summary of LCSM results. *Note*. W = Wave. We present a graphical summary of the model results focusing on the crafting changes and their relations with health over time. Crafting changes were modeled using latent change score modelling (Geiser, 2020; Wiedemann et al., 2022); however the underlying latent variables for crafting per time point are omitted in this figure to reduce complexity. This summary considers the full sample model as well as the group comparisons to highlight similarities and differences. We omit numerical model estimates and focus on the conceptual level. Non-dotted and non-bolded lines represent significant paths within our models without differences across group comparisons. Dotted lines represent non-significant paths within our models. Bolded lines represent differences in this model estimate in different group comparisons. A detailed outline of these changes is presented in the results sections and in the electronic supplementary materials

For an overview, we present the estimated latent trajectories of job and off-job crafting as well as self-rated health in Fig. 2, a conceptual summary of the latent change score model associations in Fig. 3, and a summary of the most relevant model estimates and conclusions in Table 4.^5^ As our analyses are inherently exploratory, we highlight several features of the model results related to our research questions (Latent score intercepts, variances, and covariances, as well as the latent regression coefficients).

**Table 4:** Summary of research questions, related model estimates, and conclusions

		Full sample	Work location			Living situation		Contractual changes	
			HO new	FOW	HO exp	P/F	Alone	NC	CC
Research question	Parameter	EST (SE)	EST (SE)	EST (SE)	EST (SE)	EST (SE)	EST (SE)	EST (SE)	EST (SE)
RQ1 & RQ3^a^	**Mean trends of change: Latent change score intercepts**								
	JC W1-2	−0.044 (0.01) ***	−0.046 (0.01) ***			−0.043 (0.01) ***		−0.032 (0.025)	
	JC W2-3	0.022 (0.01) *	0.011 (0.009)			0.016 (0.01)		−0.002 (0.026)	
	JC W3-4	−0.075 (0.013) ***	−0.065 (0.013) ***			−0.064 (0.012) ***		−0.147 (0.03) ***	
	OJC W1-2	−0.047 (0.019) *	−0.147 (0.048) **	−0.074 (0.025) **	0.05 (0.037)	−0.06 (0.019) **		−0.066 (0.019) ***	
	OJC W2-3	−0.095 (0.02) ***	0.002 (0.04)	−0.097 (0.026) ***	−0.141 (0.036) ***	−0.094 (0.02) ***		−0.092 (0.02) ***	
	OJC W3-4	0.028 (0.021)	0.036 (0.021)			0.037 (0.02)		0.03 (0.021)	
	**Interrelations: Concurrent covariances of latent change scores**								
	W1-2	0.008 (0.003) **	0.01 (0.003) **			0.007 (0.003) *		0.025 (0.006) ***	−0.018 (0.015)
	W2-3	0.008 (0.003) **	0.01 (0.003) **			0.007 (0.003) *		0.025 (0.006) ***	
	W3-4	0.008 (0.003) **	0.01 (0.003) **			0.007 (0.003) *		0.025 (0.006) ***	
Conclusion	We observed small decreases across the four waves in both JC and OJC, with one exception: JC between Waves 2 and 3, which showed little evidence of change. There were almost no differences between groups. During the pandemic, JC and OJC tended to increase or decrease at the same time, except for employees with contractual changes (CC) at the beginning of the pandemic (W1-2)								
RQ2 & RQ4	**Structural regression estimates crafting change scores to subsequent levels of self-rated health**								
	***Pre-pandemic to pandemic onset***								
	JC W1-2 to SRH W2	0.497 (0.203) *	0.419 (0.202) *			0.452 (0.207) *		0.245 (0.095) **	
	OJC W1-2 to SRH W2	0.087 (0.06)	0.107 (0.062)			0.115 (0.06)		0.095 (0.061)	
	BRG W3 to SRH W2	−0.049 (0.068)	−0.056 (0.07)			−0.058 (0.067)		−0.05 (0.069)	
	***Pandemic onset to second lockdown***								
	JC W2-3 to SRH W3	0.468 (0.251)	0.666 (0.276) *			0.411 (0.254)		0.197 (0.117)	
	OJC W2-3 to SRH W3	0.114 (0.062)	0.102 (0.064)			0.113 (0.06)		0.161 (0.074) *	−0.002 (0.1)
	BRG W3 to SRH W3	0 (0.064)	0.029 (0.066)			−0.023 (0.065)		0.006 (0.065)	
	***Second lockdown to normalization phase***								
	JC W3-4 to SRH W4	−0.026 (0.237)	−0.099 (0.232)			0.098 (0.241)		0.001 (0.108)	
	OJC W3-4 to SRH W4	0.236 (0.088) **	0.238 (0.093) *			0.507 (0.132) ***	0.054 (0.144)	0.209 (0.087) *	
	BRG W3 to SRH W4	0.123 (0.075)	0.113 (0.077)			0.012 (0.083)	0.413 (0.14) **	0.103 (0.074)	
Conclusion	At the start of the pandemic (W1-2), increases in JC were linked to better subsequent self-rated health. Toward the end of the pandemic (W3-4), such positive associations emerged instead between increases in OJC and subsequent self-rated health. For individuals living alone, however, increases in OJC between W3-4 were not associated with subsequent self-rated health								

^a^ In the results section, we also present the model estimates for latent change score variances but omit these here to keep the summary concise

*HO new*  new in home office, *FOW* Full office workers, *HO exp* experienced in home office, *P/F* Living with partner/family, *NC* No contractual changes, *CC* contractual changes, *JC* Job crafting, *OJC* Off-job crafting, *SRH* Self-rated health, *BRG* Belonging to risk group. In case of no differences between groups in the group comparisons, we only report one estimate per block. For the full model results, we kindly refer interested readers to ESM 3. * *p* < .05 ** *p* < .01 *** *p* < .001

#### RQ1 and RQ3: Changes in Job and Off-Job Crafting and Their Interrelations

We examine changes in job and off-job crafting across time by referring to the means of latent change scores. Significant latent change score means (results see Table 4) represent a common trend of change for the sample and are visible as a star in between two time points in Fig. 2. For the full sample, we observe a decreasing trend between Waves 1 and 2 both for job crafting and off-job crafting. Between Waves 2 and 3, job crafting increased in the full sample, but off-job crafting further decreased. Finally, between Waves 3 and 4, we find a further decrease for job crafting, but no significant mean trend for off-job crafting. Further, latent change score variances indicate the extent of interindividual variance in crafting changes. Our results show overall significant variance in changes between all time points, given that the variances of job crafting range between *ϕ*_JC CS2_ = 0.02, *SE* = 0.004, *p* < 0.001 (Waves 2 to 3), and *ϕ*_JC CS3_ = 0.029, *SE* = 0.006, *p* < 0.001 (Waves 3 to 4), and variances of off-job crafting range between *ϕ*_OJC CS3_ = 0.137, *SE* = 0.017, *p* < 0.001 (Waves 3 to 4), and *ϕ*_OJC CS1_ = 0.174, *SE* = 0.019, *p* < 0.001 (Waves 1 to 2).

In our multigroup analyses, most equality constraints on the latent change score means (results in Table 4) could be retained, indicating that the groups differ little from each other in their pattern of change. Job crafting showed the same pattern for all subgroups depending on the work locations. For off-job crafting, the pattern of change differed between the subgroups with different work locations. For those new to home office, we observed a decrease in off-job crafting between Waves 1 and 2 and no further mean trends at later time intervals. For those full-time in the office, we observed decreases in off-job crafting between Waves 1 and 2 and between Waves 2 and 3, but no trend between Waves 3 and 4. For those experienced in home office, we observed no mean trends between Waves 1 and 2 and between Waves 3 and 4, but we find a decrease of off-job crafting between Waves 2 and 3. We again observed significant variance of changes between all time points for all subgroups. For job crafting, the variances ranged *ϕ*_JC CS2_ = 0.016, *SE* = 0.006, *p* = 0.006 (Waves 2 to 3, HO exp), and *ϕ*_JC CS3_ = 0.036, *SE* = 0.008, *p* < 0.001 (Waves 3 to 4, FOW). For off-job crafting, variances ranged *ϕ*_OJC CS3_ = 0.078, *SE* = 0.018, *p* < 0.001 (Waves 3 to 4, HO exp), and *ϕ*_OJC CS1_ = 0.233, *SE* = 0.041, *p* < 0.001 (Waves 1 to 2, HO new).

For those living alone or with partner/family, we observed the same patterns of change for job crafting and off-job crafting. Further, we again observed significant variance of changes between all time points for all subgroups. For job crafting, the variances ranged *ϕ*_JC CS2_ = 0.02, *SE* = 0.006, *p* = 0.001 (Waves 2 to 3, alone), and *ϕ*_JC CS3_ = 0.032, *SE* = 0.007, *p* < 0.001 (Waves 3 to 4, P/F). For off-job crafting, variances ranged *ϕ*_OJC CS3_ = 0.101, *SE* = 0.016, *p* < 0.001 (Waves 3 to 4, P/F), and *ϕ*_OJC CS2_ = 0.235, *SE* = 0.038, *p* < 0.001 (Waves 2 to 3, alone).

Comparing those with and without contractual changes, we observed the same change patterns for job crafting and off-job crafting. Further, we again observed significant variance of changes between all time points for all subgroups. For job crafting, the variances ranged *ϕ*_JC CS2_ = 0.079, *SE* = 0.014, *p* < 0.001 (Waves 2 to 3, NC), and *ϕ*_JC CS3_ = 0.17, *SE* = 0.04, *p* < 0.001 (Waves 3 to 4, CC). For off-job crafting, variances ranged *ϕ*_OJC CS3_ = 0.132, *SE* = 0.018, *p* < 0.001 (Waves 3 to 4, NC), and *ϕ*_OJC CS2_ = 0.219, *SE* = 0.038, *p* < 0.001 (Waves 2 to 3, CC).

Interestingly, we observed decreasing mean trends for both job and off-job crafting, except for the slight increase in job crafting between Waves 2 and 3 for the full sample. This pattern did not re-emerge in any of our subgroups. However, between Waves 2 and 3, mean trends for job crafting in the subgroups do not reach the significance level of α = 0.05. All variances in job crafting change scores were significant within this time interval, pointing to heterogeneity in crafting trends, which remains unexplained by the demographic variables. Lastly, we note that visually (see Fig. 2), all job and off-job crafting trajectories remain relatively flat during the pandemic as the magnitudes of crafting changes were relatively small.

Lastly, within RQ1 and RQ3, we also considered the associations between job and off-job crafting changes over time. To answer these research questions, we referred to the cross-sectional covariances of job and off-job crafting changes and further tested whether equality constraints across time would hold as an indicator of a non-changing relationship between the changes. For the full sample and all subgroups, we could restrict covariances of crafting change scores to be equal across time and groups (exception: Waves 1 to 2 for the group comparison of contractual changes). The raw estimates of the covariance are included in Table 4. The only difference observed was for the group with contractual changes between Waves 1 and 2. In this case, the covariance between job and off-job crafting changes was insignificant (*ϕ*_JC CS1, OJC CS1_ = −0.018, *SE* = 0.015, *p* = 0.229). Standardizing these estimates lets us examine the strength of these associations, as they then represent correlations. Given that we allowed the variances to be estimated freely, the standardized estimates of covariances varied between all time points and groups. However, we note that no standardized estimate was greater than 0.30 (range from *r*_JC CS1, OJC CS1_ = 0.103, *CI* = [0.018; 0.189] (Waves 1 to 2, alone) to *r*_JC CS2, OJC CS2_ = 0.218, *CI* = [0.105; 0.331] (Waves 2 to 3, NC)), which would be the threshold to consider an effect of medium size (Rosenthal, 1996). Therefore, all covariances indicated a weak positive association between job and off-job crafting changes.

#### RQ2 and RQ4: Associations Between Crafting Changes and Self-Rated Health

Lastly, we reviewed the associations between crafting changes and self-rated health. To answer these research questions, we refer to the regression estimates from crafting changes to subsequent self-rated health (results in Table 4). A summary of these associations and patterns are graphically presented in Fig. 3. In our models, we controlled for a potential association between self-reported belonging to a COVID-19 high-risk group (assessed only at Wave 3) and self-rated health (not included in Fig. 3). Interestingly, this relationship only reached significance for those living alone at Wave 4.

For the full sample, we observed that positive job crafting changes between Waves 1 and 2 positively predicted self-rated health at Wave 2, but at no other time point. For off-job crafting, we observed the inverse temporal pattern. Positive off-job crafting changes between Waves 1 and 2 and Waves 2 to 3 were not associated with self-rated health at Waves 2 and 3, respectively. However, positive changes in off-job crafting between Waves 3 and 4 positively predicted self-rated health at Wave 4.

Throughout the multigroup analyses, we observed similar patterns. First, comparing groups by work location revealed no differences in associations between crafting changes or the control variable with self-rated health. We observed that job crafting changes between Waves 1 and 2 and Waves 2 and 3 positively predicted subsequent self-rated health. Further, we observed that off-job crafting changes between Waves 3 and 4 positively predicted self-rated health at Wave 4.

Comparing those living alone to those living with partner/family, we observed in both groups that job crafting changes between Waves 1 and 2 positively predicted self-rated health at Wave 2. However, the pattern regarding off-job crafting changes found in the full sample only re-emerged for those living with partner/family. Here, we observed that off-job crafting changes between Waves 3 and 4 positively predicted self-rated health at Wave 4, but we found no such link for those living alone.

Finally, comparing subgroups with and without contractual changes, the overall pattern between job and off-job crafting with self-rated health re-emerged, with only a minor difference between the groups. For both groups, we observed that job crafting changes between Waves 1 and 2 and off-job crafting changes between Waves 3 and 4 positively predicted subsequent self-rated health. For those without contractual changes, we additionally observed that off-job crafting changes between Waves 2 and 3 positively predicted self-rated health at Wave 3, but not for those with contractual changes.

Overall, the standardized estimates for all reported significant relationships remained below 0.30 (range: *β*_JC CS1–SRH2_ = 0.093 (Wave 2, HO new) to *β*_OJC CS3–SRH4_ = 0.242 (Wave 4, P/F)), indicating that we only found small positive predictions of crafting changes regarding self-rated health.

## Discussion

### Changes in Job and Off-Job Crafting: Mean Trends and Variances of Changes, and the Relationship Between Job and Off-Job Crafting Over Time

In our study, we sought to consider non-linear changes in crafting within certain phases of the pandemic, such as from a pre-pandemic phase to the first lockdown (RQ1). We investigated whether strong adaptive responses, e.g. sharp increases or decreases in crafting, might be linked with certain temporal or contextual characteristics (RQ3). Our results show that crafting remained on average relatively stable in both domains – for the full sample and all subgroups. The trajectories of both job and off-job crafting were almost flat and mean trends in change at different time points were negative yet also close to zero. This suggests that employees, on average, have reduced their crafting, which is surprising given that one would expect an increase in crafting to adapt to pandemic challenges. Employees were likely absorbed with managing day-to-day life, leaving no time for crafting. Syrek et al. (2022) found that participants described vividly how managing different roles and increased work-nonwork conflicts was found challenging and stressful, likely leaving little opportunity to address one’s own needs and proactively shape daily life experiences in both domains to support wellbeing. Interestingly, Syrek et al. (2022) found that work-nonwork balance improved towards the end of their study period, which they interpret as an indication that employees quickly developed suitable strategies to handle the pandemic situation. In contrast, our study, covering a longer timeframe, indicates that employees, even in the long run, seemed to have directed their energies less toward proactive crafting and potentially more towards reactively managing daily pandemic life.

However, since we measured crafting on a frequency scale, our study findings may also be interpreted in such that employees retained their crafting frequency or the time spent on crafting over the time span covered in this research (over two years from June 2019 to December 2021), despite the stress of the pandemic (Syrek et al., 2022). individuals only decreased their crafting time to a small extent. However, individuals may have altered how and what they crafted during this period. We found partial metric and scalar measurement invariance in most models, indicating potential changes in prominent crafting strategies or overall strategy usage. The pandemic may have also led to new crafting strategies not captured in current research. Future studies should explore this further.

Further, considering the decreasing trends in crafting (frequency) that we observe in this study, it also leads to the question of whether new or more prominent crafting strategies applied during the pandemic simply require less time or need to be exhibited less often to be deemed fruitful and satisfy the crafter’s needs. For example, before the pandemic, employees might have crafted for social resources through regular interactions, i.e. by joining work breaks. With social contact limited during the pandemic, they may have adapted by meeting less often, likely in virtual settings, and focusing more on the quality of these interactions. This shift may reflect an overall decrease in crafting activity, despite changes in specific strategies. Future research should explore these crafting changes qualitatively for deeper insights.

Two findings highlight the need to closely examine how and why crafting changed during the pandemic. Firstly, there was a slight increase in job crafting from Waves 2 to 3 for the full sample, but not for subgroups (RQ3), which showed no mean trend. Secondly, while overall job and off-job crafting trajectories appeared flat or slightly decreasing, significant variances in change scores indicated individual differences. This suggests that crafting patterns varied even if group-level trends did not show notable differences over time. There is apparently variation in the job and off-job crafting patterns that our analyses did not capture, e.g., by comparing the demographic subgroups in our sample.

Therefore, although we examined group differences for employee subgroups that have been previously discussed as differentially affected by the pandemic (Kaltiainen & Hakanen, 2022; OECD, 2020a, 2020b; Tušl et al., 2021), our results suggest no notable differences in the crafting patterns change over time. Employees’ perceptions of the pandemic and associated life disruptions might better explain fluctuations in their crafting behaviors than demographic factors. While the pandemic itself might be considered a worthy “reason to” engage in crafting, according to the model of proactive behavior (Parker et al., 2010), it may be that employees perceived fewer opportunities to craft due to restrictions (fitting the “can do” motivational state) or lower energy to engage in behavior to promote their own health (fitting the “energized to” motivational state). Future qualitative research could utilize this framework to capture specific perceived motivations and barriers to explain the crafting changes observed in this study. Future research should moreover consider person-centered approaches, e.g., clustering, to identify groups with more similar patterns in crafting over time and relate this to other characteristics, e.g., personality traits.

Lastly, we focus on the connection between job and off-job crafting changes. Our results indicate a consistent, small but positive association between job and off-job crafting changes throughout the pandemic. Therefore, employees decreased or increased their crafting similarly in both domains. This finding supports spillover/congruency theories for crafting (Demerouti et al., 2020), apparently regardless of the pandemic context. However, we found no connection between job and off-job crafting changes between Waves 1 and 2 among those employees experiencing contractual changes (RQ3). Conceivably, this group focused crafting efforts more on one domain temporarily, e.g., by explicitly crafting the job domain to secure a more financial future despite the forced contractual changes. Our results, however, do not support such assumptions of a consistent, temporary compensatory effect between job and off-job crafting. Instead, it seems that affected employees differed greatly in their crafting allocation in this phase, as seen in the non-significant covariance. It remains unclear what identified those crafting to compensate vs. those crafting to a similar extent in this specific phase of the pandemic. Future research could utilize qualitative methods (e.g., interviews) with affected employees to gain insights into the motives and drivers of different strategies, e.g., the pressure of precarious employment and financial issues (OECD, 2020a, 2020b) or positive aspects, such as reported increases in leisure time and time spent with partner/family (Tušl et al., 2021).

To sum up, although the trajectories of job and off-job crafting remained flat for all groups, we observed interindividual changes, and for most groups, the association between changes was weak and positive throughout the pandemic. Therefore, our findings seem to support earlier research suggesting a congruency in crafting across life domains (Demerouti et al., 2020), even when now referring to crafting changes.

### Changes in Crafting and Self-Rated Health

In RQ2, we posed the question of whether we might observe temporal differences in the (positive) associations between crafting changes and subsequent levels of self-rated health during the pandemic. In our study, we investigated how intraindividual changes in crafting during the pandemic may have led to interindividual differences in health outcomes, providing insights into the potential benefits of changing crafting efforts across life domains. We also examined temporal and contextual variations in the relationship between crafting and self-rated health to understand how different individuals have been affected by adaptive adjustments during the pandemic (RQ4).

However, before scrutinizing evidence answering RQ2 and RQ4, we review the development of self-rated health during the pandemic. Our results indicate that self-rated health remained stable during the pandemic for the full sample and all subgroups. Self-rated health is reportedly responsive to changing health-related circumstances (Perruccio et al., 2010). While some individuals affected by COVID-19 reported lower self-rated health (Peters et al., 2020), other studies support our findings of overall stability (Peters et al., 2020; Recchi et al., 2020; Szwarcwald et al., 2021; van de Weijer et al., 2022). Additionally, mental health indicators remained stable (Van Tilburg et al., 2021), and a Finnish study found only minor changes in occupational well-being across demographic groups Finland (Kaltiainen & Hakanen, 2022). Notably, our sample was not significantly impacted by COVID-19, with only 1.70% reporting infections at Wave 3 (see Table 1).

Even though self-rated health remained largely stable, positive – yet weak—associations were discernible between crafting changes and self-rated health (RQ2), that emerged in a noteworthy pattern. Early in the pandemic, we observed positive associations between job crafting increases and self-rated health, whereas later in the pandemic, we observed positive associations between off-job crafting increases and self-rated health. Changes in crafting occurred at all time points, but apparently only those changes in the job domain at the pandemic onset related to subsequent higher levels of self-rated health. Only those changes in the off-job domain towards a normalization phase related to subsequent higher levels of self-rated health, even though both domains were similarly affected by the pandemic (Tušl et al., 2021).

First, this indicates that individual increases in crafting in the job and off-job domain relate to later interindividual differences in health with a temporal shift during the pandemic. Increasing crafting in the job domain in the beginning of the pandemic relates to health-related advantages compared to those employees who retained their level of crafting or decreased it. Similarly, increasing crafting outside the job relates to health-related advantages, compared to employees who retained or decreased their level of crafting. However, this beneficial process seemed to have occurred rather at the end of the pandemic.

It may be that the job domain was more salient for individuals at the beginning of the pandemic. Employment means a source of income, structure, identity, social connections, and development opportunities (Witte, 1999). When employment and pay are threatened, individuals’ quality of life is impaired (Winefield & Tiggemann, 1990). Further, job insecurity increased at the beginning of the pandemic but later decreased again (El Khawli et al., 2022). Therefore, understandably, our results indicate that when the pandemic broke out, increasing crafting in the job domain was related to advantages regarding self-rated health, whereas less so increasing crafting in the off-job domain. However, with this study, we can only reveal this notable pattern, but not potential causes. Future research should first aim to corroborate such a domain shift in this association in other changing circumstances, e.g., when employees face major organizational restructuring or more individual health crises.

This pattern of a domain shift in the association between crafting changes and self-rated health was seen in all subgroup analyses, except for employees living alone (RQ4). For these, we found no association between off-job crafting changes and self-rated health. However, we observed a positive association between self-reported belonging to a high-risk group and self-rated health. Potentially, for this group, perceived high risks of severe complications with a COVID-19 infection overshadowed individual crafting efforts when predicting self-rated health. Future research should investigate barriers between individual off-job crafting and self-rated health for those with major salient health risks.

### Strengths and Limitations

To the best of our knowledge, our study is the first to examine the changes in crafting in different life domains during the COVID-19 pandemic and how crafting changes relate to self-rated health. Our results contribute to the knowledge of crafting as a protective factor for health during the past pandemic. In future crises, this individual strategy could be promoted to equip individuals with knowledge, skills, and opportunities to maintain their own health. Further, we note our relatively large and representative sample from Germany, Austria, and Switzerland, which we have followed for several years. This rich dataset, let us examine changes over a significant part of the pandemic and also include pre-pandemic and more stable phases.

However, our study also has several limitations. First, we encountered issues in establishing metric measurement invariance (equal factor loadings) for job crafting. These made it impossible to examine differences in the dynamic association of job and off-job crafting and also with self-rated health depending on individual caregiving duties for others (further information: see methods). For this set of subgroups, we could not establish even partial metric measurement for job crafting for subgroups with caregiving duties, a relevant criterion to correctly interpret longitudinal associations and differences in latent constructs (Putnick & Bornstein, 2016). Job crafting may mean something slightly different to an individual depending on the life stage (e.g., young parent vs. older parent) or life circumstances (e.g., children vs. no children). Differences in the understanding of job crafting by specific demographic groups should be considered and investigated more thoroughly in future research (e.g., Vignoli et al., 2023). For transparency and replicability, we provide our analyses in ESM 5.

Second, we were overall only able to establish partial scalar measurement invariance (equal item/item parcel intercepts) for job and off-job crafting in all analyses, and above that, we were only able to establish partial metric measurement invariance for job crafting for our group comparison based on contractual changes. Given the complexity of our analyses with four time points over a long period and several multigroup analyses, it is unsurprising that rigorous assumptions for measurement invariance cannot be met. Both job and off-job are constituted by four respective six facets, and it is likely that the overall composition of crafting changes over time. Such a change would indicate a shift regarding the main crafting strategy within a domain. Moreover, the overall composition of job crafting strategies may differ among employees with and without contractual changes. In our analyses, we released the factor loading from “increasing social job resources” to the overall job crafting factor. Those with contractual changes likely had less need or opportunity to contact colleagues and supervisors, therefore relying less on this job crafting strategy. Future research should consider whether the composition of job crafting strategies also changes in other similar circumstances, e.g., during organizational changes.

In general, our analyses focused on overall job and off-job crafting trends, not specific facets. Therefore, we argue that combining job and off-job crafting into single factors and allowing for partial measurement invariance is an appropriate strategy to handle complexities. Further, we suggest that the differences in the measurement model emerging due to partial measurement invariance should be relatively minor, as differences, e.g., in item intercepts were close to zero between a fixed and freely estimated model, and results are still interpretable. We compiled model fit indices and comparison tests regarding measurement invariance in ESM 1.

Lastly, our study focused specifically on the pandemic, and our results cannot be generalized to other crises (e.g., financial crises, natural disasters, war). Moreover, our sample consists solely of participants from Germany, Austria, and Switzerland, so generalizations to other socioeconomic and political settings are not advisable. Future research could investigate whether intraindividual changes can also be observed over time outside the pandemic context and which other larger changes, e.g., major organizational transformations, may also involve changes in crafting of a whole population.

### Practical Implications

Political and organizational institutions have a dual responsibility during crises—implementing measures to contain the spread of the virus and protecting individuals while also supporting their health. Our study reveals that individuals can benefit from increasing their crafting efforts in terms of self-rated health, mainly at the beginning and end of the pandemic. This has three important implications for practice. First, increasing crafting might be encouraged during times of crisis. Previous research has demonstrated the use and effectiveness of crafting interventions (Petrou & de Vries, 2023; Roczniewska et al., 2023; van den Heuvel et al., 2015; van Wingerden et al., 2017). Institutions, organizations, and political bodies might incorporate crafting interventions in future crisis mitigation plans to support employees’ health. Second, to minimize health differences between individuals emerging during crises, those individuals who did not increase their crafting might be considered a risk group needing further support. Within this study, we found no links to demographic characteristics to help identify such individuals. We encourage organizations and leaders to engage in conversations with their employees specifically to understand additional needs for support, induced e.g. by increased care duties or reduced contact with coworkers, and to offer targeted support, e.g., by increasing social support or help identifying further institutional offers such as additional childcare (for further suggestions, see, e.g.: Bernhardt et al., 2023; Greer et al., 2023; Pichler et al., 2023).

Third, we found that for those living alone, changes in off-job crafting at later pandemic phases did not predict self-rated health, and self-reported belonging to a high-risk group potentially overshadowed individual efforts to meet one’s needs. These employees may need support to stay healthy in a crisis like the COVID-19 pandemic. These employees risked severe isolation from usual crafting opportunities outside the job, as they adhered strictly to political social distancing measures to minimize the health risks. Employers and main leisure sites, e.g., sports clubs, could offer targeted consultations in form of crafting interventions to help these individuals overcome perceived barriers to their needs satisfaction. Employers could consider how affected individuals could achieve detachment and relaxation by exploring new activities, while main leisure sites might need to consider ways of allowing such individuals to participate safely in relevant activities, e.g., by continuing outdoor sports activities.

## Conclusion

Although the mean trajectories of change in crafting and self-rated health during the pandemic remained flat, individuals did change their crafting during the pandemic. Further, their crafting changes were positively associated with later self-reported health, highlighting how increasing crafting relates to broader interindividual health differences over time. We note a temporal amplification of specific life domains in explaining associations between crafting changes and self-rated health. First, job crafting changes – but not off-job crafting changes – predict self-rated health, while later in the pandemic, the relationship is reversed. However, it remains unclear which employees increased their crafting, and why others decreased it. We call for future research to investigate perceived barriers more closely to better support employees in maintaining their health in times of crisis. Overall, we suggest that as an individual strategy for maintaining health during crises, crafting complements organizational and public health measures designed to support individuals in challenging times. Employees might benefit from crafting interventions providing them with the knowledge and skills to promote their own health, making the workforce more resilient in future crises.

## Supplementary Information

Below is the link to the electronic supplementary material.

Supplementary file1 (PDF 43 KB)

Supplementary file2 (PDF 61 KB)

Supplementary file3 (PDF 77 KB)

Supplementary file4 (HTML 888 KB)

Supplementary file5 (HTML 783 KB)

Supplementary file6 (XLSX 21 KB)

## Data Availability

The data that support the findings of this study are available from the authors upon reasonable request. Study participants were asked to give consent to use the data for research and within research publication, but not for open public access. We provide additional material, including analysis syntax, in the electronic supplementary materials ESM 1–5.

## Footnotes

1 While earlier research has investigated crafting outside the job domain, often framed within the JD-R model resources (e.g., as home or leisure crafting; see: Demerouti et al., 2020; Haun et al., 2022; Petrou et al., 2017; Petrou & Bakker, 2016), we focus on needs-based off-job crafting. Unlike job crafting, which often involves concrete efforts like asking for feedback (Tims et al., 2012), off-job crafting efforts vary widely, such as in different leisure activities. Despite these differences in crafting efforts, individuals may still aim to satisfy underlying psychological needs (de Bloom et al., 2020; Kujanpää et al., 2022). Additionally, from an approach-avoidance perspective (Bruning & Campion, 2018), needs-based off-job crafting aligns with JD-R concepts: detachment efforts correspond to avoidance of demands, while crafting for mastery or autonomy can be viewed as approach strategies aimed at increasing resources. This study combines needs-based off-job crafting with the JD-R framework to explore crafting across life domains, drawing on the most inclusive perspectives available. However, we acknowledge that this is still an emerging research field, with initial efforts to increase the inclusivity of job crafting research through frameworks like the DRAMMA-based conceptualization (Tušl et al., 2024). To further understand how crafting across life domains connects from a theoretical perspective, more research that falls outside the scope of this study is needed.

2 We note, however, that our study design would not allow us to examine actual spillover processes in crafting between the life domains, as we focus on cross-sectional interrelations between crafting changes. Other study designs, for example, cross-lagged panel designs (Zyphur et al., 2020) or continuous time modelling (Ryan et al., 2018; van Montfort et al., 2018) would be more appropriate to uncover spillover processes and examine temporal precedencies in crafting. This study allows us to investigate only whether crafting efforts changed congruently across life domains from a cross-sectional perspective.

3 We thank an anonymous reviewer for pointing out existing ambiguities regarding the distinction between off-job crafting and recovery experiences. Recovery experiences are part of the six psychological needs that relate to subjective well-being (D. B. Newman et al., 2014). While the measurement for recovery experiences targets whether individuals experienced certain states, e.g., not having thought about work, off-job crafting captures the proactive and intentional actions by individuals toward satisfying specific recovery needs (Kujanpää & Olafsen, 2024; Kujanpää et al., 2022; Sonnentag & Fritz, 2007). Consequently, there is a notable difference in how these constructs are typically assessed. For example, experience of detachment is usually assessed as “I don’t think about work at all.” or “I distance myself from my work.” (Sonnentag & Fritz, 2007), whereas off-job crafting for detachment is assessed as “I’ve made sure to detach from work-related thoughts during off-job time.” or “I’ve arranged my off-job time so that I distance myself from work-related tasks.” (Kujanpää et al., 2022). Within recovery experiences, active aspects might also be integrated, as distancing oneself from work is an active form of effort. At the time of conducting this research and writing this study, the presented measure for off-job crafting is – in our opinion – the best available, as it most inclusively captures proactive efforts within the off-job domain compared to earlier conceptualizations closely mirroring the JD-R model (e.g., home or leisure crafting; Demerouti et al., 2020; Petrou et al., 2017). However, we conclude that further disentangling recovery experiences and off-job crafting, is a fruitful research field that will aid our understanding of recovery from work.

4 We initially planned to review differences in job and off-job crafting changes for subgroups also referring to reported caregiving duties (Groups: No caregiving duties (*n* = 342), Caregiving duties for children under 11 (*n* = 110), Caregiving duties for older children & adults (*n* = 331)). However, we could not establish (metric) measurement invariance for job crafting for all three groups and only partial metric measurement invariance when excluding the subgroup without caregiving duties. Possibly due to the resulting reduced sample size, a group comparison with only two subgroups having caregiving duties did not work, as the model did not converge properly due to Heywood cases. We explored other ways of splitting the sample regarding caregiving duties, but the group without caregiving duties continued to prove problematic. We suggest that the issue of measurement variance for job crafting should be further investigated in crafting research. Job crafting may be understood differentially by employees depending on their other life obligations as seen here with caregiving duties. We note, however, that our study focuses on the pandemic situation. Therefore, issues of measurement invariance for job crafting emerging outside the pandemic context also merit investigation (see, e.g., Vignoli et al., 2023). We decided to treat caregiving duties as a demographic distal to the other groups and report them in Table 1 with other demographics. For transparency, we included the respective R code for analysis in ESM 5 (available online).

5 Full model results are available in the supplementary materials ESM 3. Please note, that we overall adhere to the SEM coefficient notation as described by Bollen (1989). In ESM 3, we included the notation for all reported coefficients.
