# Supplementary material for: Crafting for Health: A Longitudinal Study of Job and Off-Job Crafting Changes during the COVID-19 Pandemic
Source: Occup Health Sci. 2025 Feb 26;9(3):675–710. doi: 10.1007/s41542-025-00222-5 (PMC12484252; doi:10.1007/s41542-025-00222-5)
Supplement: Supplementary file 1 — Supplementary file1 (PDF 43 KB) [file 41542_2025_222_MOESM1_ESM.pdf]

Overview of model comparison tests for measurement invariance

Longitudinal measurement invariance for the full sample

Model	Chisq	df	p	CFI	TLI	RMSEA	SRMR	Model comparison	Chisq difference test	Comment
M1 - Job crafting: Configural invariance	16.493	8	0.036	0.994	0.982	0.043	0.012			
M2 - Job Crafting: Metric invariance	19.71	17	0.289	0.998	0.997	0.017	0.016	M1 vs M2	3.2169 (9) ns	
M3 - Job Crafting: Scalar invariance	50.079	26	0.003	0.983	0.985	0.041	0.032	M2 vs M3	30.3690 (9) ***	
M3a -Job Crafting: Partial scalar invariance	24.061	20	0.24	0.997	0.997	0.019	0.019	M2 vs M3a	4.3519 (3) ns	Intercepts of both "Reducing challenging demands" and "Increasing structural ressources" freed across time
M4 - Off-job crafting: Configural invariance	494.21	32	0	0.925	0.86	0.16	0.049			
M5 - Off-job Crafting: Metric invariance	504.17	47	0	0.926	0.906	0.131	0.051	M4 vs M5	9.9583 (15) ns	
M6 - Off-job Crafting: Scalar invariance	533.16	62	0	0.924	0.926	0.116	0.054	M5 vs M6	28.9904 (15) *	
M6a -Off-job Crafting: Partial scalar invariance	521.38	59	0	0.925	0.924	0.118	0.053	M5 vs M6a	17.215 (12) ns	Intercept of "Crafting for affiliation" freed across time

Longitudinal measurement invariance per subgroup

Model	Chisq	df	p	CFI	TLI	RMSEA	SRMR	Model comparison	Chisq difference test	Comment
GC1: HO new										
M7-1 - Job crafting: Configural invariance	11.699	8	0.165	0.979	0.936	0.063	0.027			
M8-1 - Job Crafting: Metric invariance	14.721	17	0.616	1	1.019	0	0.035	M7-1 vs M8-1	3.0224 (9) ns	
M9-1 - Job Crafting: Scalar invariance	26.681	26	0.426	0.996	0.996	0.015	0.053	M8-1 vs M9-1	11.9598 (9) ns	
M10-1 - Off-job crafting: Configural invariance	115.88	32	0	0.93	0.869	0.15	0.053			
M11-1 - Off-job Crafting: Metric invariance	124.5	47	0	0.935	0.917	0.119	0.06	M11-1 vs M12-1	8.6215 (15) ns	
M12-1 - Off-job Crafting: Scalar invariance	145.52	62	0	0.93	0.933	0.107	0.069	M12-1 vs M13-1	21-0197 (15) ns	
GC1: FOW										
M7-2 - Job crafting: Configural invariance	18.686	8	0.017	0.986	0.959	0.068	0.022			
M8-2 - Job Crafting: Metric invariance	23.251	17	0.141	0.992	0.989	0.035	0.028	M7-2 vs M8-2	4.5654 (9) ns	
M9-2 - Job Crafting: Scalar invariance	46.547	26	0.008	0.974	0.976	0.052	0.042	M8-2 vs M9-2	23.2955 (9) **	
M9a-2 - Job Crafting: Partial scalar invariance	27.873	20	0.112	0.99	0.988	0.037	0.03	M8-2 vs M9a-2	4.6217 (3) ns	Intercepts of both "Reducing challenging demands" and "Increasing structural ressources" freed across time
M10-2 - Off-job crafting: Configural invariance	303.75	32	0	0.916	0.843	0.171	0.053			
M11-2 - Off-job Crafting: Metric invariance	311.72	47	0	0.918	0.896	0.139	0.055	M11-2 vs M12-2	7.9698 (15) ns	
M12-2 - Off-job Crafting: Scalar invariance	331.2	62	0	0.917	0.92	0.122	0.058	M12-2 vs M13-2	19.4831 (15) ns	
GC1: HO exp										
M7-3 - Job crafting: Configural invariance	13.912	8	0.084	0.982	0.946	0.074	0.028			
M8-3 - Job Crafting: Metric invariance	18.886	17	0.335	0.994	0.992	0.029	0.043	M7-3 vs M8-3	4.9739 (9) ns	
M9-3 - Job Crafting: Scalar invariance	24.986	26	0.52	1	1.003	0	0.049	M8-3 vs M9-3	6.0996 (9) ns	
M10-3 - Off-job crafting: Configural invariance	146.46	32	0	0.928	0.865	0.164	0.055			
M11-3 - Off-job Crafting: Metric invariance	165.3	47	0	0.926	0.905	0.137	0.077	M11-3 vs M12-3	18.8370 (15) ns	
M12-3 - Off-job Crafting: Scalar invariance	174.58	62	0	0.929	0.932	0.117	0.081	M12-3 vs M13-3	9.2832 (15) ns	
GC2: P/F										
M7-4 - Job crafting: Configural invariance	8.372	8	0.398	1	0.999	0.011	0.012			
M8-4 - Job Crafting: Metric invariance	16.013	17	0.523	1	1.001	0	0.025	M7-4 vs M8-4	7.641 (9) ns	
M9-4 - Job Crafting: Scalar invariance	49.325	26	0.004	0.978	0.979	0.048	0.041	M8-4 vs M9-4	33.313 (9) ***	
M9a-4 - Job Crafting: Partial scalar invariance	20.518	20	0.426	1	0.999	0.008	0.027	M8-4 vs M9a-4	4.5051 (3) ns	Intercepts of both "Reducing challenging demands" and "Increasing structural ressources" freed across time
M10-4 - Off-job crafting: Configural invariance	323.41	32	0	0.932	0.873	0.154	0.047			
M11-4 - Off-job Crafting: Metric invariance	335.78	47	0	0.933	0.915	0.127	0.052	M11-4 vs M12-4	12.365 (15) ns	
M12-4 - Off-job Crafting: Scalar invariance	355.96	62	0	0.932	0.934	0.111	0.054	M12-4 vs M13-4	20.184 (15) ns	
GC2: Alone										
M7-5 - Job crafting: Configural invariance										Did not converge properly
M8-5 - Job Crafting: Metric invariance	38.453	17	0.002	0.942	0.918	0.089	0.052			
M9-5 - Job Crafting: Scalar invariance	42.881	26	0.02	0.954	0.958	0.064	0.057	M8-5 vs M9-5	4.4275 (9) ns	
M10-5 - Off-job crafting: Configural invariance	156.41	32	0	0.927	0.863	0.157	0.048			
M11-5 - Off-job Crafting: Metric invariance	168.36	47	0	0.929	0.909	0.128	0.062	M11-5 vs M12-5	11.945 (15) ns	
M12-5 - Off-job Crafting: Scalar invariance	188.25	62	0	0.926	0.928	0.113	0.067	M12-5 vs M13-5	19.894 (15) ns	
GC4: NC										
M7-6 - Job crafting: Configural invariance	10.632	8	0.223	0.998	0.993	0.028	0.012			
M8-6 - Job Crafting: Metric invariance	13.226	17	0.721	1	1.005	0	0.018	M7-6 vs M8-6	2.5936 (9) ns	
M9-6 - Job Crafting: Scalar invariance	42.207	26	0.023	0.985	0.986	0.038	0.035	M8-6 vs M9-6	28.9809 (9) ***	
M9a-6 - Job Crafting: Partial scalar invariance	15.528	20	0.745	1	1.005	0	0.019	M8-6 vs M9a-6	2.3018 (3) ns	
M10-6 - Off-job crafting: Configural invariance	365.37	32	0	0.927	0.863	0.157	0.049			
M11-6 - Off-job Crafting: Metric invariance	375.05	47	0	0.928	0.908	0.128	0.052	M11-6 vs M12-6	9.6874 (15) ns	
M12-6 - Off-job Crafting: Scalar invariance	399.59	62	0	0.926	0.928	0.113	0.055	M12-6 vs M13-6	24.5358 (15) ns	
GC4: CC										
M7-7 - Job crafting: Configural invariance	13.493	8	0.096	0.985	0.954	0.071	0.03			
M8-7 - Job Crafting: Metric invariance	27.617	17	0.05	0.97	0.958	0.068	0.052	M7-7 vs M8-7	14.1235 (9) ns	
M9-7 - Job Crafting: Scalar invariance	34.084	26	0.133	0.977	0.979	0.048	0.058	M8-7 vs M9-7	6.4676 (9) ns	
M10-7 - Off-job crafting: Configural invariance	145.77	32	0	0.93	0.868	0.163	0.05			
M11-7 - Off-job Crafting: Metric invariance	155.8	47	0	0.933	0.914	0.131	0.063	M11-7 vs M12-7	10.030 (15) ns	
M12-7 - Off-job Crafting: Scalar invariance	174.71	62	0	0.93	0.932	0.116	0.068	M12-7 vs M13-7	18.907 (15) ns	

Intergroup measurement invariance for wave 4

Model	Chisq	df	p	CFI	TLI	RMSEA	SRMR	Model comparison	Chisq difference test	Comment
GC1										
M13-1 - Job crafting: Configural invariance	6.876	6	0.332	0.998	0.994	0.024	0.017			
M14-1 - Job Crafting: Metric invariance	14.737	12	0.256	0.994	0.991	0.03	0.033	M13-1 vs M14-1	7.8603 (6) ns	
M15-1 - Job Crafting: Scalar invariance	23.935	18	0.157	0.987	0.987	0.036	0.044	M14-1 vs M15-1	9.1979 (6) ns	
M16-1 - Off-job crafting: Configural invariance	164.52	24	0	0.927	0.864	0.153	0.048			
M17-1 - Off-job Crafting: Metric invariance	176.23	34	0	0.926	0.903	0.129	0.057	M16-1 vs M17-1	11.706 (10) ns	
M18-1 - Off-job Crafting: Scalar invariance	192.24	44	0	0.923	0.922	0.116	0.061	M17-1 vs M18-1	16.013 (10) ns	
GC2										
M13-2 - Job crafting: Configural invariance	9.001	4	0.061	0.99	0.97	0.058	0.018			
M14-2 - Job Crafting: Metric invariance	10.27	7	0.174	0.994	0.989	0.035	0.022	M13-2 vs M14-2	1.2691 (3) ns	
M15-2 - Job Crafting: Scalar invariance	12.273	10	0.267	0.996	0.995	0.025	0.024	M14-2 vs M15-2	2.0030 (3) ns	
M16-2 - Off-job crafting: Configural invariance	103.19	16	0	0.955	0.916	0.121	0.038			
M17-2 - Off-job Crafting: Metric invariance	107.73	21	0	0.955	0.936	0.105	0.043	M16-2 vs M17-2	4.538 (5) ns	
M18-2 - Off-job Crafting: Scalar invariance	154.01	26	0	0.934	0.924	0.115	0.056	M17-2 vs M18-2	46.282 (5) ***	
M18a-2 - Off-job Crafting: Partial scalar invariance	111.91	24	0	0.955	0.943	0.099	0.045	M17-2 vs M18a-2	4.187 (3) ns	Intercepts of "Crafting for Autonomy" and "Crafting for Affiliation" freed
GC4										
M13-3 - Job crafting: Configural invariance	4.295	4	0.367	0.999	0.998	0.014	0.011			
M14-3 - Job Crafting: Metric invariance	15.05	7	0.035	0.984	0.973	0.054	0.027	M13-3 vs M14-3	10.7549 (3) *	
M15-3 - Job Crafting: Scalar invariance	23.179	10	0.01	0.974	0.969	0.058	0.034	M14-3 vs M15-3	8.1283 (3) *	
M14a-3 - Job Crafting: Partial metric invariance	5.776	6	0.449	1	1.001	0	0.015	M13-3 vs M14a-3	1.481 (2) ns	Factor loading from parcel "Increasing social resources" to JC factor freed
M15a-3 - Job Crafting: Scalar invariance with partial metric invariance	16.962	9	0.049	0.985	0.979	0.048	0.03	M14a-3 vs M15a-3	11.186 (3) *	Factor loading from parcel "Increasing social resources" to JC factor freed
M15b-3 - Job Crafting: Partial scalar and metric invariance	7.868	8	0.446	1	1	0	0.018	M14a-3 vs M15b-3	2.9092 (2) ns	Intercept of "Reducing hindering job demands" freed
M16-3 - Off-job crafting: Configural invariance	116.68	16	0	0.949	0.904	0.128	0.041			
M17-3 - Off-job Crafting: Metric invariance	119.73	21	0	0.95	0.928	0.11	0.043	M16-3 vs M17-3	3.0498 (5) ns	
M18-3 - Off-job Crafting: Scalar invariance	128.13	26	0	0.948	0.94	0.101	0.046	M17-3 vs M18-3	8.3986 (5) ns	
