# Supplementary material for: Crafting for Health: A Longitudinal Study of Job and Off-Job Crafting Changes during the COVID-19 Pandemic
Source: Occup Health Sci. 2025 Feb 26;9(3):675–710. doi: 10.1007/s41542-025-00222-5 (PMC12484252; doi:10.1007/s41542-025-00222-5)
Supplement: Supplementary file 2 — Supplementary file2 (PDF 61 KB) [file 41542_2025_222_MOESM2_ESM.pdf]

Model fits SRH

Model No	Description	chisq	df	pvalue	cfi	tli	rmsea	srmr	Model comparison	Chisq difference test	Comment
	**Full Sample**										
M1	Base model	2002.710	870	0	0.928	0.918	0.041	0.076			
**M2**	**Equality constraint (EC) on change score covariances across time**	**2004.173**	**872**	**0**	**0.928**	**0.918**	**0.041**	**0.076**	M1 vs M2	1.4625 (2) ns	Final model
	**Group Comparison 1: Work Location**										
M3	Base model	4852.974	2658	0	0.864	0.848	0.057	0.093			Heywood Case
M4	EC on autoregressive paths across groups	4863.575	2664	0	0.864	0.848	0.057	0.093	M3 vs M4	10.5601 (6) ns	EC retained
M5	EC on regression from control variable to outcome across groups	4867.809	2670	0	0.864	0.849	0.057	0.093	M4 vs M5	4.2333 (6) ns	EC retained
M6	EC on change score covariances across groups	4876.998	2676	0	0.864	0.849	0.057	0.093	M5 vs M6	9.1893(6) ns	EC retained
M7	EC on JC change score regressions to outcome across groups	4878.461	2682	0	0.864	0.849	0.057	0.093	M6 vs M7	1.4627 (6) ns	EC retained
M8	EC on OJC change score regressions to outcome across groups	4884.100	2688	0	0.864	0.85	0.057	0.093	M7 vs M8	5.6398 (6) ns	EC retained
M9	EC on change score covariances across time	4886.267	2690	0	0.864	0.85	0.057	0.093	M8 vs M9	2.1671 (2) ns	EC retained
M10	EC on JC change score intercepts across groups for first time interval	4888.712	2692	0	0.864	0.85	0.057	0.093	M9 vs M10	2.4443 (2) ns	EC retained
M11	EC on JC change score intercepts across groups for first and second time interval	4894.492	2694	0	0.864	0.85	0.057	0.093	M10 vs M11	5.7801 (2) ns	EC retained
M12	EC on JC change score intercepts across groups for all time intervals	4894.749	2696	0	0.864	0.85	0.057	0.093	M11 vs M12	0.25671 (2) ns	EC retained
M13	EC on OJC change score intercepts across groups for first time interval	4906.548	2698	0	0.863	0.849	0.057	0.093	M12 vs M13	11.8 (2) **	EC rejected
M14	EC on OJC change score intercepts across groups for second time interval	4902.292	2698	0	0.863	0.85	0.057	0.093	M12 vs M14	7.5438 (2) *	EC rejected
**M15**	**EC on OJC change score intercepts across groups for third time interval**	**4895.312**	**2698**	**0**	**0.864**	**0.85**	**0.057**	**0.093**	M12 vs M15	0.56301 (2) ns	Final model
	**Group Comparison 2: Living Situation**										
M16	Base model	3328.853	1763	0	0.9	0.888	0.049	0.086			
M17	EC on autoregressive paths across groups	3332.993	1766	0	0.9	0.888	0.049	0.086	M16 vs M17	4.1399 (3) ns	EC retained
M18	EC on regression from control variable to outcome across groups	3343.557	1769	0	0.9	0.888	0.049	0.086	M17 vs M18	10.564 (3) *	EC rejected
M19	EC on regression from control variable to outcome across groups to SRH W2	3334.765	1767	0	0.9	0.888	0.049	0.086	M16 vs M19	1.7721(1) ns	EC retained
									M19 vs M20	0.39049 (1) ns	EC retained; Difference for W4 (EC
M20	EC on regression from control variable to outcome across groups to SRH W2 and W	3335.155	1768	0	0.9	0.888	0.049	0.086	M20 vs M18	8.4016 (1) **	rejected)
M21	EC on change score covariances across groups	3340.963	1771	0	0.9	0.888	0.049	0.086	M20 vs M21	5.8073 (3) ns	EC retained
M22	EC on JC change score regressions to outcome across groups	3346.355	1774	0	0.9	0.888	0.049	0.086	M21 vs M22	5.3928 (3) ns	EC retained
M23	EC on OJC change score regressions to outcome across groups	3355.558	1777	0	0.9	0.888	0.049	0.086	M22 vs M23	9.2022 (3) *	EC rejected
M24	EC on OJC change score regressions to outcome across groups to SRH W2	3350.045	1775	0	0.9	0.888	0.049	0.086	M22 vs M24	3.6899 (1) ns	EC retained
									M24 vs M25	0.0012311 (1) ns	EC retained; Difference for W4 (EC
M25	EC on OJC change score regressions to outcome across groups to SRH W2 and W3	3350.046	1776	0	0.9	0.888	0.049	0.086	M25 vs M23	5.5111 (1) *	rejected)
M26	EC on change score covariances across time	3352.263	1778	0	0.9	0.888	0.048	0.086	M25 vs M26	2.2168 (2) ns	EC retained
M27	EC on JC change score intercepts across groups for first time interval	3352.270	1779	0	0.9	0.889	0.048	0.086	M26 vs M27	0.0064154 (1) ns	EC retained
M28	EC on JC change score intercepts across groups for first and second time interval	3355.736	1780	0	0.9	0.888	0.048	0.086	M27 vs M28	3.4667 (1) ns	EC retained
M29	EC on JC change score intercepts across groups for all time intervals	3356.344	1781	0	0.9	0.889	0.048	0.086	M28 vs M29	0.60741 (1) ns	EC retained
M30	EC on OJC change score intercepts across groups for first time interval	3356.564	1782	0	0.9	0.889	0.048	0.086	M29 vs M30	0.21974 (1) ns	EC retained
M31	EC on OJC change score intercepts across groups for first and second time interval	3356.986	1783	0	0.9	0.889	0.048	0.086	M30 vs M31	0.4221 (1) ns	EC retained
**M32**	**EC on OJC change score intercepts across groups for all time intervals**	**3357.756**	**1784**	**0**	**0.9**	**0.889**	**0.048**	**0.086**	M31 vs M32	0.77022 (1) ns	Final model
	**Group Comparison 4: Contractual Changes**										
M33	Base model	3212.935	1743	0	0.909	0.896	0.047	0.085			Heywood Case
M34	EC on autoregressive paths across groups	3213.653	1746	0	0.909	0.897	0.047	0.085	M33 vs M34	0.71767 (3) ns	EC retained
M35	EC on regression from control variable to outcome across groups	3217.966	1749	0	0.909	0.897	0.047	0.085	M34 vs M35	4.3129 (3) ns	EC retained
M36	EC on change score covariances across groups	3226.989	1752	0	0.908	0.897	0.047	0.085	M35 vs M36	9.0238 (3) *	EC rejected
M37	EC on change score covariances across groups for first time interval	3223.770	1750	0	0.909	0.897	0.047	0.085	M35 vs M37	5.8043 (1) *	EC rejected
M38	EC on change score covariances across groups for second time interval	3220.964	1750	0	0.909	0.897	0.047	0.085	M35 vs M38	2.9979 (1) ns	EC retained
M39	EC on change score covariances across groups for second and third time interval	3222.080	1751	0	0.909	0.897	0.047	0.085	M38 vs M39	1.1162 (1) ns	EC retained
M40	EC on JC change score regressions to outcome across groups	3222.286	1754	0	0.909	0.897	0.046	0.085	M39 vs M40	0.20611 (3) ns	EC retained
M41	EC on OJC change score regressions to outcome across groups	3224.473	1757	0	0.909	0.897	0.046	0.085	M40 vs M41	2.187 (3) ns	EC rejected due to Heywood Case
M42	EC on OJC change score regressions to outcome across groups to SRH W2	3222.629	1755	0	0.909	0.897	0.046	0.085	M40 vs M42	0.34363 (1) ns	EC retained
M43	EC on OJC change score regressions to outcome across groups to SRH W2 and W3	3224.332	1756	0	0.909	0.897	0.046	0.085	M42 vs M43	1.703 (1) ns	EC rejected due to Heywood Case
M44	EC on OJC change score regressions to outcome across groups to SRH W2 and W4	3222.646	1756	0	0.909	0.897	0.046	0.085	M42 vs M44	0.016266 (1) ns	EC retained
											Note: EC not on COV W1-2 for CC group;
M45	EC on change score covariances across time	3222.945	1758	0	0.909	0.898	0.046	0.085	M44 vs M45	0.29882 (2) ns	EC retained
M46	EC on JC change score intercepts across groups for first time interval	3223.253	1759	0	0.909	0.898	0.046	0.085	M45 vs M46	0.30817 (1) ns	EC retained
M47	EC on JC change score intercepts across groups for first and second time interval	3224.004	1760	0	0.909	0.898	0.046	0.085	M46 vs M47	0.7517 (1) ns	EC retained
M48	EC on JC change score intercepts across groups for all time intervals	3225.395	1761	0	0.909	0.898	0.046	0.085	M47 vs M48	1.3905 (1) ns	EC retained
M49	EC on OJC change score intercepts across groups for first time interval	3226.123	1762	0	0.909	0.898	0.046	0.085	M48 vs M49	0.72831 (1) ns	EC retained
M50	EC on OJC change score intercepts across groups for first and second time interval	3226.124	1763	0	0.909	0.898	0.046	0.085	M49 vs M50	0.00080684 (1) ns	EC retained
**M51**	**EC on OJC change score intercepts across groups for all time intervals**	**3226.126**	**1764**	**0**	**0.909**	**0.898**	**0.046**	**0.085**	M50 vs M51	0.0023949 (1) ns	Final model
