# Supplementary material for: Crafting for Health: A Longitudinal Study of Job and Off-Job Crafting Changes during the COVID-19 Pandemic
Source: Occup Health Sci. 2025 Feb 26;9(3):675–710. doi: 10.1007/s41542-025-00222-5 (PMC12484252; doi:10.1007/s41542-025-00222-5)
Supplement: Supplementary file 3 — Supplementary file3 (PDF 77 KB) [file 41542_2025_222_MOESM3_ESM.pdf]

Final LCSM model results

			FULL SAMPLE			GC1: HO new			GC1: FOW			GC1: HO exp			GC2: P/F			GC2: Alone			GC4: NC			GC4: CC		
Parameter	lavaan param	param symbol	EST (SE)	p	std.all	EST (SE)	p	std.all	EST (SE)	p	std.all	EST (SE)	p	std.all	EST (SE)	p	std.all	EST (SE)	p	std.all	EST (SE)	p	std.all	EST (SE)	p	std.all
Intercept Job Crafting (JC) at Wave 1	JC_W1 ~1	xi_{JC1}	2.891 (0.023) ***	0.000	11.055	2.874 (0.029) ***	0.000	13.166	2.835 (0.025) ***	0.000	10.947	3.011 (0.032) ***	0.000	10.698	2.913 (0.024) ***	0.000	11.395	2.868 (0.028) ***	0.000	10.848	2.43 (0.037) ***	0.000	4.318	2.516 (0.057) ***	0.000	5.480
Variance JC at Wave 1	JC_W1 ~~ JC_W1	phi_{JC1}	0.068 (0.011) ***	0.000	1.000	0.048 (0.012) ***	0.000	1.000	0.067 (0.012) ***	0.000	1.000	0.079 (0.017) ***	0.000	1.000	0.065 (0.011) ***	0.000	1.000	0.07 (0.015) ***	0.000	1.000	0.317 (0.038) ***	0.000	1.000	0.211 (0.044) ***	0.000	1.000
Variance JC Change Score Wave 1–2	djc2 ~~ djc2	phi_{JC CS1}	0.024 (0.005) ***	0.000	1.000	0.021 (0.007) **	0.002	1.000	0.027 (0.006) ***	0.000	1.000	0.025 (0.007) ***	0.000	1.000	0.023 (0.005) ***	0.000	1.000	0.023 (0.006) ***	0.000	1.000	0.109 (0.017) ***	0.000	1.000	0.086 (0.022) ***	0.000	1.000
Variance JC Change Score Wave 2–3	djc3 ~~ djc3	phi_{JC CS2}	0.02 (0.004) ***	0.000	1.000	0.018 (0.006) **	0.004	1.000	0.02 (0.005) ***	0.000	1.000	0.016 (0.006) **	0.006	1.000	0.02 (0.004) ***	0.000	1.000	0.02 (0.006) **	0.001	1.000	0.079 (0.014) ***	0.000	1.000	0.121 (0.03) ***	0.000	1.000
Variance JC Change Score Wave 3–4	djc4 ~~ djc4	phi_{JC CS3}	0.029 (0.006) ***	0.000	1.000	0.018 (0.007) **	0.006	1.000	0.036 (0.008) ***	0.000	1.000	0.035 (0.01) ***	0.001	1.000	0.032 (0.007) ***	0.000	1.000	0.023 (0.007) **	0.001	1.000	0.103 (0.02) ***	0.000	1.000	0.17 (0.04) ***	0.000	1.000
Intercept Off–Job Crafting (OJC) at Wave 1	OJC_W1 ~1	xi_{OJC1}	3.939 (0.028) ***	0.000	8.871	3.98 (0.051) ***	0.000	8.393	3.965 (0.033) ***	0.000	9.304	3.903 (0.046) ***	0.000	8.187	3.935 (0.031) ***	0.000	8.762	3.957 (0.039) ***	0.000	8.821	3.943 (0.03) ***	0.000	9.201	3.973 (0.041) ***	0.000	8.018
Variance OJC at Wave 1	OJC_W1 ~~ OJC_W1	phi_{OJC1}	0.197 (0.02) ***	0.000	1.000	0.225 (0.037) ***	0.000	1.000	0.182 (0.022) ***	0.000	1.000	0.227 (0.037) ***	0.000	1.000	0.202 (0.023) ***	0.000	1.000	0.201 (0.031) ***	0.000	1.000	0.184 (0.02) ***	0.000	1.000	0.245 (0.037) ***	0.000	1.000
Variance OJC Change Score Wave 1–2	dojc2 ~~ dojc2	phi_{OJC CS1}	0.174 (0.019) ***	0.000	1.000	0.234 (0.041) ***	0.000	1.000	0.156 (0.021) ***	0.000	1.000	0.158 (0.028) ***	0.000	1.000	0.162 (0.02) ***	0.000	1.000	0.22 (0.035) ***	0.000	1.000	0.17 (0.02) ***	0.000	1.000	0.184 (0.031) ***	0.000	1.000
Variance OJC Change Score Wave 2–3	dojc3 ~~ dojc3	phi_{OJC CS2}	0.171 (0.019) ***	0.000	1.000	0.173 (0.033) ***	0.000	1.000	0.177 (0.024) ***	0.000	1.000	0.156 (0.029) ***	0.000	1.000	0.161 (0.02) ***	0.000	1.000	0.235 (0.038) ***	0.000	1.000	0.16 (0.019) ***	0.000	1.000	0.219 (0.038) ***	0.000	1.000
Variance OJC Change Score Wave 3–4	dojc4 ~~ dojc4	phi_{OJC CS3}	0.137 (0.017) ***	0.000	1.000	0.141 (0.03) ***	0.000	1.000	0.155 (0.024) ***	0.000	1.000	0.078 (0.018) ***	0.000	1.000	0.101 (0.016) ***	0.000	1.000	0.164 (0.03) ***	0.000	1.000	0.132 (0.018) ***	0.000	1.000	0.146 (0.031) ***	0.000	1.000
Intercept JC Change Score Wave 1–2	djc2 ~1	xi_{JC CS1}	−0.044 (0.01) ***	0.000	−0.287	−0.046 (0.01) ***	0.000	−0.321	−0.046 (0.01) ***	0.000	−0.281	−0.046 (0.01) ***	0.000	−0.290	−0.043 (0.01) ***	0.000	−0.282	−0.043 (0.01) ***	0.000	−0.285	−0.032 (0.025)	0.197	−0.098	−0.032 (0.025)	0.197	−0.110
Intercept JC Change Score Wave 2–3	djc3 ~1	xi_{JC CS2}	0.022 (0.01) *	0.025	0.155	0.011 (0.009)	0.243	0.083	0.011 (0.009)	0.243	0.079	0.011 (0.009)	0.243	0.088	0.016 (0.01)	0.093	0.113	0.016 (0.01)	0.093	0.113	−0.002 (0.026)	0.948	−0.006	−0.002 (0.026)	0.948	−0.005
Intercept JC Change Score Wave 3–4	djc4 ~1	xi_{JC CS3}	−0.075 (0.013) ***	0.000	−0.445	−0.065 (0.013) ***	0.000	−0.478	−0.065 (0.013) ***	0.000	−0.343	−0.065 (0.013) ***	0.000	−0.345	−0.064 (0.012) ***	0.000	−0.361	−0.064 (0.012) ***	0.000	−0.428	−0.147 (0.03) ***	0.000	−0.458	−0.147 (0.03) ***	0.000	−0.357
Intercept OJC Change Score Wave 1–2	dojc2 ~1	xi_{OJC CS1}	−0.047 (0.019) *	0.014	−0.113	−0.147 (0.048) **	0.002	−0.305	−0.074 (0.025) **	0.003	−0.187	0.05 (0.037)	0.174	0.125	−0.06 (0.019) **	0.002	−0.149	−0.06 (0.019) **	0.002	−0.128	−0.066 (0.019) ***	0.000	−0.161	−0.066 (0.019) ***	0.000	−0.155
Intercept OJC Change Score Wave 2–3	dojc3 ~1	xi_{OJC CS2}	−0.095 (0.02) ***	0.000	−0.229	0.002 (0.04)	0.958	0.005	−0.097 (0.026) ***	0.000	−0.232	−0.141 (0.036) ***	0.000	−0.358	−0.094 (0.02) ***	0.000	−0.236	−0.094 (0.02) ***	0.000	−0.195	−0.092 (0.02) ***	0.000	−0.230	−0.092 (0.02) ***	0.000	−0.197
Intercept OJC Change Score Wave 3–4	dojc4 ~1	xi_{OJC CS3}	0.028 (0.021)	0.188	0.075	0.036 (0.021)	0.080	0.096	0.036 (0.021)	0.080	0.092	0.036 (0.021)	0.080	0.129	0.037 (0.02)	0.062	0.118	0.037 (0.02)	0.062	0.092	0.03 (0.021)	0.147	0.083	0.03 (0.021)	0.147	0.079
Intercept Self–Rated Health (SRH) at Wave 1	SRH_W1 ~1	xi_{SRH1}	3.599 (0.03) ***	0.000	5.375	3.632 (0.065) ***	0.000	5.583	3.55 (0.041) ***	0.000	5.323	3.665 (0.062) ***	0.000	5.416	3.605 (0.036) ***	0.000	5.319	3.586 (0.056) ***	0.000	5.439	3.597 (0.034) ***	0.000	5.540	3.609 (0.064) ***	0.000	4.861
Intercept SRH at Wave 2	SRH_W2 ~1	eta_{SRH2}	0.269 (0.192)	0.162	0.404	0.247 (0.211)	0.243	0.384	0.258 (0.202)	0.200	0.383	0.179 (0.212)	0.399	0.265	0.342 (0.187)	0.067	0.519	0.34 (0.186)	0.068	0.522	0.283 (0.195)	0.145	0.440	0.208 (0.197)	0.291	0.286
Intercept SRH at Wave 3	SRH_W3 ~1	eta_{SRH3}	0.246 (0.182)	0.177	0.370	0.328 (0.198)	0.098	0.500	0.25 (0.186)	0.180	0.376	0.331 (0.195)	0.089	0.509	0.268 (0.186)	0.149	0.404	0.264 (0.188)	0.159	0.406	0.235 (0.185)	0.204	0.361	0.243 (0.188)	0.196	0.339
Intercept SRH at Wave 4	SRH_W4 ~1	eta_{SRH4}	0.072 (0.214)	0.737	0.108	0.063 (0.236)	0.789	0.102	0.107 (0.22)	0.628	0.156	0.039 (0.236)	0.870	0.061	0.068 (0.22)	0.756	0.103	0.015 (0.223)	0.948	0.022	0.118 (0.213)	0.580	0.179	0.112 (0.216)	0.605	0.167
Autoregression SRH W1–2	SRH_W2 ~ SRH_W1	b_{SRH 1–2}	0.955 (0.05) ***	0.000	0.962	0.963 (0.053) ***	0.000	0.976	0.963 (0.053) ***	0.000	0.953	0.963 (0.053) ***	0.000	0.968	0.936 (0.049) ***	0.000	0.963	0.936 (0.049) ***	0.000	0.947	0.952 (0.051) ***	0.000	0.959	0.952 (0.051) ***	0.000	0.971
Autoregression SRH W2–3	SRH_W3 ~ SRH_W2	b_{SRH 2–3}	0.941 (0.047) ***	0.000	0.940	0.926 (0.049) ***	0.000	0.909	0.926 (0.049) ***	0.000	0.940	0.926 (0.049) ***	0.000	0.960	0.935 (0.048) ***	0.000	0.930	0.935 (0.048) ***	0.000	0.934	0.946 (0.048) ***	0.000	0.935	0.946 (0.048) ***	0.000	0.962
Autoregression SRH W3–4	SRH_W4 ~ SRH_W3	b_{SRH 3–4}	0.958 (0.055) ***	0.000	0.958	0.956 (0.058) ***	0.000	1.007	0.956 (0.058) ***	0.000	0.929	0.956 (0.058) ***	0.000	0.974	0.966 (0.056) ***	0.000	0.963	0.966 (0.056) ***	0.000	0.929	0.949 (0.055) ***	0.000	0.940	0.949 (0.055) ***	0.000	1.015
Covariance JC and OJC Change Scores Wave 1–2	djc2 ~~ dojc2	phi/r_{JC CS1, OJC CS1}	0.008 (0.003) **	0.006	0.125	0.01 (0.003) **	0.001	0.140	0.01 (0.003) **	0.001	0.151	0.01 (0.003) **	0.001	0.154	0.007 (0.003) *	0.015	0.119	0.007 (0.003) *	0.015	0.103	0.025 (0.006) ***	0.000	0.181	−0.018 (0.015)	0.229	−0.145
Covariance JC and OJC Change Scores Wave 2–3	djc3 ~~ dojc3	phi/r_{JC CS2, OJC CS2}	0.008 (0.003) **	0.006	0.138	0.01 (0.003) **	0.001	0.175	0.01 (0.003) **	0.001	0.164	0.01 (0.003) **	0.001	0.196	0.007 (0.003) *	0.015	0.128	0.007 (0.003) *	0.015	0.106	0.025 (0.006) ***	0.000	0.218	0.025 (0.006) ***	0.000	0.152
Covariance JC and OJC Change Scores Wave 3–4	djc4 ~~ dojc4	phi/r_{JC CS3, OJC CS3}	0.008 (0.003) **	0.006	0.129	0.01 (0.003) **	0.001	0.191	0.01 (0.003) **	0.001	0.131	0.01 (0.003) **	0.001	0.185	0.007 (0.003) *	0.015	0.129	0.007 (0.003) *	0.015	0.120	0.025 (0.006) ***	0.000	0.210	0.025 (0.006) ***	0.000	0.156
Regression from JC Change Score W1–2 to SRH W2	SRH_W2 ~ djc2	b/beta_{JC CS1–SRH2}	0.497 (0.203) *	0.015	0.116	0.419 (0.202) *	0.038	0.093	0.419 (0.202) *	0.038	0.102	0.419 (0.202) *	0.038	0.099	0.452 (0.207) *	0.029	0.104	0.452 (0.207) *	0.029	0.105	0.245 (0.095) **	0.010	0.126	0.245 (0.095) **	0.010	0.099
Regression from OJC Change Score W1–2 to SRH W2	SRH_W2 ~ dojc2	b/beta_{OJC CS1–SRH2}	0.087 (0.06)	0.148	0.055	0.107 (0.062)	0.082	0.081	0.107 (0.062)	0.082	0.063	0.107 (0.062)	0.082	0.063	0.115 (0.06)	0.055	0.070	0.115 (0.06)	0.055	0.083	0.095 (0.061)	0.119	0.061	0.095 (0.061)	0.119	0.056
Regression from High Risk Group COVID–19 (W3) to SRH W2	SRH_W2 ~ c137.6	b/beta_{CV3–SRH2}	−0.049 (0.068)	0.471	−0.033	−0.056 (0.07)	0.425	−0.043	−0.056 (0.07)	0.425	−0.037	−0.056 (0.07)	0.425	−0.036	−0.058 (0.067)	0.391	−0.040	−0.058 (0.067)	0.391	−0.037	−0.05 (0.069)	0.469	−0.035	−0.05 (0.069)	0.469	−0.031
Regression from JC Change Score W2–3 to SRH W3	SRH_W3 ~ djc3	b/beta_{JC CS2–SRH3}	0.468 (0.251)	0.062	0.100	0.666 (0.276) *	0.016	0.136	0.666 (0.276) *	0.016	0.141	0.666 (0.276) *	0.016	0.129	0.411 (0.254)	0.107	0.088	0.411 (0.254)	0.107	0.089	0.197 (0.117)	0.092	0.085	0.197 (0.117)	0.092	0.095
Regression from OJC Change Score W2–3 to SRH W3	SRH_W3 ~ dojc3	b/beta_{OJC CS2–SRH3}	0.114 (0.062)	0.064	0.071	0.102 (0.064)	0.110	0.065	0.102 (0.064)	0.110	0.065	0.102 (0.064)	0.110	0.062	0.113 (0.06)	0.060	0.068	0.113 (0.06)	0.060	0.084	0.161 (0.074) *	0.030	0.099	−0.002 (0.1)	0.981	−0.002
Regression from High Risk Group COVID–19 (W3) to SRH W3	SRH_W3 ~ c137.6	b/beta_{CV3–SRH3}	0 (0.064)	0.995	0.000	0.029 (0.066)	0.667	0.021	0.029 (0.066)	0.667	0.019	0.029 (0.066)	0.667	0.019	−0.023 (0.065)	0.722	−0.016	−0.023 (0.065)	0.722	−0.015	0.006 (0.065)	0.923	0.004	0.006 (0.065)	0.923	0.004
Regression from JC Change Score W3–4 to SRH W4	SRH_W4 ~ djc4	b/beta_{JC CS3–SRH4}	−0.026 (0.237)	0.913	−0.007	−0.099 (0.232)	0.671	−0.022	−0.099 (0.232)	0.671	−0.027	−0.099 (0.232)	0.671	−0.029	0.098 (0.241)	0.685	0.026	0.098 (0.241)	0.685	0.022	0.001 (0.108)	0.992	0.001	0.001 (0.108)	0.992	0.001
Regression from OJC Change Score W3–4 to SRH W4	SRH_W4 ~ dojc4	b/beta_{OJC CS3–SRH4}	0.236 (0.088) **	0.007	0.131	0.238 (0.093) *	0.010	0.144	0.238 (0.093) *	0.010	0.137	0.238 (0.093) *	0.010	0.104	0.507 (0.132) ***	0.000	0.242	0.054 (0.144)	0.707	0.032	0.209 (0.087) *	0.017	0.116	0.209 (0.087) *	0.017	0.120
Regression from High Risk Group COVID–19 (W3) to SRH W4	SRH_W4 ~ c137.6	b/beta_{CV3–SRH4}	0.123 (0.075)	0.098	0.084	0.113 (0.077)	0.141	0.089	0.113 (0.077)	0.141	0.074	0.113 (0.077)	0.141	0.078	0.012 (0.083)	0.889	0.008	0.413 (0.14) **	0.003	0.256	0.103 (0.074)	0.167	0.071	0.103 (0.074)	0.167	0.070
Covariance between JC W1 and High Risk Group COVID–19 (W3)	JC_W1 ~~ c137.6	phi_{JC1, CV3}	−0.011 (0.007)	0.104	−0.090	0.001 (0.014)	0.954	0.008	−0.007 (0.009)	0.412	−0.063	−0.021 (0.014)	0.122	−0.174	−0.011 (0.008)	0.145	−0.096	−0.009 (0.012)	0.453	−0.079	−0.02 (0.016)	0.200	−0.080	−0.023 (0.024)	0.328	−0.110
Covariance between OJC W1 and High Risk Group COVID–19 (W3)	OJC_W1 ~~ c137.6	phi_{OJC1, CV3}	0.023 (0.011) *	0.027	0.116	0.056 (0.03)	0.060	0.239	0.014 (0.014)	0.324	0.071	0.027 (0.021)	0.205	0.127	0.024 (0.013)	0.068	0.116	0.007 (0.019)	0.701	0.039	0.029 (0.011) *	0.011	0.150	−0.014 (0.026)	0.577	−0.064
Covariance between SRH W1 and High Risk Group COVID–19 (W3)	SRH_W1 ~~ c137.6	phi_{SRH1, CV3}	−0.104 (0.017) ***	0.000	−0.343	−0.137 (0.038) ***	0.000	−0.429	−0.111 (0.022) ***	0.000	−0.374	−0.073 (0.031) *	0.021	−0.245	−0.107 (0.02) ***	0.000	−0.340	−0.077 (0.028) **	0.005	−0.279	−0.095 (0.019) ***	0.000	−0.323	−0.153 (0.037) ***	0.000	−0.451
Covariance between JC Change Score W1–2 and High Risk Group COVID–19 (W3)	djc2 ~~ c137.6	phi_{JC CS1, CV3}	0.001 (0.005)	0.771	0.020	0.001 (0.011)	0.918	0.017	−0.005 (0.007)	0.424	−0.072	0.012 (0.009)	0.198	0.176	0.001 (0.006)	0.818	0.018	0.002 (0.009)	0.843	0.027	0.005 (0.011)	0.669	0.032	−0.004 (0.017)	0.834	−0.027
Covariance between JC Change Score W2–3 and High Risk Group COVID–19 (W3)	djc3 ~~ c137.6	phi_{JC CS2, CV3}	−0.009 (0.004) *	0.034	−0.144	−0.008 (0.01)	0.423	−0.125	−0.01 (0.006)	0.097	−0.157	−0.012 (0.008)	0.136	−0.215	−0.014 (0.005) **	0.010	−0.211	−0.002 (0.008)	0.773	−0.037	−0.016 (0.01)	0.097	−0.128	−0.039 (0.02)	0.055	−0.246
Covariance between JC Change Score W3–4 and High Risk Group COVID–19 (W3)	djc4 ~~ c137.6	phi_{JC CS3, CV3}	0.001 (0.005)	0.877	0.011	−0.013 (0.011)	0.240	−0.197	0.008 (0.008)	0.309	0.100	−0.002 (0.01)	0.877	−0.019	0.011 (0.007)	0.105	0.137	−0.017 (0.009) *	0.044	−0.275	0.005 (0.012)	0.661	0.036	0.006 (0.024)	0.813	0.030
Covariance between OJC Change Score W1–2 and High Risk Group COVID–19 (W3)	dojc2 ~~ c137.6	phi_{OJC CS1, CV3}	−0.005 (0.01)	0.612	−0.027	0.01 (0.029)	0.723	0.043	0.009 (0.013)	0.492	0.052	−0.045 (0.021) *	0.027	−0.261	−0.008 (0.012)	0.518	−0.043	0.006 (0.02)	0.764	0.031	−0.002 (0.011)	0.833	−0.013	0.003 (0.022)	0.910	0.013
Covariance between OJC Change Score W2–3 and High Risk Group COVID–19 (W3)	dojc3 ~~ c137.6	phi_{OJC CS2, CV3}	−0.017 (0.01)	0.080	−0.090	−0.034 (0.023)	0.144	−0.168	−0.027 (0.014) *	0.049	−0.142	0 (0.018)	0.985	0.002	−0.021 (0.012)	0.074	−0.112	−0.007 (0.019)	0.712	−0.035	−0.013 (0.011)	0.234	−0.070	−0.04 (0.023)	0.090	−0.186
Covariance between OJC Change Score W3–4 and High Risk Group COVID–19 (W3)	dojc4 ~~ c137.6	phi_{OJC CS3, CV3}	0.013 (0.01)	0.196	0.075	0.02 (0.023)	0.392	0.107	0.013 (0.014)	0.351	0.077	0.004 (0.015)	0.763	0.036	0.017 (0.011)	0.122	0.114	0.006 (0.018)	0.728	0.036	0.008 (0.011)	0.448	0.050	0.028 (0.021)	0.190	0.161
Variance High Risk Group COVID–19 (W3)	c137.6 ~~ c137.6	phi_{CV3}	0.206 (0.014) ***	0.000	1.000	0.241 (0.036) ***	0.000	1.000	0.2 (0.018) ***	0.000	1.000	0.191 (0.025) ***	0.000	1.000	0.214 (0.017) ***	0.000	1.000	0.177 (0.022) ***	0.000	1.000	0.206 (0.015) ***	0.000	1.000	0.207 (0.029) ***	0.000	1.000
Variance SRH W1	SRH_W1 ~~ SRH_W1	phi_{SRH1}	0.448 (0.036) ***	0.000	1.000	0.423 (0.062) ***	0.000	1.000	0.445 (0.044) ***	0.000	1.000	0.458 (0.064) ***	0.000	1.000	0.459 (0.041) ***	0.000	1.000	0.435 (0.056) ***	0.000	1.000	0.421 (0.037) ***	0.000	1.000	0.551 (0.072) ***	0.000	1.000
Variance SRH W2	SRH_W2 ~~ SRH_W2	psi_{SRH2}	0.02 (0.021)	0.345	0.045	0.006 (0.029)	0.840	0.014	0.021 (0.026)	0.432	0.045	0.019 (0.034)	0.567	0.043	0.014 (0.021)	0.496	0.033	0.041 (0.032)	0.199	0.096	0.02 (0.022)	0.374	0.047	0.018 (0.031)	0.567	0.034
Variance SRH W3	SRH_W3 ~~ SRH_W3	psi_{SRH3}	0.03 (0.017)	0.079	0.067	0.048 (0.032)	0.128	0.113	0.027 (0.022)	0.227	0.061	0.007 (0.028)	0.809	0.016	0.03 (0.019)	0.103	0.069	0.041 (0.028)	0.155	0.095	0.038 (0.019) *	0.049	0.089	0.002 (0.025)	0.925	0.005
Variance SRH W4	SRH_W4 ~~ SRH_W4	psi_{SRH4}	0.062 (0.024) *	0.011	0.139	0.024 (0.043)	0.581	0.062	0.079 (0.033) *	0.016	0.170	0.051 (0.043)	0.237	0.126	0.034 (0.026)	0.194	0.077	0.098 (0.044) *	0.027	0.215	0.078 (0.028) **	0.006	0.181	0.002 (0.034)	0.950	0.005
Covariance JC W1 with JC Change Score W1–2	JC_W1 ~~ djc2	phi_{JC1, JC CS1}	−0.009 (0.003) **	0.007	−0.217	−0.004 (0.006)	0.468	−0.139	−0.008 (0.004)	0.065	−0.194	−0.015 (0.007) *	0.043	−0.330	−0.005 (0.004)	0.205	−0.116	−0.016 (0.006) **	0.010	−0.402	−0.039 (0.016) *	0.015	−0.212	−0.035 (0.019)	0.069	−0.260
Covariance JC W1 with JC Change Score W2–3	JC_W1 ~~ djc3	phi_{JC1, JC CS2}	−0.003 (0.003)	0.320	−0.083	−0.016 (0.006) *	0.012	−0.550	−0.003 (0.004)	0.471	−0.086	0.002 (0.007)	0.729	0.065	−0.002 (0.004)	0.494	−0.068	−0.009 (0.006)	0.128	−0.242	−0.025 (0.015)	0.094	−0.160	0.019 (0.025)	0.447	0.121
Covariance JC W1 with JC Change Score W3–4	JC_W1 ~~ djc4	phi_{JC1, JC CS3}	0.001 (0.004)	0.827	0.018	0.012 (0.007)	0.056	0.419	0.002 (0.006)	0.772	0.033	−0.007 (0.008)	0.395	−0.135	0.002 (0.005)	0.693	0.040	0.004 (0.006)	0.554	0.092	0.007 (0.018)	0.705	0.038	−0.02 (0.031)	0.528	−0.104
Covariance JC W1 with OJC W1	JC_W1 ~~ OJC_W1	phi_{JC1, OJC1}	0.041 (0.007) ***	0.000	0.349	0.036 (0.014) *	0.011	0.344	0.031 (0.009) ***	0.000	0.281	0.075 (0.017) ***	0.000	0.558	0.036 (0.008) ***	0.000	0.310	0.054 (0.013) ***	0.000	0.453	0.077 (0.016) ***	0.000	0.319	0.095 (0.026) ***	0.000	0.418
Covariance JC W1 with SRH W1	JC_W1 ~~ SRH_W1	phi_{JC1, SRH1}	0.034 (0.009) ***	0.000	0.194	0.006 (0.017)	0.736	0.040	0.023 (0.012) *	0.049	0.135	0.058 (0.02) **	0.004	0.303	0.032 (0.011) **	0.003	0.182	0.03 (0.016)	0.072	0.170	0.068 (0.021) **	0.001	0.186	0.064 (0.034)	0.057	0.189
Covariance OJC W1 with OJC Change Score W1–2	OJC_W1 ~~ dojc2	phi_{OJC1, OJC CS1}	−0.064 (0.011) ***	0.000	−0.348	−0.097 (0.028) ***	0.000	−0.424	−0.057 (0.014) ***	0.000	−0.341	−0.068 (0.023) **	0.003	−0.360	−0.058 (0.013) ***	0.000	−0.319	−0.071 (0.022) **	0.001	−0.339	−0.05 (0.012) ***	0.000	−0.283	−0.109 (0.025) ***	0.000	−0.512
Covariance OJC W1 with OJC Change Score W2–3	OJC_W1 ~~ dojc3	phi_{OJC1, OJC CS2}	0.001 (0.01)	0.945	0.004	−0.05 (0.028)	0.069	−0.254	0.015 (0.014)	0.280	0.082	0.004 (0.021)	0.832	0.024	0.001 (0.012)	0.910	0.008	0.001 (0.023)	0.951	0.006	−0.008 (0.011)	0.448	−0.048	0.04 (0.027)	0.149	0.171
Covariance OJC W1 with OJC Change Score W3–4	OJC_W1 ~~ dojc4	phi_{OJC1, OJC CS3}	−0.001 (0.011)	0.898	−0.008	0.025 (0.029)	0.388	0.142	0.004 (0.015)	0.806	0.022	−0.019 (0.018)	0.301	−0.141	0.003 (0.012)	0.799	0.022	0 (0.021)	0.985	−0.002	−0.001 (0.011)	0.894	−0.010	−0.006 (0.029)	0.831	−0.032
Covariance OJC W1 with SRH W1	OJC_W1 ~~ SRH_W1	phi_{OJC1, SRH1}	0.089 (0.015) ***	0.000	0.298	0.089 (0.032) **	0.005	0.289	0.082 (0.019) ***	0.000	0.288	0.111 (0.033) ***	0.001	0.344	0.107 (0.018) ***	0.000	0.351	0.07 (0.027) **	0.009	0.238	0.076 (0.016) ***	0.000	0.274	0.14 (0.036) ***	0.000	0.381
Intercept High Risk Group COVID–19 (W3)	c137.6 ~1	xi_{CV3}	0.287 (0.02) ***	0.000	0.633	0.326 (0.047) ***	0.000	0.664	0.275 (0.028) ***	0.000	0.616	0.277 (0.039) ***	0.000	0.633	0.303 (0.025) ***	0.000	0.656	0.237 (0.036) ***	0.000	0.564	0.287 (0.023) ***	0.000	0.633	0.287 (0.042) ***	0.000	0.630

Note: HO new = new in home office, FOW = Full office workers, HO exp = experienced in home office, P/F = Living with partner/family, NC = No contractual changes, CC = Contractual changes. Sample sizes: N (Full Sample= = 783, N (HO new / FOW / HO exp / HO new) = 162 / 407 / 187, N (P/F / alone) = 540 / 213, N (NC / CC) = 588 / 187
