# Supplementary material for: Crafting for Health: A Longitudinal Study of Job and Off-Job Crafting Changes during the COVID-19 Pandemic
Source: Occup Health Sci. 2025 Feb 26;9(3):675–710. doi: 10.1007/s41542-025-00222-5 (PMC12484252; doi:10.1007/s41542-025-00222-5)
Supplement: Supplementary file 4 — Supplementary file4 (HTML 888 KB) [file 41542_2025_222_MOESM4_ESM.html]

Crafting during corona times: Final latent change score models


# Crafting during corona times: Final latent change score models

## Packages & Data

```
knitr::opts_chunk$set(warning = FALSE, message = FALSE) 

# Data handling
library(tidyverse)
```

```
## ── Attaching core tidyverse packages ──────────────────────── tidyverse 2.0.0 ──
## ✔ dplyr     1.1.2     ✔ readr     2.1.4
## ✔ forcats   1.0.0     ✔ stringr   1.5.0
## ✔ ggplot2   3.4.2     ✔ tibble    3.2.1
## ✔ lubridate 1.9.2     ✔ tidyr     1.3.0
## ✔ purrr     1.0.1     
## ── Conflicts ────────────────────────────────────────── tidyverse_conflicts() ──
## ✖ dplyr::filter() masks stats::filter()
## ✖ dplyr::lag()    masks stats::lag()
## ℹ Use the conflicted package (<http://conflicted.r-lib.org/>) to force all conflicts to become errors
```

```
library(readr)
library(lubridate)
library(sjlabelled)
```

```
## 
## Attaching package: 'sjlabelled'
## 
## The following object is masked from 'package:forcats':
## 
##     as_factor
## 
## The following object is masked from 'package:dplyr':
## 
##     as_label
## 
## The following object is masked from 'package:ggplot2':
## 
##     as_label
```

```
# Plots and Tables
library(ggplot2)
library(ggstar)
library(jtools)
library(apaTables)
```

```
## Registered S3 methods overwritten by 'broom':
##   method            from  
##   tidy.glht         jtools
##   tidy.summary.glht jtools
```

```
library(semTable)
library(semPlot)
# Data Analysis
library(psych)
```

```
## 
## Attaching package: 'psych'
## 
## The following objects are masked from 'package:ggplot2':
## 
##     %+%, alpha
```

```
library(lavaan)
```

```
## This is lavaan 0.6-16
## lavaan is FREE software! Please report any bugs.
## 
## Attaching package: 'lavaan'
## 
## The following object is masked from 'package:psych':
## 
##     cor2cov
```

```
library(lcsm)
```

```
## This is lcsm 0.3.2
## Please report any issues or ideas at:
## https://github.com/milanwiedemann/lcsm/issues
```

```
# For a funny beep sound when model estimation finished
library(beepr)

data <- readRDS("Paper 2 Data_wAddVariables.RDS") # Data available from first author upon reasonable request
```

## Latent Change Score Models

### Definition of measurement models

```
# Measurement model of job and off-job crafting ----
measurement_model_parcelled_jc_ojc <- '
# # # # # # # # # # # # # # # # # # # # #
# Specify parameters for job crafting ----
# # # # # # # # # # # # # # # # # # # # #
# Specify latent true scores
JC_W1 =~ s21_rd.3 + jc1*s21_str.3 + jc2*s21_cd.3 + jc3*s21_soz.3
JC_W2 =~ s21_rd.4 + jc1*s21_str.4 + jc2*s21_cd.4 + jc3*s21_soz.4
JC_W3 =~ s21_rd.6 + jc1*s21_str.6 + jc2*s21_cd.6 + jc3*s21_soz.6
JC_W4 =~ s21_rd.7 + jc1*s21_str.7 + jc2*s21_cd.7 + jc3*s21_soz.7

# Specify manifest intercepts (partial MI)
s21_cd.3 ~ jc4*1
s21_cd.4 ~ jc4*1
s21_cd.6 ~ jc4*1
s21_cd.7 ~ jc4*1
s21_soz.3 ~jc5*1
s21_soz.4 ~jc5*1
s21_soz.6 ~jc5*1
s21_soz.7 ~jc5*1

# Residual covariances between same items
s21_rd.3 ~~ s21_rd.4 + s21_rd.6 + s21_rd.7
s21_rd.4 ~~ s21_rd.6 + s21_rd.7
s21_rd.6 ~~ s21_rd.7
s21_str.3 ~~ s21_str.4 + s21_str.6 + s21_str.7
s21_str.4 ~~ s21_str.6 + s21_str.7
s21_str.6 ~~ s21_str.7
s21_cd.3 ~~ s21_cd.4 + s21_cd.6 + s21_cd.7
s21_cd.4 ~~ s21_cd.6 + s21_cd.7
s21_cd.6 ~~ s21_cd.7
s21_soz.3 ~~ s21_soz.4 + s21_soz.6 + s21_soz.7
s21_soz.4 ~~ s21_soz.6 + s21_soz.7
s21_soz.6 ~~ s21_soz.7

# Specify mean of latent true scores
JC_W1 ~ 1 # label gamma_jc1 removed
JC_W2 ~ 0 * 1
JC_W3 ~ 0 * 1
JC_W4 ~ 0 * 1

# Specify variance of latent true scores
JC_W1 ~~ JC_W1 # label sigma2_jc1 removed
JC_W2 ~~ 0 * JC_W2
JC_W3 ~~ 0 * JC_W3
JC_W4 ~~ 0 * JC_W4

# Specify autoregressions of latent variables
JC_W2 ~ 1 * JC_W1
JC_W3 ~ 1 * JC_W2
JC_W4 ~ 1 * JC_W3

# Specify latent change scores
djc2 =~ 1 * JC_W2
djc3 =~ 1 * JC_W3
djc4 =~ 1 * JC_W4

# Specify latent change scores means (from Geiser)
djc2 ~ 1 # label gamma_jc2 removed
djc3 ~ 1 # label gamma_jc3 removed
djc4 ~ 1 # label gamma_jc4 removed

# Specify latent change scores variances (from Geiser)
djc2 ~~ djc2 # label res_jc2 removed
djc3 ~~ djc3 # label res_jc3 removed
djc4 ~~ djc4 # label res_jc4 removed

# Intercepts of reference variables set to zero (from Geiser)
s21_rd.3 ~ 0 * 1
s21_rd.4 ~ 0 * 1
s21_rd.6 ~ 0 * 1
s21_rd.7 ~ 0 * 1

# Change factors are allowed to correlate (from Geiser)
djc2 ~~ djc3 + djc4
djc3 ~~ djc4

# # # # # # # # # # # # # # # # # # # # #
# Specify parameters for off-job crafting ----
# # # # # # # # # # # # # # # # # # # # #
# Specify latent true scores
OJC_W1 =~ s200_de.3 + ojc1*s200_re.3 + ojc2*s200_au.3 + ojc3*s200_ma.3 + ojc4*s200_me.3 + ojc5*s200_af.3
OJC_W2 =~ s200_de.4 + ojc1*s200_re.4 + ojc2*s200_au.4 + ojc3*s200_ma.4 + ojc4*s200_me.4 + ojc5*s200_af.4
OJC_W3 =~ s200_de.6 + ojc1*s200_re.6 + ojc2*s200_au.6 + ojc3*s200_ma.6 + ojc4*s200_me.6 + ojc5*s200_af.6
OJC_W4 =~ s200_de.7 + ojc1*s200_re.7 + ojc2*s200_au.7 + ojc3*s200_ma.7 + ojc4*s200_me.7 + ojc5*s200_af.7

# Specify manifest intercepts (partial MI)
s200_re.3 ~ ojc7*1
s200_re.4 ~ ojc7*1
s200_re.6 ~ ojc7*1
s200_re.7 ~ ojc7*1
s200_au.3 ~ ojc8*1
s200_au.4 ~ ojc8*1
s200_au.6 ~ ojc8*1
s200_au.7 ~ ojc8*1
s200_ma.3 ~ ojc9*1
s200_ma.4 ~ ojc9*1
s200_ma.6 ~ ojc9*1
s200_ma.7 ~ ojc9*1
s200_me.3 ~ ojc10*1
s200_me.4 ~ ojc10*1
s200_me.6 ~ ojc10*1
s200_me.7 ~ ojc10*1


# Allowing a residual covariance between detachment & relaxation
s200_de.3 ~~ s200_re.3
s200_de.4 ~~ s200_re.4
s200_de.6 ~~ s200_re.6
s200_de.7 ~~ s200_re.7

# Residual covariances for same items
s200_de.3 ~~ s200_de.4 + s200_de.6 + s200_de.7
s200_de.4 ~~ s200_de.6 + s200_de.7
s200_de.6 ~~ s200_de.7
s200_re.3 ~~ s200_re.4 + s200_re.6 + s200_re.7
s200_re.4 ~~ s200_re.6 + s200_re.7
s200_re.6 ~~ s200_re.7
s200_au.3 ~~ s200_au.4 + s200_au.6 + s200_au.7
s200_au.4 ~~ s200_au.6 + s200_au.7
s200_au.6 ~~ s200_au.7
s200_ma.3 ~~ s200_ma.4 + s200_ma.6 + s200_ma.7
s200_ma.4 ~~ s200_ma.6 + s200_ma.7
s200_ma.6 ~~ s200_ma.7
s200_me.3 ~~ s200_me.4 + s200_me.6 + s200_me.7
s200_me.4 ~~ s200_me.6 + s200_me.7
s200_me.6 ~~ s200_me.7
s200_af.3 ~~ s200_af.4 + s200_af.6 + s200_af.7
s200_af.4 ~~ s200_af.6 + s200_af.7
s200_af.6 ~~ s200_af.7

# Specify mean of latent true scores 
OJC_W1 ~ 1 # label gamma_ojc1 removed
OJC_W2 ~ 0 * 1
OJC_W3 ~ 0 * 1
OJC_W4 ~ 0 * 1

# Specify variance of latent true scores 
OJC_W1 ~~ OJC_W1 # label sigma2_ojc1 removed
OJC_W2 ~~ 0 * OJC_W2
OJC_W3 ~~ 0 * OJC_W3
OJC_W4 ~~ 0 * OJC_W4

# Specify autoregressions of latent variables 
OJC_W2 ~ 1 * OJC_W1
OJC_W3 ~ 1 * OJC_W2
OJC_W4 ~ 1 * OJC_W3

# Specify latent change scores 
dojc2 =~ 1 * OJC_W2
dojc3 =~ 1 * OJC_W3
dojc4 =~ 1 * OJC_W4

# Specify latent change scores means (from Geiser)
dojc2 ~ 1 # label gamma_ojc2 removed
dojc3 ~ 1 # label gamma_ojc3 removed
dojc4 ~ 1 # label gamma_ojc4 removed

# Specify latent change scores variances (from Geiser)
dojc2 ~~ dojc2 # label res_ojc2 removed
dojc3 ~~ dojc3 # label res_ojc3 removed
dojc4 ~~ dojc4 # label res_ojc4 removed

# Intercepts of reference variables set to zero (from Geiser)
s200_de.3 ~ 0 * 1
s200_de.4 ~ 0 * 1
s200_de.6 ~ 0 * 1
s200_de.7 ~ 0 * 1

# Change factors are allowed to correlate (from Geiser)
dojc2 ~~ dojc3 + dojc4
dojc3 ~~ dojc4
'


# Measurement of self-rated health to be used in the full sample analysis ----
# In the following measurement model, the error variances are set to be equal across time. 
# This measurement model should be used for the full sample analysis.
# For the group comparison 1-4, even though this model is more restrictive, it works better than one where the 
# residual variances are only fixed intragroup.
measurement_model_srh <- '
# # # # # # # # # # # # # # # # # # # # # # # # # # #
# Add outcome self-rated health and regressions ----
# # # # # # # # # # # # # # # # # # # # # # # # # # #

# Specify latent true scores 
SRH_W1 =~ 1 * s38.3r
SRH_W2 =~ 1 * s38.4r
SRH_W3 =~ 1 * s38.6 # no reverse code needed
SRH_W4 =~ 1 * s38.7 # no reverse code needed

# Specify intercept of obseved scores
s38.3r ~ 0 * 1
s38.4r ~ 0 * 1
s38.6 ~ 0 * 1
s38.7 ~ 0 * 1

# Settng residual variances equal across time
s38.3r ~~ res_srh * s38.3r
s38.4r ~~ res_srh * s38.4r
s38.6 ~~ res_srh * s38.6
s38.7 ~~ res_srh * s38.7

# Specifying latent means
SRH_W1 ~ 1 # label gamma_srh1 removed
SRH_W2 ~ 1 # label gamma_srh2 removed
SRH_W3 ~ 1 # label gamma_srh3 removed
SRH_W4 ~ 1 # label gamma_srh4 removed

# Autoregressions
SRH_W2 ~ SRH_W1
SRH_W3 ~ SRH_W2
SRH_W4 ~ SRH_W3

'

# Auto-regressive paths of SRH set to be equal across groups
measurement_model_srh_equalAR <- '
# # # # # # # # # # # # # # # # # # # # # # # # # # #
# Add outcome self-rated health and regressions ----
# # # # # # # # # # # # # # # # # # # # # # # # # # #

# Specify latent true scores 
SRH_W1 =~ 1 * s38.3r
SRH_W2 =~ 1 * s38.4r
SRH_W3 =~ 1 * s38.6 # no reverse code needed
SRH_W4 =~ 1 * s38.7 # no reverse code needed

# Specify intercept of obseved scores
s38.3r ~ 0 * 1
s38.4r ~ 0 * 1
s38.6 ~ 0 * 1
s38.7 ~ 0 * 1

# Settng residual variances equal across time
s38.3r ~~ res_srh * s38.3r
s38.4r ~~ res_srh * s38.4r
s38.6 ~~ res_srh * s38.6
s38.7 ~~ res_srh * s38.7

# Specifying latent means
SRH_W1 ~ 1 # label gamma_srh1 removed
SRH_W2 ~ 1 # label gamma_srh2 removed
SRH_W3 ~ 1 # label gamma_srh3 removed
SRH_W4 ~ 1 # label gamma_srh4 removed

# Autoregressions - equal across groups
SRH_W2 ~ ar1_srh*SRH_W1
SRH_W3 ~ ar2_srh*SRH_W2
SRH_W4 ~ ar3_srh*SRH_W3

'

# Additional part to test for equal change score intercepts across groups ----

# Base part and change score intercepts separated
measurement_model_parcelled_jc_ojc_GC1_BASE <- '
# # # # # # # # # # # # # # # # # # # # #
# Specify parameters for job crafting ----
# # # # # # # # # # # # # # # # # # # # #
# Specify latent true scores
JC_W1 =~ s21_rd.3 + jc1*s21_str.3 + jc2*s21_cd.3 + jc3*s21_soz.3
JC_W2 =~ s21_rd.4 + jc1*s21_str.4 + jc2*s21_cd.4 + jc3*s21_soz.4
JC_W3 =~ s21_rd.6 + jc1*s21_str.6 + jc2*s21_cd.6 + jc3*s21_soz.6
JC_W4 =~ s21_rd.7 + jc1*s21_str.7 + jc2*s21_cd.7 + jc3*s21_soz.7

# Specify manifest intercepts
s21_str.3 ~ jc6*1
s21_str.4 ~ jc6*1
s21_str.6 ~ jc6*1
s21_str.7 ~ jc6*1
s21_cd.3 ~ jc4*1
s21_cd.4 ~ jc4*1
s21_cd.6 ~ jc4*1
s21_cd.7 ~ jc4*1
s21_soz.3 ~jc5*1
s21_soz.4 ~jc5*1
s21_soz.6 ~jc5*1
s21_soz.7 ~jc5*1

# Residual covariances between same items
s21_rd.3 ~~ s21_rd.4 + s21_rd.6 + s21_rd.7
s21_rd.4 ~~ s21_rd.6 + s21_rd.7
s21_rd.6 ~~ s21_rd.7
s21_str.3 ~~ s21_str.4 + s21_str.6 + s21_str.7
s21_str.4 ~~ s21_str.6 + s21_str.7
s21_str.6 ~~ s21_str.7
s21_cd.3 ~~ s21_cd.4 + s21_cd.6 + s21_cd.7
s21_cd.4 ~~ s21_cd.6 + s21_cd.7
s21_cd.6 ~~ s21_cd.7
s21_soz.3 ~~ s21_soz.4 + s21_soz.6 + s21_soz.7
s21_soz.4 ~~ s21_soz.6 + s21_soz.7
s21_soz.6 ~~ s21_soz.7

# Specify mean of latent true scores
JC_W1 ~ 1 # label gamma_jc1 removed
JC_W2 ~ 0 * 1
JC_W3 ~ 0 * 1
JC_W4 ~ 0 * 1

# Specify variance of latent true scores
JC_W1 ~~ JC_W1 # label sigma2_jc1 removed
JC_W2 ~~ 0 * JC_W2
JC_W3 ~~ 0 * JC_W3
JC_W4 ~~ 0 * JC_W4

# Specify autoregressions of latent variables
JC_W2 ~ 1 * JC_W1
JC_W3 ~ 1 * JC_W2
JC_W4 ~ 1 * JC_W3

# Specify latent change scores
djc2 =~ 1 * JC_W2
djc3 =~ 1 * JC_W3
djc4 =~ 1 * JC_W4

# Specify latent change scores variances (from Geiser)
djc2 ~~ djc2 # label res_jc2 removed
djc3 ~~ djc3 # label res_jc3 removed
djc4 ~~ djc4 # label res_jc4 removed

# Intercepts of reference variables set to zero (from Geiser)
s21_rd.3 ~ 0 * 1
s21_rd.4 ~ 0 * 1
s21_rd.6 ~ 0 * 1
s21_rd.7 ~ 0 * 1

# Change factors are allowed to correlate (from Geiser)
djc2 ~~ djc3 + djc4
djc3 ~~ djc4

# # # # # # # # # # # # # # # # # # # # #
# Specify parameters for off-job crafting ----
# # # # # # # # # # # # # # # # # # # # #
# Specify latent true scores
OJC_W1 =~ s200_de.3 + ojc1*s200_re.3 + ojc2*s200_au.3 + ojc3*s200_ma.3 + ojc4*s200_me.3 + ojc5*s200_af.3
OJC_W2 =~ s200_de.4 + ojc1*s200_re.4 + ojc2*s200_au.4 + ojc3*s200_ma.4 + ojc4*s200_me.4 + ojc5*s200_af.4
OJC_W3 =~ s200_de.6 + ojc1*s200_re.6 + ojc2*s200_au.6 + ojc3*s200_ma.6 + ojc4*s200_me.6 + ojc5*s200_af.6
OJC_W4 =~ s200_de.7 + ojc1*s200_re.7 + ojc2*s200_au.7 + ojc3*s200_ma.7 + ojc4*s200_me.7 + ojc5*s200_af.7

# Specify manifest intercepts (Partial MI: Af freed)
s200_re.3 ~ ojc6*1
s200_re.4 ~ ojc6*1
s200_re.6 ~ ojc6*1
s200_re.7 ~ ojc6*1
s200_au.3 ~ ojc7*1
s200_au.4 ~ ojc7*1
s200_au.6 ~ ojc7*1
s200_au.7 ~ ojc7*1
s200_ma.3 ~ ojc8*1
s200_ma.4 ~ ojc8*1
s200_ma.6 ~ ojc8*1
s200_ma.7 ~ ojc8*1
s200_me.3 ~ ojc9*1
s200_me.4 ~ ojc9*1
s200_me.6 ~ ojc9*1
s200_me.7 ~ ojc9*1
s200_af.3 ~ c(ojc10_1, ojc10_2, ojc10_3)*1
s200_af.4 ~ c(ojc10_1, ojc10_2, ojc10_3)*1
s200_af.6 ~ c(ojc10_1, ojc10_2, ojc10_3)*1
s200_af.7 ~ c(ojc10_1, ojc10_2, ojc10_3)*1

# Allowing a residual covariance between detachment & relaxation
s200_de.3 ~~ s200_re.3
s200_de.4 ~~ s200_re.4
s200_de.6 ~~ s200_re.6
s200_de.7 ~~ s200_re.7

# Residual covariances for same items
s200_de.3 ~~ s200_de.4 + s200_de.6 + s200_de.7
s200_de.4 ~~ s200_de.6 + s200_de.7
s200_de.6 ~~ s200_de.7
s200_re.3 ~~ s200_re.4 + s200_re.6 + s200_re.7
s200_re.4 ~~ s200_re.6 + s200_re.7
s200_re.6 ~~ s200_re.7
s200_au.3 ~~ s200_au.4 + s200_au.6 + s200_au.7
s200_au.4 ~~ s200_au.6 + s200_au.7
s200_au.6 ~~ s200_au.7
s200_ma.3 ~~ s200_ma.4 + s200_ma.6 + s200_ma.7
s200_ma.4 ~~ s200_ma.6 + s200_ma.7
s200_ma.6 ~~ s200_ma.7
s200_me.3 ~~ s200_me.4 + s200_me.6 + s200_me.7
s200_me.4 ~~ s200_me.6 + s200_me.7
s200_me.6 ~~ s200_me.7
s200_af.3 ~~ s200_af.4 + s200_af.6 + s200_af.7
s200_af.4 ~~ s200_af.6 + s200_af.7
s200_af.6 ~~ s200_af.7

# Specify mean of latent true scores 
OJC_W1 ~ 1 # label gamma_ojc1 removed
OJC_W2 ~ 0 * 1
OJC_W3 ~ 0 * 1
OJC_W4 ~ 0 * 1

# Specify variance of latent true scores 
OJC_W1 ~~ OJC_W1 # label sigma2_ojc1 removed
OJC_W2 ~~ 0 * OJC_W2
OJC_W3 ~~ 0 * OJC_W3
OJC_W4 ~~ 0 * OJC_W4

# Specify autoregressions of latent variables 
OJC_W2 ~ 1 * OJC_W1
OJC_W3 ~ 1 * OJC_W2
OJC_W4 ~ 1 * OJC_W3

# Specify latent change scores 
dojc2 =~ 1 * OJC_W2
dojc3 =~ 1 * OJC_W3
dojc4 =~ 1 * OJC_W4

# Specify latent change scores variances (from Geiser)
dojc2 ~~ dojc2 # label res_ojc2 removed
dojc3 ~~ dojc3 # label res_ojc3 removed
dojc4 ~~ dojc4 # label res_ojc4 removed

# Intercepts of reference variables set to zero (from Geiser)
s200_de.3 ~ 0 * 1
s200_de.4 ~ 0 * 1
s200_de.6 ~ 0 * 1
s200_de.7 ~ 0 * 1

# Change factors are allowed to correlate (from Geiser)
dojc2 ~~ dojc3 + dojc4
dojc3 ~~ dojc4
'

measurement_model_parcelled_jc_ojc_GC2_BASE <- '
# # # # # # # # # # # # # # # # # # # # #
# Specify parameters for job crafting ----
# # # # # # # # # # # # # # # # # # # # #
# Specify latent true scores
JC_W1 =~ s21_rd.3 + jc1*s21_str.3 + jc2*s21_cd.3 + jc3*s21_soz.3
JC_W2 =~ s21_rd.4 + jc1*s21_str.4 + jc2*s21_cd.4 + jc3*s21_soz.4
JC_W3 =~ s21_rd.6 + jc1*s21_str.6 + jc2*s21_cd.6 + jc3*s21_soz.6
JC_W4 =~ s21_rd.7 + jc1*s21_str.7 + jc2*s21_cd.7 + jc3*s21_soz.7

# Specify manifest intercepts
s21_str.3 ~ c(NA, jc6)*1
s21_str.4 ~ c(NA, jc6)*1
s21_str.6 ~ c(NA, jc6)*1
s21_str.7 ~ c(NA, jc6)*1
s21_cd.3 ~ jc4*1
s21_cd.4 ~ jc4*1
s21_cd.6 ~ jc4*1
s21_cd.7 ~ jc4*1
s21_soz.3 ~jc5*1
s21_soz.4 ~jc5*1
s21_soz.6 ~jc5*1
s21_soz.7 ~jc5*1

# Residual covariances between same items
s21_rd.3 ~~ s21_rd.4 + s21_rd.6 + s21_rd.7
s21_rd.4 ~~ s21_rd.6 + s21_rd.7
s21_rd.6 ~~ s21_rd.7
s21_str.3 ~~ s21_str.4 + s21_str.6 + s21_str.7
s21_str.4 ~~ s21_str.6 + s21_str.7
s21_str.6 ~~ s21_str.7
s21_cd.3 ~~ s21_cd.4 + s21_cd.6 + s21_cd.7
s21_cd.4 ~~ s21_cd.6 + s21_cd.7
s21_cd.6 ~~ s21_cd.7
s21_soz.3 ~~ s21_soz.4 + s21_soz.6 + s21_soz.7
s21_soz.4 ~~ s21_soz.6 + s21_soz.7
s21_soz.6 ~~ s21_soz.7

# Specify mean of latent true scores
JC_W1 ~ 1 # label gamma_jc1 removed
JC_W2 ~ 0 * 1
JC_W3 ~ 0 * 1
JC_W4 ~ 0 * 1

# Specify variance of latent true scores
JC_W1 ~~ JC_W1 # label sigma2_jc1 removed
JC_W2 ~~ 0 * JC_W2
JC_W3 ~~ 0 * JC_W3
JC_W4 ~~ 0 * JC_W4

# Specify autoregressions of latent variables
JC_W2 ~ 1 * JC_W1
JC_W3 ~ 1 * JC_W2
JC_W4 ~ 1 * JC_W3

# Specify latent change scores
djc2 =~ 1 * JC_W2
djc3 =~ 1 * JC_W3
djc4 =~ 1 * JC_W4

# Specify latent change scores variances (from Geiser)
djc2 ~~ djc2 # label res_jc2 removed
djc3 ~~ djc3 # label res_jc3 removed
djc4 ~~ djc4 # label res_jc4 removed

# Intercepts of reference variables set to zero (from Geiser)
s21_rd.3 ~ 0 * 1
s21_rd.4 ~ 0 * 1
s21_rd.6 ~ 0 * 1
s21_rd.7 ~ 0 * 1

# Change factors are allowed to correlate (from Geiser)
djc2 ~~ djc3 + djc4
djc3 ~~ djc4

# # # # # # # # # # # # # # # # # # # # #
# Specify parameters for off-job crafting ----
# # # # # # # # # # # # # # # # # # # # #
# Specify latent true scores
OJC_W1 =~ s200_de.3 + ojc1*s200_re.3 + ojc2*s200_au.3 + ojc3*s200_ma.3 + ojc4*s200_me.3 + ojc5*s200_af.3
OJC_W2 =~ s200_de.4 + ojc1*s200_re.4 + ojc2*s200_au.4 + ojc3*s200_ma.4 + ojc4*s200_me.4 + ojc5*s200_af.4
OJC_W3 =~ s200_de.6 + ojc1*s200_re.6 + ojc2*s200_au.6 + ojc3*s200_ma.6 + ojc4*s200_me.6 + ojc5*s200_af.6
OJC_W4 =~ s200_de.7 + ojc1*s200_re.7 + ojc2*s200_au.7 + ojc3*s200_ma.7 + ojc4*s200_me.7 + ojc5*s200_af.7

# Specify manifest intercepts (Partial MI: Af freed)
s200_re.3 ~ ojc6*1
s200_re.4 ~ ojc6*1
s200_re.6 ~ ojc6*1
s200_re.7 ~ ojc6*1
s200_au.3 ~ c(ojc7_1, ojc7_2)*1
s200_au.4 ~ c(ojc7_1, ojc7_2)*1
s200_au.6 ~ c(ojc7_1, ojc7_2)*1
s200_au.7 ~ c(ojc7_1, ojc7_2)*1
s200_ma.3 ~ ojc8*1
s200_ma.4 ~ ojc8*1
s200_ma.6 ~ ojc8*1
s200_ma.7 ~ ojc8*1
s200_me.3 ~ ojc9*1
s200_me.4 ~ ojc9*1
s200_me.6 ~ ojc9*1
s200_me.7 ~ ojc9*1
s200_af.3 ~ c(ojc10_1, ojc10_2)*1
s200_af.4 ~ c(ojc10_1, ojc10_2)*1
s200_af.6 ~ c(ojc10_1, ojc10_2)*1
s200_af.7 ~ c(ojc10_1, ojc10_2)*1

# Allowing a residual covariance between detachment & relaxation
s200_de.3 ~~ s200_re.3
s200_de.4 ~~ s200_re.4
s200_de.6 ~~ s200_re.6
s200_de.7 ~~ s200_re.7

# Residual covariances for same items
s200_de.3 ~~ s200_de.4 + s200_de.6 + s200_de.7
s200_de.4 ~~ s200_de.6 + s200_de.7
s200_de.6 ~~ s200_de.7
s200_re.3 ~~ s200_re.4 + s200_re.6 + s200_re.7
s200_re.4 ~~ s200_re.6 + s200_re.7
s200_re.6 ~~ s200_re.7
s200_au.3 ~~ s200_au.4 + s200_au.6 + s200_au.7
s200_au.4 ~~ s200_au.6 + s200_au.7
s200_au.6 ~~ s200_au.7
s200_ma.3 ~~ s200_ma.4 + s200_ma.6 + s200_ma.7
s200_ma.4 ~~ s200_ma.6 + s200_ma.7
s200_ma.6 ~~ s200_ma.7
s200_me.3 ~~ s200_me.4 + s200_me.6 + s200_me.7
s200_me.4 ~~ s200_me.6 + s200_me.7
s200_me.6 ~~ s200_me.7
s200_af.3 ~~ s200_af.4 + s200_af.6 + s200_af.7
s200_af.4 ~~ s200_af.6 + s200_af.7
s200_af.6 ~~ s200_af.7

# Specify mean of latent true scores 
OJC_W1 ~ 1 # label gamma_ojc1 removed
OJC_W2 ~ 0 * 1
OJC_W3 ~ 0 * 1
OJC_W4 ~ 0 * 1

# Specify variance of latent true scores 
OJC_W1 ~~ OJC_W1 # label sigma2_ojc1 removed
OJC_W2 ~~ 0 * OJC_W2
OJC_W3 ~~ 0 * OJC_W3
OJC_W4 ~~ 0 * OJC_W4

# Specify autoregressions of latent variables 
OJC_W2 ~ 1 * OJC_W1
OJC_W3 ~ 1 * OJC_W2
OJC_W4 ~ 1 * OJC_W3

# Specify latent change scores 
dojc2 =~ 1 * OJC_W2
dojc3 =~ 1 * OJC_W3
dojc4 =~ 1 * OJC_W4

# Specify latent change scores variances (from Geiser)
dojc2 ~~ dojc2 # label res_ojc2 removed
dojc3 ~~ dojc3 # label res_ojc3 removed
dojc4 ~~ dojc4 # label res_ojc4 removed

# Intercepts of reference variables set to zero (from Geiser)
s200_de.3 ~ 0 * 1
s200_de.4 ~ 0 * 1
s200_de.6 ~ 0 * 1
s200_de.7 ~ 0 * 1

# Change factors are allowed to correlate (from Geiser)
dojc2 ~~ dojc3 + dojc4
dojc3 ~~ dojc4
'

# GC4 did not properly converge with all restrictions (heywood case)
# After changing the reference indicator and freeing the intercept of SOZ, model converges
measurement_model_parcelled_jc_ojc_GC4_BASE <- '
# # # # # # # # # # # # # # # # # # # # #
# Specify parameters for job crafting ----
# # # # # # # # # # # # # # # # # # # # #
# Specify latent true scores (partial metric MI)
JC_W1 =~ s21_cd.3 + jc1*s21_str.3 + s21_rd.3 + c(jc3_1, jc3_2)*s21_soz.3
JC_W2 =~ s21_cd.4 + jc1*s21_str.4 + s21_rd.4 + c(jc3_1, jc3_2)*s21_soz.4
JC_W3 =~ s21_cd.6 + jc1*s21_str.6 + s21_rd.6 + c(jc3_1, jc3_2)*s21_soz.6
JC_W4 =~ s21_cd.7 + jc1*s21_str.7 + s21_rd.7 + c(jc3_1, jc3_2)*s21_soz.7

# specify manifest intercepts (partial scalar MI: for first group, RD & STR need to be freed, for second only)
s21_str.3 ~ c(NA, jc6)*1
s21_str.4 ~ c(NA, jc6)*1
s21_str.6 ~ c(NA, jc6)*1
s21_str.7 ~ c(NA, jc6)*1
# s21_soz.3 ~jc5*1
# s21_soz.4 ~jc5*1
# s21_soz.6 ~jc5*1
# s21_soz.7 ~jc5*1

# Residual covariances between same items
s21_rd.3 ~~ s21_rd.4 + s21_rd.6 + s21_rd.7
s21_rd.4 ~~ s21_rd.6 + s21_rd.7
s21_rd.6 ~~ s21_rd.7
s21_str.3 ~~ s21_str.4 + s21_str.6 + s21_str.7
s21_str.4 ~~ s21_str.6 + s21_str.7
s21_str.6 ~~ s21_str.7
s21_cd.3 ~~ s21_cd.4 + s21_cd.6 + s21_cd.7
s21_cd.4 ~~ s21_cd.6 + s21_cd.7
s21_cd.6 ~~ s21_cd.7
s21_soz.3 ~~ s21_soz.4 + s21_soz.6 + s21_soz.7
s21_soz.4 ~~ s21_soz.6 + s21_soz.7
s21_soz.6 ~~ s21_soz.7

# Specify mean of latent true scores
JC_W1 ~ 1 # label gamma_jc1 removed
JC_W2 ~ 0 * 1
JC_W3 ~ 0 * 1
JC_W4 ~ 0 * 1

# Specify variance of latent true scores
JC_W1 ~~ JC_W1 # label sigma2_jc1 removed
JC_W2 ~~ 0 * JC_W2
JC_W3 ~~ 0 * JC_W3
JC_W4 ~~ 0 * JC_W4

# Specify autoregressions of latent variables
JC_W2 ~ 1 * JC_W1
JC_W3 ~ 1 * JC_W2
JC_W4 ~ 1 * JC_W3

# Specify latent change scores
djc2 =~ 1 * JC_W2
djc3 =~ 1 * JC_W3
djc4 =~ 1 * JC_W4

# Specify latent change scores variances (from Geiser)
djc2 ~~ djc2 # label res_jc2 removed
djc3 ~~ djc3 # label res_jc3 removed
djc4 ~~ djc4 # label res_jc4 removed

# Intercepts of reference variables set to zero (from Geiser)
s21_cd.3 ~ 0 * 1
s21_cd.4 ~ 0 * 1
s21_cd.6 ~ 0 * 1
s21_cd.7 ~ 0 * 1

# Change factors are allowed to correlate (from Geiser)
djc2 ~~ djc3 + djc4
djc3 ~~ djc4

# # # # # # # # # # # # # # # # # # # # #
# Specify parameters for off-job crafting ----
# # # # # # # # # # # # # # # # # # # # #
# Specify latent true scores
OJC_W1 =~ s200_de.3 + ojc1*s200_re.3 + ojc2*s200_au.3 + ojc3*s200_ma.3 + ojc4*s200_me.3 + ojc5*s200_af.3
OJC_W2 =~ s200_de.4 + ojc1*s200_re.4 + ojc2*s200_au.4 + ojc3*s200_ma.4 + ojc4*s200_me.4 + ojc5*s200_af.4
OJC_W3 =~ s200_de.6 + ojc1*s200_re.6 + ojc2*s200_au.6 + ojc3*s200_ma.6 + ojc4*s200_me.6 + ojc5*s200_af.6
OJC_W4 =~ s200_de.7 + ojc1*s200_re.7 + ojc2*s200_au.7 + ojc3*s200_ma.7 + ojc4*s200_me.7 + ojc5*s200_af.7

# Specify manifest intercepts
s200_re.3 ~ ojc6*1
s200_re.4 ~ ojc6*1
s200_re.6 ~ ojc6*1
s200_re.7 ~ ojc6*1
s200_au.3 ~ ojc7*1
s200_au.4 ~ ojc7*1
s200_au.6 ~ ojc7*1
s200_au.7 ~ ojc7*1
s200_ma.3 ~ ojc8*1
s200_ma.4 ~ ojc8*1
s200_ma.6 ~ ojc8*1
s200_ma.7 ~ ojc8*1
s200_me.3 ~ ojc9*1
s200_me.4 ~ ojc9*1
s200_me.6 ~ ojc9*1
s200_me.7 ~ ojc9*1
s200_af.3 ~ ojc10*1
s200_af.4 ~ ojc10*1
s200_af.6 ~ ojc10*1
s200_af.7 ~ ojc10*1

# Allowing a residual covariance between detachment & relaxation
s200_de.3 ~~ s200_re.3
s200_de.4 ~~ s200_re.4
s200_de.6 ~~ s200_re.6
s200_de.7 ~~ s200_re.7

# Residual covariances for same items
s200_de.3 ~~ s200_de.4 + s200_de.6 + s200_de.7
s200_de.4 ~~ s200_de.6 + s200_de.7
s200_de.6 ~~ s200_de.7
s200_re.3 ~~ s200_re.4 + s200_re.6 + s200_re.7
s200_re.4 ~~ s200_re.6 + s200_re.7
s200_re.6 ~~ s200_re.7
s200_au.3 ~~ s200_au.4 + s200_au.6 + s200_au.7
s200_au.4 ~~ s200_au.6 + s200_au.7
s200_au.6 ~~ s200_au.7
s200_ma.3 ~~ s200_ma.4 + s200_ma.6 + s200_ma.7
s200_ma.4 ~~ s200_ma.6 + s200_ma.7
s200_ma.6 ~~ s200_ma.7
s200_me.3 ~~ s200_me.4 + s200_me.6 + s200_me.7
s200_me.4 ~~ s200_me.6 + s200_me.7
s200_me.6 ~~ s200_me.7
s200_af.3 ~~ s200_af.4 + s200_af.6 + s200_af.7
s200_af.4 ~~ s200_af.6 + s200_af.7
s200_af.6 ~~ s200_af.7

# Specify mean of latent true scores 
OJC_W1 ~ 1 # label gamma_ojc1 removed
OJC_W2 ~ 0 * 1
OJC_W3 ~ 0 * 1
OJC_W4 ~ 0 * 1

# Specify variance of latent true scores 
OJC_W1 ~~ OJC_W1 # label sigma2_ojc1 removed
OJC_W2 ~~ 0 * OJC_W2
OJC_W3 ~~ 0 * OJC_W3
OJC_W4 ~~ 0 * OJC_W4

# Specify autoregressions of latent variables 
OJC_W2 ~ 1 * OJC_W1
OJC_W3 ~ 1 * OJC_W2
OJC_W4 ~ 1 * OJC_W3

# Specify latent change scores 
dojc2 =~ 1 * OJC_W2
dojc3 =~ 1 * OJC_W3
dojc4 =~ 1 * OJC_W4

# Specify latent change scores variances (from Geiser)
dojc2 ~~ dojc2 # label res_ojc2 removed
dojc3 ~~ dojc3 # label res_ojc3 removed
dojc4 ~~ dojc4 # label res_ojc4 removed

# Intercepts of reference variables set to zero (from Geiser)
s200_de.3 ~ 0 * 1
s200_de.4 ~ 0 * 1
s200_de.6 ~ 0 * 1
s200_de.7 ~ 0 * 1

# Change factors are allowed to correlate (from Geiser)
dojc2 ~~ dojc3 + dojc4
dojc3 ~~ dojc4
'
# Change score JC
fixed_cs_jc_123 <- '
# Specify latent change scores means (from Geiser)
djc2 ~ gamma_jc2*1 # label gamma_jc2 added
djc3 ~ gamma_jc3*1 # label gamma_jc3 added
djc4 ~ gamma_jc4*1 # label gamma_jc4 added
'

# Change scores OJC
fixed_cs_ojc_3 <- '
# Specify latent change scores means (from Geiser)
dojc2 ~ 1 
dojc3 ~ 1 
dojc4 ~ gamma_ojc4*1 # label gamma_ojc4 added
'

fixed_cs_ojc_123 <- '
# Specify latent change scores means (from Geiser)
dojc2 ~ gamma_ojc2*1 # label gamma_ojc2 added
dojc3 ~ gamma_ojc3*1 # label gamma_ojc3 added
dojc4 ~ gamma_ojc4*1 # label gamma_ojc4 added
'
```

### Latent structural models

```
# Latent structural model part with either self-rated health or work-life satisfaction as outcomes

# Considering the non-equidistant time intervals, we suggest that only the covariances between djc and dojc are unaffected. 
# Regressions between djc/dojc and subsequent SRH represent the strength of the relationship given the time interval, and this is affected by 
# Latent structural model part with either self-rated health or work-life satisfaction as outcomes

model_latent_srh_timeequalCOV <- '
# Covariances of change scores
djc2 ~~ cov1*dojc2
djc3 ~~ cov1*dojc3
djc4 ~~ cov1*dojc4
                           
# Specify regressions of latent variables and of change scores 
SRH_W2 ~ djc2 + dojc2 + c137.6 # Controlling for high risk group 
SRH_W3 ~ djc3 + dojc3 + c137.6 # Controlling for high risk group 
SRH_W4 ~ djc4 + dojc4 + c137.6 # Controlling for high risk group

# control variable is allowed to correlate with remaining factors
c137.6 ~~ JC_W1 + OJC_W1 + SRH_W1 + djc2 + djc3 + djc4 + dojc2 + dojc3 + dojc4
                    
'
# Version with equal regression weights from c137.6 to SRH, equal covariances of JC and OJC changes, equal paths from djc to SRH, and equal paths from dojc to SRH
# And adding equality constraint across time too
model_latent_srh_equalc137COVDJCDOJC_timeequalCOV <- '
# Covariances of change scores
djc2 ~~ cov1*dojc2
djc3 ~~ cov1*dojc3
djc4 ~~ cov1*dojc4
                           
# Specify regressions of latent variables and of change scores 
SRH_W2 ~ jcsrh1*djc2 + ojcsrh1*dojc2 + covid1*c137.6 # Controlling for high risk group 
SRH_W3 ~ jcsrh2*djc3 + ojcsrh2*dojc3 + covid2*c137.6 # Controlling for high risk group 
SRH_W4 ~ jcsrh3*djc4 + ojcsrh3*dojc4 + covid3*c137.6 # Controlling for high risk group

# control variable is allowed to correlate with remaining factors
c137.6 ~~ JC_W1 + OJC_W1 + SRH_W1 + djc2 + djc3 + djc4 + dojc2 + dojc3 + dojc4
                    
'

# Version with equal regression weights from c137.6 to SRH (W2 and W3 only), equal covariances of JC and OJC changes, equal paths from djc to SRH, and equal paths from dojc to SRH (W3 only)
# And adding equality constraint across time too
model_latent_srh_equalc137W23COVJCOJCW23_timeequalCOV <- '
# Covariances of change scores
djc2 ~~ cov1*dojc2
djc3 ~~ cov1*dojc3
djc4 ~~ cov1*dojc4
                           
# Specify regressions of latent variables and of change scores 
SRH_W2 ~ jcsrh1*djc2 + ojcsrh1*dojc2 + covid1*c137.6 # Controlling for high risk group 
SRH_W3 ~ jcsrh2*djc3 + ojcsrh2*dojc3 + covid2*c137.6 # Controlling for high risk group 
SRH_W4 ~ jcsrh3*djc4 + dojc4 + c137.6 # Controlling for high risk group

# control variable is allowed to correlate with remaining factors
c137.6 ~~ JC_W1 + OJC_W1 + SRH_W1 + djc2 + djc3 + djc4 + dojc2 + dojc3 + dojc4
                    
'

# Version with equal regression weights from c137.6 to SRH and equal covariances of JC and OJC changes (W3 and 4 only), equal paths from djc to SRH, and equal paths from dojc to SRH (Wave 1-2 & 3-4 only)
model_latent_srh_equalc137COVW34JCOJCW24_timeequalCOV <- '
# Covariances of change scores
djc2 ~~ c(cov1, cov2)*dojc2
djc3 ~~ cov1*dojc3
djc4 ~~ cov1*dojc4
                           
# Specify regressions of latent variables and of change scores 
SRH_W2 ~ jcsrh1*djc2 + ojcsrh1*dojc2 + covid1*c137.6 # Controlling for high risk group 
SRH_W3 ~ jcsrh2*djc3 + dojc3 + covid2*c137.6 # Controlling for high risk group 
SRH_W4 ~ jcsrh3*djc4 + ojcsrh3*dojc4 + covid3*c137.6 # Controlling for high risk group

# control variable is allowed to correlate with remaining factors
c137.6 ~~ JC_W1 + OJC_W1 + SRH_W1 + djc2 + djc3 + djc4 + dojc2 + dojc3 + dojc4
                    
'
```

### Full sample

```
# Within the following syntax, the structural part for the JC & OJC latent change scores is adapted from Geiser (2020) for latent change score models using multiple indicators
# The structural part to fit the outcome variables is adapted from Geiser (2020) for simplex models using single indicators

## LCSM Job and Off-Job Crafting with self-rated health as outcome ----
### Model estimation ----
model_lcsm_parcel_SRH_equalCOV <- paste(measurement_model_parcelled_jc_ojc, measurement_model_srh, model_latent_srh_timeequalCOV, sep = "")

lcsm_parcel_SRH_equalCOV <- sem(model_lcsm_parcel_SRH_equalCOV, data, 
                   estimator = "ML", 
                   missing = "ML",
                   fixed.x = FALSE); beep(6) # Has to be set to zero, otherwise the missings in the exogenous var c137.6 cause deletion of cases

summary(lcsm_parcel_SRH_equalCOV, fit.measures = TRUE, standardized = TRUE, fm.args = list(robust = FALSE))
```

```
## lavaan 0.6.16 ended normally after 354 iterations
## 
##   Estimator                                         ML
##   Optimization method                           NLMINB
##   Number of model parameters                       255
##   Number of equality constraints                    47
## 
##   Number of observations                           783
##   Number of missing patterns                        38
## 
## Model Test User Model:
##                                                       
##   Test statistic                              2004.173
##   Degrees of freedom                               872
##   P-value (Chi-square)                           0.000
## 
## Model Test Baseline Model:
## 
##   Test statistic                             16761.101
##   Degrees of freedom                               990
##   P-value                                        0.000
## 
## User Model versus Baseline Model:
## 
##   Comparative Fit Index (CFI)                    0.928
##   Tucker-Lewis Index (TLI)                       0.918
## 
## Loglikelihood and Information Criteria:
## 
##   Loglikelihood user model (H0)             -21800.166
##   Loglikelihood unrestricted model (H1)     -20798.079
##                                                       
##   Akaike (AIC)                               44016.331
##   Bayesian (BIC)                             44986.263
##   Sample-size adjusted Bayesian (SABIC)      44325.758
## 
## Root Mean Square Error of Approximation:
## 
##   RMSEA                                          0.041
##   90 Percent confidence interval - lower         0.038
##   90 Percent confidence interval - upper         0.043
##   P-value H_0: RMSEA <= 0.050                    1.000
##   P-value H_0: RMSEA >= 0.080                    0.000
## 
## Standardized Root Mean Square Residual:
## 
##   SRMR                                           0.076
## 
## Parameter Estimates:
## 
##   Standard errors                             Standard
##   Information                                 Observed
##   Observed information based on                Hessian
## 
## Latent Variables:
##                    Estimate  Std.Err  z-value  P(>|z|)   Std.lv  Std.all
##   JC_W1 =~                                                              
##     s21_r.3           1.000                               0.262    0.372
##     s21_s.3  (jc1)    1.360    0.118   11.566    0.000    0.356    0.571
##     s21_c.3  (jc2)    2.055    0.172   11.937    0.000    0.537    0.625
##     s21_s.3  (jc3)    1.776    0.146   12.192    0.000    0.464    0.733
##   JC_W2 =~                                                              
##     s21_r.4           1.000                               0.273    0.406
##     s21_s.4  (jc1)    1.360    0.118   11.566    0.000    0.372    0.587
##     s21_c.4  (jc2)    2.055    0.172   11.937    0.000    0.562    0.663
##     s21_s.4  (jc3)    1.776    0.146   12.192    0.000    0.486    0.743
##   JC_W3 =~                                                              
##     s21_r.6           1.000                               0.275    0.397
##     s21_s.6  (jc1)    1.360    0.118   11.566    0.000    0.373    0.567
##     s21_c.6  (jc2)    2.055    0.172   11.937    0.000    0.564    0.634
##     s21_s.6  (jc3)    1.776    0.146   12.192    0.000    0.488    0.745
##   JC_W4 =~                                                              
##     s21_r.7           1.000                               0.275    0.376
##     s21_s.7  (jc1)    1.360    0.118   11.566    0.000    0.374    0.556
##     s21_c.7  (jc2)    2.055    0.172   11.937    0.000    0.565    0.677
##     s21_s.7  (jc3)    1.776    0.146   12.192    0.000    0.489    0.743
##   djc2 =~                                                               
##     JC_W2             1.000                               0.565    0.565
##   djc3 =~                                                               
##     JC_W3             1.000                               0.515    0.515
##   djc4 =~                                                               
##     JC_W4             1.000                               0.614    0.614
##   OJC_W1 =~                                                             
##     s200_.3           1.000                               0.444    0.503
##     s200_.3 (ojc1)    0.980    0.041   23.721    0.000    0.435    0.537
##     s200_.3 (ojc2)    1.357    0.059   22.934    0.000    0.603    0.777
##     s200_.3 (ojc3)    1.412    0.065   21.699    0.000    0.627    0.729
##     s200_.3 (ojc4)    1.307    0.062   21.116    0.000    0.580    0.768
##     s200_.3 (ojc5)    1.191    0.062   19.296    0.000    0.529    0.641
##   OJC_W2 =~                                                             
##     s200_.4           1.000                               0.492    0.540
##     s200_.4 (ojc1)    0.980    0.041   23.721    0.000    0.482    0.593
##     s200_.4 (ojc2)    1.357    0.059   22.934    0.000    0.668    0.802
##     s200_.4 (ojc3)    1.412    0.065   21.699    0.000    0.695    0.805
##     s200_.4 (ojc4)    1.307    0.062   21.116    0.000    0.643    0.786
##     s200_.4 (ojc5)    1.191    0.062   19.296    0.000    0.586    0.627
##   OJC_W3 =~                                                             
##     s200_.6           1.000                               0.464    0.550
##     s200_.6 (ojc1)    0.980    0.041   23.721    0.000    0.455    0.588
##     s200_.6 (ojc2)    1.357    0.059   22.934    0.000    0.630    0.840
##     s200_.6 (ojc3)    1.412    0.065   21.699    0.000    0.656    0.826
##     s200_.6 (ojc4)    1.307    0.062   21.116    0.000    0.607    0.788
##     s200_.6 (ojc5)    1.191    0.062   19.296    0.000    0.553    0.636
##   OJC_W4 =~                                                             
##     s200_.7           1.000                               0.474    0.540
##     s200_.7 (ojc1)    0.980    0.041   23.721    0.000    0.464    0.601
##     s200_.7 (ojc2)    1.357    0.059   22.934    0.000    0.643    0.838
##     s200_.7 (ojc3)    1.412    0.065   21.699    0.000    0.669    0.826
##     s200_.7 (ojc4)    1.307    0.062   21.116    0.000    0.620    0.812
##     s200_.7 (ojc5)    1.191    0.062   19.296    0.000    0.565    0.698
##   dojc2 =~                                                              
##     OJC_W2            1.000                               0.847    0.847
##   dojc3 =~                                                              
##     OJC_W3            1.000                               0.892    0.892
##   dojc4 =~                                                              
##     OJC_W4            1.000                               0.780    0.780
##   SRH_W1 =~                                                             
##     s38.3r            1.000                               0.670    0.864
##   SRH_W2 =~                                                             
##     s38.4r            1.000                               0.665    0.862
##   SRH_W3 =~                                                             
##     s38.6             1.000                               0.665    0.862
##   SRH_W4 =~                                                             
##     s38.7             1.000                               0.665    0.862
## 
## Regressions:
##                    Estimate  Std.Err  z-value  P(>|z|)   Std.lv  Std.all
##   JC_W2 ~                                                               
##     JC_W1             1.000                               0.957    0.957
##   JC_W3 ~                                                               
##     JC_W2             1.000                               0.996    0.996
##   JC_W4 ~                                                               
##     JC_W3             1.000                               0.998    0.998
##   OJC_W2 ~                                                              
##     OJC_W1            1.000                               0.902    0.902
##   OJC_W3 ~                                                              
##     OJC_W2            1.000                               1.059    1.059
##   OJC_W4 ~                                                              
##     OJC_W3            1.000                               0.980    0.980
##   SRH_W2 ~                                                              
##     SRH_W1            0.955    0.050   18.945    0.000    0.962    0.962
##   SRH_W3 ~                                                              
##     SRH_W2            0.941    0.047   19.881    0.000    0.940    0.940
##   SRH_W4 ~                                                              
##     SRH_W3            0.958    0.055   17.281    0.000    0.958    0.958
##   SRH_W2 ~                                                              
##     djc2              0.497    0.203    2.444    0.015    0.116    0.116
##     dojc2             0.087    0.060    1.446    0.148    0.055    0.055
##     c137.6           -0.049    0.068   -0.721    0.471   -0.074   -0.033
##   SRH_W3 ~                                                              
##     djc3              0.468    0.251    1.865    0.062    0.100    0.100
##     dojc3             0.114    0.062    1.854    0.064    0.071    0.071
##     c137.6            0.000    0.064    0.007    0.995    0.001    0.000
##   SRH_W4 ~                                                              
##     djc4             -0.026    0.237   -0.110    0.913   -0.007   -0.007
##     dojc4             0.236    0.088    2.689    0.007    0.131    0.131
##     c137.6            0.123    0.075    1.653    0.098    0.185    0.084
## 
## Covariances:
##                    Estimate  Std.Err  z-value  P(>|z|)   Std.lv  Std.all
##  .s21_rd.3 ~~                                                           
##    .s21_r.4           0.209    0.019   11.133    0.000    0.209    0.519
##    .s21_r.6           0.239    0.022   10.836    0.000    0.239    0.576
##    .s21_r.7           0.258    0.024   10.676    0.000    0.258    0.582
##  .s21_rd.4 ~~                                                           
##    .s21_r.6           0.222    0.019   11.651    0.000    0.222    0.567
##    .s21_r.7           0.210    0.021    9.804    0.000    0.210    0.503
##  .s21_rd.6 ~~                                                           
##    .s21_r.7           0.259    0.024   10.732    0.000    0.259    0.602
##  .s21_str.3 ~~                                                          
##    .s21_s.4           0.163    0.014   11.547    0.000    0.163    0.620
##    .s21_s.6           0.191    0.016   11.655    0.000    0.191    0.690
##    .s21_s.7           0.201    0.017   11.485    0.000    0.201    0.702
##  .s21_str.4 ~~                                                          
##    .s21_s.6           0.188    0.015   12.253    0.000    0.188    0.676
##    .s21_s.7           0.190    0.016   11.612    0.000    0.190    0.663
##  .s21_str.6 ~~                                                          
##    .s21_s.7           0.217    0.019   11.666    0.000    0.217    0.715
##  .s21_cd.3 ~~                                                           
##    .s21_c.4           0.258    0.025   10.436    0.000    0.258    0.606
##    .s21_c.6           0.299    0.029   10.328    0.000    0.299    0.648
##    .s21_c.7           0.244    0.028    8.744    0.000    0.244    0.592
##  .s21_cd.4 ~~                                                           
##    .s21_c.6           0.289    0.027   10.790    0.000    0.289    0.664
##    .s21_c.7           0.236    0.026    9.206    0.000    0.236    0.606
##  .s21_cd.6 ~~                                                           
##    .s21_c.7           0.286    0.030    9.656    0.000    0.286    0.676
##  .s21_soz.3 ~~                                                          
##    .s21_s.4           0.106    0.014    7.568    0.000    0.106    0.563
##    .s21_s.6           0.107    0.015    7.020    0.000    0.107    0.567
##    .s21_s.7           0.096    0.016    6.219    0.000    0.096    0.508
##  .s21_soz.4 ~~                                                          
##    .s21_s.6           0.100    0.015    6.881    0.000    0.100    0.524
##    .s21_s.7           0.084    0.015    5.483    0.000    0.084    0.434
##  .s21_soz.6 ~~                                                          
##    .s21_s.7           0.101    0.017    6.111    0.000    0.101    0.526
##   djc2 ~~                                                               
##     djc3             -0.007    0.003   -2.540    0.011   -0.303   -0.303
##     djc4             -0.002    0.003   -0.827    0.408   -0.086   -0.086
##   djc3 ~~                                                               
##     djc4             -0.013    0.003   -3.829    0.000   -0.530   -0.530
##  .s200_de.3 ~~                                                          
##    .s200_.3           0.137    0.021    6.503    0.000    0.137    0.263
##  .s200_de.4 ~~                                                          
##    .s200_.4           0.146    0.018    8.001    0.000    0.146    0.292
##  .s200_de.6 ~~                                                          
##    .s200_.6           0.150    0.020    7.493    0.000    0.150    0.339
##  .s200_de.7 ~~                                                          
##    .s200_.7           0.187    0.023    8.106    0.000    0.187    0.409
##  .s200_de.3 ~~                                                          
##    .s200_.4           0.268    0.026   10.428    0.000    0.268    0.457
##    .s200_.6           0.215    0.025    8.581    0.000    0.215    0.399
##    .s200_.7           0.230    0.027    8.464    0.000    0.230    0.407
##  .s200_de.4 ~~                                                          
##    .s200_.6           0.194    0.023    8.373    0.000    0.194    0.359
##    .s200_.7           0.202    0.025    8.042    0.000    0.202    0.356
##  .s200_de.6 ~~                                                          
##    .s200_.7           0.228    0.025    9.158    0.000    0.228    0.436
##  .s200_re.3 ~~                                                          
##    .s200_.4           0.122    0.019    6.405    0.000    0.122    0.273
##    .s200_.6           0.146    0.021    7.073    0.000    0.146    0.342
##    .s200_.7           0.138    0.020    6.928    0.000    0.138    0.326
##  .s200_re.4 ~~                                                          
##    .s200_.6           0.134    0.018    7.544    0.000    0.134    0.329
##    .s200_.7           0.120    0.018    6.728    0.000    0.120    0.297
##  .s200_re.6 ~~                                                          
##    .s200_.7           0.120    0.019    6.345    0.000    0.120    0.310
##  .s200_au.3 ~~                                                          
##    .s200_.4           0.093    0.014    6.619    0.000    0.093    0.386
##    .s200_.6           0.059    0.013    4.379    0.000    0.059    0.296
##    .s200_.7           0.049    0.015    3.323    0.001    0.049    0.240
##  .s200_au.4 ~~                                                          
##    .s200_.6           0.053    0.013    4.228    0.000    0.053    0.263
##    .s200_.7           0.053    0.014    3.881    0.000    0.053    0.257
##  .s200_au.6 ~~                                                          
##    .s200_.7           0.062    0.013    4.652    0.000    0.062    0.361
##  .s200_ma.3 ~~                                                          
##    .s200_.4           0.093    0.017    5.451    0.000    0.093    0.309
##    .s200_.6           0.091    0.017    5.320    0.000    0.091    0.345
##    .s200_.7           0.068    0.018    3.694    0.000    0.068    0.252
##  .s200_ma.4 ~~                                                          
##    .s200_.6           0.071    0.014    5.202    0.000    0.071    0.312
##    .s200_.7           0.073    0.015    4.827    0.000    0.073    0.313
##  .s200_ma.6 ~~                                                          
##    .s200_.7           0.079    0.015    5.332    0.000    0.079    0.387
##  .s200_me.3 ~~                                                          
##    .s200_.4           0.070    0.014    5.129    0.000    0.070    0.287
##    .s200_.6           0.065    0.015    4.249    0.000    0.065    0.281
##    .s200_.7           0.076    0.015    5.156    0.000    0.076    0.351
##  .s200_me.4 ~~                                                          
##    .s200_.6           0.066    0.014    4.702    0.000    0.066    0.275
##    .s200_.7           0.065    0.015    4.425    0.000    0.065    0.288
##  .s200_me.6 ~~                                                          
##    .s200_.7           0.073    0.015    4.722    0.000    0.073    0.346
##  .s200_af.3 ~~                                                          
##    .s200_.4           0.180    0.022    8.131    0.000    0.180    0.390
##    .s200_.6           0.186    0.024    7.717    0.000    0.186    0.438
##    .s200_.7           0.168    0.021    7.908    0.000    0.168    0.459
##  .s200_af.4 ~~                                                          
##    .s200_.6           0.207    0.024    8.587    0.000    0.207    0.424
##    .s200_.7           0.183    0.023    7.795    0.000    0.183    0.434
##  .s200_af.6 ~~                                                          
##    .s200_.7           0.201    0.023    8.610    0.000    0.201    0.517
##   dojc2 ~~                                                              
##     dojc3            -0.100    0.013   -7.438    0.000   -0.578   -0.578
##     dojc4             0.007    0.011    0.651    0.515    0.046    0.046
##   dojc3 ~~                                                              
##     dojc4            -0.069    0.012   -5.770    0.000   -0.454   -0.454
##   djc2 ~~                                                               
##     dojc2   (cov1)    0.008    0.003    2.723    0.006    0.125    0.125
##   djc3 ~~                                                               
##     dojc3   (cov1)    0.008    0.003    2.723    0.006    0.138    0.138
##   djc4 ~~                                                               
##     dojc4   (cov1)    0.008    0.003    2.723    0.006    0.129    0.129
##   JC_W1 ~~                                                              
##     c137.6           -0.011    0.007   -1.626    0.104   -0.041   -0.090
##   OJC_W1 ~~                                                             
##     c137.6            0.023    0.011    2.217    0.027    0.053    0.116
##   SRH_W1 ~~                                                             
##     c137.6           -0.104    0.017   -6.109    0.000   -0.156   -0.343
##   djc2 ~~                                                               
##     c137.6            0.001    0.005    0.291    0.771    0.009    0.020
##   djc3 ~~                                                               
##     c137.6           -0.009    0.004   -2.123    0.034   -0.065   -0.144
##   djc4 ~~                                                               
##     c137.6            0.001    0.005    0.155    0.877    0.005    0.011
##   dojc2 ~~                                                              
##     c137.6           -0.005    0.010   -0.507    0.612   -0.012   -0.027
##   dojc3 ~~                                                              
##     c137.6           -0.017    0.010   -1.753    0.080   -0.041   -0.090
##   dojc4 ~~                                                              
##     c137.6            0.013    0.010    1.293    0.196    0.034    0.075
##   JC_W1 ~~                                                              
##     djc2             -0.009    0.003   -2.675    0.007   -0.217   -0.217
##     djc3             -0.003    0.003   -0.994    0.320   -0.083   -0.083
##     djc4              0.001    0.004    0.219    0.827    0.018    0.018
##     OJC_W1            0.041    0.007    5.639    0.000    0.349    0.349
##     dojc2             0.008    0.006    1.321    0.186    0.070    0.070
##     dojc3            -0.013    0.006   -1.991    0.046   -0.116   -0.116
##     dojc4             0.001    0.007    0.171    0.864    0.012    0.012
##     SRH_W1            0.034    0.009    3.769    0.000    0.194    0.194
##   djc2 ~~                                                               
##     OJC_W1           -0.009    0.004   -2.165    0.030   -0.131   -0.131
##     dojc3             0.006    0.004    1.286    0.199    0.086    0.086
##     dojc4            -0.004    0.005   -0.957    0.338   -0.078   -0.078
##     SRH_W1           -0.007    0.007   -1.080    0.280   -0.072   -0.072
##   djc3 ~~                                                               
##     OJC_W1            0.000    0.005    0.009    0.993    0.001    0.001
##     dojc2             0.002    0.004    0.524    0.600    0.040    0.040
##     dojc4            -0.005    0.004   -1.183    0.237   -0.094   -0.094
##     SRH_W1            0.020    0.007    2.902    0.004    0.216    0.216
##   djc4 ~~                                                               
##     OJC_W1           -0.003    0.006   -0.546    0.585   -0.043   -0.043
##     dojc2            -0.001    0.006   -0.163    0.871   -0.013   -0.013
##     dojc3            -0.007    0.005   -1.395    0.163   -0.097   -0.097
##     SRH_W1           -0.015    0.008   -1.769    0.077   -0.129   -0.129
##   OJC_W1 ~~                                                             
##     dojc2            -0.064    0.011   -5.711    0.000   -0.348   -0.348
##     dojc3             0.001    0.010    0.069    0.945    0.004    0.004
##     dojc4            -0.001    0.011   -0.128    0.898   -0.008   -0.008
##     SRH_W1            0.089    0.015    5.920    0.000    0.298    0.298
##   dojc2 ~~                                                              
##     SRH_W1            0.011    0.015    0.746    0.456    0.039    0.039
##   dojc3 ~~                                                              
##     SRH_W1            0.002    0.015    0.102    0.919    0.005    0.005
##   dojc4 ~~                                                              
##     SRH_W1           -0.016    0.015   -1.061    0.289   -0.065   -0.065
## 
## Intercepts:
##                    Estimate  Std.Err  z-value  P(>|z|)   Std.lv  Std.all
##    .s21_c.3  (jc4)   -3.453    0.493   -6.999    0.000   -3.453   -4.015
##    .s21_c.4  (jc4)   -3.453    0.493   -6.999    0.000   -3.453   -4.076
##    .s21_c.6  (jc4)   -3.453    0.493   -6.999    0.000   -3.453   -3.882
##    .s21_c.7  (jc4)   -3.453    0.493   -6.999    0.000   -3.453   -4.134
##    .s21_s.3  (jc5)   -2.538    0.417   -6.080    0.000   -2.538   -4.005
##    .s21_s.4  (jc5)   -2.538    0.417   -6.080    0.000   -2.538   -3.882
##    .s21_s.6  (jc5)   -2.538    0.417   -6.080    0.000   -2.538   -3.879
##    .s21_s.7  (jc5)   -2.538    0.417   -6.080    0.000   -2.538   -3.859
##     JC_W1             2.891    0.023  127.488    0.000   11.055   11.055
##    .JC_W2             0.000                               0.000    0.000
##    .JC_W3             0.000                               0.000    0.000
##    .JC_W4             0.000                               0.000    0.000
##     djc2             -0.044    0.010   -4.379    0.000   -0.287   -0.287
##     djc3              0.022    0.010    2.236    0.025    0.155    0.155
##     djc4             -0.075    0.013   -5.762    0.000   -0.445   -0.445
##    .s21_r.3           0.000                               0.000    0.000
##    .s21_r.4           0.000                               0.000    0.000
##    .s21_r.6           0.000                               0.000    0.000
##    .s21_r.7           0.000                               0.000    0.000
##    .s200_.3 (ojc7)   -0.046    0.161   -0.282    0.778   -0.046   -0.056
##    .s200_.4 (ojc7)   -0.046    0.161   -0.282    0.778   -0.046   -0.056
##    .s200_.6 (ojc7)   -0.046    0.161   -0.282    0.778   -0.046   -0.059
##    .s200_.7 (ojc7)   -0.046    0.161   -0.282    0.778   -0.046   -0.059
##    .s200_.3 (ojc8)   -1.463    0.231   -6.331    0.000   -1.463   -1.887
##    .s200_.4 (ojc8)   -1.463    0.231   -6.331    0.000   -1.463   -1.758
##    .s200_.6 (ojc8)   -1.463    0.231   -6.331    0.000   -1.463   -1.949
##    .s200_.7 (ojc8)   -1.463    0.231   -6.331    0.000   -1.463   -1.907
##    .s200_.3 (ojc9)   -2.050    0.254   -8.075    0.000   -2.050   -2.383
##    .s200_.4 (ojc9)   -2.050    0.254   -8.075    0.000   -2.050   -2.374
##    .s200_.6 (ojc9)   -2.050    0.254   -8.075    0.000   -2.050   -2.583
##    .s200_.7 (ojc9)   -2.050    0.254   -8.075    0.000   -2.050   -2.531
##    .s200_.3 (oj10)   -1.510    0.242   -6.251    0.000   -1.510   -1.997
##    .s200_.4 (oj10)   -1.510    0.242   -6.251    0.000   -1.510   -1.847
##    .s200_.6 (oj10)   -1.510    0.242   -6.251    0.000   -1.510   -1.961
##    .s200_.7 (oj10)   -1.510    0.242   -6.251    0.000   -1.510   -1.979
##     OJC_W1            3.939    0.028  138.776    0.000    8.871    8.871
##    .OJC_W2            0.000                               0.000    0.000
##    .OJC_W3            0.000                               0.000    0.000
##    .OJC_W4            0.000                               0.000    0.000
##     dojc2            -0.047    0.019   -2.456    0.014   -0.113   -0.113
##     dojc3            -0.095    0.020   -4.729    0.000   -0.229   -0.229
##     dojc4             0.028    0.021    1.317    0.188    0.075    0.075
##    .s200_.3           0.000                               0.000    0.000
##    .s200_.4           0.000                               0.000    0.000
##    .s200_.6           0.000                               0.000    0.000
##    .s200_.7           0.000                               0.000    0.000
##    .s38.3r            0.000                               0.000    0.000
##    .s38.4r            0.000                               0.000    0.000
##    .s38.6             0.000                               0.000    0.000
##    .s38.7             0.000                               0.000    0.000
##     SRH_W1            3.599    0.030  120.589    0.000    5.375    5.375
##    .SRH_W2            0.269    0.192    1.400    0.162    0.404    0.404
##    .SRH_W3            0.246    0.182    1.352    0.177    0.370    0.370
##    .SRH_W4            0.072    0.214    0.335    0.737    0.108    0.108
##    .s21_s.3          -0.120    0.339   -0.354    0.724   -0.120   -0.192
##    .s21_s.4          -0.140    0.337   -0.415    0.678   -0.140   -0.221
##    .s21_s.6          -0.224    0.338   -0.662    0.508   -0.224   -0.340
##    .s21_s.7          -0.194    0.335   -0.580    0.562   -0.194   -0.289
##    .s200_.3          -0.862    0.243   -3.543    0.000   -0.862   -1.046
##    .s200_.4          -0.992    0.242   -4.094    0.000   -0.992   -1.061
##    .s200_.6          -0.963    0.240   -4.005    0.000   -0.963   -1.107
##    .s200_.7          -0.906    0.241   -3.761    0.000   -0.906   -1.119
##     c137.6            0.287    0.020   14.141    0.000    0.287    0.633
## 
## Variances:
##                    Estimate  Std.Err  z-value  P(>|z|)   Std.lv  Std.all
##     JC_W1             0.068    0.011    6.230    0.000    1.000    1.000
##    .JC_W2             0.000                               0.000    0.000
##    .JC_W3             0.000                               0.000    0.000
##    .JC_W4             0.000                               0.000    0.000
##     djc2              0.024    0.005    5.210    0.000    1.000    1.000
##     djc3              0.020    0.004    5.010    0.000    1.000    1.000
##     djc4              0.029    0.006    5.019    0.000    1.000    1.000
##     OJC_W1            0.197    0.020    9.712    0.000    1.000    1.000
##    .OJC_W2            0.000                               0.000    0.000
##    .OJC_W3            0.000                               0.000    0.000
##    .OJC_W4            0.000                               0.000    0.000
##     dojc2             0.174    0.019    9.263    0.000    1.000    1.000
##     dojc3             0.171    0.019    9.002    0.000    1.000    1.000
##     dojc4             0.137    0.017    8.057    0.000    1.000    1.000
##    .s38.3r  (rs_s)    0.153    0.012   12.745    0.000    0.153    0.254
##    .s38.4r  (rs_s)    0.153    0.012   12.745    0.000    0.153    0.257
##    .s38.6   (rs_s)    0.153    0.012   12.745    0.000    0.153    0.257
##    .s38.7   (rs_s)    0.153    0.012   12.745    0.000    0.153    0.257
##    .s21_r.3           0.426    0.026   16.282    0.000    0.426    0.862
##    .s21_s.3           0.261    0.018   14.460    0.000    0.261    0.674
##    .s21_c.3           0.451    0.033   13.777    0.000    0.451    0.609
##    .s21_s.3           0.186    0.018   10.559    0.000    0.186    0.463
##    .s21_r.4           0.379    0.020   18.520    0.000    0.379    0.835
##    .s21_s.4           0.263    0.016   16.338    0.000    0.263    0.656
##    .s21_c.4           0.402    0.027   14.679    0.000    0.402    0.560
##    .s21_s.4           0.192    0.016   11.749    0.000    0.192    0.449
##    .s21_r.6           0.403    0.026   15.548    0.000    0.403    0.843
##    .s21_s.6           0.294    0.021   14.163    0.000    0.294    0.678
##    .s21_c.6           0.473    0.036   12.966    0.000    0.473    0.598
##    .s21_s.6           0.190    0.019    9.971    0.000    0.190    0.445
##    .s21_r.7           0.460    0.032   14.172    0.000    0.460    0.859
##    .s21_s.7           0.312    0.023   13.344    0.000    0.312    0.691
##    .s21_c.7           0.378    0.035   10.873    0.000    0.378    0.542
##    .s21_s.7           0.194    0.021    9.117    0.000    0.194    0.448
##    .s200_.3           0.583    0.034   17.219    0.000    0.583    0.747
##    .s200_.3           0.468    0.028   16.621    0.000    0.468    0.712
##    .s200_.3           0.238    0.019   12.413    0.000    0.238    0.396
##    .s200_.3           0.347    0.026   13.592    0.000    0.347    0.469
##    .s200_.3           0.235    0.019   12.613    0.000    0.235    0.411
##    .s200_.3           0.400    0.027   14.977    0.000    0.400    0.589
##    .s200_.4           0.587    0.030   19.492    0.000    0.587    0.708
##    .s200_.4           0.427    0.023   18.857    0.000    0.427    0.648
##    .s200_.4           0.247    0.017   14.342    0.000    0.247    0.356
##    .s200_.4           0.263    0.018   14.425    0.000    0.263    0.352
##    .s200_.4           0.255    0.017   14.801    0.000    0.255    0.382
##    .s200_.4           0.530    0.030   17.735    0.000    0.530    0.607
##    .s200_.6           0.498    0.030   16.482    0.000    0.498    0.698
##    .s200_.6           0.391    0.025   15.637    0.000    0.391    0.654
##    .s200_.6           0.166    0.015   10.836    0.000    0.166    0.295
##    .s200_.6           0.200    0.017   11.619    0.000    0.200    0.317
##    .s200_.6           0.225    0.019   12.097    0.000    0.225    0.379
##    .s200_.6           0.450    0.031   14.387    0.000    0.450    0.595
##    .s200_.7           0.547    0.036   15.386    0.000    0.547    0.709
##    .s200_.7           0.381    0.027   14.372    0.000    0.381    0.639
##    .s200_.7           0.175    0.017   10.118    0.000    0.175    0.297
##    .s200_.7           0.208    0.019   10.700    0.000    0.208    0.317
##    .s200_.7           0.198    0.019   10.703    0.000    0.198    0.340
##    .s200_.7           0.336    0.027   12.574    0.000    0.336    0.513
##     c137.6            0.206    0.014   15.235    0.000    0.206    1.000
##     SRH_W1            0.448    0.036   12.606    0.000    1.000    1.000
##    .SRH_W2            0.020    0.021    0.945    0.345    0.045    0.045
##    .SRH_W3            0.030    0.017    1.757    0.079    0.067    0.067
##    .SRH_W4            0.062    0.024    2.541    0.011    0.139    0.139
```

### Full-office workers vs. Forced teleworking to more than 50%

```
## LCSM Job and Off-Job Crafting with self-rated health as outcome ----

### Model estimation ----
model_lcsm_parcel_SRH_GC1_equalARc137COVJCOJC_timeequalCOV_equalCSJC123OJC3 <- paste(measurement_model_parcelled_jc_ojc_GC1_BASE, fixed_cs_jc_123, fixed_cs_ojc_3, measurement_model_srh_equalAR, model_latent_srh_equalc137COVDJCDOJC_timeequalCOV, sep = "")

lcsm_parcel_SRH_GC1_equalARc137COVJCOJC_timeequalCOV_equalCSJC123OJC3 <- sem(model_lcsm_parcel_SRH_GC1_equalARc137COVJCOJC_timeequalCOV_equalCSJC123OJC3, data, 
                   estimator = "ML", 
                   missing = "ML",
                   group = "group1_workloc.4",
                   fixed.x = FALSE); beep(6) # Has to be set to zero, otherwise the missings in the exogenous var c137.6 cause deletion of cases

summary(lcsm_parcel_SRH_GC1_equalARc137COVJCOJC_timeequalCOV_equalCSJC123OJC3, fit.measures = TRUE, standardized = TRUE, fm.args = list(robust = FALSE))
```

```
## lavaan 0.6.16 ended normally after 558 iterations
## 
##   Estimator                                         ML
##   Optimization method                           NLMINB
##   Number of model parameters                       765
##   Number of equality constraints                   225
## 
##   Number of observations per group:                   
##     HO new                                         162
##     FOW                                            407
##     HO exp                                         187
##   Number of missing patterns per group:               
##     HO new                                          15
##     FOW                                             30
##     HO exp                                          14
## 
## Model Test User Model:
##                                                       
##   Test statistic                              4911.145
##   Degrees of freedom                              2700
##   P-value (Chi-square)                           0.000
##   Test statistic for each group:
##     HO new                                    1643.675
##     FOW                                       1664.734
##     HO exp                                    1602.736
## 
## Model Test Baseline Model:
## 
##   Test statistic                             19109.530
##   Degrees of freedom                              2970
##   P-value                                        0.000
## 
## User Model versus Baseline Model:
## 
##   Comparative Fit Index (CFI)                    0.863
##   Tucker-Lewis Index (TLI)                       0.849
## 
## Loglikelihood and Information Criteria:
## 
##   Loglikelihood user model (H0)             -20803.529
##   Loglikelihood unrestricted model (H1)     -18347.957
##                                                       
##   Akaike (AIC)                               42687.059
##   Bayesian (BIC)                             45186.201
##   Sample-size adjusted Bayesian (SABIC)      43471.479
## 
## Root Mean Square Error of Approximation:
## 
##   RMSEA                                          0.057
##   90 Percent confidence interval - lower         0.054
##   90 Percent confidence interval - upper         0.060
##   P-value H_0: RMSEA <= 0.050                    0.000
##   P-value H_0: RMSEA >= 0.080                    0.000
## 
## Standardized Root Mean Square Residual:
## 
##   SRMR                                           0.093
## 
## Parameter Estimates:
## 
##   Standard errors                             Standard
##   Information                                 Observed
##   Observed information based on                Hessian
## 
## 
## Group 1 [HO new]:
## 
## Latent Variables:
##                    Estimate  Std.Err  z-value  P(>|z|)   Std.lv  Std.all
##   JC_W1 =~                                                              
##     s21_r.3           1.000                               0.216    0.310
##     s21_s.3  (jc1)    1.369    0.118   11.648    0.000    0.295    0.496
##     s21_c.3  (jc2)    1.992    0.167   11.921    0.000    0.429    0.518
##     s21_s.3  (jc3)    1.765    0.145   12.186    0.000    0.380    0.627
##   JC_W2 =~                                                              
##     s21_r.4           1.000                               0.241    0.384
##     s21_s.4  (jc1)    1.369    0.118   11.648    0.000    0.330    0.559
##     s21_c.4  (jc2)    1.992    0.167   11.921    0.000    0.481    0.581
##     s21_s.4  (jc3)    1.765    0.145   12.186    0.000    0.426    0.695
##   JC_W3 =~                                                              
##     s21_r.6           1.000                               0.193    0.267
##     s21_s.6  (jc1)    1.369    0.118   11.648    0.000    0.264    0.447
##     s21_c.6  (jc2)    1.992    0.167   11.921    0.000    0.384    0.448
##     s21_s.6  (jc3)    1.765    0.145   12.186    0.000    0.340    0.592
##   JC_W4 =~                                                              
##     s21_r.7           1.000                               0.262    0.341
##     s21_s.7  (jc1)    1.369    0.118   11.648    0.000    0.358    0.574
##     s21_c.7  (jc2)    1.992    0.167   11.921    0.000    0.521    0.683
##     s21_s.7  (jc3)    1.765    0.145   12.186    0.000    0.462    0.690
##   djc2 =~                                                               
##     JC_W2             1.000                               0.592    0.592
##   djc3 =~                                                               
##     JC_W3             1.000                               0.686    0.686
##   djc4 =~                                                               
##     JC_W4             1.000                               0.512    0.512
##   OJC_W1 =~                                                             
##     s200_.3           1.000                               0.474    0.523
##     s200_.3 (ojc1)    0.998    0.042   23.534    0.000    0.473    0.543
##     s200_.3 (ojc2)    1.350    0.060   22.478    0.000    0.640    0.864
##     s200_.3 (ojc3)    1.397    0.066   21.235    0.000    0.662    0.796
##     s200_.3 (ojc4)    1.294    0.062   20.779    0.000    0.613    0.775
##     s200_.3 (ojc5)    1.198    0.062   19.281    0.000    0.568    0.674
##   OJC_W2 =~                                                             
##     s200_.4           1.000                               0.514    0.568
##     s200_.4 (ojc1)    0.998    0.042   23.534    0.000    0.513    0.612
##     s200_.4 (ojc2)    1.350    0.060   22.478    0.000    0.694    0.787
##     s200_.4 (ojc3)    1.397    0.066   21.235    0.000    0.718    0.791
##     s200_.4 (ojc4)    1.294    0.062   20.779    0.000    0.665    0.804
##     s200_.4 (ojc5)    1.198    0.062   19.281    0.000    0.616    0.634
##   OJC_W3 =~                                                             
##     s200_.6           1.000                               0.382    0.509
##     s200_.6 (ojc1)    0.998    0.042   23.534    0.000    0.381    0.528
##     s200_.6 (ojc2)    1.350    0.060   22.478    0.000    0.515    0.803
##     s200_.6 (ojc3)    1.397    0.066   21.235    0.000    0.533    0.735
##     s200_.6 (ojc4)    1.294    0.062   20.779    0.000    0.494    0.741
##     s200_.6 (ojc5)    1.198    0.062   19.281    0.000    0.457    0.545
##   OJC_W4 =~                                                             
##     s200_.7           1.000                               0.384    0.447
##     s200_.7 (ojc1)    0.998    0.042   23.534    0.000    0.383    0.527
##     s200_.7 (ojc2)    1.350    0.060   22.478    0.000    0.518    0.773
##     s200_.7 (ojc3)    1.397    0.066   21.235    0.000    0.537    0.824
##     s200_.7 (ojc4)    1.294    0.062   20.779    0.000    0.497    0.751
##     s200_.7 (ojc5)    1.198    0.062   19.281    0.000    0.460    0.608
##   dojc2 =~                                                              
##     OJC_W2            1.000                               0.940    0.940
##   dojc3 =~                                                              
##     OJC_W3            1.000                               1.088    1.088
##   dojc4 =~                                                              
##     OJC_W4            1.000                               0.978    0.978
##   SRH_W1 =~                                                             
##     s38.3r            1.000                               0.651    0.856
##   SRH_W2 =~                                                             
##     s38.4r            1.000                               0.643    0.853
##   SRH_W3 =~                                                             
##     s38.6             1.000                               0.655    0.858
##   SRH_W4 =~                                                             
##     s38.7             1.000                               0.622    0.845
## 
## Regressions:
##                    Estimate  Std.Err  z-value  P(>|z|)   Std.lv  Std.all
##   JC_W2 ~                                                               
##     JC_W1             1.000                               0.893    0.893
##   JC_W3 ~                                                               
##     JC_W2             1.000                               1.253    1.253
##   JC_W4 ~                                                               
##     JC_W3             1.000                               0.736    0.736
##   OJC_W2 ~                                                              
##     OJC_W1            1.000                               0.922    0.922
##   OJC_W3 ~                                                              
##     OJC_W2            1.000                               1.347    1.347
##   OJC_W4 ~                                                              
##     OJC_W3            1.000                               0.994    0.994
##   SRH_W2 ~                                                              
##     SRH_W1  (ar1_)    0.963    0.053   18.044    0.000    0.975    0.975
##   SRH_W3 ~                                                              
##     SRH_W2  (ar2_)    0.925    0.049   18.977    0.000    0.908    0.908
##   SRH_W4 ~                                                              
##     SRH_W3  (ar3_)    0.956    0.058   16.468    0.000    1.006    1.006
##   SRH_W2 ~                                                              
##     djc2    (jcs1)    0.424    0.204    2.081    0.037    0.094    0.094
##     dojc2   (ojc1)    0.107    0.062    1.728    0.084    0.080    0.080
##     c137.6  (cvd1)   -0.056    0.070   -0.795    0.427   -0.087   -0.043
##   SRH_W3 ~                                                              
##     djc3    (jcs2)    0.692    0.284    2.431    0.015    0.139    0.139
##     dojc3   (ojc2)    0.100    0.064    1.561    0.119    0.064    0.064
##     c137.6  (cvd2)    0.029    0.066    0.431    0.666    0.044    0.021
##   SRH_W4 ~                                                              
##     djc4    (jcs3)   -0.101    0.237   -0.428    0.669   -0.022   -0.022
##     dojc4   (ojc3)    0.239    0.093    2.568    0.010    0.144    0.144
##     c137.6  (cvd3)    0.113    0.077    1.467    0.142    0.181    0.089
## 
## Covariances:
##                    Estimate  Std.Err  z-value  P(>|z|)   Std.lv  Std.all
##  .s21_rd.3 ~~                                                           
##    .s21_r.4           0.237    0.042    5.684    0.000    0.237    0.618
##    .s21_r.6           0.296    0.052    5.716    0.000    0.296    0.643
##    .s21_r.7           0.259    0.054    4.816    0.000    0.259    0.542
##  .s21_rd.4 ~~                                                           
##    .s21_r.6           0.230    0.042    5.468    0.000    0.230    0.569
##    .s21_r.7           0.188    0.047    4.006    0.000    0.188    0.447
##  .s21_rd.6 ~~                                                           
##    .s21_r.7           0.356    0.062    5.785    0.000    0.356    0.709
##  .s21_str.3 ~~                                                          
##    .s21_s.4           0.159    0.030    5.264    0.000    0.159    0.625
##    .s21_s.6           0.185    0.034    5.447    0.000    0.185    0.679
##    .s21_s.7           0.167    0.036    4.662    0.000    0.167    0.631
##  .s21_str.4 ~~                                                          
##    .s21_s.6           0.158    0.030    5.212    0.000    0.158    0.610
##    .s21_s.7           0.149    0.032    4.621    0.000    0.149    0.594
##  .s21_str.6 ~~                                                          
##    .s21_s.7           0.196    0.036    5.456    0.000    0.196    0.728
##  .s21_cd.3 ~~                                                           
##    .s21_c.4           0.309    0.058    5.324    0.000    0.309    0.645
##    .s21_c.6           0.425    0.071    6.006    0.000    0.425    0.782
##    .s21_c.7           0.258    0.058    4.465    0.000    0.258    0.651
##  .s21_cd.4 ~~                                                           
##    .s21_c.6           0.411    0.063    6.477    0.000    0.411    0.796
##    .s21_c.7           0.253    0.051    4.942    0.000    0.253    0.671
##  .s21_cd.6 ~~                                                           
##    .s21_c.7           0.278    0.059    4.694    0.000    0.278    0.650
##  .s21_soz.3 ~~                                                          
##    .s21_s.4           0.127    0.030    4.270    0.000    0.127    0.610
##    .s21_s.6           0.132    0.032    4.082    0.000    0.132    0.603
##    .s21_s.7           0.169    0.035    4.829    0.000    0.169    0.738
##  .s21_soz.4 ~~                                                          
##    .s21_s.6           0.108    0.028    3.798    0.000    0.108    0.529
##    .s21_s.7           0.167    0.033    5.079    0.000    0.167    0.778
##  .s21_soz.6 ~~                                                          
##    .s21_s.7           0.152    0.035    4.307    0.000    0.152    0.676
##   djc2 ~~                                                               
##     djc3             -0.003    0.005   -0.698    0.485   -0.181   -0.181
##     djc4              0.004    0.005    0.756    0.449    0.190    0.190
##   djc3 ~~                                                               
##     djc4             -0.009    0.005   -1.939    0.052   -0.514   -0.514
##  .s200_de.3 ~~                                                          
##    .s200_.3           0.184    0.053    3.478    0.001    0.184    0.326
##  .s200_de.4 ~~                                                          
##    .s200_.4           0.131    0.040    3.249    0.001    0.131    0.265
##  .s200_de.6 ~~                                                          
##    .s200_.6           0.167    0.041    4.077    0.000    0.167    0.421
##  .s200_de.7 ~~                                                          
##    .s200_.7           0.267    0.059    4.526    0.000    0.267    0.561
##  .s200_de.3 ~~                                                          
##    .s200_.4           0.232    0.055    4.215    0.000    0.232    0.403
##    .s200_.6           0.213    0.049    4.335    0.000    0.213    0.427
##    .s200_.7           0.152    0.057    2.683    0.007    0.152    0.256
##  .s200_de.4 ~~                                                          
##    .s200_.6           0.185    0.043    4.287    0.000    0.185    0.385
##    .s200_.7           0.201    0.052    3.874    0.000    0.201    0.351
##  .s200_de.6 ~~                                                          
##    .s200_.7           0.173    0.046    3.755    0.000    0.173    0.348
##  .s200_re.3 ~~                                                          
##    .s200_.4           0.152    0.045    3.422    0.001    0.152    0.314
##    .s200_.6           0.135    0.043    3.136    0.002    0.135    0.302
##    .s200_.7           0.145    0.042    3.446    0.001    0.145    0.320
##  .s200_re.4 ~~                                                          
##    .s200_.6           0.074    0.036    2.076    0.038    0.074    0.182
##    .s200_.7           0.085    0.035    2.418    0.016    0.085    0.208
##  .s200_re.6 ~~                                                          
##    .s200_.7           0.064    0.034    1.889    0.059    0.064    0.170
##  .s200_au.3 ~~                                                          
##    .s200_.4           0.066    0.029    2.272    0.023    0.066    0.327
##    .s200_.6           0.061    0.025    2.490    0.013    0.061    0.431
##    .s200_.7           0.019    0.026    0.714    0.475    0.019    0.117
##  .s200_au.4 ~~                                                          
##    .s200_.6           0.029    0.028    1.037    0.300    0.029    0.139
##    .s200_.7           0.034    0.032    1.069    0.285    0.034    0.147
##  .s200_au.6 ~~                                                          
##    .s200_.7           0.024    0.027    0.892    0.373    0.024    0.148
##  .s200_ma.3 ~~                                                          
##    .s200_.4           0.071    0.035    2.029    0.042    0.071    0.252
##    .s200_.6           0.014    0.034    0.403    0.687    0.014    0.055
##    .s200_.7           0.048    0.028    1.724    0.085    0.048    0.257
##  .s200_ma.4 ~~                                                          
##    .s200_.6           0.078    0.032    2.420    0.016    0.078    0.284
##    .s200_.7           0.057    0.031    1.872    0.061    0.057    0.279
##  .s200_ma.6 ~~                                                          
##    .s200_.7           0.076    0.030    2.511    0.012    0.076    0.415
##  .s200_me.3 ~~                                                          
##    .s200_.4           0.083    0.032    2.565    0.010    0.083    0.335
##    .s200_.6           0.021    0.034    0.617    0.537    0.021    0.095
##    .s200_.7           0.030    0.032    0.936    0.349    0.030    0.135
##  .s200_me.4 ~~                                                          
##    .s200_.6           0.017    0.028    0.601    0.548    0.017    0.077
##    .s200_.7           0.008    0.030    0.254    0.800    0.008    0.036
##  .s200_me.6 ~~                                                          
##    .s200_.7           0.056    0.033    1.679    0.093    0.056    0.287
##  .s200_af.3 ~~                                                          
##    .s200_.4           0.182    0.052    3.479    0.001    0.182    0.390
##    .s200_.6           0.182    0.056    3.243    0.001    0.182    0.415
##    .s200_.7           0.210    0.048    4.382    0.000    0.210    0.562
##  .s200_af.4 ~~                                                          
##    .s200_.6           0.176    0.054    3.293    0.001    0.176    0.334
##    .s200_.7           0.170    0.050    3.391    0.001    0.170    0.377
##  .s200_af.6 ~~                                                          
##    .s200_.7           0.201    0.053    3.831    0.000    0.201    0.476
##   dojc2 ~~                                                              
##     dojc3            -0.096    0.029   -3.294    0.001   -0.478   -0.478
##     dojc4            -0.052    0.030   -1.748    0.081   -0.287   -0.287
##   dojc3 ~~                                                              
##     dojc4            -0.042    0.022   -1.890    0.059   -0.271   -0.271
##   djc2 ~~                                                               
##     dojc2   (cov1)    0.010    0.003    3.239    0.001    0.141    0.141
##   djc3 ~~                                                               
##     dojc3   (cov1)    0.010    0.003    3.239    0.001    0.178    0.178
##   djc4 ~~                                                               
##     dojc4   (cov1)    0.010    0.003    3.239    0.001    0.194    0.194
##   JC_W1 ~~                                                              
##     c137.6            0.001    0.014    0.093    0.926    0.006    0.012
##   OJC_W1 ~~                                                             
##     c137.6            0.056    0.030    1.881    0.060    0.118    0.239
##   SRH_W1 ~~                                                             
##     c137.6           -0.137    0.038   -3.600    0.000   -0.211   -0.429
##   djc2 ~~                                                               
##     c137.6            0.001    0.011    0.091    0.927    0.007    0.015
##   djc3 ~~                                                               
##     c137.6           -0.008    0.010   -0.806    0.420   -0.062   -0.126
##   djc4 ~~                                                               
##     c137.6           -0.013    0.011   -1.170    0.242   -0.097   -0.196
##   dojc2 ~~                                                              
##     c137.6            0.010    0.029    0.354    0.724    0.021    0.043
##   dojc3 ~~                                                              
##     c137.6           -0.034    0.023   -1.461    0.144   -0.083   -0.168
##   dojc4 ~~                                                              
##     c137.6            0.020    0.023    0.850    0.395    0.052    0.106
##   JC_W1 ~~                                                              
##     djc2             -0.004    0.006   -0.730    0.465   -0.140   -0.140
##     djc3             -0.016    0.006   -2.552    0.011   -0.558   -0.558
##     djc4              0.012    0.006    1.913    0.056    0.422    0.422
##     OJC_W1            0.036    0.014    2.575    0.010    0.353    0.353
##     dojc2            -0.004    0.013   -0.296    0.767   -0.036   -0.036
##     dojc3            -0.003    0.012   -0.275    0.784   -0.038   -0.038
##     dojc4             0.006    0.013    0.490    0.624    0.079    0.079
##     SRH_W1            0.006    0.017    0.334    0.739    0.040    0.040
##   djc2 ~~                                                               
##     OJC_W1           -0.014    0.009   -1.625    0.104   -0.210   -0.210
##     dojc3            -0.009    0.009   -0.968    0.333   -0.151   -0.151
##     dojc4             0.008    0.010    0.796    0.426    0.148    0.148
##     SRH_W1            0.002    0.013    0.184    0.854    0.026    0.026
##   djc3 ~~                                                               
##     OJC_W1           -0.000    0.012   -0.012    0.990   -0.002   -0.002
##     dojc2            -0.007    0.010   -0.720    0.472   -0.116   -0.116
##     dojc4            -0.011    0.007   -1.432    0.152   -0.215   -0.215
##     SRH_W1            0.020    0.014    1.474    0.141    0.236    0.236
##   djc4 ~~                                                               
##     OJC_W1            0.009    0.013    0.725    0.469    0.147    0.147
##     dojc2             0.012    0.012    1.004    0.315    0.188    0.188
##     dojc3            -0.024    0.009   -2.668    0.008   -0.423   -0.423
##     SRH_W1            0.008    0.014    0.575    0.565    0.096    0.096
##   OJC_W1 ~~                                                             
##     dojc2            -0.097    0.028   -3.508    0.000   -0.423   -0.423
##     dojc3            -0.050    0.028   -1.804    0.071   -0.252   -0.252
##     dojc4             0.025    0.029    0.841    0.400    0.138    0.138
##     SRH_W1            0.089    0.032    2.793    0.005    0.290    0.290
##   dojc2 ~~                                                              
##     SRH_W1           -0.050    0.033   -1.507    0.132   -0.160   -0.160
##   dojc3 ~~                                                              
##     SRH_W1            0.020    0.031    0.636    0.525    0.074    0.074
##   dojc4 ~~                                                              
##     SRH_W1           -0.013    0.032   -0.417    0.677   -0.054   -0.054
## 
## Intercepts:
##                    Estimate  Std.Err  z-value  P(>|z|)   Std.lv  Std.all
##    .s        (jc6)   -0.172    0.337   -0.509    0.611   -0.172   -0.288
##    .s        (jc6)   -0.172    0.337   -0.509    0.611   -0.172   -0.290
##    .s        (jc6)   -0.172    0.337   -0.509    0.611   -0.172   -0.291
##    .s        (jc6)   -0.172    0.337   -0.509    0.611   -0.172   -0.275
##    .s        (jc4)   -3.271    0.478   -6.837    0.000   -3.271   -3.945
##    .s        (jc4)   -3.271    0.478   -6.837    0.000   -3.271   -3.951
##    .s        (jc4)   -3.271    0.478   -6.837    0.000   -3.271   -3.819
##    .s        (jc4)   -3.271    0.478   -6.837    0.000   -3.271   -4.282
##    .s        (jc5)   -2.496    0.414   -6.023    0.000   -2.496   -4.116
##    .s        (jc5)   -2.496    0.414   -6.023    0.000   -2.496   -4.070
##    .s        (jc5)   -2.496    0.414   -6.023    0.000   -2.496   -4.349
##    .s        (jc5)   -2.496    0.414   -6.023    0.000   -2.496   -3.725
##     J                 2.885    0.029   99.222    0.000   13.381   13.381
##    .J                 0.000                               0.000    0.000
##    .J                 0.000                               0.000    0.000
##    .J                 0.000                               0.000    0.000
##    .s                 0.000                               0.000    0.000
##    .s                 0.000                               0.000    0.000
##    .s                 0.000                               0.000    0.000
##    .s                 0.000                               0.000    0.000
##    .s       (ojc6)   -0.133    0.166   -0.802    0.423   -0.133   -0.153
##    .s       (ojc6)   -0.133    0.166   -0.802    0.423   -0.133   -0.159
##    .s       (ojc6)   -0.133    0.166   -0.802    0.423   -0.133   -0.184
##    .s       (ojc6)   -0.133    0.166   -0.802    0.423   -0.133   -0.183
##    .s       (ojc7)   -1.452    0.235   -6.175    0.000   -1.452   -1.960
##    .s       (ojc7)   -1.452    0.235   -6.175    0.000   -1.452   -1.646
##    .s       (ojc7)   -1.452    0.235   -6.175    0.000   -1.452   -2.263
##    .s       (ojc7)   -1.452    0.235   -6.175    0.000   -1.452   -2.165
##    .s       (ojc8)   -2.012    0.257   -7.815    0.000   -2.012   -2.417
##    .s       (ojc8)   -2.012    0.257   -7.815    0.000   -2.012   -2.215
##    .s       (ojc8)   -2.012    0.257   -7.815    0.000   -2.012   -2.771
##    .s       (ojc8)   -2.012    0.257   -7.815    0.000   -2.012   -3.087
##    .s       (ojc9)   -1.481    0.244   -6.075    0.000   -1.481   -1.870
##    .s       (ojc9)   -1.481    0.244   -6.075    0.000   -1.481   -1.789
##    .s       (ojc9)   -1.481    0.244   -6.075    0.000   -1.481   -2.222
##    .s       (ojc9)   -1.481    0.244   -6.075    0.000   -1.481   -2.238
##    .s       (o10_)   -0.927    0.247   -3.762    0.000   -0.927   -1.101
##    .s       (o10_)   -0.927    0.247   -3.762    0.000   -0.927   -0.955
##    .s       (o10_)   -0.927    0.247   -3.762    0.000   -0.927   -1.105
##    .s       (o10_)   -0.927    0.247   -3.762    0.000   -0.927   -1.225
##     O                 3.978    0.051   78.651    0.000    8.392    8.392
##    .O                 0.000                               0.000    0.000
##    .O                 0.000                               0.000    0.000
##    .O                 0.000                               0.000    0.000
##    .s                 0.000                               0.000    0.000
##    .s                 0.000                               0.000    0.000
##    .s                 0.000                               0.000    0.000
##    .s                 0.000                               0.000    0.000
##     d       (gm_2)   -0.046    0.010   -4.726    0.000   -0.325   -0.325
##     d       (gm_3)    0.004    0.009    0.432    0.666    0.030    0.030
##     d  (gamma_jc4)   -0.061    0.012   -5.019    0.000   -0.456   -0.456
##     d                -0.148    0.047   -3.136    0.002   -0.307   -0.307
##     d                 0.002    0.040    0.059    0.953    0.006    0.006
##     d (gamma_ojc4)    0.037    0.021    1.787    0.074    0.098    0.098
##    .s                 0.000                               0.000    0.000
##    .s                 0.000                               0.000    0.000
##    .s                 0.000                               0.000    0.000
##    .s                 0.000                               0.000    0.000
##     S                 3.630    0.065   55.699    0.000    5.578    5.578
##    .S                 0.247    0.211    1.169    0.242    0.384    0.384
##    .S                 0.334    0.199    1.684    0.092    0.511    0.511
##    .S                 0.066    0.236    0.278    0.781    0.106    0.106
##     c                 0.328    0.047    6.968    0.000    0.328    0.667
## 
## Variances:
##                    Estimate  Std.Err  z-value  P(>|z|)   Std.lv  Std.all
##     JC_W1             0.046    0.012    3.792    0.000    1.000    1.000
##    .JC_W2             0.000                               0.000    0.000
##    .JC_W3             0.000                               0.000    0.000
##    .JC_W4             0.000                               0.000    0.000
##     djc2              0.020    0.007    3.039    0.002    1.000    1.000
##     djc3              0.017    0.006    2.865    0.004    1.000    1.000
##     djc4              0.018    0.006    2.767    0.006    1.000    1.000
##     OJC_W1            0.225    0.037    6.037    0.000    1.000    1.000
##    .OJC_W2            0.000                               0.000    0.000
##    .OJC_W3            0.000                               0.000    0.000
##    .OJC_W4            0.000                               0.000    0.000
##     dojc2             0.233    0.041    5.736    0.000    1.000    1.000
##     dojc3             0.173    0.033    5.182    0.000    1.000    1.000
##     dojc4             0.141    0.030    4.676    0.000    1.000    1.000
##    .s38.3r  (rs_s)    0.154    0.013   12.236    0.000    0.154    0.267
##    .s38.4r  (rs_s)    0.154    0.013   12.236    0.000    0.154    0.272
##    .s38.6   (rs_s)    0.154    0.013   12.236    0.000    0.154    0.264
##    .s38.7   (rs_s)    0.154    0.013   12.236    0.000    0.154    0.285
##    .s21_r.3           0.436    0.059    7.345    0.000    0.436    0.904
##    .s21_s.3           0.268    0.041    6.593    0.000    0.268    0.754
##    .s21_c.3           0.503    0.078    6.445    0.000    0.503    0.732
##    .s21_s.3           0.223    0.039    5.682    0.000    0.223    0.606
##    .s21_r.4           0.337    0.040    8.404    0.000    0.337    0.853
##    .s21_s.4           0.241    0.032    7.426    0.000    0.241    0.688
##    .s21_c.4           0.454    0.062    7.350    0.000    0.454    0.663
##    .s21_s.4           0.195    0.032    6.111    0.000    0.195    0.518
##    .s21_r.6           0.484    0.063    7.632    0.000    0.484    0.929
##    .s21_s.6           0.278    0.041    6.862    0.000    0.278    0.800
##    .s21_c.6           0.586    0.083    7.039    0.000    0.586    0.799
##    .s21_s.6           0.214    0.038    5.608    0.000    0.214    0.649
##    .s21_r.7           0.522    0.079    6.626    0.000    0.522    0.884
##    .s21_s.7           0.262    0.044    5.951    0.000    0.262    0.671
##    .s21_c.7           0.312    0.060    5.220    0.000    0.312    0.534
##    .s21_s.7           0.235    0.045    5.289    0.000    0.235    0.525
##    .s200_.3           0.596    0.076    7.891    0.000    0.596    0.726
##    .s200_.3           0.535    0.072    7.380    0.000    0.535    0.705
##    .s200_.3           0.140    0.029    4.742    0.000    0.140    0.254
##    .s200_.3           0.254    0.043    5.940    0.000    0.254    0.367
##    .s200_.3           0.251    0.042    6.021    0.000    0.251    0.400
##    .s200_.3           0.387    0.060    6.452    0.000    0.387    0.545
##    .s200_.4           0.554    0.065    8.480    0.000    0.554    0.677
##    .s200_.4           0.440    0.051    8.573    0.000    0.440    0.626
##    .s200_.4           0.296    0.044    6.740    0.000    0.296    0.381
##    .s200_.4           0.309    0.045    6.803    0.000    0.309    0.375
##    .s200_.4           0.242    0.037    6.583    0.000    0.242    0.354
##    .s200_.4           0.564    0.070    8.064    0.000    0.564    0.598
##    .s200_.6           0.417    0.054    7.682    0.000    0.417    0.741
##    .s200_.6           0.376    0.053    7.139    0.000    0.376    0.721
##    .s200_.6           0.146    0.029    5.068    0.000    0.146    0.355
##    .s200_.6           0.243    0.041    5.954    0.000    0.243    0.460
##    .s200_.6           0.200    0.034    5.868    0.000    0.200    0.450
##    .s200_.6           0.495    0.072    6.889    0.000    0.495    0.703
##    .s200_.7           0.590    0.085    6.911    0.000    0.590    0.800
##    .s200_.7           0.382    0.058    6.632    0.000    0.382    0.722
##    .s200_.7           0.181    0.036    4.972    0.000    0.181    0.402
##    .s200_.7           0.137    0.031    4.354    0.000    0.137    0.322
##    .s200_.7           0.191    0.038    5.060    0.000    0.191    0.436
##    .s200_.7           0.361    0.059    6.109    0.000    0.361    0.630
##     c137.6            0.242    0.036    6.652    0.000    0.242    1.000
##     SRH_W1            0.423    0.062    6.826    0.000    1.000    1.000
##    .SRH_W2            0.006    0.029    0.199    0.842    0.014    0.014
##    .SRH_W3            0.048    0.032    1.507    0.132    0.112    0.112
##    .SRH_W4            0.024    0.043    0.557    0.578    0.062    0.062
## 
## 
## Group 2 [FOW]:
## 
## Latent Variables:
##                    Estimate  Std.Err  z-value  P(>|z|)   Std.lv  Std.all
##   JC_W1 =~                                                              
##     s21_r.3           1.000                               0.256    0.361
##     s21_s.3  (jc1)    1.369    0.118   11.648    0.000    0.351    0.559
##     s21_c.3  (jc2)    1.992    0.167   11.921    0.000    0.511    0.609
##     s21_s.3  (jc3)    1.765    0.145   12.186    0.000    0.453    0.742
##   JC_W2 =~                                                              
##     s21_r.4           1.000                               0.276    0.398
##     s21_s.4  (jc1)    1.369    0.118   11.648    0.000    0.378    0.579
##     s21_c.4  (jc2)    1.992    0.167   11.921    0.000    0.549    0.647
##     s21_s.4  (jc3)    1.765    0.145   12.186    0.000    0.487    0.753
##   JC_W3 =~                                                              
##     s21_r.6           1.000                               0.273    0.406
##     s21_s.6  (jc1)    1.369    0.118   11.648    0.000    0.373    0.550
##     s21_c.6  (jc2)    1.992    0.167   11.921    0.000    0.543    0.629
##     s21_s.6  (jc3)    1.765    0.145   12.186    0.000    0.481    0.740
##   JC_W4 =~                                                              
##     s21_r.7           1.000                               0.282    0.389
##     s21_s.7  (jc1)    1.369    0.118   11.648    0.000    0.386    0.537
##     s21_c.7  (jc2)    1.992    0.167   11.921    0.000    0.561    0.694
##     s21_s.7  (jc3)    1.765    0.145   12.186    0.000    0.497    0.759
##   djc2 =~                                                               
##     JC_W2             1.000                               0.589    0.589
##   djc3 =~                                                               
##     JC_W3             1.000                               0.504    0.504
##   djc4 =~                                                               
##     JC_W4             1.000                               0.661    0.661
##   OJC_W1 =~                                                             
##     s200_.3           1.000                               0.426    0.518
##     s200_.3 (ojc1)    0.998    0.042   23.534    0.000    0.425    0.547
##     s200_.3 (ojc2)    1.350    0.060   22.478    0.000    0.575    0.746
##     s200_.3 (ojc3)    1.397    0.066   21.235    0.000    0.595    0.683
##     s200_.3 (ojc4)    1.294    0.062   20.779    0.000    0.551    0.751
##     s200_.3 (ojc5)    1.198    0.062   19.281    0.000    0.511    0.618
##   OJC_W2 =~                                                             
##     s200_.4           1.000                               0.472    0.520
##     s200_.4 (ojc1)    0.998    0.042   23.534    0.000    0.471    0.598
##     s200_.4 (ojc2)    1.350    0.060   22.478    0.000    0.637    0.798
##     s200_.4 (ojc3)    1.397    0.066   21.235    0.000    0.659    0.777
##     s200_.4 (ojc4)    1.294    0.062   20.779    0.000    0.611    0.766
##     s200_.4 (ojc5)    1.198    0.062   19.281    0.000    0.565    0.620
##   OJC_W3 =~                                                             
##     s200_.6           1.000                               0.493    0.549
##     s200_.6 (ojc1)    0.998    0.042   23.534    0.000    0.492    0.612
##     s200_.6 (ojc2)    1.350    0.060   22.478    0.000    0.665    0.842
##     s200_.6 (ojc3)    1.397    0.066   21.235    0.000    0.689    0.847
##     s200_.6 (ojc4)    1.294    0.062   20.779    0.000    0.638    0.793
##     s200_.6 (ojc5)    1.198    0.062   19.281    0.000    0.591    0.651
##   OJC_W4 =~                                                             
##     s200_.7           1.000                               0.512    0.594
##     s200_.7 (ojc1)    0.998    0.042   23.534    0.000    0.511    0.623
##     s200_.7 (ojc2)    1.350    0.060   22.478    0.000    0.691    0.849
##     s200_.7 (ojc3)    1.397    0.066   21.235    0.000    0.715    0.818
##     s200_.7 (ojc4)    1.294    0.062   20.779    0.000    0.662    0.800
##     s200_.7 (ojc5)    1.198    0.062   19.281    0.000    0.613    0.737
##   dojc2 =~                                                              
##     OJC_W2            1.000                               0.836    0.836
##   dojc3 =~                                                              
##     OJC_W3            1.000                               0.854    0.854
##   dojc4 =~                                                              
##     OJC_W4            1.000                               0.769    0.769
##   SRH_W1 =~                                                             
##     s38.3r            1.000                               0.667    0.862
##   SRH_W2 =~                                                             
##     s38.4r            1.000                               0.674    0.864
##   SRH_W3 =~                                                             
##     s38.6             1.000                               0.665    0.861
##   SRH_W4 =~                                                             
##     s38.7             1.000                               0.684    0.867
## 
## Regressions:
##                    Estimate  Std.Err  z-value  P(>|z|)   Std.lv  Std.all
##   JC_W2 ~                                                               
##     JC_W1             1.000                               0.930    0.930
##   JC_W3 ~                                                               
##     JC_W2             1.000                               1.011    1.011
##   JC_W4 ~                                                               
##     JC_W3             1.000                               0.968    0.968
##   OJC_W2 ~                                                              
##     OJC_W1            1.000                               0.903    0.903
##   OJC_W3 ~                                                              
##     OJC_W2            1.000                               0.958    0.958
##   OJC_W4 ~                                                              
##     OJC_W3            1.000                               0.963    0.963
##   SRH_W2 ~                                                              
##     SRH_W1  (ar1_)    0.963    0.053   18.044    0.000    0.953    0.953
##   SRH_W3 ~                                                              
##     SRH_W2  (ar2_)    0.925    0.049   18.977    0.000    0.939    0.939
##   SRH_W4 ~                                                              
##     SRH_W3  (ar3_)    0.956    0.058   16.468    0.000    0.929    0.929
##   SRH_W2 ~                                                              
##     djc2    (jcs1)    0.424    0.204    2.081    0.037    0.102    0.102
##     dojc2   (ojc1)    0.107    0.062    1.728    0.084    0.062    0.062
##     c137.6  (cvd1)   -0.056    0.070   -0.795    0.427   -0.083   -0.037
##   SRH_W3 ~                                                              
##     djc3    (jcs2)    0.692    0.284    2.431    0.015    0.143    0.143
##     dojc3   (ojc2)    0.100    0.064    1.561    0.119    0.063    0.063
##     c137.6  (cvd2)    0.029    0.066    0.431    0.666    0.043    0.019
##   SRH_W4 ~                                                              
##     djc4    (jcs3)   -0.101    0.237   -0.428    0.669   -0.028   -0.028
##     dojc4   (ojc3)    0.239    0.093    2.568    0.010    0.137    0.137
##     c137.6  (cvd3)    0.113    0.077    1.467    0.142    0.165    0.074
## 
## Covariances:
##                    Estimate  Std.Err  z-value  P(>|z|)   Std.lv  Std.all
##  .s21_rd.3 ~~                                                           
##    .s21_r.4           0.207    0.026    7.837    0.000    0.207    0.493
##    .s21_r.6           0.240    0.031    7.764    0.000    0.240    0.592
##    .s21_r.7           0.262    0.034    7.768    0.000    0.262    0.594
##  .s21_rd.4 ~~                                                           
##    .s21_r.6           0.223    0.027    8.398    0.000    0.223    0.572
##    .s21_r.7           0.231    0.030    7.617    0.000    0.231    0.545
##  .s21_rd.6 ~~                                                           
##    .s21_r.7           0.216    0.032    6.859    0.000    0.216    0.528
##  .s21_str.3 ~~                                                          
##    .s21_s.4           0.158    0.020    7.960    0.000    0.158    0.572
##    .s21_s.6           0.190    0.024    7.906    0.000    0.190    0.643
##    .s21_s.7           0.223    0.026    8.419    0.000    0.223    0.705
##  .s21_str.4 ~~                                                          
##    .s21_s.6           0.207    0.023    9.054    0.000    0.207    0.686
##    .s21_s.7           0.212    0.025    8.400    0.000    0.212    0.656
##  .s21_str.6 ~~                                                          
##    .s21_s.7           0.238    0.029    8.219    0.000    0.238    0.692
##  .s21_cd.3 ~~                                                           
##    .s21_c.4           0.265    0.033    7.932    0.000    0.265    0.615
##    .s21_c.6           0.305    0.039    7.844    0.000    0.305    0.683
##    .s21_c.7           0.236    0.037    6.310    0.000    0.236    0.609
##  .s21_cd.4 ~~                                                           
##    .s21_c.6           0.297    0.036    8.174    0.000    0.297    0.684
##    .s21_c.7           0.221    0.034    6.466    0.000    0.221    0.587
##  .s21_cd.6 ~~                                                           
##    .s21_c.7           0.262    0.038    6.825    0.000    0.262    0.670
##  .s21_soz.3 ~~                                                          
##    .s21_s.4           0.090    0.017    5.146    0.000    0.090    0.515
##    .s21_s.6           0.096    0.020    4.702    0.000    0.096    0.535
##    .s21_s.7           0.084    0.020    4.192    0.000    0.084    0.483
##  .s21_soz.4 ~~                                                          
##    .s21_s.6           0.088    0.020    4.516    0.000    0.088    0.476
##    .s21_s.7           0.058    0.020    2.887    0.004    0.058    0.317
##  .s21_soz.6 ~~                                                          
##    .s21_s.7           0.090    0.022    4.072    0.000    0.090    0.485
##   djc2 ~~                                                               
##     djc3             -0.007    0.004   -1.981    0.048   -0.321   -0.321
##     djc4             -0.003    0.004   -0.839    0.401   -0.115   -0.115
##   djc3 ~~                                                               
##     djc4             -0.013    0.005   -2.846    0.004   -0.518   -0.518
##  .s200_de.3 ~~                                                          
##    .s200_.3           0.106    0.026    4.029    0.000    0.106    0.233
##  .s200_de.4 ~~                                                          
##    .s200_.4           0.128    0.024    5.245    0.000    0.128    0.263
##  .s200_de.6 ~~                                                          
##    .s200_.6           0.174    0.030    5.830    0.000    0.174    0.365
##  .s200_de.7 ~~                                                          
##    .s200_.7           0.154    0.029    5.341    0.000    0.154    0.346
##  .s200_de.3 ~~                                                          
##    .s200_.4           0.224    0.035    6.468    0.000    0.224    0.411
##    .s200_.6           0.178    0.034    5.169    0.000    0.178    0.337
##    .s200_.7           0.205    0.034    6.059    0.000    0.205    0.420
##  .s200_de.4 ~~                                                          
##    .s200_.6           0.173    0.035    4.992    0.000    0.173    0.297
##    .s200_.7           0.171    0.034    5.088    0.000    0.171    0.318
##  .s200_de.6 ~~                                                          
##    .s200_.7           0.238    0.035    6.750    0.000    0.238    0.458
##  .s200_re.3 ~~                                                          
##    .s200_.4           0.155    0.026    5.908    0.000    0.155    0.378
##    .s200_.6           0.144    0.028    5.099    0.000    0.144    0.348
##    .s200_.7           0.157    0.029    5.458    0.000    0.157    0.377
##  .s200_re.4 ~~                                                          
##    .s200_.6           0.176    0.025    6.987    0.000    0.176    0.440
##    .s200_.7           0.167    0.026    6.330    0.000    0.167    0.412
##  .s200_re.6 ~~                                                          
##    .s200_.7           0.149    0.028    5.279    0.000    0.149    0.366
##  .s200_au.3 ~~                                                          
##    .s200_.4           0.076    0.019    3.945    0.000    0.076    0.309
##    .s200_.6           0.057    0.020    2.896    0.004    0.057    0.258
##    .s200_.7           0.043    0.022    1.970    0.049    0.043    0.194
##  .s200_au.4 ~~                                                          
##    .s200_.6           0.043    0.018    2.446    0.014    0.043    0.209
##    .s200_.7           0.031    0.019    1.610    0.107    0.031    0.150
##  .s200_au.6 ~~                                                          
##    .s200_.7           0.074    0.020    3.710    0.000    0.074    0.404
##  .s200_ma.3 ~~                                                          
##    .s200_.4           0.114    0.026    4.443    0.000    0.114    0.335
##    .s200_.6           0.132    0.024    5.423    0.000    0.132    0.482
##    .s200_.7           0.093    0.028    3.298    0.001    0.093    0.290
##  .s200_ma.4 ~~                                                          
##    .s200_.6           0.084    0.020    4.245    0.000    0.084    0.365
##    .s200_.7           0.100    0.023    4.336    0.000    0.100    0.371
##  .s200_ma.6 ~~                                                          
##    .s200_.7           0.085    0.021    3.994    0.000    0.085    0.394
##  .s200_me.3 ~~                                                          
##    .s200_.4           0.085    0.019    4.540    0.000    0.085    0.344
##    .s200_.6           0.075    0.021    3.648    0.000    0.075    0.317
##    .s200_.7           0.092    0.022    4.216    0.000    0.092    0.383
##  .s200_me.4 ~~                                                          
##    .s200_.6           0.088    0.020    4.323    0.000    0.088    0.351
##    .s200_.7           0.097    0.022    4.349    0.000    0.097    0.381
##  .s200_me.6 ~~                                                          
##    .s200_.7           0.094    0.023    4.023    0.000    0.094    0.385
##  .s200_af.3 ~~                                                          
##    .s200_.4           0.208    0.031    6.664    0.000    0.208    0.448
##    .s200_.6           0.227    0.035    6.412    0.000    0.227    0.509
##    .s200_.7           0.160    0.030    5.299    0.000    0.160    0.439
##  .s200_af.4 ~~                                                          
##    .s200_.6           0.261    0.035    7.453    0.000    0.261    0.530
##    .s200_.7           0.190    0.032    5.891    0.000    0.190    0.471
##  .s200_af.6 ~~                                                          
##    .s200_.7           0.215    0.033    6.534    0.000    0.215    0.557
##   dojc2 ~~                                                              
##     dojc3            -0.093    0.016   -5.709    0.000   -0.562   -0.562
##     dojc4             0.023    0.015    1.568    0.117    0.150    0.150
##   dojc3 ~~                                                              
##     dojc4            -0.095    0.018   -5.196    0.000   -0.572   -0.572
##   djc2 ~~                                                               
##     dojc2   (cov1)    0.010    0.003    3.239    0.001    0.152    0.152
##   djc3 ~~                                                               
##     dojc3   (cov1)    0.010    0.003    3.239    0.001    0.169    0.169
##   djc4 ~~                                                               
##     dojc4   (cov1)    0.010    0.003    3.239    0.001    0.133    0.133
##   JC_W1 ~~                                                              
##     c137.6           -0.007    0.009   -0.829    0.407   -0.028   -0.064
##   OJC_W1 ~~                                                             
##     c137.6            0.014    0.014    0.983    0.325    0.032    0.071
##   SRH_W1 ~~                                                             
##     c137.6           -0.112    0.022   -5.108    0.000   -0.167   -0.374
##   djc2 ~~                                                               
##     c137.6           -0.005    0.007   -0.807    0.420   -0.033   -0.073
##   djc3 ~~                                                               
##     c137.6           -0.010    0.006   -1.665    0.096   -0.071   -0.159
##   djc4 ~~                                                               
##     c137.6            0.009    0.008    1.043    0.297    0.046    0.103
##   dojc2 ~~                                                              
##     c137.6            0.009    0.013    0.688    0.491    0.023    0.052
##   dojc3 ~~                                                              
##     c137.6           -0.027    0.014   -1.975    0.048   -0.064   -0.142
##   dojc4 ~~                                                              
##     c137.6            0.014    0.014    0.939    0.348    0.035    0.077
##   JC_W1 ~~                                                              
##     djc2             -0.008    0.004   -1.838    0.066   -0.194   -0.194
##     djc3             -0.003    0.004   -0.734    0.463   -0.088   -0.088
##     djc4              0.002    0.005    0.355    0.723    0.040    0.040
##     OJC_W1            0.031    0.009    3.626    0.000    0.282    0.282
##     dojc2             0.010    0.007    1.357    0.175    0.099    0.099
##     dojc3            -0.015    0.009   -1.723    0.085   -0.138   -0.138
##     dojc4             0.008    0.009    0.886    0.375    0.082    0.082
##     SRH_W1            0.024    0.012    2.008    0.045    0.138    0.138
##   djc2 ~~                                                               
##     OJC_W1           -0.006    0.006   -1.043    0.297   -0.085   -0.085
##     dojc3             0.013    0.006    2.137    0.033    0.185    0.185
##     dojc4            -0.013    0.007   -1.895    0.058   -0.205   -0.205
##     SRH_W1           -0.001    0.009   -0.123    0.902   -0.011   -0.011
##   djc3 ~~                                                               
##     OJC_W1            0.007    0.007    0.990    0.322    0.111    0.111
##     dojc2            -0.010    0.006   -1.719    0.086   -0.186   -0.186
##     dojc4             0.001    0.006    0.224    0.823    0.024    0.024
##     SRH_W1            0.019    0.009    1.992    0.046    0.203    0.203
##   djc4 ~~                                                               
##     OJC_W1           -0.010    0.009   -1.155    0.248   -0.126   -0.126
##     dojc2             0.008    0.008    0.940    0.347    0.105    0.105
##     dojc3            -0.012    0.007   -1.685    0.092   -0.149   -0.149
##     SRH_W1           -0.026    0.012   -2.082    0.037   -0.206   -0.206
##   OJC_W1 ~~                                                             
##     dojc2            -0.057    0.014   -4.194    0.000   -0.341   -0.341
##     dojc3             0.015    0.014    1.083    0.279    0.083    0.083
##     dojc4             0.004    0.015    0.238    0.812    0.021    0.021
##     SRH_W1            0.082    0.019    4.262    0.000    0.288    0.288
##   dojc2 ~~                                                              
##     SRH_W1            0.010    0.018    0.522    0.602    0.037    0.037
##   dojc3 ~~                                                              
##     SRH_W1            0.012    0.020    0.587    0.557    0.043    0.043
##   dojc4 ~~                                                              
##     SRH_W1           -0.010    0.022   -0.440    0.660   -0.037   -0.037
## 
## Intercepts:
##                    Estimate  Std.Err  z-value  P(>|z|)   Std.lv  Std.all
##    .s        (jc6)   -0.172    0.337   -0.509    0.611   -0.172   -0.273
##    .s        (jc6)   -0.172    0.337   -0.509    0.611   -0.172   -0.263
##    .s        (jc6)   -0.172    0.337   -0.509    0.611   -0.172   -0.253
##    .s        (jc6)   -0.172    0.337   -0.509    0.611   -0.172   -0.239
##    .s        (jc4)   -3.271    0.478   -6.837    0.000   -3.271   -3.902
##    .s        (jc4)   -3.271    0.478   -6.837    0.000   -3.271   -3.853
##    .s        (jc4)   -3.271    0.478   -6.837    0.000   -3.271   -3.787
##    .s        (jc4)   -3.271    0.478   -6.837    0.000   -3.271   -4.044
##    .s        (jc5)   -2.496    0.414   -6.023    0.000   -2.496   -4.091
##    .s        (jc5)   -2.496    0.414   -6.023    0.000   -2.496   -3.861
##    .s        (jc5)   -2.496    0.414   -6.023    0.000   -2.496   -3.839
##    .s        (jc5)   -2.496    0.414   -6.023    0.000   -2.496   -3.810
##     J                 2.832    0.025  113.965    0.000   11.041   11.041
##    .J                 0.000                               0.000    0.000
##    .J                 0.000                               0.000    0.000
##    .J                 0.000                               0.000    0.000
##    .s                 0.000                               0.000    0.000
##    .s                 0.000                               0.000    0.000
##    .s                 0.000                               0.000    0.000
##    .s                 0.000                               0.000    0.000
##    .s       (ojc6)   -0.133    0.166   -0.802    0.423   -0.133   -0.171
##    .s       (ojc6)   -0.133    0.166   -0.802    0.423   -0.133   -0.169
##    .s       (ojc6)   -0.133    0.166   -0.802    0.423   -0.133   -0.166
##    .s       (ojc6)   -0.133    0.166   -0.802    0.423   -0.133   -0.162
##    .s       (ojc7)   -1.452    0.235   -6.175    0.000   -1.452   -1.883
##    .s       (ojc7)   -1.452    0.235   -6.175    0.000   -1.452   -1.819
##    .s       (ojc7)   -1.452    0.235   -6.175    0.000   -1.452   -1.836
##    .s       (ojc7)   -1.452    0.235   -6.175    0.000   -1.452   -1.784
##    .s       (ojc8)   -2.012    0.257   -7.815    0.000   -2.012   -2.309
##    .s       (ojc8)   -2.012    0.257   -7.815    0.000   -2.012   -2.370
##    .s       (ojc8)   -2.012    0.257   -7.815    0.000   -2.012   -2.476
##    .s       (ojc8)   -2.012    0.257   -7.815    0.000   -2.012   -2.302
##    .s       (ojc9)   -1.481    0.244   -6.075    0.000   -1.481   -2.017
##    .s       (ojc9)   -1.481    0.244   -6.075    0.000   -1.481   -1.857
##    .s       (ojc9)   -1.481    0.244   -6.075    0.000   -1.481   -1.841
##    .s       (ojc9)   -1.481    0.244   -6.075    0.000   -1.481   -1.790
##    .s       (o10_)   -1.001    0.244   -4.104    0.000   -1.001   -1.213
##    .s       (o10_)   -1.001    0.244   -4.104    0.000   -1.001   -1.098
##    .s       (o10_)   -1.001    0.244   -4.104    0.000   -1.001   -1.104
##    .s       (o10_)   -1.001    0.244   -4.104    0.000   -1.001   -1.203
##     O                 3.967    0.033  119.744    0.000    9.310    9.310
##    .O                 0.000                               0.000    0.000
##    .O                 0.000                               0.000    0.000
##    .O                 0.000                               0.000    0.000
##    .s                 0.000                               0.000    0.000
##    .s                 0.000                               0.000    0.000
##    .s                 0.000                               0.000    0.000
##    .s                 0.000                               0.000    0.000
##     d       (gm_2)   -0.046    0.010   -4.726    0.000   -0.286   -0.286
##     d       (gm_3)    0.004    0.009    0.432    0.666    0.029    0.029
##     d  (gamma_jc4)   -0.061    0.012   -5.019    0.000   -0.329   -0.329
##     d                -0.075    0.025   -2.979    0.003   -0.189   -0.189
##     d                -0.096    0.026   -3.685    0.000   -0.227   -0.227
##     d (gamma_ojc4)    0.037    0.021    1.787    0.074    0.094    0.094
##    .s                 0.000                               0.000    0.000
##    .s                 0.000                               0.000    0.000
##    .s                 0.000                               0.000    0.000
##    .s                 0.000                               0.000    0.000
##     S                 3.551    0.041   86.815    0.000    5.324    5.324
##    .S                 0.259    0.202    1.283    0.199    0.384    0.384
##    .S                 0.260    0.187    1.394    0.163    0.392    0.392
##    .S                 0.109    0.220    0.494    0.622    0.159    0.159
##     c                 0.273    0.028    9.710    0.000    0.273    0.612
## 
## Variances:
##                    Estimate  Std.Err  z-value  P(>|z|)   Std.lv  Std.all
##     JC_W1             0.066    0.012    5.579    0.000    1.000    1.000
##    .JC_W2             0.000                               0.000    0.000
##    .JC_W3             0.000                               0.000    0.000
##    .JC_W4             0.000                               0.000    0.000
##     djc2              0.026    0.006    4.523    0.000    1.000    1.000
##     djc3              0.019    0.005    3.992    0.000    1.000    1.000
##     djc4              0.035    0.008    4.218    0.000    1.000    1.000
##     OJC_W1            0.182    0.022    8.078    0.000    1.000    1.000
##    .OJC_W2            0.000                               0.000    0.000
##    .OJC_W3            0.000                               0.000    0.000
##    .OJC_W4            0.000                               0.000    0.000
##     dojc2             0.156    0.021    7.572    0.000    1.000    1.000
##     dojc3             0.177    0.024    7.355    0.000    1.000    1.000
##     dojc4             0.155    0.024    6.479    0.000    1.000    1.000
##    .s38.3r  (rs_s)    0.154    0.013   12.236    0.000    0.154    0.257
##    .s38.4r  (rs_s)    0.154    0.013   12.236    0.000    0.154    0.253
##    .s38.6   (rs_s)    0.154    0.013   12.236    0.000    0.154    0.259
##    .s38.7   (rs_s)    0.154    0.013   12.236    0.000    0.154    0.248
##    .s21_r.3           0.438    0.037   11.863    0.000    0.438    0.869
##    .s21_s.3           0.271    0.025   10.721    0.000    0.271    0.688
##    .s21_c.3           0.442    0.043   10.299    0.000    0.442    0.629
##    .s21_s.3           0.167    0.021    7.789    0.000    0.167    0.449
##    .s21_r.4           0.405    0.030   13.496    0.000    0.405    0.842
##    .s21_s.4           0.283    0.023   12.114    0.000    0.283    0.665
##    .s21_c.4           0.419    0.038   11.156    0.000    0.419    0.581
##    .s21_s.4           0.181    0.021    8.550    0.000    0.181    0.433
##    .s21_r.6           0.377    0.034   10.963    0.000    0.377    0.835
##    .s21_s.6           0.321    0.031   10.254    0.000    0.321    0.697
##    .s21_c.6           0.451    0.048    9.313    0.000    0.451    0.604
##    .s21_s.6           0.191    0.027    7.158    0.000    0.191    0.452
##    .s21_r.7           0.444    0.044   10.141    0.000    0.444    0.848
##    .s21_s.7           0.368    0.038    9.674    0.000    0.368    0.712
##    .s21_c.7           0.339    0.045    7.465    0.000    0.339    0.519
##    .s21_s.7           0.182    0.028    6.534    0.000    0.182    0.424
##    .s200_.3           0.495    0.041   12.081    0.000    0.495    0.732
##    .s200_.3           0.423    0.035   12.190    0.000    0.423    0.700
##    .s200_.3           0.264    0.028    9.437    0.000    0.264    0.444
##    .s200_.3           0.405    0.039   10.264    0.000    0.405    0.533
##    .s200_.3           0.235    0.025    9.349    0.000    0.235    0.436
##    .s200_.3           0.421    0.038   11.009    0.000    0.421    0.618
##    .s200_.4           0.601    0.043   14.019    0.000    0.601    0.730
##    .s200_.4           0.398    0.029   13.513    0.000    0.398    0.642
##    .s200_.4           0.232    0.023   10.261    0.000    0.232    0.363
##    .s200_.4           0.286    0.026   10.944    0.000    0.286    0.397
##    .s200_.4           0.263    0.024   10.937    0.000    0.263    0.414
##    .s200_.4           0.512    0.041   12.568    0.000    0.512    0.616
##    .s200_.6           0.562    0.049   11.468    0.000    0.562    0.698
##    .s200_.6           0.403    0.035   11.420    0.000    0.403    0.625
##    .s200_.6           0.182    0.023    7.912    0.000    0.182    0.292
##    .s200_.6           0.186    0.023    8.025    0.000    0.186    0.282
##    .s200_.6           0.240    0.027    8.837    0.000    0.240    0.371
##    .s200_.6           0.473    0.046   10.202    0.000    0.473    0.576
##    .s200_.7           0.481    0.045   10.718    0.000    0.481    0.647
##    .s200_.7           0.411    0.040   10.275    0.000    0.411    0.612
##    .s200_.7           0.185    0.026    7.196    0.000    0.185    0.279
##    .s200_.7           0.252    0.031    8.038    0.000    0.252    0.330
##    .s200_.7           0.246    0.030    8.120    0.000    0.246    0.359
##    .s200_.7           0.316    0.036    8.824    0.000    0.316    0.457
##     c137.6            0.200    0.018   10.846    0.000    0.200    1.000
##     SRH_W1            0.445    0.044   10.143    0.000    1.000    1.000
##    .SRH_W2            0.021    0.026    0.794    0.427    0.046    0.046
##    .SRH_W3            0.027    0.022    1.207    0.227    0.061    0.061
##    .SRH_W4            0.080    0.033    2.406    0.016    0.170    0.170
## 
## 
## Group 3 [HO exp]:
## 
## Latent Variables:
##                    Estimate  Std.Err  z-value  P(>|z|)   Std.lv  Std.all
##   JC_W1 =~                                                              
##     s21_r.3           1.000                               0.278    0.422
##     s21_s.3  (jc1)    1.369    0.118   11.648    0.000    0.380    0.614
##     s21_c.3  (jc2)    1.992    0.167   11.921    0.000    0.553    0.620
##     s21_s.3  (jc3)    1.765    0.145   12.186    0.000    0.490    0.746
##   JC_W2 =~                                                              
##     s21_r.4           1.000                               0.270    0.417
##     s21_s.4  (jc1)    1.369    0.118   11.648    0.000    0.369    0.607
##     s21_c.4  (jc2)    1.992    0.167   11.921    0.000    0.538    0.670
##     s21_s.4  (jc3)    1.765    0.145   12.186    0.000    0.476    0.721
##   JC_W3 =~                                                              
##     s21_r.6           1.000                               0.291    0.425
##     s21_s.6  (jc1)    1.369    0.118   11.648    0.000    0.399    0.607
##     s21_c.6  (jc2)    1.992    0.167   11.921    0.000    0.581    0.642
##     s21_s.6  (jc3)    1.765    0.145   12.186    0.000    0.514    0.762
##   JC_W4 =~                                                              
##     s21_r.7           1.000                               0.228    0.345
##     s21_s.7  (jc1)    1.369    0.118   11.648    0.000    0.313    0.535
##     s21_c.7  (jc2)    1.992    0.167   11.921    0.000    0.455    0.550
##     s21_s.7  (jc3)    1.765    0.145   12.186    0.000    0.403    0.656
##   djc2 =~                                                               
##     JC_W2             1.000                               0.582    0.582
##   djc3 =~                                                               
##     JC_W3             1.000                               0.429    0.429
##   djc4 =~                                                               
##     JC_W4             1.000                               0.809    0.809
##   OJC_W1 =~                                                             
##     s200_.3           1.000                               0.477    0.498
##     s200_.3 (ojc1)    0.998    0.042   23.534    0.000    0.476    0.551
##     s200_.3 (ojc2)    1.350    0.060   22.478    0.000    0.643    0.778
##     s200_.3 (ojc3)    1.397    0.066   21.235    0.000    0.666    0.762
##     s200_.3 (ojc4)    1.294    0.062   20.779    0.000    0.617    0.777
##     s200_.3 (ojc5)    1.198    0.062   19.281    0.000    0.571    0.670
##   OJC_W2 =~                                                             
##     s200_.4           1.000                               0.499    0.550
##     s200_.4 (ojc1)    0.998    0.042   23.534    0.000    0.498    0.585
##     s200_.4 (ojc2)    1.350    0.060   22.478    0.000    0.674    0.825
##     s200_.4 (ojc3)    1.397    0.066   21.235    0.000    0.697    0.831
##     s200_.4 (ojc4)    1.294    0.062   20.779    0.000    0.646    0.779
##     s200_.4 (ojc5)    1.198    0.062   19.281    0.000    0.598    0.621
##   OJC_W3 =~                                                             
##     s200_.6           1.000                               0.481    0.592
##     s200_.6 (ojc1)    0.998    0.042   23.534    0.000    0.480    0.610
##     s200_.6 (ojc2)    1.350    0.060   22.478    0.000    0.650    0.854
##     s200_.6 (ojc3)    1.397    0.066   21.235    0.000    0.673    0.816
##     s200_.6 (ojc4)    1.294    0.062   20.779    0.000    0.623    0.802
##     s200_.6 (ojc5)    1.198    0.062   19.281    0.000    0.577    0.700
##   OJC_W4 =~                                                             
##     s200_.7           1.000                               0.479    0.514
##     s200_.7 (ojc1)    0.998    0.042   23.534    0.000    0.478    0.641
##     s200_.7 (ojc2)    1.350    0.060   22.478    0.000    0.647    0.837
##     s200_.7 (ojc3)    1.397    0.066   21.235    0.000    0.670    0.844
##     s200_.7 (ojc4)    1.294    0.062   20.779    0.000    0.620    0.884
##     s200_.7 (ojc5)    1.198    0.062   19.281    0.000    0.574    0.709
##   dojc2 =~                                                              
##     OJC_W2            1.000                               0.798    0.798
##   dojc3 =~                                                              
##     OJC_W3            1.000                               0.820    0.820
##   dojc4 =~                                                              
##     OJC_W4            1.000                               0.582    0.582
##   SRH_W1 =~                                                             
##     s38.3r            1.000                               0.677    0.865
##   SRH_W2 =~                                                             
##     s38.4r            1.000                               0.674    0.864
##   SRH_W3 =~                                                             
##     s38.6             1.000                               0.651    0.856
##   SRH_W4 =~                                                             
##     s38.7             1.000                               0.639    0.852
## 
## Regressions:
##                    Estimate  Std.Err  z-value  P(>|z|)   Std.lv  Std.all
##   JC_W2 ~                                                               
##     JC_W1             1.000                               1.029    1.029
##   JC_W3 ~                                                               
##     JC_W2             1.000                               0.926    0.926
##   JC_W4 ~                                                               
##     JC_W3             1.000                               1.277    1.277
##   OJC_W2 ~                                                              
##     OJC_W1            1.000                               0.955    0.955
##   OJC_W3 ~                                                              
##     OJC_W2            1.000                               1.037    1.037
##   OJC_W4 ~                                                              
##     OJC_W3            1.000                               1.005    1.005
##   SRH_W2 ~                                                              
##     SRH_W1  (ar1_)    0.963    0.053   18.044    0.000    0.968    0.968
##   SRH_W3 ~                                                              
##     SRH_W2  (ar2_)    0.925    0.049   18.977    0.000    0.958    0.958
##   SRH_W4 ~                                                              
##     SRH_W3  (ar3_)    0.956    0.058   16.468    0.000    0.974    0.974
##   SRH_W2 ~                                                              
##     djc2    (jcs1)    0.424    0.204    2.081    0.037    0.099    0.099
##     dojc2   (ojc1)    0.107    0.062    1.728    0.084    0.063    0.063
##     c137.6  (cvd1)   -0.056    0.070   -0.795    0.427   -0.083   -0.036
##   SRH_W3 ~                                                              
##     djc3    (jcs2)    0.692    0.284    2.431    0.015    0.133    0.133
##     dojc3   (ojc2)    0.100    0.064    1.561    0.119    0.061    0.061
##     c137.6  (cvd2)    0.029    0.066    0.431    0.666    0.044    0.019
##   SRH_W4 ~                                                              
##     djc4    (jcs3)   -0.101    0.237   -0.428    0.669   -0.029   -0.029
##     dojc4   (ojc3)    0.239    0.093    2.568    0.010    0.104    0.104
##     c137.6  (cvd3)    0.113    0.077    1.467    0.142    0.177    0.077
## 
## Covariances:
##                    Estimate  Std.Err  z-value  P(>|z|)   Std.lv  Std.all
##  .s21_rd.3 ~~                                                           
##    .s21_r.4           0.185    0.035    5.289    0.000    0.185    0.526
##    .s21_r.6           0.177    0.039    4.490    0.000    0.177    0.477
##    .s21_r.7           0.199    0.044    4.515    0.000    0.199    0.536
##  .s21_rd.4 ~~                                                           
##    .s21_r.6           0.193    0.037    5.277    0.000    0.193    0.530
##    .s21_r.7           0.158    0.039    4.095    0.000    0.158    0.431
##  .s21_rd.6 ~~                                                           
##    .s21_r.7           0.237    0.045    5.312    0.000    0.237    0.614
##  .s21_str.3 ~~                                                          
##    .s21_s.4           0.173    0.027    6.292    0.000    0.173    0.730
##    .s21_s.6           0.192    0.032    5.939    0.000    0.192    0.750
##    .s21_s.7           0.184    0.031    5.976    0.000    0.184    0.764
##  .s21_str.4 ~~                                                          
##    .s21_s.6           0.166    0.029    5.699    0.000    0.166    0.657
##    .s21_s.7           0.170    0.028    6.114    0.000    0.170    0.709
##  .s21_str.6 ~~                                                          
##    .s21_s.7           0.193    0.034    5.754    0.000    0.193    0.748
##  .s21_cd.3 ~~                                                           
##    .s21_c.4           0.231    0.048    4.757    0.000    0.231    0.552
##    .s21_c.6           0.227    0.056    4.073    0.000    0.227    0.468
##    .s21_c.7           0.248    0.058    4.241    0.000    0.248    0.512
##  .s21_cd.4 ~~                                                           
##    .s21_c.6           0.224    0.050    4.451    0.000    0.224    0.543
##    .s21_c.7           0.231    0.052    4.392    0.000    0.231    0.559
##  .s21_cd.6 ~~                                                           
##    .s21_c.7           0.362    0.065    5.577    0.000    0.362    0.757
##  .s21_soz.3 ~~                                                          
##    .s21_s.4           0.122    0.030    4.077    0.000    0.122    0.608
##    .s21_s.6           0.113    0.030    3.745    0.000    0.113    0.589
##    .s21_s.7           0.080    0.032    2.524    0.012    0.080    0.395
##  .s21_soz.4 ~~                                                          
##    .s21_s.6           0.111    0.028    3.917    0.000    0.111    0.555
##    .s21_s.7           0.075    0.031    2.403    0.016    0.075    0.352
##  .s21_soz.6 ~~                                                          
##    .s21_s.7           0.127    0.033    3.780    0.000    0.127    0.625
##   djc2 ~~                                                               
##     djc3             -0.004    0.005   -0.902    0.367   -0.215   -0.215
##     djc4             -0.013    0.006   -2.351    0.019   -0.459   -0.459
##   djc3 ~~                                                               
##     djc4             -0.013    0.006   -2.267    0.023   -0.568   -0.568
##  .s200_de.3 ~~                                                          
##    .s200_.3           0.174    0.051    3.389    0.001    0.174    0.290
##  .s200_de.4 ~~                                                          
##    .s200_.4           0.191    0.039    4.857    0.000    0.191    0.365
##  .s200_de.6 ~~                                                          
##    .s200_.6           0.108    0.039    2.739    0.006    0.108    0.263
##  .s200_de.7 ~~                                                          
##    .s200_.7           0.169    0.055    3.052    0.002    0.169    0.369
##  .s200_de.3 ~~                                                          
##    .s200_.4           0.310    0.055    5.681    0.000    0.310    0.492
##    .s200_.6           0.269    0.055    4.900    0.000    0.269    0.495
##    .s200_.7           0.316    0.079    3.997    0.000    0.316    0.474
##  .s200_de.4 ~~                                                          
##    .s200_.6           0.223    0.046    4.837    0.000    0.223    0.449
##    .s200_.7           0.263    0.066    3.992    0.000    0.263    0.433
##  .s200_de.6 ~~                                                          
##    .s200_.7           0.254    0.060    4.215    0.000    0.254    0.484
##  .s200_re.3 ~~                                                          
##    .s200_.4           0.052    0.039    1.323    0.186    0.052    0.104
##    .s200_.6           0.142    0.048    2.952    0.003    0.142    0.317
##    .s200_.7           0.084    0.042    1.988    0.047    0.084    0.205
##  .s200_re.4 ~~                                                          
##    .s200_.6           0.103    0.038    2.705    0.007    0.103    0.238
##    .s200_.7           0.063    0.038    1.686    0.092    0.063    0.161
##  .s200_re.6 ~~                                                          
##    .s200_.7           0.100    0.044    2.281    0.023    0.100    0.281
##  .s200_au.3 ~~                                                          
##    .s200_.4           0.145    0.029    4.940    0.000    0.145    0.604
##    .s200_.6           0.066    0.029    2.303    0.021    0.066    0.321
##    .s200_.7           0.085    0.032    2.683    0.007    0.085    0.386
##  .s200_au.4 ~~                                                          
##    .s200_.6           0.078    0.024    3.312    0.001    0.078    0.429
##    .s200_.7           0.099    0.027    3.694    0.000    0.099    0.509
##  .s200_au.6 ~~                                                          
##    .s200_.7           0.076    0.025    2.995    0.003    0.076    0.456
##  .s200_ma.3 ~~                                                          
##    .s200_.4           0.056    0.032    1.765    0.078    0.056    0.211
##    .s200_.6           0.070    0.038    1.837    0.066    0.070    0.260
##    .s200_.7           0.038    0.041    0.930    0.352    0.038    0.158
##  .s200_ma.4 ~~                                                          
##    .s200_.6           0.054    0.026    2.064    0.039    0.054    0.241
##    .s200_.7           0.052    0.027    1.931    0.053    0.052    0.261
##  .s200_ma.6 ~~                                                          
##    .s200_.7           0.079    0.029    2.664    0.008    0.079    0.388
##  .s200_me.3 ~~                                                          
##    .s200_.4           0.043    0.029    1.498    0.134    0.043    0.167
##    .s200_.6           0.097    0.033    2.949    0.003    0.097    0.419
##    .s200_.7           0.074    0.025    2.928    0.003    0.074    0.453
##  .s200_me.4 ~~                                                          
##    .s200_.6           0.076    0.027    2.768    0.006    0.076    0.314
##    .s200_.7           0.056    0.025    2.267    0.023    0.056    0.327
##  .s200_me.6 ~~                                                          
##    .s200_.7           0.030    0.024    1.207    0.228    0.030    0.194
##  .s200_af.3 ~~                                                          
##    .s200_.4           0.122    0.044    2.775    0.006    0.122    0.256
##    .s200_.6           0.108    0.045    2.431    0.015    0.108    0.291
##    .s200_.7           0.155    0.043    3.635    0.000    0.155    0.429
##  .s200_af.4 ~~                                                          
##    .s200_.6           0.142    0.044    3.210    0.001    0.142    0.321
##    .s200_.7           0.171    0.049    3.499    0.000    0.171    0.397
##  .s200_af.6 ~~                                                          
##    .s200_.7           0.173    0.043    4.069    0.000    0.173    0.516
##   dojc2 ~~                                                              
##     dojc3            -0.091    0.023   -3.964    0.000   -0.580   -0.580
##     dojc4             0.005    0.017    0.278    0.781    0.041    0.041
##   dojc3 ~~                                                              
##     dojc4            -0.026    0.016   -1.576    0.115   -0.234   -0.234
##   djc2 ~~                                                               
##     dojc2   (cov1)    0.010    0.003    3.239    0.001    0.156    0.156
##   djc3 ~~                                                               
##     dojc3   (cov1)    0.010    0.003    3.239    0.001    0.198    0.198
##   djc4 ~~                                                               
##     dojc4   (cov1)    0.010    0.003    3.239    0.001    0.189    0.189
##   JC_W1 ~~                                                              
##     c137.6           -0.021    0.014   -1.522    0.128   -0.075   -0.172
##   OJC_W1 ~~                                                             
##     c137.6            0.026    0.021    1.264    0.206    0.055    0.127
##   SRH_W1 ~~                                                             
##     c137.6           -0.073    0.031   -2.313    0.021   -0.107   -0.246
##   djc2 ~~                                                               
##     c137.6            0.012    0.009    1.325    0.185    0.079    0.181
##   djc3 ~~                                                               
##     c137.6           -0.012    0.008   -1.501    0.133   -0.094   -0.216
##   djc4 ~~                                                               
##     c137.6           -0.002    0.010   -0.165    0.869   -0.009   -0.020
##   dojc2 ~~                                                              
##     c137.6           -0.045    0.021   -2.203    0.028   -0.114   -0.260
##   dojc3 ~~                                                              
##     c137.6            0.000    0.018    0.011    0.991    0.001    0.001
##   dojc4 ~~                                                              
##     c137.6            0.005    0.015    0.311    0.756    0.016    0.038
##   JC_W1 ~~                                                              
##     djc2             -0.015    0.007   -2.037    0.042   -0.333   -0.333
##     djc3              0.002    0.007    0.380    0.704    0.071    0.071
##     djc4             -0.007    0.008   -0.855    0.393   -0.137   -0.137
##     OJC_W1            0.075    0.017    4.488    0.000    0.565    0.565
##     dojc2            -0.008    0.012   -0.673    0.501   -0.073   -0.073
##     dojc3             0.003    0.013    0.248    0.804    0.030    0.030
##     dojc4            -0.006    0.013   -0.479    0.632   -0.078   -0.078
##     SRH_W1            0.058    0.020    2.883    0.004    0.307    0.307
##   djc2 ~~                                                               
##     OJC_W1           -0.017    0.009   -1.816    0.069   -0.221   -0.221
##     dojc3            -0.007    0.008   -0.855    0.393   -0.115   -0.115
##     dojc4             0.010    0.008    1.223    0.221    0.224    0.224
##     SRH_W1           -0.018    0.014   -1.234    0.217   -0.167   -0.167
##   djc3 ~~                                                               
##     OJC_W1            0.001    0.010    0.061    0.951    0.010    0.010
##     dojc2             0.021    0.008    2.495    0.013    0.419    0.419
##     dojc4            -0.012    0.007   -1.861    0.063   -0.351   -0.351
##     SRH_W1            0.019    0.014    1.422    0.155    0.227    0.227
##   djc4 ~~                                                               
##     OJC_W1            0.000    0.011    0.032    0.975    0.004    0.004
##     dojc2            -0.033    0.011   -2.975    0.003   -0.451   -0.451
##     dojc3             0.010    0.009    1.052    0.293    0.131    0.131
##     SRH_W1            0.000    0.017    0.006    0.995    0.001    0.001
##   OJC_W1 ~~                                                             
##     dojc2            -0.068    0.023   -3.010    0.003   -0.360   -0.360
##     dojc3             0.005    0.021    0.216    0.829    0.024    0.024
##     dojc4            -0.019    0.018   -1.041    0.298   -0.142   -0.142
##     SRH_W1            0.111    0.033    3.350    0.001    0.344    0.344
##   dojc2 ~~                                                              
##     SRH_W1            0.039    0.029    1.319    0.187    0.144    0.144
##   dojc3 ~~                                                              
##     SRH_W1           -0.003    0.029   -0.100    0.920   -0.011   -0.011
##   dojc4 ~~                                                              
##     SRH_W1           -0.051    0.028   -1.828    0.067   -0.272   -0.272
## 
## Intercepts:
##                    Estimate  Std.Err  z-value  P(>|z|)   Std.lv  Std.all
##    .s        (jc6)   -0.172    0.337   -0.509    0.611   -0.172   -0.277
##    .s        (jc6)   -0.172    0.337   -0.509    0.611   -0.172   -0.282
##    .s        (jc6)   -0.172    0.337   -0.509    0.611   -0.172   -0.261
##    .s        (jc6)   -0.172    0.337   -0.509    0.611   -0.172   -0.293
##    .s        (jc4)   -3.271    0.478   -6.837    0.000   -3.271   -3.663
##    .s        (jc4)   -3.271    0.478   -6.837    0.000   -3.271   -4.076
##    .s        (jc4)   -3.271    0.478   -6.837    0.000   -3.271   -3.619
##    .s        (jc4)   -3.271    0.478   -6.837    0.000   -3.271   -3.952
##    .s        (jc5)   -2.496    0.414   -6.023    0.000   -2.496   -3.797
##    .s        (jc5)   -2.496    0.414   -6.023    0.000   -2.496   -3.777
##    .s        (jc5)   -2.496    0.414   -6.023    0.000   -2.496   -3.697
##    .s        (jc5)   -2.496    0.414   -6.023    0.000   -2.496   -4.063
##     J                 3.019    0.032   94.509    0.000   10.867   10.867
##    .J                 0.000                               0.000    0.000
##    .J                 0.000                               0.000    0.000
##    .J                 0.000                               0.000    0.000
##    .s                 0.000                               0.000    0.000
##    .s                 0.000                               0.000    0.000
##    .s                 0.000                               0.000    0.000
##    .s                 0.000                               0.000    0.000
##    .s       (ojc6)   -0.133    0.166   -0.802    0.423   -0.133   -0.154
##    .s       (ojc6)   -0.133    0.166   -0.802    0.423   -0.133   -0.156
##    .s       (ojc6)   -0.133    0.166   -0.802    0.423   -0.133   -0.169
##    .s       (ojc6)   -0.133    0.166   -0.802    0.423   -0.133   -0.179
##    .s       (ojc7)   -1.452    0.235   -6.175    0.000   -1.452   -1.755
##    .s       (ojc7)   -1.452    0.235   -6.175    0.000   -1.452   -1.779
##    .s       (ojc7)   -1.452    0.235   -6.175    0.000   -1.452   -1.909
##    .s       (ojc7)   -1.452    0.235   -6.175    0.000   -1.452   -1.878
##    .s       (ojc8)   -2.012    0.257   -7.815    0.000   -2.012   -2.302
##    .s       (ojc8)   -2.012    0.257   -7.815    0.000   -2.012   -2.397
##    .s       (ojc8)   -2.012    0.257   -7.815    0.000   -2.012   -2.440
##    .s       (ojc8)   -2.012    0.257   -7.815    0.000   -2.012   -2.537
##    .s       (ojc9)   -1.481    0.244   -6.075    0.000   -1.481   -1.865
##    .s       (ojc9)   -1.481    0.244   -6.075    0.000   -1.481   -1.787
##    .s       (ojc9)   -1.481    0.244   -6.075    0.000   -1.481   -1.906
##    .s       (ojc9)   -1.481    0.244   -6.075    0.000   -1.481   -2.111
##    .s       (o10_)   -0.989    0.246   -4.013    0.000   -0.989   -1.160
##    .s       (o10_)   -0.989    0.246   -4.013    0.000   -0.989   -1.028
##    .s       (o10_)   -0.989    0.246   -4.013    0.000   -0.989   -1.201
##    .s       (o10_)   -0.989    0.246   -4.013    0.000   -0.989   -1.222
##     O                 3.905    0.047   83.757    0.000    8.192    8.192
##    .O                 0.000                               0.000    0.000
##    .O                 0.000                               0.000    0.000
##    .O                 0.000                               0.000    0.000
##    .s                 0.000                               0.000    0.000
##    .s                 0.000                               0.000    0.000
##    .s                 0.000                               0.000    0.000
##    .s                 0.000                               0.000    0.000
##     d       (gm_2)   -0.046    0.010   -4.726    0.000   -0.295   -0.295
##     d       (gm_3)    0.004    0.009    0.432    0.666    0.031    0.031
##     d  (gamma_jc4)   -0.061    0.012   -5.019    0.000   -0.331   -0.331
##     d                 0.047    0.037    1.277    0.202    0.118    0.118
##     d                -0.144    0.036   -3.976    0.000   -0.365   -0.365
##     d (gamma_ojc4)    0.037    0.021    1.787    0.074    0.132    0.132
##    .s                 0.000                               0.000    0.000
##    .s                 0.000                               0.000    0.000
##    .s                 0.000                               0.000    0.000
##    .s                 0.000                               0.000    0.000
##     S                 3.663    0.062   59.405    0.000    5.415    5.415
##    .S                 0.179    0.212    0.844    0.399    0.266    0.266
##    .S                 0.338    0.195    1.733    0.083    0.519    0.519
##    .S                 0.041    0.236    0.173    0.862    0.064    0.064
##     c                 0.278    0.039    7.104    0.000    0.278    0.637
## 
## Variances:
##                    Estimate  Std.Err  z-value  P(>|z|)   Std.lv  Std.all
##     JC_W1             0.077    0.017    4.662    0.000    1.000    1.000
##    .JC_W2             0.000                               0.000    0.000
##    .JC_W3             0.000                               0.000    0.000
##    .JC_W4             0.000                               0.000    0.000
##     djc2              0.025    0.007    3.622    0.000    1.000    1.000
##     djc3              0.016    0.006    2.760    0.006    1.000    1.000
##     djc4              0.034    0.010    3.408    0.001    1.000    1.000
##     OJC_W1            0.227    0.037    6.204    0.000    1.000    1.000
##    .OJC_W2            0.000                               0.000    0.000
##    .OJC_W3            0.000                               0.000    0.000
##    .OJC_W4            0.000                               0.000    0.000
##     dojc2             0.158    0.028    5.679    0.000    1.000    1.000
##     dojc3             0.156    0.029    5.457    0.000    1.000    1.000
##     dojc4             0.078    0.018    4.260    0.000    1.000    1.000
##    .s38.3r  (rs_s)    0.154    0.013   12.236    0.000    0.154    0.252
##    .s38.4r  (rs_s)    0.154    0.013   12.236    0.000    0.154    0.254
##    .s38.6   (rs_s)    0.154    0.013   12.236    0.000    0.154    0.267
##    .s38.7   (rs_s)    0.154    0.013   12.236    0.000    0.154    0.275
##    .s21_r.3           0.357    0.047    7.634    0.000    0.357    0.822
##    .s21_s.3           0.239    0.034    7.108    0.000    0.239    0.623
##    .s21_c.3           0.491    0.070    6.976    0.000    0.491    0.616
##    .s21_s.3           0.192    0.038    5.086    0.000    0.192    0.444
##    .s21_r.4           0.345    0.038    9.076    0.000    0.345    0.826
##    .s21_s.4           0.234    0.030    7.925    0.000    0.234    0.632
##    .s21_c.4           0.355    0.049    7.253    0.000    0.355    0.551
##    .s21_s.4           0.210    0.032    6.493    0.000    0.210    0.481
##    .s21_r.6           0.386    0.051    7.502    0.000    0.386    0.819
##    .s21_s.6           0.273    0.040    6.753    0.000    0.273    0.632
##    .s21_c.6           0.480    0.073    6.580    0.000    0.480    0.587
##    .s21_s.6           0.191    0.035    5.404    0.000    0.191    0.419
##    .s21_r.7           0.387    0.058    6.723    0.000    0.387    0.881
##    .s21_s.7           0.244    0.037    6.646    0.000    0.244    0.714
##    .s21_c.7           0.478    0.077    6.216    0.000    0.478    0.698
##    .s21_s.7           0.215    0.043    5.037    0.000    0.215    0.570
##    .s200_.3           0.691    0.082    8.464    0.000    0.691    0.752
##    .s200_.3           0.518    0.066    7.812    0.000    0.518    0.696
##    .s200_.3           0.270    0.042    6.364    0.000    0.270    0.395
##    .s200_.3           0.320    0.051    6.325    0.000    0.320    0.419
##    .s200_.3           0.250    0.040    6.177    0.000    0.250    0.396
##    .s200_.3           0.400    0.056    7.143    0.000    0.400    0.551
##    .s200_.4           0.576    0.060    9.647    0.000    0.576    0.698
##    .s200_.4           0.478    0.052    9.266    0.000    0.478    0.658
##    .s200_.4           0.212    0.031    6.889    0.000    0.212    0.319
##    .s200_.4           0.218    0.032    6.736    0.000    0.218    0.310
##    .s200_.4           0.270    0.035    7.612    0.000    0.270    0.393
##    .s200_.4           0.568    0.064    8.915    0.000    0.568    0.614
##    .s200_.6           0.429    0.052    8.248    0.000    0.429    0.649
##    .s200_.6           0.390    0.051    7.664    0.000    0.390    0.628
##    .s200_.6           0.156    0.028    5.563    0.000    0.156    0.270
##    .s200_.6           0.227    0.038    5.999    0.000    0.227    0.334
##    .s200_.6           0.215    0.035    6.137    0.000    0.215    0.357
##    .s200_.6           0.346    0.050    6.952    0.000    0.346    0.509
##    .s200_.7           0.641    0.083    7.691    0.000    0.641    0.736
##    .s200_.7           0.327    0.048    6.838    0.000    0.327    0.589
##    .s200_.7           0.179    0.034    5.281    0.000    0.179    0.300
##    .s200_.7           0.180    0.034    5.357    0.000    0.180    0.287
##    .s200_.7           0.108    0.024    4.471    0.000    0.108    0.219
##    .s200_.7           0.325    0.052    6.229    0.000    0.325    0.497
##     c137.6            0.191    0.025    7.762    0.000    0.191    1.000
##     SRH_W1            0.458    0.064    7.176    0.000    1.000    1.000
##    .SRH_W2            0.020    0.034    0.585    0.558    0.044    0.044
##    .SRH_W3            0.007    0.028    0.249    0.804    0.017    0.017
##    .SRH_W4            0.051    0.043    1.186    0.236    0.126    0.126
```

```
# FINAL MODEL: lcsm_parcel_SRH_GC1_equalARc137COVJCOJC_timeequalCOV_equalCSJC123OJC3
```

### Living alone vs. Living with Partner/family

```
## LCSM Job and Off-Job Crafting with self-rated health as outcome ----

### Model estimation ----
model_lcsm_parcel_SRH_GC2_equalARc137W23COVJCOJCW23_timeequalCOV_equalCSJC123OJC123 <- paste(measurement_model_parcelled_jc_ojc_GC2_BASE, fixed_cs_jc_123, fixed_cs_ojc_123, measurement_model_srh_equalAR, model_latent_srh_equalc137W23COVJCOJCW23_timeequalCOV, sep = "")

lcsm_parcel_SRH_GC2_equalARc137W23COVJCOJCW23_timeequalCOV_equalCSJC123OJC123 <- sem(model_lcsm_parcel_SRH_GC2_equalARc137W23COVJCOJCW23_timeequalCOV_equalCSJC123OJC123, data, 
                   estimator = "ML", 
                   missing = "ML",
                   group = "group2_livingsit.4",
                   fixed.x = FALSE); beep(6) # Has to be set to zero, otherwise the missings in the exogenous var c137.6 cause deletion of cases

summary(lcsm_parcel_SRH_GC2_equalARc137W23COVJCOJCW23_timeequalCOV_equalCSJC123OJC123, fit.measures = TRUE, standardized = TRUE, fm.args = list(robust = FALSE))
```

```
## lavaan 0.6.16 ended normally after 491 iterations
## 
##   Estimator                                         ML
##   Optimization method                           NLMINB
##   Number of model parameters                       510
##   Number of equality constraints                   138
## 
##   Number of observations per group:                   
##     Partner/Family                                 540
##     alone                                          213
##   Number of missing patterns per group:               
##     Partner/Family                                  28
##     alone                                           21
## 
## Model Test User Model:
##                                                       
##   Test statistic                              3388.040
##   Degrees of freedom                              1788
##   P-value (Chi-square)                           0.000
##   Test statistic for each group:
##     Partner/Family                            1774.067
##     alone                                     1613.973
## 
## Model Test Baseline Model:
## 
##   Test statistic                             17699.900
##   Degrees of freedom                              1980
##   P-value                                        0.000
## 
## User Model versus Baseline Model:
## 
##   Comparative Fit Index (CFI)                    0.898
##   Tucker-Lewis Index (TLI)                       0.887
## 
## Loglikelihood and Information Criteria:
## 
##   Loglikelihood user model (H0)             -20762.860
##   Loglikelihood unrestricted model (H1)     -19068.840
##                                                       
##   Akaike (AIC)                               42269.720
##   Bayesian (BIC)                             43989.872
##   Sample-size adjusted Bayesian (SABIC)      42808.623
## 
## Root Mean Square Error of Approximation:
## 
##   RMSEA                                          0.049
##   90 Percent confidence interval - lower         0.046
##   90 Percent confidence interval - upper         0.051
##   P-value H_0: RMSEA <= 0.050                    0.792
##   P-value H_0: RMSEA >= 0.080                    0.000
## 
## Standardized Root Mean Square Residual:
## 
##   SRMR                                           0.086
## 
## Parameter Estimates:
## 
##   Standard errors                             Standard
##   Information                                 Observed
##   Observed information based on                Hessian
## 
## 
## Group 1 [Partner/Family]:
## 
## Latent Variables:
##                    Estimate  Std.Err  z-value  P(>|z|)   Std.lv  Std.all
##   JC_W1 =~                                                              
##     s21_r.3           1.000                               0.252    0.374
##     s21_s.3  (jc1)    1.426    0.124   11.494    0.000    0.359    0.575
##     s21_c.3  (jc2)    2.066    0.177   11.664    0.000    0.521    0.606
##     s21_s.3  (jc3)    1.790    0.151   11.862    0.000    0.451    0.702
##   JC_W2 =~                                                              
##     s21_r.4           1.000                               0.280    0.413
##     s21_s.4  (jc1)    1.426    0.124   11.494    0.000    0.399    0.623
##     s21_c.4  (jc2)    2.066    0.177   11.664    0.000    0.578    0.672
##     s21_s.4  (jc3)    1.790    0.151   11.862    0.000    0.501    0.749
##   JC_W3 =~                                                              
##     s21_r.6           1.000                               0.282    0.424
##     s21_s.6  (jc1)    1.426    0.124   11.494    0.000    0.402    0.602
##     s21_c.6  (jc2)    2.066    0.177   11.664    0.000    0.582    0.647
##     s21_s.6  (jc3)    1.790    0.151   11.862    0.000    0.504    0.742
##   JC_W4 =~                                                              
##     s21_r.7           1.000                               0.287    0.407
##     s21_s.7  (jc1)    1.426    0.124   11.494    0.000    0.409    0.586
##     s21_c.7  (jc2)    2.066    0.177   11.664    0.000    0.593    0.690
##     s21_s.7  (jc3)    1.790    0.151   11.862    0.000    0.514    0.774
##   djc2 =~                                                               
##     JC_W2             1.000                               0.546    0.546
##   djc3 =~                                                               
##     JC_W3             1.000                               0.500    0.500
##   djc4 =~                                                               
##     JC_W4             1.000                               0.615    0.615
##   OJC_W1 =~                                                             
##     s200_.3           1.000                               0.449    0.515
##     s200_.3 (ojc1)    0.971    0.041   23.411    0.000    0.436    0.541
##     s200_.3 (ojc2)    1.323    0.058   22.888    0.000    0.594    0.795
##     s200_.3 (ojc3)    1.368    0.064   21.383    0.000    0.614    0.726
##     s200_.3 (ojc4)    1.270    0.060   21.066    0.000    0.570    0.753
##     s200_.3 (ojc5)    1.174    0.060   19.486    0.000    0.527    0.660
##   OJC_W2 =~                                                             
##     s200_.4           1.000                               0.498    0.545
##     s200_.4 (ojc1)    0.971    0.041   23.411    0.000    0.484    0.589
##     s200_.4 (ojc2)    1.323    0.058   22.888    0.000    0.659    0.815
##     s200_.4 (ojc3)    1.368    0.064   21.383    0.000    0.681    0.800
##     s200_.4 (ojc4)    1.270    0.060   21.066    0.000    0.633    0.791
##     s200_.4 (ojc5)    1.174    0.060   19.486    0.000    0.585    0.664
##   OJC_W3 =~                                                             
##     s200_.6           1.000                               0.475    0.564
##     s200_.6 (ojc1)    0.971    0.041   23.411    0.000    0.461    0.596
##     s200_.6 (ojc2)    1.323    0.058   22.888    0.000    0.629    0.853
##     s200_.6 (ojc3)    1.368    0.064   21.383    0.000    0.650    0.818
##     s200_.6 (ojc4)    1.270    0.060   21.066    0.000    0.604    0.795
##     s200_.6 (ojc5)    1.174    0.060   19.486    0.000    0.558    0.683
##   OJC_W4 =~                                                             
##     s200_.7           1.000                               0.459    0.539
##     s200_.7 (ojc1)    0.971    0.041   23.411    0.000    0.446    0.603
##     s200_.7 (ojc2)    1.323    0.058   22.888    0.000    0.608    0.826
##     s200_.7 (ojc3)    1.368    0.064   21.383    0.000    0.628    0.809
##     s200_.7 (ojc4)    1.270    0.060   21.066    0.000    0.583    0.773
##     s200_.7 (ojc5)    1.174    0.060   19.486    0.000    0.539    0.688
##   dojc2 =~                                                              
##     OJC_W2            1.000                               0.807    0.807
##   dojc3 =~                                                              
##     OJC_W3            1.000                               0.843    0.843
##   dojc4 =~                                                              
##     OJC_W4            1.000                               0.691    0.691
##   SRH_W1 =~                                                             
##     s38.3r            1.000                               0.678    0.870
##   SRH_W2 =~                                                             
##     s38.4r            1.000                               0.659    0.864
##   SRH_W3 =~                                                             
##     s38.6             1.000                               0.663    0.865
##   SRH_W4 =~                                                             
##     s38.7             1.000                               0.665    0.866
## 
## Regressions:
##                    Estimate  Std.Err  z-value  P(>|z|)   Std.lv  Std.all
##   JC_W2 ~                                                               
##     JC_W1             1.000                               0.901    0.901
##   JC_W3 ~                                                               
##     JC_W2             1.000                               0.993    0.993
##   JC_W4 ~                                                               
##     JC_W3             1.000                               0.982    0.982
##   OJC_W2 ~                                                              
##     OJC_W1            1.000                               0.901    0.901
##   OJC_W3 ~                                                              
##     OJC_W2            1.000                               1.049    1.049
##   OJC_W4 ~                                                              
##     OJC_W3            1.000                               1.035    1.035
##   SRH_W2 ~                                                              
##     SRH_W1  (ar1_)    0.935    0.049   19.242    0.000    0.962    0.962
##   SRH_W3 ~                                                              
##     SRH_W2  (ar2_)    0.934    0.048   19.443    0.000    0.929    0.929
##   SRH_W4 ~                                                              
##     SRH_W3  (ar3_)    0.966    0.056   17.114    0.000    0.963    0.963
##   SRH_W2 ~                                                              
##     djc2    (jcs1)    0.450    0.206    2.183    0.029    0.104    0.104
##     dojc2   (ojc1)    0.115    0.060    1.912    0.056    0.070    0.070
##     c137.6  (cvd1)   -0.058    0.067   -0.858    0.391   -0.088   -0.040
##   SRH_W3 ~                                                              
##     djc3    (jcs2)    0.428    0.260    1.651    0.099    0.091    0.091
##     dojc3   (ojc2)    0.112    0.060    1.861    0.063    0.068    0.068
##     c137.6  (cvd2)   -0.023    0.065   -0.352    0.725   -0.034   -0.016
##   SRH_W4 ~                                                              
##     djc4    (jcs3)    0.112    0.245    0.457    0.648    0.030    0.030
##     dojc4             0.508    0.132    3.847    0.000    0.242    0.242
##     c137.6            0.011    0.083    0.136    0.892    0.017    0.008
## 
## Covariances:
##                    Estimate  Std.Err  z-value  P(>|z|)   Std.lv  Std.all
##  .s21_rd.3 ~~                                                           
##    .s21_r.4           0.184    0.021    8.556    0.000    0.184    0.477
##    .s21_r.6           0.214    0.024    8.733    0.000    0.214    0.567
##    .s21_r.7           0.217    0.027    8.105    0.000    0.217    0.540
##  .s21_rd.4 ~~                                                           
##    .s21_r.6           0.209    0.022    9.518    0.000    0.209    0.564
##    .s21_r.7           0.185    0.025    7.377    0.000    0.185    0.468
##  .s21_rd.6 ~~                                                           
##    .s21_r.7           0.208    0.027    7.622    0.000    0.208    0.537
##  .s21_str.3 ~~                                                          
##    .s21_s.4           0.155    0.017    9.299    0.000    0.155    0.604
##    .s21_s.6           0.174    0.019    8.923    0.000    0.174    0.637
##    .s21_s.7           0.201    0.021    9.382    0.000    0.201    0.694
##  .s21_str.4 ~~                                                          
##    .s21_s.6           0.170    0.018    9.579    0.000    0.170    0.635
##    .s21_s.7           0.178    0.020    9.057    0.000    0.178    0.628
##  .s21_str.6 ~~                                                          
##    .s21_s.7           0.205    0.023    9.117    0.000    0.205    0.679
##  .s21_cd.3 ~~                                                           
##    .s21_c.4           0.260    0.030    8.604    0.000    0.260    0.599
##    .s21_c.6           0.314    0.035    8.913    0.000    0.314    0.670
##    .s21_c.7           0.245    0.034    7.194    0.000    0.245    0.578
##  .s21_cd.4 ~~                                                           
##    .s21_c.6           0.292    0.032    9.114    0.000    0.292    0.670
##    .s21_c.7           0.241    0.031    7.759    0.000    0.241    0.610
##  .s21_cd.6 ~~                                                           
##    .s21_c.7           0.284    0.036    7.974    0.000    0.284    0.666
##  .s21_soz.3 ~~                                                          
##    .s21_s.4           0.124    0.018    7.035    0.000    0.124    0.612
##    .s21_s.6           0.133    0.020    6.796    0.000    0.133    0.635
##    .s21_s.7           0.099    0.019    5.226    0.000    0.099    0.517
##  .s21_soz.4 ~~                                                          
##    .s21_s.6           0.113    0.018    6.361    0.000    0.113    0.560
##    .s21_s.7           0.095    0.018    5.200    0.000    0.095    0.510
##  .s21_soz.6 ~~                                                          
##    .s21_s.7           0.111    0.020    5.577    0.000    0.111    0.578
##   djc2 ~~                                                               
##     djc3             -0.007    0.003   -2.394    0.017   -0.341   -0.341
##     djc4             -0.001    0.003   -0.294    0.769   -0.036   -0.036
##   djc3 ~~                                                               
##     djc4             -0.015    0.004   -3.696    0.000   -0.616   -0.616
##  .s200_de.3 ~~                                                          
##    .s200_.3           0.120    0.025    4.867    0.000    0.120    0.236
##  .s200_de.4 ~~                                                          
##    .s200_.4           0.169    0.023    7.379    0.000    0.169    0.333
##  .s200_de.6 ~~                                                          
##    .s200_.6           0.180    0.025    7.192    0.000    0.180    0.417
##  .s200_de.7 ~~                                                          
##    .s200_.7           0.142    0.026    5.548    0.000    0.142    0.336
##  .s200_de.3 ~~                                                          
##    .s200_.4           0.252    0.030    8.459    0.000    0.252    0.439
##    .s200_.6           0.199    0.029    6.943    0.000    0.199    0.382
##    .s200_.7           0.240    0.033    7.264    0.000    0.240    0.448
##  .s200_de.4 ~~                                                          
##    .s200_.6           0.187    0.026    7.042    0.000    0.187    0.350
##    .s200_.7           0.224    0.031    7.321    0.000    0.224    0.408
##  .s200_de.6 ~~                                                          
##    .s200_.7           0.222    0.029    7.590    0.000    0.222    0.446
##  .s200_re.3 ~~                                                          
##    .s200_.4           0.123    0.023    5.416    0.000    0.123    0.274
##    .s200_.6           0.140    0.024    5.822    0.000    0.140    0.332
##    .s200_.7           0.151    0.025    6.073    0.000    0.151    0.379
##  .s200_re.4 ~~                                                          
##    .s200_.6           0.098    0.020    4.840    0.000    0.098    0.238
##    .s200_.7           0.107    0.021    5.016    0.000    0.107    0.275
##  .s200_re.6 ~~                                                          
##    .s200_.7           0.104    0.023    4.615    0.000    0.104    0.284
##  .s200_au.3 ~~                                                          
##    .s200_.4           0.073    0.015    4.824    0.000    0.073    0.343
##    .s200_.6           0.031    0.015    2.116    0.034    0.031    0.176
##    .s200_.7           0.026    0.017    1.534    0.125    0.026    0.141
##  .s200_au.4 ~~                                                          
##    .s200_.6           0.050    0.014    3.638    0.000    0.050    0.279
##    .s200_.7           0.053    0.016    3.264    0.001    0.053    0.272
##  .s200_au.6 ~~                                                          
##    .s200_.7           0.047    0.016    3.010    0.003    0.047    0.294
##  .s200_ma.3 ~~                                                          
##    .s200_.4           0.092    0.019    4.714    0.000    0.092    0.309
##    .s200_.6           0.106    0.020    5.287    0.000    0.106    0.396
##    .s200_.7           0.058    0.022    2.639    0.008    0.058    0.218
##  .s200_ma.4 ~~                                                          
##    .s200_.6           0.077    0.016    4.719    0.000    0.077    0.331
##    .s200_.7           0.078    0.019    4.183    0.000    0.078    0.335
##  .s200_ma.6 ~~                                                          
##    .s200_.7           0.077    0.018    4.220    0.000    0.077    0.368
##  .s200_me.3 ~~                                                          
##    .s200_.4           0.070    0.016    4.316    0.000    0.070    0.285
##    .s200_.6           0.057    0.017    3.260    0.001    0.057    0.246
##    .s200_.7           0.092    0.019    4.808    0.000    0.092    0.385
##  .s200_me.4 ~~                                                          
##    .s200_.6           0.055    0.016    3.526    0.000    0.055    0.243
##    .s200_.7           0.066    0.018    3.760    0.000    0.066    0.282
##  .s200_me.6 ~~                                                          
##    .s200_.7           0.082    0.019    4.234    0.000    0.082    0.371
##  .s200_af.3 ~~                                                          
##    .s200_.4           0.137    0.023    6.032    0.000    0.137    0.346
##    .s200_.6           0.120    0.025    4.856    0.000    0.120    0.336
##    .s200_.7           0.121    0.025    4.889    0.000    0.121    0.355
##  .s200_af.4 ~~                                                          
##    .s200_.6           0.128    0.024    5.271    0.000    0.128    0.325
##    .s200_.7           0.152    0.026    5.821    0.000    0.152    0.407
##  .s200_af.6 ~~                                                          
##    .s200_.7           0.178    0.026    6.895    0.000    0.178    0.525
##   dojc2 ~~                                                              
##     dojc3            -0.093    0.015   -6.402    0.000   -0.576   -0.576
##     dojc4            -0.014    0.012   -1.183    0.237   -0.113   -0.113
##   dojc3 ~~                                                              
##     dojc4            -0.046    0.012   -3.846    0.000   -0.366   -0.366
##   djc2 ~~                                                               
##     dojc2   (cov1)    0.008    0.003    2.527    0.011    0.123    0.123
##   djc3 ~~                                                               
##     dojc3   (cov1)    0.008    0.003    2.527    0.011    0.134    0.134
##   djc4 ~~                                                               
##     dojc4   (cov1)    0.008    0.003    2.527    0.011    0.135    0.135
##   JC_W1 ~~                                                              
##     c137.6           -0.011    0.008   -1.382    0.167   -0.042   -0.092
##   OJC_W1 ~~                                                             
##     c137.6            0.024    0.013    1.832    0.067    0.054    0.116
##   SRH_W1 ~~                                                             
##     c137.6           -0.106    0.020   -5.233    0.000   -0.157   -0.340
##   djc2 ~~                                                               
##     c137.6            0.001    0.006    0.199    0.842    0.007    0.016
##   djc3 ~~                                                               
##     c137.6           -0.014    0.005   -2.582    0.010   -0.098   -0.212
##   djc4 ~~                                                               
##     c137.6            0.011    0.007    1.599    0.110    0.063    0.135
##   dojc2 ~~                                                              
##     c137.6           -0.008    0.012   -0.653    0.514   -0.020   -0.043
##   dojc3 ~~                                                              
##     c137.6           -0.021    0.012   -1.782    0.075   -0.052   -0.112
##   dojc4 ~~                                                              
##     c137.6            0.017    0.011    1.547    0.122    0.053    0.115
##   JC_W1 ~~                                                              
##     djc2             -0.004    0.004   -1.216    0.224   -0.112   -0.112
##     djc3             -0.002    0.004   -0.567    0.571   -0.057   -0.057
##     djc4              0.002    0.005    0.483    0.629    0.049    0.049
##     OJC_W1            0.036    0.008    4.443    0.000    0.316    0.316
##     dojc2             0.018    0.007    2.519    0.012    0.173    0.173
##     dojc3            -0.013    0.007   -1.768    0.077   -0.125   -0.125
##     dojc4            -0.003    0.007   -0.461    0.645   -0.041   -0.041
##     SRH_W1            0.032    0.011    3.016    0.003    0.186    0.186
##   djc2 ~~                                                               
##     OJC_W1           -0.006    0.005   -1.226    0.220   -0.088   -0.088
##     dojc3             0.004    0.005    0.763    0.445    0.060    0.060
##     dojc4            -0.002    0.005   -0.365    0.715   -0.038   -0.038
##     SRH_W1           -0.006    0.008   -0.745    0.456   -0.058   -0.058
##   djc3 ~~                                                               
##     OJC_W1            0.007    0.006    1.150    0.250    0.110    0.110
##     dojc2             0.003    0.005    0.531    0.595    0.049    0.049
##     dojc4            -0.010    0.004   -2.217    0.027   -0.222   -0.222
##     SRH_W1            0.028    0.009    3.227    0.001    0.288    0.288
##   djc4 ~~                                                               
##     OJC_W1           -0.012    0.008   -1.578    0.115   -0.155   -0.155
##     dojc2             0.007    0.007    0.918    0.359    0.092    0.092
##     dojc3            -0.010    0.006   -1.577    0.115   -0.135   -0.135
##     SRH_W1           -0.027    0.010   -2.622    0.009   -0.229   -0.229
##   OJC_W1 ~~                                                             
##     dojc2            -0.058    0.013   -4.478    0.000   -0.319   -0.319
##     dojc3             0.001    0.012    0.115    0.908    0.008    0.008
##     dojc4             0.003    0.012    0.250    0.802    0.022    0.022
##     SRH_W1            0.107    0.018    5.790    0.000    0.351    0.351
##   dojc2 ~~                                                              
##     SRH_W1            0.013    0.017    0.769    0.442    0.048    0.048
##   dojc3 ~~                                                              
##     SRH_W1            0.003    0.017    0.188    0.851    0.012    0.012
##   dojc4 ~~                                                              
##     SRH_W1           -0.009    0.017   -0.530    0.596   -0.041   -0.041
## 
## Intercepts:
##                    Estimate  Std.Err  z-value  P(>|z|)   Std.lv  Std.all
##    .s        (jc6)   -0.347    0.358   -0.970    0.332   -0.347   -0.555
##    .s        (jc6)   -0.347    0.358   -0.970    0.332   -0.347   -0.542
##    .s        (jc6)   -0.347    0.358   -0.970    0.332   -0.347   -0.519
##    .s        (jc6)   -0.347    0.358   -0.970    0.332   -0.347   -0.497
##    .s        (jc4)   -3.505    0.510   -6.877    0.000   -3.505   -4.081
##    .s        (jc4)   -3.505    0.510   -6.877    0.000   -3.505   -4.076
##    .s        (jc4)   -3.505    0.510   -6.877    0.000   -3.505   -3.896
##    .s        (jc4)   -3.505    0.510   -6.877    0.000   -3.505   -4.082
##    .s        (jc5)   -2.593    0.434   -5.974    0.000   -2.593   -4.035
##    .s        (jc5)   -2.593    0.434   -5.974    0.000   -2.593   -3.879
##    .s        (jc5)   -2.593    0.434   -5.974    0.000   -2.593   -3.812
##    .s        (jc5)   -2.593    0.434   -5.974    0.000   -2.593   -3.907
##     J                 2.922    0.024  122.903    0.000   11.595   11.595
##    .J                 0.000                               0.000    0.000
##    .J                 0.000                               0.000    0.000
##    .J                 0.000                               0.000    0.000
##    .s                 0.000                               0.000    0.000
##    .s                 0.000                               0.000    0.000
##    .s                 0.000                               0.000    0.000
##    .s                 0.000                               0.000    0.000
##    .s       (ojc6)   -0.011    0.162   -0.068    0.946   -0.011   -0.014
##    .s       (ojc6)   -0.011    0.162   -0.068    0.946   -0.011   -0.013
##    .s       (ojc6)   -0.011    0.162   -0.068    0.946   -0.011   -0.014
##    .s       (ojc6)   -0.011    0.162   -0.068    0.946   -0.011   -0.015
##    .s       (o7_1)   -1.365    0.226   -6.045    0.000   -1.365   -1.827
##    .s       (o7_1)   -1.365    0.226   -6.045    0.000   -1.365   -1.686
##    .s       (o7_1)   -1.365    0.226   -6.045    0.000   -1.365   -1.852
##    .s       (o7_1)   -1.365    0.226   -6.045    0.000   -1.365   -1.856
##    .s       (ojc8)   -1.864    0.249   -7.478    0.000   -1.864   -2.204
##    .s       (ojc8)   -1.864    0.249   -7.478    0.000   -1.864   -2.190
##    .s       (ojc8)   -1.864    0.249   -7.478    0.000   -1.864   -2.345
##    .s       (ojc8)   -1.864    0.249   -7.478    0.000   -1.864   -2.402
##    .s       (ojc9)   -1.355    0.235   -5.762    0.000   -1.355   -1.788
##    .s       (ojc9)   -1.355    0.235   -5.762    0.000   -1.355   -1.693
##    .s       (ojc9)   -1.355    0.235   -5.762    0.000   -1.355   -1.785
##    .s       (ojc9)   -1.355    0.235   -5.762    0.000   -1.355   -1.795
##    .s       (o10_)   -0.782    0.236   -3.321    0.001   -0.782   -0.980
##    .s       (o10_)   -0.782    0.236   -3.321    0.001   -0.782   -0.888
##    .s       (o10_)   -0.782    0.236   -3.321    0.001   -0.782   -0.958
##    .s       (o10_)   -0.782    0.236   -3.321    0.001   -0.782   -0.999
##     O                 3.935    0.031  127.302    0.000    8.766    8.766
##    .O                 0.000                               0.000    0.000
##    .O                 0.000                               0.000    0.000
##    .O                 0.000                               0.000    0.000
##    .s                 0.000                               0.000    0.000
##    .s                 0.000                               0.000    0.000
##    .s                 0.000                               0.000    0.000
##    .s                 0.000                               0.000    0.000
##     d  (gamma_jc2)   -0.048    0.010   -4.952    0.000   -0.315   -0.315
##     d  (gamma_jc3)    0.007    0.009    0.724    0.469    0.046    0.046
##     d  (gamma_jc4)   -0.064    0.012   -5.337    0.000   -0.365   -0.365
##     d (gamma_ojc2)   -0.059    0.019   -3.019    0.003   -0.146   -0.146
##     d (gamma_ojc3)   -0.095    0.020   -4.631    0.000   -0.237   -0.237
##     d (gamma_ojc4)    0.036    0.020    1.783    0.075    0.113    0.113
##    .s                 0.000                               0.000    0.000
##    .s                 0.000                               0.000    0.000
##    .s                 0.000                               0.000    0.000
##    .s                 0.000                               0.000    0.000
##     S                 3.606    0.036   99.634    0.000    5.318    5.318
##    .S                 0.347    0.186    1.862    0.063    0.527    0.527
##    .S                 0.276    0.186    1.482    0.138    0.417    0.417
##    .S                 0.069    0.220    0.312    0.755    0.103    0.103
##     c                 0.302    0.025   12.142    0.000    0.302    0.655
## 
## Variances:
##                    Estimate  Std.Err  z-value  P(>|z|)   Std.lv  Std.all
##     JC_W1             0.064    0.011    5.824    0.000    1.000    1.000
##    .JC_W2             0.000                               0.000    0.000
##    .JC_W3             0.000                               0.000    0.000
##    .JC_W4             0.000                               0.000    0.000
##     djc2              0.023    0.005    4.706    0.000    1.000    1.000
##     djc3              0.020    0.004    4.578    0.000    1.000    1.000
##     djc4              0.031    0.007    4.401    0.000    1.000    1.000
##     OJC_W1            0.202    0.023    8.838    0.000    1.000    1.000
##    .OJC_W2            0.000                               0.000    0.000
##    .OJC_W3            0.000                               0.000    0.000
##    .OJC_W4            0.000                               0.000    0.000
##     dojc2             0.162    0.020    8.231    0.000    1.000    1.000
##     dojc3             0.160    0.020    8.030    0.000    1.000    1.000
##     dojc4             0.101    0.016    6.478    0.000    1.000    1.000
##    .s38.3r  (rs_s)    0.148    0.012   12.331    0.000    0.148    0.243
##    .s38.4r  (rs_s)    0.148    0.012   12.331    0.000    0.148    0.253
##    .s38.6   (rs_s)    0.148    0.012   12.331    0.000    0.148    0.252
##    .s38.7   (rs_s)    0.148    0.012   12.331    0.000    0.148    0.250
##    .s21_r.3           0.391    0.029   13.440    0.000    0.391    0.860
##    .s21_s.3           0.262    0.022   11.924    0.000    0.262    0.669
##    .s21_c.3           0.466    0.041   11.510    0.000    0.466    0.632
##    .s21_s.3           0.209    0.023    9.304    0.000    0.209    0.507
##    .s21_r.4           0.380    0.025   15.405    0.000    0.380    0.829
##    .s21_s.4           0.251    0.019   13.504    0.000    0.251    0.612
##    .s21_c.4           0.405    0.033   12.340    0.000    0.405    0.548
##    .s21_s.4           0.196    0.020   10.021    0.000    0.196    0.439
##    .s21_r.6           0.362    0.029   12.669    0.000    0.362    0.820
##    .s21_s.6           0.285    0.024   11.658    0.000    0.285    0.638
##    .s21_c.6           0.470    0.043   10.851    0.000    0.470    0.581
##    .s21_s.6           0.208    0.023    8.886    0.000    0.208    0.450
##    .s21_r.7           0.414    0.037   11.276    0.000    0.414    0.834
##    .s21_s.7           0.321    0.029   10.879    0.000    0.321    0.657
##    .s21_c.7           0.386    0.042    9.268    0.000    0.386    0.523
##    .s21_s.7           0.177    0.024    7.280    0.000    0.177    0.401
##    .s200_.3           0.560    0.040   14.110    0.000    0.560    0.735
##    .s200_.3           0.458    0.034   13.644    0.000    0.458    0.707
##    .s200_.3           0.205    0.021   10.009    0.000    0.205    0.368
##    .s200_.3           0.338    0.029   11.518    0.000    0.338    0.473
##    .s200_.3           0.249    0.023   11.062    0.000    0.249    0.434
##    .s200_.3           0.360    0.029   12.329    0.000    0.360    0.564
##    .s200_.4           0.587    0.036   16.486    0.000    0.587    0.703
##    .s200_.4           0.440    0.028   15.761    0.000    0.440    0.653
##    .s200_.4           0.220    0.019   11.886    0.000    0.220    0.336
##    .s200_.4           0.260    0.021   12.429    0.000    0.260    0.359
##    .s200_.4           0.240    0.019   12.497    0.000    0.240    0.375
##    .s200_.4           0.433    0.030   14.536    0.000    0.433    0.558
##    .s200_.6           0.483    0.035   13.863    0.000    0.483    0.681
##    .s200_.6           0.387    0.030   12.950    0.000    0.387    0.645
##    .s200_.6           0.148    0.017    8.785    0.000    0.148    0.272
##    .s200_.6           0.210    0.021   10.134    0.000    0.210    0.332
##    .s200_.6           0.212    0.021   10.220    0.000    0.212    0.367
##    .s200_.6           0.356    0.031   11.473    0.000    0.356    0.533
##    .s200_.7           0.514    0.041   12.500    0.000    0.514    0.709
##    .s200_.7           0.347    0.030   11.678    0.000    0.347    0.636
##    .s200_.7           0.172    0.021    8.312    0.000    0.172    0.317
##    .s200_.7           0.208    0.023    8.901    0.000    0.208    0.346
##    .s200_.7           0.230    0.025    9.352    0.000    0.230    0.403
##    .s200_.7           0.323    0.032   10.118    0.000    0.323    0.526
##     c137.6            0.214    0.017   12.590    0.000    0.214    1.000
##     SRH_W1            0.460    0.041   11.152    0.000    1.000    1.000
##    .SRH_W2            0.015    0.021    0.699    0.484    0.034    0.034
##    .SRH_W3            0.030    0.019    1.641    0.101    0.069    0.069
##    .SRH_W4            0.035    0.026    1.309    0.191    0.078    0.078
## 
## 
## Group 2 [alone]:
## 
## Latent Variables:
##                    Estimate  Std.Err  z-value  P(>|z|)   Std.lv  Std.all
##   JC_W1 =~                                                              
##     s21_r.3           1.000                               0.265    0.350
##     s21_s.3  (jc1)    1.426    0.124   11.494    0.000    0.377    0.620
##     s21_c.3  (jc2)    2.066    0.177   11.664    0.000    0.547    0.620
##     s21_s.3  (jc3)    1.790    0.151   11.862    0.000    0.474    0.770
##   JC_W2 =~                                                              
##     s21_r.4           1.000                               0.245    0.376
##     s21_s.4  (jc1)    1.426    0.124   11.494    0.000    0.350    0.556
##     s21_c.4  (jc2)    2.066    0.177   11.664    0.000    0.507    0.617
##     s21_s.4  (jc3)    1.790    0.151   11.862    0.000    0.439    0.715
##   JC_W3 =~                                                              
##     s21_r.6           1.000                               0.241    0.324
##     s21_s.6  (jc1)    1.426    0.124   11.494    0.000    0.343    0.510
##     s21_c.6  (jc2)    2.066    0.177   11.664    0.000    0.497    0.573
##     s21_s.6  (jc3)    1.790    0.151   11.862    0.000    0.431    0.747
##   JC_W4 =~                                                              
##     s21_r.7           1.000                               0.236    0.303
##     s21_s.7  (jc1)    1.426    0.124   11.494    0.000    0.337    0.543
##     s21_c.7  (jc2)    2.066    0.177   11.664    0.000    0.488    0.630
##     s21_s.7  (jc3)    1.790    0.151   11.862    0.000    0.423    0.660
##   djc2 =~                                                               
##     JC_W2             1.000                               0.608    0.608
##   djc3 =~                                                               
##     JC_W3             1.000                               0.577    0.577
##   djc4 =~                                                               
##     JC_W4             1.000                               0.626    0.626
##   OJC_W1 =~                                                             
##     s200_.3           1.000                               0.448    0.487
##     s200_.3 (ojc1)    0.971    0.041   23.411    0.000    0.435    0.534
##     s200_.3 (ojc2)    1.323    0.058   22.888    0.000    0.593    0.748
##     s200_.3 (ojc3)    1.368    0.064   21.383    0.000    0.613    0.706
##     s200_.3 (ojc4)    1.270    0.060   21.066    0.000    0.569    0.769
##     s200_.3 (ojc5)    1.174    0.060   19.486    0.000    0.526    0.620
##   OJC_W2 =~                                                             
##     s200_.4           1.000                               0.528    0.568
##     s200_.4 (ojc1)    0.971    0.041   23.411    0.000    0.513    0.634
##     s200_.4 (ojc2)    1.323    0.058   22.888    0.000    0.699    0.799
##     s200_.4 (ojc3)    1.368    0.064   21.383    0.000    0.722    0.799
##     s200_.4 (ojc4)    1.270    0.060   21.066    0.000    0.671    0.790
##     s200_.4 (ojc5)    1.174    0.060   19.486    0.000    0.620    0.591
##   OJC_W3 =~                                                             
##     s200_.6           1.000                               0.476    0.554
##     s200_.6 (ojc1)    0.971    0.041   23.411    0.000    0.462    0.584
##     s200_.6 (ojc2)    1.323    0.058   22.888    0.000    0.630    0.837
##     s200_.6 (ojc3)    1.368    0.064   21.383    0.000    0.651    0.817
##     s200_.6 (ojc4)    1.270    0.060   21.066    0.000    0.605    0.770
##     s200_.6 (ojc5)    1.174    0.060   19.486    0.000    0.559    0.579
##   OJC_W4 =~                                                             
##     s200_.7           1.000                               0.522    0.562
##     s200_.7 (ojc1)    0.971    0.041   23.411    0.000    0.507    0.601
##     s200_.7 (ojc2)    1.323    0.058   22.888    0.000    0.691    0.866
##     s200_.7 (ojc3)    1.368    0.064   21.383    0.000    0.714    0.845
##     s200_.7 (ojc4)    1.270    0.060   21.066    0.000    0.663    0.857
##     s200_.7 (ojc5)    1.174    0.060   19.486    0.000    0.613    0.717
##   dojc2 =~                                                              
##     OJC_W2            1.000                               0.888    0.888
##   dojc3 =~                                                              
##     OJC_W3            1.000                               1.018    1.018
##   dojc4 =~                                                              
##     OJC_W4            1.000                               0.775    0.775
##   SRH_W1 =~                                                             
##     s38.3r            1.000                               0.659    0.864
##   SRH_W2 =~                                                             
##     s38.4r            1.000                               0.652    0.861
##   SRH_W3 =~                                                             
##     s38.6             1.000                               0.652    0.862
##   SRH_W4 =~                                                             
##     s38.7             1.000                               0.678    0.870
## 
## Regressions:
##                    Estimate  Std.Err  z-value  P(>|z|)   Std.lv  Std.all
##   JC_W2 ~                                                               
##     JC_W1             1.000                               1.079    1.079
##   JC_W3 ~                                                               
##     JC_W2             1.000                               1.020    1.020
##   JC_W4 ~                                                               
##     JC_W3             1.000                               1.018    1.018
##   OJC_W2 ~                                                              
##     OJC_W1            1.000                               0.849    0.849
##   OJC_W3 ~                                                              
##     OJC_W2            1.000                               1.109    1.109
##   OJC_W4 ~                                                              
##     OJC_W3            1.000                               0.912    0.912
##   SRH_W2 ~                                                              
##     SRH_W1  (ar1_)    0.935    0.049   19.242    0.000    0.946    0.946
##   SRH_W3 ~                                                              
##     SRH_W2  (ar2_)    0.934    0.048   19.443    0.000    0.934    0.934
##   SRH_W4 ~                                                              
##     SRH_W3  (ar3_)    0.966    0.056   17.114    0.000    0.929    0.929
##   SRH_W2 ~                                                              
##     djc2    (jcs1)    0.450    0.206    2.183    0.029    0.103    0.103
##     dojc2   (ojc1)    0.115    0.060    1.912    0.056    0.083    0.083
##     c137.6  (cvd1)   -0.058    0.067   -0.858    0.391   -0.089   -0.037
##   SRH_W3 ~                                                              
##     djc3    (jcs2)    0.428    0.260    1.651    0.099    0.091    0.091
##     dojc3   (ojc2)    0.112    0.060    1.861    0.063    0.083    0.083
##     c137.6  (cvd2)   -0.023    0.065   -0.352    0.725   -0.035   -0.015
##   SRH_W4 ~                                                              
##     djc4    (jcs3)    0.112    0.245    0.457    0.648    0.024    0.024
##     dojc4             0.053    0.144    0.370    0.712    0.032    0.032
##     c137.6            0.414    0.140    2.957    0.003    0.611    0.257
## 
## Covariances:
##                    Estimate  Std.Err  z-value  P(>|z|)   Std.lv  Std.all
##  .s21_rd.3 ~~                                                           
##    .s21_r.4           0.241    0.038    6.276    0.000    0.241    0.563
##    .s21_r.6           0.294    0.050    5.935    0.000    0.294    0.594
##    .s21_r.7           0.337    0.054    6.245    0.000    0.337    0.643
##  .s21_rd.4 ~~                                                           
##    .s21_r.6           0.218    0.039    5.624    0.000    0.218    0.515
##    .s21_r.7           0.252    0.042    5.940    0.000    0.252    0.561
##  .s21_rd.6 ~~                                                           
##    .s21_r.7           0.371    0.054    6.841    0.000    0.371    0.713
##  .s21_str.3 ~~                                                          
##    .s21_s.4           0.168    0.026    6.408    0.000    0.168    0.673
##    .s21_s.6           0.214    0.032    6.731    0.000    0.214    0.773
##    .s21_s.7           0.183    0.030    6.123    0.000    0.183    0.735
##  .s21_str.4 ~~                                                          
##    .s21_s.6           0.229    0.032    7.129    0.000    0.229    0.758
##    .s21_s.7           0.205    0.030    6.886    0.000    0.205    0.753
##  .s21_str.6 ~~                                                          
##    .s21_s.7           0.246    0.036    6.838    0.000    0.246    0.815
##  .s21_cd.3 ~~                                                           
##    .s21_c.4           0.290    0.047    6.130    0.000    0.290    0.649
##    .s21_c.6           0.292    0.056    5.198    0.000    0.292    0.595
##    .s21_c.7           0.248    0.052    4.797    0.000    0.248    0.597
##  .s21_cd.4 ~~                                                           
##    .s21_c.6           0.307    0.051    6.067    0.000    0.307    0.669
##    .s21_c.7           0.219    0.046    4.795    0.000    0.219    0.564
##  .s21_cd.6 ~~                                                           
##    .s21_c.7           0.299    0.054    5.573    0.000    0.299    0.701
##  .s21_soz.3 ~~                                                          
##    .s21_s.4           0.069    0.022    3.112    0.002    0.069    0.411
##    .s21_s.6           0.065    0.023    2.855    0.004    0.065    0.432
##    .s21_s.7           0.088    0.027    3.313    0.001    0.088    0.468
##  .s21_soz.4 ~~                                                          
##    .s21_s.6           0.067    0.024    2.798    0.005    0.067    0.407
##    .s21_s.7           0.046    0.027    1.683    0.092    0.046    0.221
##  .s21_soz.6 ~~                                                          
##    .s21_s.7           0.071    0.028    2.518    0.012    0.071    0.387
##   djc2 ~~                                                               
##     djc3             -0.002    0.004   -0.444    0.657   -0.093   -0.093
##     djc4             -0.006    0.005   -1.399    0.162   -0.293   -0.293
##   djc3 ~~                                                               
##     djc4             -0.009    0.005   -1.963    0.050   -0.439   -0.439
##  .s200_de.3 ~~                                                          
##    .s200_.3           0.165    0.041    4.058    0.000    0.165    0.298
##  .s200_de.4 ~~                                                          
##    .s200_.4           0.111    0.031    3.557    0.000    0.111    0.233
##  .s200_de.6 ~~                                                          
##    .s200_.6           0.072    0.033    2.180    0.029    0.072    0.156
##  .s200_de.7 ~~                                                          
##    .s200_.7           0.249    0.049    5.126    0.000    0.249    0.481
##  .s200_de.3 ~~                                                          
##    .s200_.4           0.296    0.053    5.634    0.000    0.296    0.482
##    .s200_.6           0.297    0.054    5.492    0.000    0.297    0.517
##    .s200_.7           0.252    0.054    4.676    0.000    0.252    0.407
##  .s200_de.4 ~~                                                          
##    .s200_.6           0.217    0.048    4.545    0.000    0.217    0.396
##    .s200_.7           0.146    0.046    3.150    0.002    0.146    0.249
##  .s200_de.6 ~~                                                          
##    .s200_.7           0.225    0.049    4.629    0.000    0.225    0.409
##  .s200_re.3 ~~                                                          
##    .s200_.4           0.097    0.036    2.709    0.007    0.097    0.224
##    .s200_.6           0.166    0.043    3.827    0.000    0.166    0.374
##    .s200_.7           0.121    0.038    3.214    0.001    0.121    0.260
##  .s200_re.4 ~~                                                          
##    .s200_.6           0.202    0.037    5.464    0.000    0.202    0.501
##    .s200_.7           0.146    0.035    4.193    0.000    0.146    0.347
##  .s200_re.6 ~~                                                          
##    .s200_.7           0.166    0.039    4.292    0.000    0.166    0.382
##  .s200_au.3 ~~                                                          
##    .s200_.4           0.107    0.031    3.427    0.001    0.107    0.387
##    .s200_.6           0.093    0.029    3.241    0.001    0.093    0.427
##    .s200_.7           0.079    0.028    2.839    0.005    0.079    0.374
##  .s200_au.4 ~~                                                          
##    .s200_.6           0.023    0.025    0.901    0.368    0.023    0.105
##    .s200_.7           0.025    0.026    0.973    0.330    0.025    0.119
##  .s200_au.6 ~~                                                          
##    .s200_.7           0.066    0.023    2.836    0.005    0.066    0.404
##  .s200_ma.3 ~~                                                          
##    .s200_.4           0.103    0.036    2.830    0.005    0.103    0.308
##    .s200_.6           0.081    0.035    2.300    0.021    0.081    0.287
##    .s200_.7           0.089    0.034    2.585    0.010    0.089    0.319
##  .s200_ma.4 ~~                                                          
##    .s200_.6           0.074    0.028    2.690    0.007    0.074    0.296
##    .s200_.7           0.081    0.028    2.921    0.003    0.081    0.328
##  .s200_ma.6 ~~                                                          
##    .s200_.7           0.084    0.026    3.159    0.002    0.084    0.403
##  .s200_me.3 ~~                                                          
##    .s200_.4           0.077    0.027    2.840    0.005    0.077    0.312
##    .s200_.6           0.086    0.032    2.652    0.008    0.086    0.363
##    .s200_.7           0.048    0.025    1.912    0.056    0.048    0.251
##  .s200_me.4 ~~                                                          
##    .s200_.6           0.085    0.029    2.889    0.004    0.085    0.325
##    .s200_.7           0.067    0.028    2.413    0.016    0.067    0.323
##  .s200_me.6 ~~                                                          
##    .s200_.7           0.065    0.027    2.381    0.017    0.065    0.325
##  .s200_af.3 ~~                                                          
##    .s200_.4           0.203    0.053    3.800    0.000    0.203    0.359
##    .s200_.6           0.274    0.059    4.652    0.000    0.274    0.521
##    .s200_.7           0.243    0.044    5.497    0.000    0.243    0.613
##  .s200_af.4 ~~                                                          
##    .s200_.6           0.342    0.062    5.502    0.000    0.342    0.512
##    .s200_.7           0.192    0.051    3.752    0.000    0.192    0.381
##  .s200_af.6 ~~                                                          
##    .s200_.7           0.191    0.050    3.845    0.000    0.191    0.408
##   dojc2 ~~                                                              
##     dojc3            -0.145    0.029   -4.944    0.000   -0.636   -0.636
##     dojc4             0.044    0.023    1.893    0.058    0.231    0.231
##   dojc3 ~~                                                              
##     dojc4            -0.103    0.026   -4.017    0.000   -0.524   -0.524
##   djc2 ~~                                                               
##     dojc2   (cov1)    0.008    0.003    2.527    0.011    0.108    0.108
##   djc3 ~~                                                               
##     dojc3   (cov1)    0.008    0.003    2.527    0.011    0.112    0.112
##   djc4 ~~                                                               
##     dojc4   (cov1)    0.008    0.003    2.527    0.011    0.126    0.126
##   JC_W1 ~~                                                              
##     c137.6           -0.009    0.012   -0.731    0.465   -0.033   -0.077
##   OJC_W1 ~~                                                             
##     c137.6            0.007    0.019    0.388    0.698    0.017    0.039
##   SRH_W1 ~~                                                             
##     c137.6           -0.077    0.028   -2.792    0.005   -0.118   -0.279
##   djc2 ~~                                                               
##     c137.6            0.002    0.009    0.185    0.853    0.011    0.025
##   djc3 ~~                                                               
##     c137.6           -0.002    0.007   -0.307    0.759   -0.017   -0.039
##   djc4 ~~                                                               
##     c137.6           -0.017    0.008   -2.006    0.045   -0.115   -0.273
##   dojc2 ~~                                                              
##     c137.6            0.006    0.020    0.304    0.761    0.013    0.031
##   dojc3 ~~                                                              
##     c137.6           -0.007    0.019   -0.372    0.710   -0.015   -0.035
##   dojc4 ~~                                                              
##     c137.6            0.006    0.018    0.348    0.728    0.015    0.036
##   JC_W1 ~~                                                              
##     djc2             -0.016    0.006   -2.606    0.009   -0.406   -0.406
##     djc3             -0.009    0.006   -1.516    0.130   -0.242   -0.242
##     djc4              0.004    0.006    0.575    0.565    0.090    0.090
##     OJC_W1            0.054    0.013    4.142    0.000    0.458    0.458
##     dojc2            -0.013    0.011   -1.144    0.252   -0.101   -0.101
##     dojc3            -0.011    0.013   -0.863    0.388   -0.089   -0.089
##     dojc4             0.003    0.013    0.272    0.786    0.032    0.032
##     SRH_W1            0.030    0.016    1.840    0.066    0.173    0.173
##   djc2 ~~                                                               
##     OJC_W1           -0.019    0.008   -2.389    0.017   -0.284   -0.284
##     dojc3             0.012    0.008    1.395    0.163    0.161    0.161
##     dojc4            -0.006    0.009   -0.688    0.491   -0.101   -0.101
##     SRH_W1           -0.013    0.012   -1.069    0.285   -0.129   -0.129
##   djc3 ~~                                                               
##     OJC_W1           -0.015    0.009   -1.561    0.119   -0.235   -0.235
##     dojc2            -0.002    0.008   -0.261    0.794   -0.032   -0.032
##     dojc4             0.007    0.007    0.918    0.358    0.119    0.119
##     SRH_W1            0.004    0.012    0.362    0.717    0.048    0.048
##   djc4 ~~                                                               
##     OJC_W1            0.010    0.010    1.094    0.274    0.158    0.158
##     dojc2            -0.010    0.010   -0.970    0.332   -0.137   -0.137
##     dojc3            -0.004    0.008   -0.471    0.638   -0.052   -0.052
##     SRH_W1            0.026    0.014    1.829    0.067    0.262    0.262
##   OJC_W1 ~~                                                             
##     dojc2            -0.071    0.022   -3.250    0.001   -0.338   -0.338
##     dojc3             0.001    0.023    0.055    0.956    0.006    0.006
##     dojc4             0.000    0.021    0.003    0.998    0.000    0.000
##     SRH_W1            0.070    0.027    2.623    0.009    0.238    0.238
##   dojc2 ~~                                                              
##     SRH_W1           -0.018    0.029   -0.637    0.524   -0.059   -0.059
##   dojc3 ~~                                                              
##     SRH_W1            0.015    0.031    0.499    0.618    0.048    0.048
##   dojc4 ~~                                                              
##     SRH_W1           -0.029    0.029   -0.991    0.322   -0.109   -0.109
## 
## Intercepts:
##                    Estimate  Std.Err  z-value  P(>|z|)   Std.lv  Std.all
##    .s        (jc6)   -0.347    0.358   -0.970    0.332   -0.347   -0.570
##    .s        (jc6)   -0.347    0.358   -0.970    0.332   -0.347   -0.551
##    .s        (jc6)   -0.347    0.358   -0.970    0.332   -0.347   -0.516
##    .s        (jc6)   -0.347    0.358   -0.970    0.332   -0.347   -0.559
##    .s        (jc4)   -3.505    0.510   -6.877    0.000   -3.505   -3.975
##    .s        (jc4)   -3.505    0.510   -6.877    0.000   -3.505   -4.265
##    .s        (jc4)   -3.505    0.510   -6.877    0.000   -3.505   -4.044
##    .s        (jc4)   -3.505    0.510   -6.877    0.000   -3.505   -4.524
##    .s        (jc5)   -2.593    0.434   -5.974    0.000   -2.593   -4.216
##    .s        (jc5)   -2.593    0.434   -5.974    0.000   -2.593   -4.224
##    .s        (jc5)   -2.593    0.434   -5.974    0.000   -2.593   -4.498
##    .s        (jc5)   -2.593    0.434   -5.974    0.000   -2.593   -4.048
##     J                 2.872    0.028  103.983    0.000   10.854   10.854
##    .J                 0.000                               0.000    0.000
##    .J                 0.000                               0.000    0.000
##    .J                 0.000                               0.000    0.000
##    .s                 0.000                               0.000    0.000
##    .s                 0.000                               0.000    0.000
##    .s                 0.000                               0.000    0.000
##    .s                 0.000                               0.000    0.000
##    .s       (ojc6)   -0.011    0.162   -0.068    0.946   -0.011   -0.014
##    .s       (ojc6)   -0.011    0.162   -0.068    0.946   -0.011   -0.014
##    .s       (ojc6)   -0.011    0.162   -0.068    0.946   -0.011   -0.014
##    .s       (ojc6)   -0.011    0.162   -0.068    0.946   -0.011   -0.013
##    .s       (o7_2)   -1.221    0.227   -5.387    0.000   -1.221   -1.539
##    .s       (o7_2)   -1.221    0.227   -5.387    0.000   -1.221   -1.396
##    .s       (o7_2)   -1.221    0.227   -5.387    0.000   -1.221   -1.622
##    .s       (o7_2)   -1.221    0.227   -5.387    0.000   -1.221   -1.531
##    .s       (ojc8)   -1.864    0.249   -7.478    0.000   -1.864   -2.147
##    .s       (ojc8)   -1.864    0.249   -7.478    0.000   -1.864   -2.061
##    .s       (ojc8)   -1.864    0.249   -7.478    0.000   -1.864   -2.337
##    .s       (ojc8)   -1.864    0.249   -7.478    0.000   -1.864   -2.206
##    .s       (ojc9)   -1.355    0.235   -5.762    0.000   -1.355   -1.829
##    .s       (ojc9)   -1.355    0.235   -5.762    0.000   -1.355   -1.596
##    .s       (ojc9)   -1.355    0.235   -5.762    0.000   -1.355   -1.725
##    .s       (ojc9)   -1.355    0.235   -5.762    0.000   -1.355   -1.749
##    .s       (o10_)   -1.000    0.238   -4.200    0.000   -1.000   -1.178
##    .s       (o10_)   -1.000    0.238   -4.200    0.000   -1.000   -0.953
##    .s       (o10_)   -1.000    0.238   -4.200    0.000   -1.000   -1.035
##    .s       (o10_)   -1.000    0.238   -4.200    0.000   -1.000   -1.171
##     O                 3.963    0.039  101.084    0.000    8.839    8.839
##    .O                 0.000                               0.000    0.000
##    .O                 0.000                               0.000    0.000
##    .O                 0.000                               0.000    0.000
##    .s                 0.000                               0.000    0.000
##    .s                 0.000                               0.000    0.000
##    .s                 0.000                               0.000    0.000
##    .s                 0.000                               0.000    0.000
##     d  (gamma_jc2)   -0.048    0.010   -4.952    0.000   -0.323   -0.323
##     d  (gamma_jc3)    0.007    0.009    0.724    0.469    0.047    0.047
##     d  (gamma_jc4)   -0.064    0.012   -5.337    0.000   -0.436   -0.436
##     d (gamma_ojc2)   -0.059    0.019   -3.019    0.003   -0.125   -0.125
##     d (gamma_ojc3)   -0.095    0.020   -4.631    0.000   -0.196   -0.196
##     d (gamma_ojc4)    0.036    0.020    1.783    0.075    0.088    0.088
##    .s                 0.000                               0.000    0.000
##    .s                 0.000                               0.000    0.000
##    .s                 0.000                               0.000    0.000
##    .s                 0.000                               0.000    0.000
##     S                 3.583    0.056   64.470    0.000    5.435    5.435
##    .S                 0.342    0.186    1.840    0.066    0.526    0.526
##    .S                 0.270    0.188    1.434    0.152    0.414    0.414
##    .S                 0.014    0.223    0.063    0.950    0.021    0.021
##     c                 0.242    0.036    6.662    0.000    0.242    0.574
## 
## Variances:
##                    Estimate  Std.Err  z-value  P(>|z|)   Std.lv  Std.all
##     JC_W1             0.070    0.015    4.800    0.000    1.000    1.000
##    .JC_W2             0.000                               0.000    0.000
##    .JC_W3             0.000                               0.000    0.000
##    .JC_W4             0.000                               0.000    0.000
##     djc2              0.022    0.006    3.594    0.000    1.000    1.000
##     djc3              0.019    0.006    3.180    0.001    1.000    1.000
##     djc4              0.022    0.007    3.223    0.001    1.000    1.000
##     OJC_W1            0.201    0.031    6.514    0.000    1.000    1.000
##    .OJC_W2            0.000                               0.000    0.000
##    .OJC_W3            0.000                               0.000    0.000
##    .OJC_W4            0.000                               0.000    0.000
##     dojc2             0.220    0.035    6.337    0.000    1.000    1.000
##     dojc3             0.235    0.038    6.176    0.000    1.000    1.000
##     dojc4             0.164    0.030    5.438    0.000    1.000    1.000
##    .s38.3r  (rs_s)    0.148    0.012   12.331    0.000    0.148    0.253
##    .s38.4r  (rs_s)    0.148    0.012   12.331    0.000    0.148    0.258
##    .s38.6   (rs_s)    0.148    0.012   12.331    0.000    0.148    0.258
##    .s38.7   (rs_s)    0.148    0.012   12.331    0.000    0.148    0.243
##    .s21_r.3           0.500    0.058    8.664    0.000    0.500    0.877
##    .s21_s.3           0.228    0.031    7.440    0.000    0.228    0.616
##    .s21_c.3           0.478    0.063    7.598    0.000    0.478    0.615
##    .s21_s.3           0.154    0.027    5.648    0.000    0.154    0.407
##    .s21_r.4           0.366    0.037    9.762    0.000    0.366    0.859
##    .s21_s.4           0.274    0.031    8.704    0.000    0.274    0.691
##    .s21_c.4           0.418    0.051    8.277    0.000    0.418    0.619
##    .s21_s.4           0.184    0.028    6.621    0.000    0.184    0.488
##    .s21_r.6           0.492    0.059    8.328    0.000    0.492    0.895
##    .s21_s.6           0.335    0.044    7.644    0.000    0.335    0.740
##    .s21_c.6           0.504    0.070    7.234    0.000    0.504    0.671
##    .s21_s.6           0.147    0.031    4.808    0.000    0.147    0.442
##    .s21_r.7           0.550    0.069    8.007    0.000    0.550    0.908
##    .s21_s.7           0.271    0.038    7.106    0.000    0.271    0.705
##    .s21_c.7           0.362    0.059    6.104    0.000    0.362    0.603
##    .s21_s.7           0.232    0.041    5.651    0.000    0.232    0.564
##    .s200_.3           0.646    0.071    9.133    0.000    0.646    0.763
##    .s200_.3           0.474    0.053    8.895    0.000    0.474    0.715
##    .s200_.3           0.277    0.042    6.663    0.000    0.277    0.441
##    .s200_.3           0.378    0.053    7.198    0.000    0.378    0.501
##    .s200_.3           0.224    0.036    6.249    0.000    0.224    0.409
##    .s200_.3           0.445    0.058    7.607    0.000    0.445    0.616
##    .s200_.4           0.585    0.060    9.777    0.000    0.585    0.677
##    .s200_.4           0.392    0.041    9.658    0.000    0.392    0.598
##    .s200_.4           0.277    0.036    7.653    0.000    0.277    0.362
##    .s200_.4           0.296    0.038    7.724    0.000    0.296    0.362
##    .s200_.4           0.270    0.035    7.765    0.000    0.270    0.375
##    .s200_.4           0.718    0.078    9.242    0.000    0.718    0.651
##    .s200_.6           0.513    0.061    8.410    0.000    0.513    0.693
##    .s200_.6           0.414    0.052    7.940    0.000    0.414    0.659
##    .s200_.6           0.169    0.030    5.682    0.000    0.169    0.299
##    .s200_.6           0.212    0.034    6.205    0.000    0.212    0.333
##    .s200_.6           0.251    0.038    6.514    0.000    0.251    0.406
##    .s200_.6           0.622    0.079    7.910    0.000    0.622    0.665
##    .s200_.7           0.590    0.071    8.296    0.000    0.590    0.684
##    .s200_.7           0.454    0.058    7.785    0.000    0.454    0.639
##    .s200_.7           0.159    0.029    5.445    0.000    0.159    0.249
##    .s200_.7           0.204    0.034    5.942    0.000    0.204    0.285
##    .s200_.7           0.160    0.029    5.486    0.000    0.160    0.266
##    .s200_.7           0.354    0.051    6.997    0.000    0.354    0.485
##     c137.6            0.177    0.022    8.067    0.000    0.177    1.000
##     SRH_W1            0.435    0.056    7.825    0.000    1.000    1.000
##    .SRH_W2            0.042    0.032    1.312    0.190    0.099    0.099
##    .SRH_W3            0.040    0.028    1.420    0.156    0.095    0.095
##    .SRH_W4            0.098    0.044    2.215    0.027    0.214    0.214
```

```
# FINAL MODEL: lcsm_parcel_SRH_GC2_equalARc137W23COVJCOJCW23_timeequalCOV_equalCSJC123OJC123
```

### Major contract changes vs. no contract changes

```
# For this group comparison, the same measurement model as for the full sample can not be used, as the factor loading from JC_SOZ needs to be released.

## LCSM Job and Off-Job Crafting with self-rated health as outcome ----

### Model estimation ----
model_lcsm_parcel_SRH_GC4_equalARc137COVW34JCOJCW24_timeequalCOV_equalCSJC123OJC123 <- paste(measurement_model_parcelled_jc_ojc_GC4_BASE, fixed_cs_jc_123, fixed_cs_ojc_123, measurement_model_srh_equalAR, model_latent_srh_equalc137COVW34JCOJCW24_timeequalCOV, sep = "")

lcsm_parcel_SRH_GC4_equalARc137COVW34JCOJCW24_timeequalCOV_equalCSJC123OJC123 <- sem(model_lcsm_parcel_SRH_GC4_equalARc137COVW34JCOJCW24_timeequalCOV_equalCSJC123OJC123, data, 
                   estimator = "ML", 
                   missing = "ML",
                   group = "group4_contractchanges.4",
                   fixed.x = FALSE); beep(6) # Has to be set to zero, otherwise the missings in the exogenous var c137.6 cause deletion of cases

summary(lcsm_parcel_SRH_GC4_equalARc137COVW34JCOJCW24_timeequalCOV_equalCSJC123OJC123, fit.measures = TRUE, standardized = TRUE, fm.args = list(robust = FALSE))
```

```
## lavaan 0.6.16 ended normally after 348 iterations
## 
##   Estimator                                         ML
##   Optimization method                           NLMINB
##   Number of model parameters                       510
##   Number of equality constraints                   118
## 
##   Number of observations per group:                   
##     none                                           588
##     contractual reduction                          187
##   Number of missing patterns per group:               
##     none                                            33
##     contractual reduction                           19
## 
## Model Test User Model:
##                                                       
##   Test statistic                              3246.183
##   Degrees of freedom                              1768
##   P-value (Chi-square)                           0.000
##   Test statistic for each group:
##     none                                      1726.958
##     contractual reduction                     1519.225
## 
## Model Test Baseline Model:
## 
##   Test statistic                             18097.682
##   Degrees of freedom                              1980
##   P-value                                        0.000
## 
## User Model versus Baseline Model:
## 
##   Comparative Fit Index (CFI)                    0.908
##   Tucker-Lewis Index (TLI)                       0.897
## 
## Loglikelihood and Information Criteria:
## 
##   Loglikelihood user model (H0)             -21476.904
##   Loglikelihood unrestricted model (H1)     -19853.812
##                                                       
##   Akaike (AIC)                               43737.807
##   Bayesian (BIC)                             45561.729
##   Sample-size adjusted Bayesian (SABIC)      44316.943
## 
## Root Mean Square Error of Approximation:
## 
##   RMSEA                                          0.046
##   90 Percent confidence interval - lower         0.044
##   90 Percent confidence interval - upper         0.049
##   P-value H_0: RMSEA <= 0.050                    0.990
##   P-value H_0: RMSEA >= 0.080                    0.000
## 
## Standardized Root Mean Square Residual:
## 
##   SRMR                                           0.085
## 
## Parameter Estimates:
## 
##   Standard errors                             Standard
##   Information                                 Observed
##   Observed information based on                Hessian
## 
## 
## Group 1 [none]:
## 
## Latent Variables:
##                    Estimate  Std.Err  z-value  P(>|z|)   Std.lv  Std.all
##   JC_W1 =~                                                              
##     s21_c.3           1.000                               0.543    0.625
##     s21_s.3  (jc1)    0.711    0.043   16.357    0.000    0.386    0.603
##     s21_r.3           0.396    0.066    6.004    0.000    0.215    0.303
##     s21_s.3 (j3_1)    0.809    0.052   15.485    0.000    0.439    0.718
##   JC_W2 =~                                                              
##     s21_c.4           1.000                               0.571    0.678
##     s21_s.4  (jc1)    0.711    0.043   16.357    0.000    0.406    0.629
##     s21_r.4           0.469    0.054    8.665    0.000    0.268    0.402
##     s21_s.4 (j3_1)    0.809    0.052   15.485    0.000    0.462    0.720
##   JC_W3 =~                                                              
##     s21_c.6           1.000                               0.549    0.620
##     s21_s.6  (jc1)    0.711    0.043   16.357    0.000    0.390    0.572
##     s21_r.6           0.536    0.067    7.965    0.000    0.294    0.422
##     s21_s.6 (j3_1)    0.809    0.052   15.485    0.000    0.444    0.708
##   JC_W4 =~                                                              
##     s21_c.7           1.000                               0.557    0.666
##     s21_s.7  (jc1)    0.711    0.043   16.357    0.000    0.396    0.575
##     s21_r.7           0.557    0.079    7.056    0.000    0.310    0.412
##     s21_s.7 (j3_1)    0.809    0.052   15.485    0.000    0.451    0.686
##   djc2 =~                                                               
##     JC_W2             1.000                               0.562    0.562
##   djc3 =~                                                               
##     JC_W3             1.000                               0.497    0.497
##   djc4 =~                                                               
##     JC_W4             1.000                               0.558    0.558
##   OJC_W1 =~                                                             
##     s200_.3           1.000                               0.428    0.478
##     s200_.3 (ojc1)    0.982    0.041   23.774    0.000    0.421    0.529
##     s200_.3 (ojc2)    1.351    0.059   22.983    0.000    0.579    0.759
##     s200_.3 (ojc3)    1.404    0.065   21.745    0.000    0.602    0.706
##     s200_.3 (ojc4)    1.306    0.062   21.213    0.000    0.560    0.752
##     s200_.3 (ojc5)    1.200    0.061   19.535    0.000    0.514    0.628
##   OJC_W2 =~                                                             
##     s200_.4           1.000                               0.503    0.552
##     s200_.4 (ojc1)    0.982    0.041   23.774    0.000    0.494    0.600
##     s200_.4 (ojc2)    1.351    0.059   22.983    0.000    0.680    0.799
##     s200_.4 (ojc3)    1.404    0.065   21.745    0.000    0.707    0.805
##     s200_.4 (ojc4)    1.306    0.062   21.213    0.000    0.658    0.794
##     s200_.4 (ojc5)    1.200    0.061   19.535    0.000    0.604    0.638
##   OJC_W3 =~                                                             
##     s200_.6           1.000                               0.458    0.539
##     s200_.6 (ojc1)    0.982    0.041   23.774    0.000    0.450    0.581
##     s200_.6 (ojc2)    1.351    0.059   22.983    0.000    0.619    0.831
##     s200_.6 (ojc3)    1.404    0.065   21.745    0.000    0.644    0.814
##     s200_.6 (ojc4)    1.306    0.062   21.213    0.000    0.599    0.782
##     s200_.6 (ojc5)    1.200    0.061   19.535    0.000    0.550    0.635
##   OJC_W4 =~                                                             
##     s200_.7           1.000                               0.476    0.533
##     s200_.7 (ojc1)    0.982    0.041   23.774    0.000    0.467    0.599
##     s200_.7 (ojc2)    1.351    0.059   22.983    0.000    0.643    0.832
##     s200_.7 (ojc3)    1.404    0.065   21.745    0.000    0.668    0.819
##     s200_.7 (ojc4)    1.306    0.062   21.213    0.000    0.621    0.815
##     s200_.7 (ojc5)    1.200    0.061   19.535    0.000    0.571    0.690
##   dojc2 =~                                                              
##     OJC_W2            1.000                               0.818    0.818
##   dojc3 =~                                                              
##     OJC_W3            1.000                               0.873    0.873
##   dojc4 =~                                                              
##     OJC_W4            1.000                               0.765    0.765
##   SRH_W1 =~                                                             
##     s38.3r            1.000                               0.649    0.857
##   SRH_W2 =~                                                             
##     s38.4r            1.000                               0.644    0.855
##   SRH_W3 =~                                                             
##     s38.6             1.000                               0.652    0.858
##   SRH_W4 =~                                                             
##     s38.7             1.000                               0.658    0.860
## 
## Regressions:
##                    Estimate  Std.Err  z-value  P(>|z|)   Std.lv  Std.all
##   JC_W2 ~                                                               
##     JC_W1             1.000                               0.952    0.952
##   JC_W3 ~                                                               
##     JC_W2             1.000                               1.040    1.040
##   JC_W4 ~                                                               
##     JC_W3             1.000                               0.985    0.985
##   OJC_W2 ~                                                              
##     OJC_W1            1.000                               0.851    0.851
##   OJC_W3 ~                                                              
##     OJC_W2            1.000                               1.098    1.098
##   OJC_W4 ~                                                              
##     OJC_W3            1.000                               0.963    0.963
##   SRH_W2 ~                                                              
##     SRH_W1  (ar1_)    0.952    0.051   18.803    0.000    0.960    0.960
##   SRH_W3 ~                                                              
##     SRH_W2  (ar2_)    0.946    0.048   19.850    0.000    0.935    0.935
##   SRH_W4 ~                                                              
##     SRH_W3  (ar3_)    0.949    0.055   17.277    0.000    0.940    0.940
##   SRH_W2 ~                                                              
##     djc2    (jcs1)    0.253    0.098    2.587    0.010    0.126    0.126
##     dojc2   (ojc1)    0.094    0.061    1.540    0.123    0.060    0.060
##     c137.6  (cvd1)   -0.050    0.069   -0.724    0.469   -0.077   -0.035
##   SRH_W3 ~                                                              
##     djc3    (jcs2)    0.206    0.120    1.715    0.086    0.086    0.086
##     dojc3             0.160    0.074    2.158    0.031    0.099    0.099
##     c137.6  (cvd2)    0.006    0.065    0.097    0.923    0.010    0.004
##   SRH_W4 ~                                                              
##     djc4    (jcs3)    0.003    0.112    0.028    0.978    0.001    0.001
##     dojc4   (ojc3)    0.209    0.087    2.394    0.017    0.116    0.116
##     c137.6  (cvd3)    0.103    0.074    1.384    0.166    0.156    0.071
## 
## Covariances:
##                    Estimate  Std.Err  z-value  P(>|z|)   Std.lv  Std.all
##  .s21_rd.3 ~~                                                           
##    .s21_r.4           0.214    0.022    9.693    0.000    0.214    0.518
##    .s21_r.6           0.242    0.026    9.353    0.000    0.242    0.564
##    .s21_r.7           0.267    0.029    9.167    0.000    0.267    0.574
##  .s21_rd.4 ~~                                                           
##    .s21_r.6           0.217    0.021   10.158    0.000    0.217    0.563
##    .s21_r.7           0.197    0.025    8.020    0.000    0.197    0.471
##  .s21_rd.6 ~~                                                           
##    .s21_r.7           0.255    0.028    9.206    0.000    0.255    0.589
##  .s21_str.3 ~~                                                          
##    .s21_s.4           0.158    0.017    9.582    0.000    0.158    0.617
##    .s21_s.6           0.203    0.020   10.303    0.000    0.203    0.708
##    .s21_s.7           0.209    0.021   10.032    0.000    0.209    0.724
##  .s21_str.4 ~~                                                          
##    .s21_s.6           0.199    0.018   10.761    0.000    0.199    0.707
##    .s21_s.7           0.191    0.019   10.002    0.000    0.191    0.676
##  .s21_str.6 ~~                                                          
##    .s21_s.7           0.231    0.023   10.253    0.000    0.231    0.733
##  .s21_cd.3 ~~                                                           
##    .s21_c.4           0.268    0.029    9.210    0.000    0.268    0.639
##    .s21_c.6           0.304    0.035    8.754    0.000    0.304    0.644
##    .s21_c.7           0.243    0.033    7.277    0.000    0.243    0.574
##  .s21_cd.4 ~~                                                           
##    .s21_c.6           0.283    0.031    9.048    0.000    0.283    0.659
##    .s21_c.7           0.226    0.030    7.563    0.000    0.226    0.585
##  .s21_cd.6 ~~                                                           
##    .s21_c.7           0.293    0.036    8.174    0.000    0.293    0.675
##  .s21_soz.3 ~~                                                          
##    .s21_s.4           0.108    0.016    6.889    0.000    0.108    0.567
##    .s21_s.6           0.107    0.017    6.422    0.000    0.107    0.565
##    .s21_s.7           0.104    0.018    5.862    0.000    0.104    0.509
##  .s21_soz.4 ~~                                                          
##    .s21_s.6           0.109    0.016    6.791    0.000    0.109    0.554
##    .s21_s.7           0.098    0.018    5.592    0.000    0.098    0.461
##  .s21_soz.6 ~~                                                          
##    .s21_s.7           0.122    0.019    6.275    0.000    0.122    0.575
##   djc2 ~~                                                               
##     djc3             -0.025    0.011   -2.282    0.022   -0.290   -0.290
##     djc4             -0.011    0.012   -0.967    0.334   -0.115   -0.115
##   djc3 ~~                                                               
##     djc4             -0.040    0.012   -3.280    0.001   -0.470   -0.470
##  .s200_de.3 ~~                                                          
##    .s200_.3           0.146    0.025    5.943    0.000    0.146    0.276
##  .s200_de.4 ~~                                                          
##    .s200_.4           0.127    0.021    6.132    0.000    0.127    0.253
##  .s200_de.6 ~~                                                          
##    .s200_.6           0.153    0.023    6.526    0.000    0.153    0.338
##  .s200_de.7 ~~                                                          
##    .s200_.7           0.200    0.028    7.208    0.000    0.200    0.425
##  .s200_de.3 ~~                                                          
##    .s200_.4           0.281    0.031    9.080    0.000    0.281    0.470
##    .s200_.6           0.232    0.030    7.746    0.000    0.232    0.412
##    .s200_.7           0.252    0.033    7.691    0.000    0.252    0.425
##  .s200_de.4 ~~                                                          
##    .s200_.6           0.192    0.027    7.041    0.000    0.192    0.354
##    .s200_.7           0.225    0.030    7.484    0.000    0.225    0.392
##  .s200_de.6 ~~                                                          
##    .s200_.7           0.227    0.029    7.739    0.000    0.227    0.421
##  .s200_re.3 ~~                                                          
##    .s200_.4           0.118    0.022    5.395    0.000    0.118    0.265
##    .s200_.6           0.134    0.024    5.710    0.000    0.134    0.316
##    .s200_.7           0.116    0.022    5.190    0.000    0.116    0.275
##  .s200_re.4 ~~                                                          
##    .s200_.6           0.144    0.021    6.942    0.000    0.144    0.347
##    .s200_.7           0.109    0.021    5.271    0.000    0.109    0.266
##  .s200_re.6 ~~                                                          
##    .s200_.7           0.111    0.022    5.164    0.000    0.111    0.283
##  .s200_au.3 ~~                                                          
##    .s200_.4           0.100    0.017    5.950    0.000    0.100    0.393
##    .s200_.6           0.069    0.016    4.404    0.000    0.069    0.336
##    .s200_.7           0.052    0.017    2.975    0.003    0.052    0.243
##  .s200_au.4 ~~                                                          
##    .s200_.6           0.060    0.015    4.108    0.000    0.060    0.285
##    .s200_.7           0.053    0.016    3.221    0.001    0.053    0.241
##  .s200_au.6 ~~                                                          
##    .s200_.7           0.071    0.016    4.531    0.000    0.071    0.399
##  .s200_ma.3 ~~                                                          
##    .s200_.4           0.091    0.020    4.536    0.000    0.091    0.291
##    .s200_.6           0.096    0.020    4.750    0.000    0.096    0.344
##    .s200_.7           0.057    0.022    2.618    0.009    0.057    0.200
##  .s200_ma.4 ~~                                                          
##    .s200_.6           0.067    0.016    4.159    0.000    0.067    0.281
##    .s200_.7           0.071    0.018    3.937    0.000    0.071    0.292
##  .s200_ma.6 ~~                                                          
##    .s200_.7           0.083    0.018    4.736    0.000    0.083    0.388
##  .s200_me.3 ~~                                                          
##    .s200_.4           0.065    0.016    4.149    0.000    0.065    0.266
##    .s200_.6           0.059    0.018    3.346    0.001    0.059    0.254
##    .s200_.7           0.078    0.017    4.568    0.000    0.078    0.362
##  .s200_me.4 ~~                                                          
##    .s200_.6           0.076    0.016    4.753    0.000    0.076    0.317
##    .s200_.7           0.064    0.017    3.826    0.000    0.064    0.290
##  .s200_me.6 ~~                                                          
##    .s200_.7           0.070    0.018    3.989    0.000    0.070    0.335
##  .s200_af.3 ~~                                                          
##    .s200_.4           0.164    0.026    6.333    0.000    0.164    0.354
##    .s200_.6           0.166    0.028    5.928    0.000    0.166    0.390
##    .s200_.7           0.177    0.025    6.971    0.000    0.177    0.465
##  .s200_af.4 ~~                                                          
##    .s200_.6           0.195    0.027    7.154    0.000    0.195    0.400
##    .s200_.7           0.193    0.028    6.997    0.000    0.193    0.443
##  .s200_af.6 ~~                                                          
##    .s200_.7           0.196    0.027    7.148    0.000    0.196    0.488
##   dojc2 ~~                                                              
##     dojc3            -0.094    0.014   -6.661    0.000   -0.567   -0.567
##     dojc4             0.005    0.012    0.391    0.695    0.031    0.031
##   dojc3 ~~                                                              
##     dojc4            -0.061    0.013   -4.850    0.000   -0.420   -0.420
##   djc2 ~~                                                               
##     dojc2   (cov1)    0.024    0.006    3.871    0.000    0.182    0.182
##   djc3 ~~                                                               
##     dojc3   (cov1)    0.024    0.006    3.871    0.000    0.221    0.221
##   djc4 ~~                                                               
##     dojc4   (cov1)    0.024    0.006    3.871    0.000    0.213    0.213
##   JC_W1 ~~                                                              
##     c137.6           -0.019    0.015   -1.201    0.230   -0.034   -0.075
##   OJC_W1 ~~                                                             
##     c137.6            0.029    0.011    2.548    0.011    0.068    0.150
##   SRH_W1 ~~                                                             
##     c137.6           -0.095    0.019   -5.124    0.000   -0.146   -0.323
##   djc2 ~~                                                               
##     c137.6            0.004    0.011    0.398    0.691    0.014    0.030
##   djc3 ~~                                                               
##     c137.6           -0.016    0.010   -1.663    0.096   -0.059   -0.129
##   djc4 ~~                                                               
##     c137.6            0.005    0.012    0.416    0.678    0.016    0.035
##   dojc2 ~~                                                              
##     c137.6           -0.002    0.011   -0.215    0.830   -0.006   -0.013
##   dojc3 ~~                                                              
##     c137.6           -0.013    0.011   -1.188    0.235   -0.032   -0.070
##   dojc4 ~~                                                              
##     c137.6            0.008    0.011    0.760    0.447    0.023    0.050
##   JC_W1 ~~                                                              
##     djc2             -0.036    0.015   -2.366    0.018   -0.207   -0.207
##     djc3             -0.024    0.014   -1.694    0.090   -0.163   -0.163
##     djc4              0.008    0.017    0.452    0.651    0.045    0.045
##     OJC_W1            0.076    0.015    5.012    0.000    0.327    0.327
##     dojc2             0.024    0.014    1.790    0.073    0.109    0.109
##     dojc3            -0.020    0.014   -1.427    0.154   -0.094   -0.094
##     dojc4            -0.004    0.015   -0.254    0.800   -0.020   -0.020
##     SRH_W1            0.066    0.021    3.194    0.001    0.187    0.187
##   djc2 ~~                                                               
##     OJC_W1           -0.028    0.010   -2.898    0.004   -0.205   -0.205
##     dojc3            -0.000    0.010   -0.011    0.991   -0.001   -0.001
##     dojc4            -0.004    0.010   -0.420    0.675   -0.038   -0.038
##     SRH_W1           -0.016    0.016   -1.028    0.304   -0.078   -0.078
##   djc3 ~~                                                               
##     OJC_W1           -0.003    0.010   -0.265    0.791   -0.023   -0.023
##     dojc2            -0.001    0.010   -0.098    0.922   -0.008   -0.008
##     dojc4            -0.011    0.009   -1.192    0.233   -0.112   -0.112
##     SRH_W1            0.027    0.015    1.846    0.065    0.155    0.155
##   djc4 ~~                                                               
##     OJC_W1           -0.004    0.012   -0.307    0.759   -0.028   -0.028
##     dojc2             0.008    0.012    0.645    0.519    0.061    0.061
##     dojc3            -0.021    0.010   -2.031    0.042   -0.171   -0.171
##     SRH_W1           -0.015    0.017   -0.844    0.399   -0.073   -0.073
##   OJC_W1 ~~                                                             
##     dojc2            -0.050    0.012   -4.263    0.000   -0.283   -0.283
##     dojc3            -0.008    0.011   -0.759    0.448   -0.048   -0.048
##     dojc4            -0.001    0.011   -0.133    0.894   -0.010   -0.010
##     SRH_W1            0.076    0.016    4.781    0.000    0.274    0.274
##   dojc2 ~~                                                              
##     SRH_W1            0.013    0.016    0.814    0.416    0.049    0.049
##   dojc3 ~~                                                              
##     SRH_W1            0.009    0.016    0.572    0.568    0.035    0.035
##   dojc4 ~~                                                              
##     SRH_W1           -0.032    0.017   -1.962    0.050   -0.138   -0.138
## 
## Intercepts:
##                    Estimate  Std.Err  z-value  P(>|z|)   Std.lv  Std.all
##    .s        (jc6)    2.017    0.107   18.835    0.000    2.017    3.147
##    .s        (jc6)    2.017    0.107   18.835    0.000    2.017    3.124
##    .s        (jc6)    2.017    0.107   18.835    0.000    2.017    2.957
##    .s        (jc6)    2.017    0.107   18.835    0.000    2.017    2.927
##     J                 2.465    0.035   71.286    0.000    4.536    4.536
##    .J                 0.000                               0.000    0.000
##    .J                 0.000                               0.000    0.000
##    .J                 0.000                               0.000    0.000
##    .s                 0.000                               0.000    0.000
##    .s                 0.000                               0.000    0.000
##    .s                 0.000                               0.000    0.000
##    .s                 0.000                               0.000    0.000
##    .s       (ojc6)   -0.050    0.161   -0.312    0.755   -0.050   -0.063
##    .s       (ojc6)   -0.050    0.161   -0.312    0.755   -0.050   -0.061
##    .s       (ojc6)   -0.050    0.161   -0.312    0.755   -0.050   -0.065
##    .s       (ojc6)   -0.050    0.161   -0.312    0.755   -0.050   -0.065
##    .s       (ojc7)   -1.438    0.230   -6.265    0.000   -1.438   -1.885
##    .s       (ojc7)   -1.438    0.230   -6.265    0.000   -1.438   -1.690
##    .s       (ojc7)   -1.438    0.230   -6.265    0.000   -1.438   -1.932
##    .s       (ojc7)   -1.438    0.230   -6.265    0.000   -1.438   -1.862
##    .s       (ojc8)   -2.015    0.252   -7.996    0.000   -2.015   -2.364
##    .s       (ojc8)   -2.015    0.252   -7.996    0.000   -2.015   -2.296
##    .s       (ojc8)   -2.015    0.252   -7.996    0.000   -2.015   -2.547
##    .s       (ojc8)   -2.015    0.252   -7.996    0.000   -2.015   -2.471
##    .s       (ojc9)   -1.503    0.240   -6.250    0.000   -1.503   -2.020
##    .s       (ojc9)   -1.503    0.240   -6.250    0.000   -1.503   -1.815
##    .s       (ojc9)   -1.503    0.240   -6.250    0.000   -1.503   -1.963
##    .s       (ojc9)   -1.503    0.240   -6.250    0.000   -1.503   -1.971
##    .s       (oj10)   -0.962    0.240   -4.010    0.000   -0.962   -1.177
##    .s       (oj10)   -0.962    0.240   -4.010    0.000   -0.962   -1.016
##    .s       (oj10)   -0.962    0.240   -4.010    0.000   -0.962   -1.111
##    .s       (oj10)   -0.962    0.240   -4.010    0.000   -0.962   -1.164
##     O                 3.942    0.030  133.329    0.000    9.199    9.199
##    .O                 0.000                               0.000    0.000
##    .O                 0.000                               0.000    0.000
##    .O                 0.000                               0.000    0.000
##    .s                 0.000                               0.000    0.000
##    .s                 0.000                               0.000    0.000
##    .s                 0.000                               0.000    0.000
##    .s                 0.000                               0.000    0.000
##     d  (gamma_jc2)   -0.069    0.022   -3.181    0.001   -0.214   -0.214
##     d  (gamma_jc3)   -0.032    0.022   -1.468    0.142   -0.116   -0.116
##     d  (gamma_jc4)   -0.131    0.026   -4.980    0.000   -0.420   -0.420
##     d (gamma_ojc2)   -0.064    0.019   -3.396    0.001   -0.156   -0.156
##     d (gamma_ojc3)   -0.093    0.020   -4.703    0.000   -0.233   -0.233
##     d (gamma_ojc4)    0.031    0.021    1.493    0.135    0.086    0.086
##    .s                 0.000                               0.000    0.000
##    .s                 0.000                               0.000    0.000
##    .s                 0.000                               0.000    0.000
##    .s                 0.000                               0.000    0.000
##     S                 3.597    0.034  106.384    0.000    5.539    5.539
##    .S                 0.293    0.194    1.510    0.131    0.455    0.455
##    .S                 0.244    0.186    1.309    0.191    0.374    0.374
##    .S                 0.119    0.214    0.556    0.578    0.180    0.180
##    .s                 1.952    0.165   11.836    0.000    1.952    2.744
##    .s                 0.584    0.131    4.445    0.000    0.584    0.954
##    .s                 1.630    0.132   12.342    0.000    1.630    2.452
##    .s                 0.558    0.127    4.381    0.000    0.558    0.870
##    .s                 1.611    0.161    9.980    0.000    1.611    2.311
##    .s                 0.618    0.126    4.885    0.000    0.618    0.985
##    .s                 1.580    0.179    8.837    0.000    1.580    2.098
##    .s                 0.607    0.122    4.975    0.000    0.607    0.925
##     c                 0.287    0.023   12.377    0.000    0.287    0.632
## 
## Variances:
##                    Estimate  Std.Err  z-value  P(>|z|)   Std.lv  Std.all
##     JC_W1             0.295    0.036    8.163    0.000    1.000    1.000
##    .JC_W2             0.000                               0.000    0.000
##    .JC_W3             0.000                               0.000    0.000
##    .JC_W4             0.000                               0.000    0.000
##     djc2              0.103    0.016    6.334    0.000    1.000    1.000
##     djc3              0.074    0.013    5.555    0.000    1.000    1.000
##     djc4              0.097    0.019    5.188    0.000    1.000    1.000
##     OJC_W1            0.184    0.020    9.006    0.000    1.000    1.000
##    .OJC_W2            0.000                               0.000    0.000
##    .OJC_W3            0.000                               0.000    0.000
##    .OJC_W4            0.000                               0.000    0.000
##     dojc2             0.170    0.020    8.574    0.000    1.000    1.000
##     dojc3             0.160    0.019    8.341    0.000    1.000    1.000
##     dojc4             0.132    0.018    7.404    0.000    1.000    1.000
##    .s38.3r  (rs_s)    0.152    0.012   12.641    0.000    0.152    0.265
##    .s38.4r  (rs_s)    0.152    0.012   12.641    0.000    0.152    0.269
##    .s38.6   (rs_s)    0.152    0.012   12.641    0.000    0.152    0.264
##    .s38.7   (rs_s)    0.152    0.012   12.641    0.000    0.152    0.260
##    .s21_c.3           0.460    0.039   11.717    0.000    0.460    0.609
##    .s21_s.3           0.261    0.022   12.077    0.000    0.261    0.637
##    .s21_r.3           0.459    0.032   14.278    0.000    0.459    0.908
##    .s21_s.3           0.182    0.019    9.380    0.000    0.182    0.485
##    .s21_c.4           0.383    0.031   12.336    0.000    0.383    0.540
##    .s21_s.4           0.252    0.019   13.613    0.000    0.252    0.605
##    .s21_r.4           0.371    0.023   16.044    0.000    0.371    0.838
##    .s21_s.4           0.198    0.018   10.866    0.000    0.198    0.482
##    .s21_c.6           0.483    0.043   11.120    0.000    0.483    0.616
##    .s21_s.6           0.313    0.025   12.381    0.000    0.313    0.673
##    .s21_r.6           0.400    0.029   13.591    0.000    0.400    0.822
##    .s21_s.6           0.196    0.021    9.444    0.000    0.196    0.499
##    .s21_c.7           0.389    0.042    9.345    0.000    0.389    0.556
##    .s21_s.7           0.318    0.028   11.423    0.000    0.318    0.669
##    .s21_r.7           0.471    0.039   12.104    0.000    0.471    0.830
##    .s21_s.7           0.228    0.026    8.921    0.000    0.228    0.529
##    .s200_.3           0.621    0.042   14.913    0.000    0.621    0.772
##    .s200_.3           0.455    0.032   14.239    0.000    0.455    0.720
##    .s200_.3           0.247    0.022   10.990    0.000    0.247    0.424
##    .s200_.3           0.365    0.030   11.964    0.000    0.365    0.502
##    .s200_.3           0.240    0.022   11.018    0.000    0.240    0.434
##    .s200_.3           0.405    0.032   12.732    0.000    0.405    0.605
##    .s200_.4           0.577    0.035   16.687    0.000    0.577    0.695
##    .s200_.4           0.434    0.027   16.229    0.000    0.434    0.640
##    .s200_.4           0.261    0.021   12.721    0.000    0.261    0.361
##    .s200_.4           0.271    0.021   12.637    0.000    0.271    0.351
##    .s200_.4           0.253    0.020   12.790    0.000    0.253    0.369
##    .s200_.4           0.532    0.035   15.324    0.000    0.532    0.593
##    .s200_.6           0.513    0.036   14.417    0.000    0.513    0.709
##    .s200_.6           0.398    0.029   13.817    0.000    0.398    0.663
##    .s200_.6           0.171    0.018    9.769    0.000    0.171    0.309
##    .s200_.6           0.212    0.020   10.438    0.000    0.212    0.338
##    .s200_.6           0.227    0.021   10.739    0.000    0.227    0.388
##    .s200_.6           0.448    0.036   12.614    0.000    0.448    0.597
##    .s200_.7           0.569    0.042   13.398    0.000    0.569    0.715
##    .s200_.7           0.389    0.031   12.378    0.000    0.389    0.641
##    .s200_.7           0.184    0.021    8.869    0.000    0.184    0.308
##    .s200_.7           0.219    0.023    9.364    0.000    0.219    0.329
##    .s200_.7           0.195    0.021    9.172    0.000    0.195    0.336
##    .s200_.7           0.358    0.032   11.070    0.000    0.358    0.524
##     c137.6            0.206    0.015   13.372    0.000    0.206    1.000
##     SRH_W1            0.422    0.037   11.405    0.000    1.000    1.000
##    .SRH_W2            0.020    0.022    0.892    0.372    0.047    0.047
##    .SRH_W3            0.038    0.019    1.967    0.049    0.089    0.089
##    .SRH_W4            0.079    0.028    2.769    0.006    0.181    0.181
## 
## 
## Group 2 [contractual reduction]:
## 
## Latent Variables:
##                    Estimate  Std.Err  z-value  P(>|z|)   Std.lv  Std.all
##   JC_W1 =~                                                              
##     s21_c.3           1.000                               0.448    0.556
##     s21_s.3  (jc1)    0.711    0.043   16.357    0.000    0.319    0.534
##     s21_r.3           0.617    0.117    5.285    0.000    0.276    0.434
##     s21_s.3 (j3_2)    1.239    0.125    9.912    0.000    0.555    0.800
##   JC_W2 =~                                                              
##     s21_c.4           1.000                               0.464    0.561
##     s21_s.4  (jc1)    0.711    0.043   16.357    0.000    0.330    0.533
##     s21_r.4           0.420    0.102    4.115    0.000    0.195    0.305
##     s21_s.4 (j3_2)    1.239    0.125    9.912    0.000    0.575    0.849
##   JC_W3 =~                                                              
##     s21_c.6           1.000                               0.534    0.608
##     s21_s.6  (jc1)    0.711    0.043   16.357    0.000    0.380    0.621
##     s21_r.6           0.435    0.106    4.111    0.000    0.232    0.342
##     s21_s.6 (j3_2)    1.239    0.125    9.912    0.000    0.662    0.888
##   JC_W4 =~                                                              
##     s21_c.7           1.000                               0.492    0.619
##     s21_s.7  (jc1)    0.711    0.043   16.357    0.000    0.350    0.541
##     s21_r.7           0.391    0.123    3.186    0.001    0.192    0.289
##     s21_s.7 (j3_2)    1.239    0.125    9.912    0.000    0.610    0.929
##   djc2 =~                                                               
##     JC_W2             1.000                               0.619    0.619
##   djc3 =~                                                               
##     JC_W3             1.000                               0.637    0.637
##   djc4 =~                                                               
##     JC_W4             1.000                               0.821    0.821
##   OJC_W1 =~                                                             
##     s200_.3           1.000                               0.495    0.580
##     s200_.3 (ojc1)    0.982    0.041   23.774    0.000    0.486    0.567
##     s200_.3 (ojc2)    1.351    0.059   22.983    0.000    0.669    0.825
##     s200_.3 (ojc3)    1.404    0.065   21.745    0.000    0.695    0.785
##     s200_.3 (ojc4)    1.306    0.062   21.213    0.000    0.646    0.818
##     s200_.3 (ojc5)    1.200    0.061   19.535    0.000    0.594    0.686
##   OJC_W2 =~                                                             
##     s200_.4           1.000                               0.460    0.505
##     s200_.4 (ojc1)    0.982    0.041   23.774    0.000    0.452    0.591
##     s200_.4 (ojc2)    1.351    0.059   22.983    0.000    0.622    0.806
##     s200_.4 (ojc3)    1.404    0.065   21.745    0.000    0.646    0.794
##     s200_.4 (ojc4)    1.306    0.062   21.213    0.000    0.601    0.773
##     s200_.4 (ojc5)    1.200    0.061   19.535    0.000    0.552    0.615
##   OJC_W3 =~                                                             
##     s200_.6           1.000                               0.504    0.598
##     s200_.6 (ojc1)    0.982    0.041   23.774    0.000    0.495    0.632
##     s200_.6 (ojc2)    1.351    0.059   22.983    0.000    0.681    0.857
##     s200_.6 (ojc3)    1.404    0.065   21.745    0.000    0.708    0.867
##     s200_.6 (ojc4)    1.306    0.062   21.213    0.000    0.658    0.825
##     s200_.6 (ojc5)    1.200    0.061   19.535    0.000    0.605    0.669
##   OJC_W4 =~                                                             
##     s200_.7           1.000                               0.478    0.568
##     s200_.7 (ojc1)    0.982    0.041   23.774    0.000    0.469    0.618
##     s200_.7 (ojc2)    1.351    0.059   22.983    0.000    0.646    0.857
##     s200_.7 (ojc3)    1.404    0.065   21.745    0.000    0.671    0.841
##     s200_.7 (ojc4)    1.306    0.062   21.213    0.000    0.624    0.814
##     s200_.7 (ojc5)    1.200    0.061   19.535    0.000    0.573    0.752
##   dojc2 =~                                                              
##     OJC_W2            1.000                               0.931    0.931
##   dojc3 =~                                                              
##     OJC_W3            1.000                               0.927    0.927
##   dojc4 =~                                                              
##     OJC_W4            1.000                               0.800    0.800
##   SRH_W1 =~                                                             
##     s38.3r            1.000                               0.742    0.885
##   SRH_W2 =~                                                             
##     s38.4r            1.000                               0.728    0.881
##   SRH_W3 =~                                                             
##     s38.6             1.000                               0.716    0.878
##   SRH_W4 =~                                                             
##     s38.7             1.000                               0.670    0.864
## 
## Regressions:
##                    Estimate  Std.Err  z-value  P(>|z|)   Std.lv  Std.all
##   JC_W2 ~                                                               
##     JC_W1             1.000                               0.966    0.966
##   JC_W3 ~                                                               
##     JC_W2             1.000                               0.869    0.869
##   JC_W4 ~                                                               
##     JC_W3             1.000                               1.086    1.086
##   OJC_W2 ~                                                              
##     OJC_W1            1.000                               1.075    1.075
##   OJC_W3 ~                                                              
##     OJC_W2            1.000                               0.913    0.913
##   OJC_W4 ~                                                              
##     OJC_W3            1.000                               1.054    1.054
##   SRH_W2 ~                                                              
##     SRH_W1  (ar1_)    0.952    0.051   18.803    0.000    0.972    0.972
##   SRH_W3 ~                                                              
##     SRH_W2  (ar2_)    0.946    0.048   19.850    0.000    0.960    0.960
##   SRH_W4 ~                                                              
##     SRH_W3  (ar3_)    0.949    0.055   17.277    0.000    1.015    1.015
##   SRH_W2 ~                                                              
##     djc2    (jcs1)    0.253    0.098    2.587    0.010    0.100    0.100
##     dojc2   (ojc1)    0.094    0.061    1.540    0.123    0.055    0.055
##     c137.6  (cvd1)   -0.050    0.069   -0.724    0.469   -0.069   -0.031
##   SRH_W3 ~                                                              
##     djc3    (jcs2)    0.206    0.120    1.715    0.086    0.098    0.098
##     dojc3            -0.003    0.100   -0.028    0.978   -0.002   -0.002
##     c137.6  (cvd2)    0.006    0.065    0.097    0.923    0.009    0.004
##   SRH_W4 ~                                                              
##     djc4    (jcs3)    0.003    0.112    0.028    0.978    0.002    0.002
##     dojc4   (ojc3)    0.209    0.087    2.394    0.017    0.120    0.120
##     c137.6  (cvd3)    0.103    0.074    1.384    0.166    0.154    0.070
## 
## Covariances:
##                    Estimate  Std.Err  z-value  P(>|z|)   Std.lv  Std.all
##  .s21_rd.3 ~~                                                           
##    .s21_r.4           0.218    0.034    6.417    0.000    0.218    0.623
##    .s21_r.6           0.241    0.041    5.862    0.000    0.241    0.657
##    .s21_r.7           0.251    0.042    6.006    0.000    0.251    0.686
##  .s21_rd.4 ~~                                                           
##    .s21_r.6           0.240    0.040    5.956    0.000    0.240    0.617
##    .s21_r.7           0.252    0.042    5.978    0.000    0.252    0.650
##  .s21_rd.6 ~~                                                           
##    .s21_r.7           0.257    0.049    5.218    0.000    0.257    0.630
##  .s21_str.3 ~~                                                          
##    .s21_s.4           0.167    0.027    6.104    0.000    0.167    0.630
##    .s21_s.6           0.146    0.031    4.696    0.000    0.146    0.602
##    .s21_s.7           0.177    0.033    5.293    0.000    0.177    0.642
##  .s21_str.4 ~~                                                          
##    .s21_s.6           0.151    0.027    5.581    0.000    0.151    0.602
##    .s21_s.7           0.182    0.032    5.618    0.000    0.182    0.637
##  .s21_str.6 ~~                                                          
##    .s21_s.7           0.171    0.033    5.200    0.000    0.171    0.655
##  .s21_cd.3 ~~                                                           
##    .s21_c.4           0.249    0.046    5.444    0.000    0.249    0.542
##    .s21_c.6           0.285    0.053    5.338    0.000    0.285    0.609
##    .s21_c.7           0.276    0.051    5.397    0.000    0.276    0.662
##  .s21_cd.4 ~~                                                           
##    .s21_c.6           0.315    0.052    6.020    0.000    0.315    0.658
##    .s21_c.7           0.284    0.048    5.862    0.000    0.284    0.665
##  .s21_cd.6 ~~                                                           
##    .s21_c.7           0.297    0.053    5.610    0.000    0.297    0.681
##  .s21_soz.3 ~~                                                          
##    .s21_s.4           0.093    0.034    2.788    0.005    0.093    0.626
##    .s21_s.6           0.081    0.036    2.268    0.023    0.081    0.571
##    .s21_s.7           0.051    0.034    1.500    0.134    0.051    0.504
##  .s21_soz.4 ~~                                                          
##    .s21_s.6           0.054    0.033    1.621    0.105    0.054    0.442
##    .s21_s.7           0.025    0.034    0.732    0.464    0.025    0.286
##  .s21_soz.6 ~~                                                          
##    .s21_s.7           0.034    0.033    1.043    0.297    0.034    0.413
##   djc2 ~~                                                               
##     djc3             -0.041    0.020   -2.031    0.042   -0.421   -0.421
##     djc4              0.009    0.024    0.391    0.696    0.081    0.081
##   djc3 ~~                                                               
##     djc4             -0.094    0.028   -3.371    0.001   -0.686   -0.686
##  .s200_de.3 ~~                                                          
##    .s200_.3           0.102    0.040    2.568    0.010    0.102    0.209
##  .s200_de.4 ~~                                                          
##    .s200_.4           0.187    0.037    5.089    0.000    0.187    0.384
##  .s200_de.6 ~~                                                          
##    .s200_.6           0.136    0.037    3.682    0.000    0.136    0.333
##  .s200_de.7 ~~                                                          
##    .s200_.7           0.146    0.039    3.689    0.000    0.146    0.352
##  .s200_de.3 ~~                                                          
##    .s200_.4           0.236    0.046    5.083    0.000    0.236    0.432
##    .s200_.6           0.164    0.047    3.505    0.000    0.164    0.348
##    .s200_.7           0.169    0.048    3.497    0.000    0.169    0.350
##  .s200_de.4 ~~                                                          
##    .s200_.6           0.212    0.044    4.798    0.000    0.212    0.399
##    .s200_.7           0.155    0.046    3.407    0.001    0.155    0.285
##  .s200_de.6 ~~                                                          
##    .s200_.7           0.233    0.047    4.947    0.000    0.233    0.496
##  .s200_re.3 ~~                                                          
##    .s200_.4           0.159    0.038    4.150    0.000    0.159    0.365
##    .s200_.6           0.184    0.043    4.272    0.000    0.184    0.430
##    .s200_.7           0.208    0.043    4.822    0.000    0.208    0.494
##  .s200_re.4 ~~                                                          
##    .s200_.6           0.109    0.035    3.145    0.002    0.109    0.290
##    .s200_.7           0.154    0.034    4.522    0.000    0.154    0.418
##  .s200_re.6 ~~                                                          
##    .s200_.7           0.153    0.039    3.872    0.000    0.153    0.421
##  .s200_au.3 ~~                                                          
##    .s200_.4           0.071    0.026    2.761    0.006    0.071    0.338
##    .s200_.6           0.038    0.026    1.430    0.153    0.038    0.201
##    .s200_.7           0.035    0.028    1.276    0.202    0.035    0.198
##  .s200_au.4 ~~                                                          
##    .s200_.6           0.026    0.025    1.057    0.291    0.026    0.141
##    .s200_.7           0.061    0.025    2.398    0.016    0.061    0.342
##  .s200_au.6 ~~                                                          
##    .s200_.7           0.034    0.026    1.313    0.189    0.034    0.212
##  .s200_ma.3 ~~                                                          
##    .s200_.4           0.099    0.032    3.120    0.002    0.099    0.364
##    .s200_.6           0.072    0.032    2.290    0.022    0.072    0.324
##    .s200_.7           0.108    0.036    3.047    0.002    0.108    0.456
##  .s200_ma.4 ~~                                                          
##    .s200_.6           0.082    0.026    3.178    0.001    0.082    0.408
##    .s200_.7           0.074    0.028    2.622    0.009    0.074    0.345
##  .s200_ma.6 ~~                                                          
##    .s200_.7           0.056    0.026    2.129    0.033    0.056    0.320
##  .s200_me.3 ~~                                                          
##    .s200_.4           0.068    0.025    2.699    0.007    0.068    0.301
##    .s200_.6           0.071    0.027    2.638    0.008    0.071    0.346
##    .s200_.7           0.087    0.027    3.228    0.001    0.087    0.429
##  .s200_me.4 ~~                                                          
##    .s200_.6           0.030    0.027    1.120    0.263    0.030    0.135
##    .s200_.7           0.066    0.028    2.385    0.017    0.066    0.299
##  .s200_me.6 ~~                                                          
##    .s200_.7           0.081    0.029    2.794    0.005    0.081    0.406
##  .s200_af.3 ~~                                                          
##    .s200_.4           0.183    0.042    4.326    0.000    0.183    0.410
##    .s200_.6           0.234    0.050    4.708    0.000    0.234    0.552
##    .s200_.7           0.133    0.040    3.359    0.001    0.133    0.422
##  .s200_af.4 ~~                                                          
##    .s200_.6           0.240    0.050    4.837    0.000    0.240    0.504
##    .s200_.7           0.120    0.041    2.943    0.003    0.120    0.338
##  .s200_af.6 ~~                                                          
##    .s200_.7           0.212    0.043    4.930    0.000    0.212    0.628
##   dojc2 ~~                                                              
##     dojc3            -0.128    0.028   -4.585    0.000   -0.638   -0.638
##     dojc4             0.016    0.025    0.650    0.516    0.101    0.101
##   dojc3 ~~                                                              
##     dojc4            -0.096    0.027   -3.537    0.000   -0.539   -0.539
##   djc2 ~~                                                               
##     dojc2   (cov2)   -0.016    0.015   -1.109    0.267   -0.133   -0.133
##   djc3 ~~                                                               
##     dojc3   (cov1)    0.024    0.006    3.871    0.000    0.151    0.151
##   djc4 ~~                                                               
##     dojc4   (cov1)    0.024    0.006    3.871    0.000    0.156    0.156
##   JC_W1 ~~                                                              
##     c137.6           -0.024    0.023   -1.038    0.299   -0.053   -0.117
##   OJC_W1 ~~                                                             
##     c137.6           -0.014    0.026   -0.550    0.583   -0.029   -0.063
##   SRH_W1 ~~                                                             
##     c137.6           -0.153    0.037   -4.102    0.000   -0.206   -0.452
##   djc2 ~~                                                               
##     c137.6           -0.002    0.017   -0.138    0.890   -0.008   -0.018
##   djc3 ~~                                                               
##     c137.6           -0.038    0.020   -1.918    0.055   -0.111   -0.244
##   djc4 ~~                                                               
##     c137.6            0.006    0.023    0.253    0.801    0.014    0.032
##   dojc2 ~~                                                              
##     c137.6            0.002    0.022    0.105    0.916    0.006    0.012
##   dojc3 ~~                                                              
##     c137.6           -0.039    0.023   -1.690    0.091   -0.084   -0.185
##   dojc4 ~~                                                              
##     c137.6            0.028    0.021    1.313    0.189    0.073    0.161
##   JC_W1 ~~                                                              
##     djc2             -0.034    0.018   -1.848    0.065   -0.264   -0.264
##     djc3              0.018    0.024    0.754    0.451    0.119    0.119
##     djc4             -0.018    0.030   -0.618    0.536   -0.101   -0.101
##     OJC_W1            0.094    0.025    3.748    0.000    0.425    0.425
##     dojc2            -0.009    0.021   -0.428    0.669   -0.046   -0.046
##     dojc3            -0.036    0.025   -1.440    0.150   -0.173   -0.173
##     dojc4             0.014    0.025    0.556    0.578    0.082    0.082
##     SRH_W1            0.065    0.033    1.966    0.049    0.196    0.196
##   djc2 ~~                                                               
##     OJC_W1            0.010    0.016    0.639    0.523    0.074    0.074
##     dojc3             0.040    0.018    2.268    0.023    0.299    0.299
##     dojc4            -0.007    0.019   -0.366    0.714   -0.063   -0.063
##     SRH_W1           -0.019    0.024   -0.767    0.443   -0.088   -0.088
##   djc3 ~~                                                               
##     OJC_W1            0.013    0.026    0.496    0.620    0.077    0.077
##     dojc2             0.011    0.019    0.568    0.570    0.076    0.076
##     dojc4            -0.031    0.016   -1.941    0.052   -0.239   -0.239
##     SRH_W1            0.101    0.034    2.940    0.003    0.399    0.399
##   djc4 ~~                                                               
##     OJC_W1           -0.011    0.032   -0.344    0.731   -0.055   -0.055
##     dojc2            -0.034    0.028   -1.246    0.213   -0.199   -0.199
##     dojc3            -0.021    0.020   -1.062    0.288   -0.113   -0.113
##     SRH_W1           -0.081    0.040   -2.037    0.042   -0.271   -0.271
##   OJC_W1 ~~                                                             
##     dojc2            -0.108    0.025   -4.263    0.000   -0.511   -0.511
##     dojc3             0.040    0.027    1.443    0.149    0.171    0.171
##     dojc4            -0.006    0.029   -0.214    0.831   -0.032   -0.032
##     SRH_W1            0.140    0.036    3.926    0.000    0.381    0.381
##   dojc2 ~~                                                              
##     SRH_W1           -0.005    0.031   -0.147    0.883   -0.014   -0.014
##   dojc3 ~~                                                              
##     SRH_W1           -0.000    0.037   -0.010    0.992   -0.001   -0.001
##   dojc4 ~~                                                              
##     SRH_W1            0.023    0.036    0.621    0.535    0.080    0.080
## 
## Intercepts:
##                    Estimate  Std.Err  z-value  P(>|z|)   Std.lv  Std.all
##    .s        (jc6)    2.017    0.107   18.835    0.000    2.017    3.377
##    .s        (jc6)    2.017    0.107   18.835    0.000    2.017    3.256
##    .s        (jc6)    2.017    0.107   18.835    0.000    2.017    3.297
##    .s        (jc6)    2.017    0.107   18.835    0.000    2.017    3.117
##     J                 2.545    0.050   51.051    0.000    5.678    5.678
##    .J                 0.000                               0.000    0.000
##    .J                 0.000                               0.000    0.000
##    .J                 0.000                               0.000    0.000
##    .s                 0.000                               0.000    0.000
##    .s                 0.000                               0.000    0.000
##    .s                 0.000                               0.000    0.000
##    .s                 0.000                               0.000    0.000
##    .s       (ojc6)   -0.050    0.161   -0.312    0.755   -0.050   -0.059
##    .s       (ojc6)   -0.050    0.161   -0.312    0.755   -0.050   -0.066
##    .s       (ojc6)   -0.050    0.161   -0.312    0.755   -0.050   -0.064
##    .s       (ojc6)   -0.050    0.161   -0.312    0.755   -0.050   -0.066
##    .s       (ojc7)   -1.438    0.230   -6.265    0.000   -1.438   -1.774
##    .s       (ojc7)   -1.438    0.230   -6.265    0.000   -1.438   -1.865
##    .s       (ojc7)   -1.438    0.230   -6.265    0.000   -1.438   -1.810
##    .s       (ojc7)   -1.438    0.230   -6.265    0.000   -1.438   -1.909
##    .s       (ojc8)   -2.015    0.252   -7.996    0.000   -2.015   -2.274
##    .s       (ojc8)   -2.015    0.252   -7.996    0.000   -2.015   -2.476
##    .s       (ojc8)   -2.015    0.252   -7.996    0.000   -2.015   -2.467
##    .s       (ojc8)   -2.015    0.252   -7.996    0.000   -2.015   -2.523
##    .s       (ojc9)   -1.503    0.240   -6.250    0.000   -1.503   -1.901
##    .s       (ojc9)   -1.503    0.240   -6.250    0.000   -1.503   -1.931
##    .s       (ojc9)   -1.503    0.240   -6.250    0.000   -1.503   -1.883
##    .s       (ojc9)   -1.503    0.240   -6.250    0.000   -1.503   -1.959
##    .s       (oj10)   -0.962    0.240   -4.010    0.000   -0.962   -1.112
##    .s       (oj10)   -0.962    0.240   -4.010    0.000   -0.962   -1.071
##    .s       (oj10)   -0.962    0.240   -4.010    0.000   -0.962   -1.064
##    .s       (oj10)   -0.962    0.240   -4.010    0.000   -0.962   -1.262
##     O                 3.968    0.041   96.957    0.000    8.018    8.018
##    .O                 0.000                               0.000    0.000
##    .O                 0.000                               0.000    0.000
##    .O                 0.000                               0.000    0.000
##    .s                 0.000                               0.000    0.000
##    .s                 0.000                               0.000    0.000
##    .s                 0.000                               0.000    0.000
##    .s                 0.000                               0.000    0.000
##     d  (gamma_jc2)   -0.069    0.022   -3.181    0.001   -0.239   -0.239
##     d  (gamma_jc3)   -0.032    0.022   -1.468    0.142   -0.093   -0.093
##     d  (gamma_jc4)   -0.131    0.026   -4.980    0.000   -0.323   -0.323
##     d (gamma_ojc2)   -0.064    0.019   -3.396    0.001   -0.150   -0.150
##     d (gamma_ojc3)   -0.093    0.020   -4.703    0.000   -0.200   -0.200
##     d (gamma_ojc4)    0.031    0.021    1.493    0.135    0.081    0.081
##    .s                 0.000                               0.000    0.000
##    .s                 0.000                               0.000    0.000
##    .s                 0.000                               0.000    0.000
##    .s                 0.000                               0.000    0.000
##     S                 3.601    0.064   55.975    0.000    4.850    4.850
##    .S                 0.215    0.197    1.091    0.275    0.295    0.295
##    .S                 0.248    0.188    1.315    0.189    0.346    0.346
##    .S                 0.113    0.216    0.524    0.600    0.169    0.169
##    .s                 1.406    0.300    4.695    0.000    1.406    2.207
##    .s                -0.471    0.323   -1.458    0.145   -0.471   -0.679
##    .s                 1.902    0.258    7.381    0.000    1.902    2.975
##    .s                -0.498    0.314   -1.587    0.113   -0.498   -0.735
##    .s                 1.940    0.267    7.261    0.000    1.940    2.854
##    .s                -0.422    0.312   -1.352    0.176   -0.422   -0.566
##    .s                 1.958    0.288    6.793    0.000    1.958    2.937
##    .s                -0.408    0.294   -1.386    0.166   -0.408   -0.621
##     c                 0.293    0.042    7.013    0.000    0.293    0.643
## 
## Variances:
##                    Estimate  Std.Err  z-value  P(>|z|)   Std.lv  Std.all
##     JC_W1             0.201    0.042    4.817    0.000    1.000    1.000
##    .JC_W2             0.000                               0.000    0.000
##    .JC_W3             0.000                               0.000    0.000
##    .JC_W4             0.000                               0.000    0.000
##     djc2              0.082    0.021    3.959    0.000    1.000    1.000
##     djc3              0.116    0.029    4.065    0.000    1.000    1.000
##     djc4              0.163    0.038    4.276    0.000    1.000    1.000
##     OJC_W1            0.245    0.037    6.566    0.000    1.000    1.000
##    .OJC_W2            0.000                               0.000    0.000
##    .OJC_W3            0.000                               0.000    0.000
##    .OJC_W4            0.000                               0.000    0.000
##     dojc2             0.183    0.031    5.987    0.000    1.000    1.000
##     dojc3             0.218    0.038    5.701    0.000    1.000    1.000
##     dojc4             0.146    0.031    4.752    0.000    1.000    1.000
##    .s38.3r  (rs_s)    0.152    0.012   12.641    0.000    0.152    0.217
##    .s38.4r  (rs_s)    0.152    0.012   12.641    0.000    0.152    0.224
##    .s38.6   (rs_s)    0.152    0.012   12.641    0.000    0.152    0.229
##    .s38.7   (rs_s)    0.152    0.012   12.641    0.000    0.152    0.254
##    .s21_c.3           0.449    0.059    7.634    0.000    0.449    0.691
##    .s21_s.3           0.255    0.034    7.544    0.000    0.255    0.715
##    .s21_r.3           0.330    0.042    7.862    0.000    0.330    0.812
##    .s21_s.3           0.173    0.043    4.073    0.000    0.173    0.360
##    .s21_c.4           0.469    0.055    8.577    0.000    0.469    0.685
##    .s21_s.4           0.275    0.032    8.644    0.000    0.275    0.716
##    .s21_r.4           0.371    0.040    9.320    0.000    0.371    0.907
##    .s21_s.4           0.128    0.041    3.167    0.002    0.128    0.280
##    .s21_c.6           0.488    0.071    6.906    0.000    0.488    0.631
##    .s21_s.6           0.230    0.035    6.599    0.000    0.230    0.615
##    .s21_r.6           0.408    0.056    7.240    0.000    0.408    0.883
##    .s21_s.6           0.117    0.046    2.536    0.011    0.117    0.211
##    .s21_c.7           0.389    0.060    6.504    0.000    0.389    0.616
##    .s21_s.7           0.296    0.044    6.689    0.000    0.296    0.708
##    .s21_r.7           0.407    0.060    6.810    0.000    0.407    0.917
##    .s21_s.7           0.059    0.042    1.400    0.161    0.059    0.136
##    .s200_.3           0.483    0.057    8.457    0.000    0.483    0.663
##    .s200_.3           0.497    0.058    8.601    0.000    0.497    0.678
##    .s200_.3           0.210    0.035    6.052    0.000    0.210    0.320
##    .s200_.3           0.302    0.046    6.606    0.000    0.302    0.384
##    .s200_.3           0.207    0.033    6.344    0.000    0.207    0.331
##    .s200_.3           0.397    0.051    7.718    0.000    0.397    0.530
##    .s200_.4           0.619    0.063    9.865    0.000    0.619    0.745
##    .s200_.4           0.381    0.040    9.548    0.000    0.381    0.651
##    .s200_.4           0.208    0.030    6.968    0.000    0.208    0.350
##    .s200_.4           0.244    0.034    7.291    0.000    0.244    0.369
##    .s200_.4           0.244    0.032    7.531    0.000    0.244    0.403
##    .s200_.4           0.502    0.058    8.714    0.000    0.502    0.622
##    .s200_.6           0.457    0.057    8.038    0.000    0.457    0.643
##    .s200_.6           0.367    0.049    7.492    0.000    0.367    0.600
##    .s200_.6           0.168    0.032    5.266    0.000    0.168    0.266
##    .s200_.6           0.166    0.031    5.388    0.000    0.166    0.249
##    .s200_.6           0.203    0.035    5.844    0.000    0.203    0.319
##    .s200_.6           0.452    0.066    6.895    0.000    0.452    0.553
##    .s200_.7           0.481    0.064    7.505    0.000    0.481    0.678
##    .s200_.7           0.357    0.050    7.126    0.000    0.357    0.619
##    .s200_.7           0.151    0.030    4.996    0.000    0.151    0.265
##    .s200_.7           0.187    0.036    5.228    0.000    0.187    0.293
##    .s200_.7           0.198    0.035    5.588    0.000    0.198    0.337
##    .s200_.7           0.252    0.042    6.073    0.000    0.252    0.434
##     c137.6            0.207    0.029    7.199    0.000    0.207    1.000
##     SRH_W1            0.551    0.072    7.618    0.000    1.000    1.000
##    .SRH_W2            0.018    0.031    0.584    0.559    0.034    0.034
##    .SRH_W3            0.002    0.025    0.096    0.924    0.005    0.005
##    .SRH_W4            0.002    0.034    0.066    0.948    0.005    0.005
```

```
# Final model for GC4: lcsm_parcel_SRH_GC4_equalARc137COVW34JCOJCW24_timeequalCOV_equalCSJC123OJC123
```
