# Supplementary material for: Crafting for Health: A Longitudinal Study of Job and Off-Job Crafting Changes during the COVID-19 Pandemic
Source: Occup Health Sci. 2025 Feb 26;9(3):675–710. doi: 10.1007/s41542-025-00222-5 (PMC12484252; doi:10.1007/s41542-025-00222-5)
Supplement: Supplementary file 5 — Supplementary file5 (HTML 783 KB) [file 41542_2025_222_MOESM5_ESM.html]

Crafting during corona times: Additional Analyses regarding Care Duties


# Crafting during corona times: Additional Analyses regarding Care Duties

## Packages & Data

```
knitr::opts_chunk$set(warning = FALSE, message = FALSE) 

# Data handling
library(tidyverse)
```

```
## ── Attaching core tidyverse packages ──────────────────────── tidyverse 2.0.0 ──
## ✔ dplyr     1.1.2     ✔ readr     2.1.4
## ✔ forcats   1.0.0     ✔ stringr   1.5.0
## ✔ ggplot2   3.4.2     ✔ tibble    3.2.1
## ✔ lubridate 1.9.2     ✔ tidyr     1.3.0
## ✔ purrr     1.0.1     
## ── Conflicts ────────────────────────────────────────── tidyverse_conflicts() ──
## ✖ dplyr::filter() masks stats::filter()
## ✖ dplyr::lag()    masks stats::lag()
## ℹ Use the conflicted package (<http://conflicted.r-lib.org/>) to force all conflicts to become errors
```

```
library(readr)
library(lubridate)
library(sjlabelled)
```

```
## 
## Attaching package: 'sjlabelled'
## 
## The following object is masked from 'package:forcats':
## 
##     as_factor
## 
## The following object is masked from 'package:dplyr':
## 
##     as_label
## 
## The following object is masked from 'package:ggplot2':
## 
##     as_label
```

```
# Plots and Tables
library(ggplot2)
library(ggstar)
library(jtools)
library(apaTables)
```

```
## Registered S3 methods overwritten by 'broom':
##   method            from  
##   tidy.glht         jtools
##   tidy.summary.glht jtools
```

```
library(semTable)
library(semPlot)
# Data Analysis
library(psych)
```

```
## 
## Attaching package: 'psych'
## 
## The following objects are masked from 'package:ggplot2':
## 
##     %+%, alpha
```

```
library(lavaan)
```

```
## This is lavaan 0.6-16
## lavaan is FREE software! Please report any bugs.
## 
## Attaching package: 'lavaan'
## 
## The following object is masked from 'package:psych':
## 
##     cor2cov
```

```
library(lcsm)
```

```
## This is lcsm 0.3.2
## Please report any issues or ideas at:
## https://github.com/milanwiedemann/lcsm/issues
```

```
# For a funny beep sound when model estimation finished
library(beepr)

data <- readRDS("Paper 2 Data_wAddVariables.RDS") # Data available from first author upon reasonable request
```

## Subgroup analyses with non-converging model for care duties

```
# Longitudinal MI for each subgroup

# Testing MI using item parcels ----
### Job Crafting
## Create a tidy dataset
jc_gc <- select(data, 
                group1_workloc.4,
                group2_livingsit.4,
                group3_caredutieskids.4,
                group4_contractchanges.4,
                s21_rd.3, s21_rd.4, s21_rd.6, s21_rd.7, 
                s21_str.3, s21_str.4, s21_str.6, s21_str.7, 
                s21_cd.3, s21_cd.4, s21_cd.6, s21_cd.7, 
                s21_soz.3, s21_soz.4, s21_soz.6, s21_soz.7)

jc_gc_tidy <- pivot_longer(jc_gc,
                           cols = s21_rd.3:s21_soz.7,
                           names_to = c(".value", "time"),
                           names_pattern = "(.{6,7}).(.)")

## Model specification
model_jc <- '
JC =~ s21_rd + s21_str + s21_cd + s21_soz
'

### Off-Job Crafting
## Create a tidy dataset
ojc_gc <- select(data, 
                 group1_workloc.4,
                 group2_livingsit.4,
                 group3_caredutieskids.4,
                 group4_contractchanges.4,
                 s200_de.3, s200_de.4, s200_de.6, s200_de.7,
                 s200_re.3, s200_re.4, s200_re.6, s200_re.7,
                 s200_au.3, s200_au.4, s200_au.6, s200_au.7,
                 s200_ma.3, s200_ma.4, s200_ma.6, s200_ma.7,
                 s200_me.3, s200_me.4, s200_me.6, s200_me.7,
                 s200_af.3, s200_af.4, s200_af.6, s200_af.7
                 ) 

ojc_gc_tidy <- pivot_longer(ojc_gc,
                            cols = s200_de.3:s200_af.7,
                            names_to = c(".value", "time"),
                            names_pattern = "(.{7}).(.)")

ojc_gc_tidy <- remove_all_labels(ojc_gc_tidy)

## Model specification
model_ojc <- '
OJC =~ s200_de + s200_re + s200_au + s200_ma + s200_me + s200_af
s200_de ~~ s200_re # allowing for a residual covariance here
'

## For Group Comparison 3 (Care duties) ----
tmp_jc <- jc_gc_tidy %>%
  filter(!(is.na(group3_caredutieskids.4))) %>%
  select(group3_caredutieskids.4, s21_rd:s21_soz, time) %>% 
  group_by(group3_caredutieskids.4) %>% 
  nest(data = c(s21_rd:s21_soz, time)) 

cfa_list <- list()

for (i in 1:length(tmp_jc$group3_caredutieskids.4)) {
  
  cfa_list[[1 + 3*(i-1)]] <- cfa(model = model_jc,
                                 data  = tmp_jc$data[i][[1]],
                                 estimator = "ML",
                                 missing = "ML",
                                 group = "time")
  
  cfa_list[[2 + 3*(i-1)]] <- cfa(model = model_jc,
                                 data  = tmp_jc$data[i][[1]],
                                 estimator = "ML",
                                 missing = "ML",
                                 group = "time",
                                 group.equal = c("loadings"))
  
  cfa_list[[3 + 3*(i-1)]] <- cfa(model = model_jc,
                                 data  = tmp_jc$data[i][[1]],
                                 estimator = "ML",
                                 missing = "ML",
                                 group = "time",
                                 group.equal = c("intercepts", "loadings"))
  
}

names(cfa_list) <- paste(rep(tmp_jc$group3_caredutieskids.4, each = 3), 
                         rep(c("_STR", "_MET", "_SCA"), times = 3), 
                         sep = "")

anova(cfa_list$`no kids_STR`, cfa_list$`no kids_MET`, cfa_list$`no kids_SCA`) # JC has even metric MI
```

```
## 
## Chi-Squared Difference Test
## 
##                        Df    AIC    BIC   Chisq Chisq diff    RMSEA Df diff
## cfa_list$`no kids_STR`  8 5779.0 5998.8  8.7595                            
## cfa_list$`no kids_MET` 17 5771.9 5950.5 19.7259     10.966 0.034840       9
## cfa_list$`no kids_SCA` 26 5773.5 5910.9 39.3107     19.585 0.080832       9
##                        Pr(>Chisq)  
## cfa_list$`no kids_STR`             
## cfa_list$`no kids_MET`    0.27802  
## cfa_list$`no kids_SCA`    0.02066 *
## ---
## Signif. codes:  0 '***' 0.001 '**' 0.01 '*' 0.05 '.' 0.1 ' ' 1
```

```
anova(cfa_list$`none at all_STR`, cfa_list$`none at all_MET`, cfa_list$`none at all_SCA`) # JC has even scalar MI
```

```
## 
## Chi-Squared Difference Test
## 
##                            Df    AIC    BIC   Chisq Chisq diff    RMSEA Df diff
## cfa_list$`none at all_STR`  8 4201.6 4407.1  4.5180                            
## cfa_list$`none at all_MET` 17 4187.1 4354.0  7.9569     3.4389 0.000000       9
## cfa_list$`none at all_SCA` 26 4182.2 4310.6 21.0529    13.0960 0.058387       9
##                            Pr(>Chisq)
## cfa_list$`none at all_STR`           
## cfa_list$`none at all_MET`     0.9443
## cfa_list$`none at all_SCA`     0.1583
```

```
anova(cfa_list$`1+_STR`, cfa_list$`1+_MET`, cfa_list$`1+_SCA`) # JC has even scalar MI
```

```
## 
## Chi-Squared Difference Test
## 
##                   Df    AIC    BIC  Chisq Chisq diff    RMSEA Df diff
## cfa_list$`1+_STR`  8 2295.1 2469.5 18.690                            
## cfa_list$`1+_MET` 17 2283.1 2424.8 24.730     6.0395 0.000000       9
## cfa_list$`1+_SCA` 26 2276.1 2385.1 35.736    11.0062 0.056431       9
##                   Pr(>Chisq)
## cfa_list$`1+_STR`           
## cfa_list$`1+_MET`     0.7360
## cfa_list$`1+_SCA`     0.2753
```

```
tmp_ojc <- ojc_gc_tidy %>%
  mutate(NAs = rowSums(is.na(ojc_gc_tidy[, 6:10]), na.rm = TRUE)) %>%
  filter(NAs < 6 & !(is.na(group3_caredutieskids.4))) %>%
  select(group3_caredutieskids.4, s200_de:s200_af, time) %>% 
  group_by(group3_caredutieskids.4) %>% 
  nest(data = c(s200_de:s200_af, time)) 

cfa_list <- list()

for (i in 1:length(tmp_ojc$group3_caredutieskids.4)) {
    
    cfa_list[[1 + 3*(i-1)]] <- cfa(model = model_ojc,
                         data  = tmp_ojc$data[i][[1]],
                         estimator = "ML",
                         missing = "ML",
                         group = "time")
    
    cfa_list[[2 + 3*(i-1)]] <- cfa(model = model_ojc,
                                  data  = tmp_ojc$data[i][[1]],
                                  estimator = "ML",
                                  missing = "ML",
                                  group = "time",
                                  group.equal = c("loadings"))
    
    cfa_list[[3 + 3*(i-1)]] <- cfa(model = model_ojc,
                                  data  = tmp_ojc$data[i][[1]],
                                  estimator = "ML",
                                  missing = "ML",
                                  group = "time",
                                  group.equal = c("intercepts", "loadings"))
    
}

names(cfa_list) <- paste(rep(tmp_ojc$group3_caredutieskids.4, each = 3), 
                         rep(c("_STR", "_MET", "_SCA"), times = 3), 
                         sep = "")

anova(cfa_list$`no kids_STR`, cfa_list$`no kids_MET`, cfa_list$`no kids_SCA`) # OJC has even scalar MI
```

```
## 
## Chi-Squared Difference Test
## 
##                        Df    AIC    BIC  Chisq Chisq diff    RMSEA Df diff
## cfa_list$`no kids_STR` 32 8629.8 8977.6 224.27                            
## cfa_list$`no kids_MET` 47 8615.7 8894.9 240.19    15.9194 0.018479      15
## cfa_list$`no kids_SCA` 62 8592.2 8802.8 246.69     6.5022 0.000000      15
##                        Pr(>Chisq)
## cfa_list$`no kids_STR`           
## cfa_list$`no kids_MET`     0.3874
## cfa_list$`no kids_SCA`     0.9700
```

```
anova(cfa_list$`none at all_STR`, cfa_list$`none at all_MET`, cfa_list$`none at all_SCA`) # OJC has metric MI
```

```
## 
## Chi-Squared Difference Test
## 
##                            Df    AIC    BIC   Chisq Chisq diff    RMSEA Df diff
## cfa_list$`none at all_STR` 32 6483.7 6808.9  95.984                            
## cfa_list$`none at all_MET` 47 6463.0 6723.9 105.250     9.2663 0.000000      15
## cfa_list$`none at all_SCA` 62 6460.3 6657.2 132.644    27.3944 0.078747      15
##                            Pr(>Chisq)  
## cfa_list$`none at all_STR`             
## cfa_list$`none at all_MET`    0.86319  
## cfa_list$`none at all_SCA`    0.02568 *
## ---
## Signif. codes:  0 '***' 0.001 '**' 0.01 '*' 0.05 '.' 0.1 ' ' 1
```

```
anova(cfa_list$`1+_STR`, cfa_list$`1+_MET`, cfa_list$`1+_SCA`) # OJC has even scalar MI
```

```
## 
## Chi-Squared Difference Test
## 
##                   Df    AIC    BIC   Chisq Chisq diff RMSEA Df diff Pr(>Chisq)
## cfa_list$`1+_STR` 32 3313.7 3590.0  99.795                                    
## cfa_list$`1+_MET` 47 3294.3 3516.0 110.388    10.5922     0      15     0.7809
## cfa_list$`1+_SCA` 62 3273.6 3440.8 119.731     9.3434     0      15     0.8589
```

```
# Intergroup MI at Wave 4

# Testing measurement invariance with item parcels ---- 
## Wave 4 ---- 
## Job crafting
model_jc_w <- '
JC =~ s21_rd.4 + s21_str.4 + s21_cd.4 + s21_soz.4
' 

fit1 <- cfa(model = model_jc_w,
    data  = data,
    estimator = "ML",
    missing = "ML",
    group = "group3_caredutieskids.4")

fit2 <- cfa(model = model_jc_w,
    data  = data,
    estimator = "ML",
    missing = "ML",
    group = "group3_caredutieskids.4",
    group.equal = c("loadings"))

fit3 <- cfa(model = model_jc_w,
    data  = data,
    estimator = "ML",
    missing = "ML",
    group = "group3_caredutieskids.4",
    group.equal = c("loadings", "intercepts"))

anova(fit1, fit2, fit3) # Metric MI does not hold for this time point
```

```
## 
## Chi-Squared Difference Test
## 
##      Df    AIC    BIC  Chisq Chisq diff   RMSEA Df diff Pr(>Chisq)    
## fit1  6 4235.4 4389.9 15.506                                          
## fit2 12 4247.4 4376.2 39.536    24.0302 0.12921       6  0.0005156 ***
## fit3 18 4237.7 4340.7 41.860     2.3238 0.00000       6  0.8876408    
## ---
## Signif. codes:  0 '***' 0.001 '**' 0.01 '*' 0.05 '.' 0.1 ' ' 1
```

```
summary(fit1, fit.measures = TRUE)
```

```
## lavaan 0.6.16 ended normally after 68 iterations
## 
##   Estimator                                         ML
##   Optimization method                           NLMINB
##   Number of model parameters                        36
## 
##   Number of observations per group:                   
##     no kids                                        246
##     none at all                                    189
##     1+                                             105
##   Number of missing patterns per group:               
##     no kids                                          1
##     none at all                                      1
##     1+                                               1
## 
## Model Test User Model:
##                                                       
##   Test statistic                                15.506
##   Degrees of freedom                                 6
##   P-value (Chi-square)                           0.017
##   Test statistic for each group:
##     no kids                                      2.708
##     none at all                                  0.760
##     1+                                          12.039
## 
## Model Test Baseline Model:
## 
##   Test statistic                               433.304
##   Degrees of freedom                                18
##   P-value                                        0.000
## 
## User Model versus Baseline Model:
## 
##   Comparative Fit Index (CFI)                    0.977
##   Tucker-Lewis Index (TLI)                       0.931
##                                                       
##   Robust Comparative Fit Index (CFI)             0.977
##   Robust Tucker-Lewis Index (TLI)                0.931
## 
## Loglikelihood and Information Criteria:
## 
##   Loglikelihood user model (H0)              -2081.693
##   Loglikelihood unrestricted model (H1)      -2073.940
##                                                       
##   Akaike (AIC)                                4235.387
##   Bayesian (BIC)                              4389.883
##   Sample-size adjusted Bayesian (SABIC)       4275.606
## 
## Root Mean Square Error of Approximation:
## 
##   RMSEA                                          0.094
##   90 Percent confidence interval - lower         0.037
##   90 Percent confidence interval - upper         0.153
##   P-value H_0: RMSEA <= 0.050                    0.091
##   P-value H_0: RMSEA >= 0.080                    0.702
##                                                       
##   Robust RMSEA                                   0.094
##   90 Percent confidence interval - lower         0.037
##   90 Percent confidence interval - upper         0.153
##   P-value H_0: Robust RMSEA <= 0.050             0.091
##   P-value H_0: Robust RMSEA >= 0.080             0.702
## 
## Standardized Root Mean Square Residual:
## 
##   SRMR                                           0.024
## 
## Parameter Estimates:
## 
##   Standard errors                             Standard
##   Information                                 Observed
##   Observed information based on                Hessian
## 
## 
## Group 1 [no kids]:
## 
## Latent Variables:
##                    Estimate  Std.Err  z-value  P(>|z|)
##   JC =~                                               
##     s21_rd.4          1.000                           
##     s21_str.4         1.495    0.280    5.345    0.000
##     s21_cd.4          2.037    0.379    5.381    0.000
##     s21_soz.4         2.126    0.406    5.232    0.000
## 
## Intercepts:
##                    Estimate  Std.Err  z-value  P(>|z|)
##    .s21_rd.4          2.796    0.044   64.189    0.000
##    .s21_str.4         3.759    0.042   89.327    0.000
##    .s21_cd.4          2.455    0.056   43.652    0.000
##    .s21_soz.4         2.511    0.043   58.685    0.000
##     JC                0.000                           
## 
## Variances:
##                    Estimate  Std.Err  z-value  P(>|z|)
##    .s21_rd.4          0.392    0.037   10.460    0.000
##    .s21_str.4         0.269    0.030    9.087    0.000
##    .s21_cd.4          0.469    0.053    8.916    0.000
##    .s21_soz.4         0.113    0.035    3.252    0.001
##     JC                0.075    0.026    2.870    0.004
## 
## 
## Group 2 [none at all]:
## 
## Latent Variables:
##                    Estimate  Std.Err  z-value  P(>|z|)
##   JC =~                                               
##     s21_rd.4          1.000                           
##     s21_str.4         2.437    0.977    2.495    0.013
##     s21_cd.4          5.206    2.096    2.484    0.013
##     s21_soz.4         2.667    1.053    2.534    0.011
## 
## Intercepts:
##                    Estimate  Std.Err  z-value  P(>|z|)
##    .s21_rd.4          2.796    0.049   57.440    0.000
##    .s21_str.4         3.739    0.044   84.790    0.000
##    .s21_cd.4          2.372    0.060   39.810    0.000
##    .s21_soz.4         2.451    0.045   54.713    0.000
##     JC                0.000                           
## 
## Variances:
##                    Estimate  Std.Err  z-value  P(>|z|)
##    .s21_rd.4          0.429    0.045    9.616    0.000
##    .s21_str.4         0.253    0.031    8.082    0.000
##    .s21_cd.4          0.150    0.082    1.835    0.067
##    .s21_soz.4         0.242    0.033    7.328    0.000
##     JC                0.019    0.015    1.293    0.196
## 
## 
## Group 3 [1+]:
## 
## Latent Variables:
##                    Estimate  Std.Err  z-value  P(>|z|)
##   JC =~                                               
##     s21_rd.4          1.000                           
##     s21_str.4         0.565    0.189    2.987    0.003
##     s21_cd.4          1.782    0.321    5.543    0.000
##     s21_soz.4         1.314    0.292    4.500    0.000
## 
## Intercepts:
##                    Estimate  Std.Err  z-value  P(>|z|)
##    .s21_rd.4          2.888    0.062   46.449    0.000
##    .s21_str.4         3.813    0.055   69.636    0.000
##    .s21_cd.4          2.632    0.086   30.686    0.000
##    .s21_soz.4         2.712    0.075   36.265    0.000
##     JC                0.000                           
## 
## Variances:
##                    Estimate  Std.Err  z-value  P(>|z|)
##    .s21_rd.4          0.249    0.043    5.807    0.000
##    .s21_str.4         0.265    0.040    6.553    0.000
##    .s21_cd.4          0.273    0.096    2.850    0.004
##    .s21_soz.4         0.316    0.070    4.512    0.000
##     JC                0.157    0.051    3.073    0.002
```

```
fit2_partial <- cfa(model = model_jc_w,
    data  = data,
    estimator = "ML",
    missing = "ML",
    group = "group3_caredutieskids.4",
    group.equal = c("loadings"),
    group.partial = c("JC =~ s21_str.4", "JC =~ s21_cd.4")) 

anova(fit1, fit2_partial) # Partial MI would work
```

```
## 
## Chi-Squared Difference Test
## 
##              Df    AIC    BIC  Chisq Chisq diff    RMSEA Df diff Pr(>Chisq)
## fit1          6 4235.4 4389.9 15.506                                       
## fit2_partial  8 4234.8 4380.7 18.879     3.3724 0.061742       2     0.1852
```

```
# The full model later on does not converge, we discarded one subgroup
### Group 3 - only 1+ vs no kids <11y but other care duties
fit1 <- cfa(model = model_jc_w,
    data  = filter(data, group3_caredutieskids.4 %in% c("1+", "no kids")),
    estimator = "ML",
    missing = "ML",
    group = "group3_caredutieskids.4")

fit2 <- cfa(model = model_jc_w,
    data  = filter(data, group3_caredutieskids.4 %in% c("1+", "no kids")),
    estimator = "ML",
    missing = "ML",
    group = "group3_caredutieskids.4",
    group.equal = c("loadings"))

fit3 <- cfa(model = model_jc_w,
    data  = filter(data, group3_caredutieskids.4 %in% c("1+", "no kids")),
    estimator = "ML",
    missing = "ML",
    group = "group3_caredutieskids.4",
    group.equal = c("loadings", "intercepts"))

anova(fit1, fit2, fit3) # Metric MI does not hold for this time point
```

```
## 
## Chi-Squared Difference Test
## 
##      Df    AIC    BIC  Chisq Chisq diff    RMSEA Df diff Pr(>Chisq)  
## fit1  4 2788.6 2881.3 14.747                                         
## fit2  7 2790.8 2871.9 22.903     8.1565 0.098964       3    0.04289 *
## fit3 10 2785.9 2855.4 23.982     1.0788 0.000000       3    0.78219  
## ---
## Signif. codes:  0 '***' 0.001 '**' 0.01 '*' 0.05 '.' 0.1 ' ' 1
```

```
summary(fit1, fit.measures = TRUE)
```

```
## lavaan 0.6.16 ended normally after 49 iterations
## 
##   Estimator                                         ML
##   Optimization method                           NLMINB
##   Number of model parameters                        24
## 
##   Number of observations per group:                   
##     no kids                                        246
##     1+                                             105
##   Number of missing patterns per group:               
##     no kids                                          1
##     1+                                               1
## 
## Model Test User Model:
##                                                       
##   Test statistic                                14.747
##   Degrees of freedom                                 4
##   P-value (Chi-square)                           0.005
##   Test statistic for each group:
##     no kids                                      2.708
##     1+                                          12.039
## 
## Model Test Baseline Model:
## 
##   Test statistic                               309.526
##   Degrees of freedom                                12
##   P-value                                        0.000
## 
## User Model versus Baseline Model:
## 
##   Comparative Fit Index (CFI)                    0.964
##   Tucker-Lewis Index (TLI)                       0.892
##                                                       
##   Robust Comparative Fit Index (CFI)             0.964
##   Robust Tucker-Lewis Index (TLI)                0.892
## 
## Loglikelihood and Information Criteria:
## 
##   Loglikelihood user model (H0)              -1370.321
##   Loglikelihood unrestricted model (H1)      -1362.948
##                                                       
##   Akaike (AIC)                                2788.642
##   Bayesian (BIC)                              2881.301
##   Sample-size adjusted Bayesian (SABIC)       2805.164
## 
## Root Mean Square Error of Approximation:
## 
##   RMSEA                                          0.124
##   90 Percent confidence interval - lower         0.060
##   90 Percent confidence interval - upper         0.194
##   P-value H_0: RMSEA <= 0.050                    0.031
##   P-value H_0: RMSEA >= 0.080                    0.884
##                                                       
##   Robust RMSEA                                   0.124
##   90 Percent confidence interval - lower         0.060
##   90 Percent confidence interval - upper         0.194
##   P-value H_0: Robust RMSEA <= 0.050             0.031
##   P-value H_0: Robust RMSEA >= 0.080             0.884
## 
## Standardized Root Mean Square Residual:
## 
##   SRMR                                           0.030
## 
## Parameter Estimates:
## 
##   Standard errors                             Standard
##   Information                                 Observed
##   Observed information based on                Hessian
## 
## 
## Group 1 [no kids]:
## 
## Latent Variables:
##                    Estimate  Std.Err  z-value  P(>|z|)
##   JC =~                                               
##     s21_rd.4          1.000                           
##     s21_str.4         1.495    0.280    5.345    0.000
##     s21_cd.4          2.037    0.379    5.381    0.000
##     s21_soz.4         2.126    0.406    5.232    0.000
## 
## Intercepts:
##                    Estimate  Std.Err  z-value  P(>|z|)
##    .s21_rd.4          2.796    0.044   64.189    0.000
##    .s21_str.4         3.759    0.042   89.327    0.000
##    .s21_cd.4          2.455    0.056   43.652    0.000
##    .s21_soz.4         2.511    0.043   58.685    0.000
##     JC                0.000                           
## 
## Variances:
##                    Estimate  Std.Err  z-value  P(>|z|)
##    .s21_rd.4          0.392    0.037   10.460    0.000
##    .s21_str.4         0.269    0.030    9.087    0.000
##    .s21_cd.4          0.469    0.053    8.916    0.000
##    .s21_soz.4         0.113    0.035    3.252    0.001
##     JC                0.075    0.026    2.870    0.004
## 
## 
## Group 2 [1+]:
## 
## Latent Variables:
##                    Estimate  Std.Err  z-value  P(>|z|)
##   JC =~                                               
##     s21_rd.4          1.000                           
##     s21_str.4         0.565    0.189    2.987    0.003
##     s21_cd.4          1.782    0.321    5.543    0.000
##     s21_soz.4         1.314    0.292    4.500    0.000
## 
## Intercepts:
##                    Estimate  Std.Err  z-value  P(>|z|)
##    .s21_rd.4          2.888    0.062   46.449    0.000
##    .s21_str.4         3.813    0.055   69.636    0.000
##    .s21_cd.4          2.632    0.086   30.686    0.000
##    .s21_soz.4         2.712    0.075   36.265    0.000
##     JC                0.000                           
## 
## Variances:
##                    Estimate  Std.Err  z-value  P(>|z|)
##    .s21_rd.4          0.249    0.043    5.807    0.000
##    .s21_str.4         0.265    0.040    6.553    0.000
##    .s21_cd.4          0.273    0.096    2.850    0.004
##    .s21_soz.4         0.316    0.070    4.512    0.000
##     JC                0.157    0.051    3.073    0.002
```

```
lavTestScore(fit2) #suggesting to free str, cd, soz
```

```
## $test
## 
## total score test:
## 
##    test   X2 df p.value
## 1 score 6.13  3   0.105
## 
## $uni
## 
## univariate score tests:
## 
##    lhs op   rhs    X2 df p.value
## 1 .p2. == .p16. 5.721  1   0.017
## 2 .p3. == .p17. 0.712  1   0.399
## 3 .p4. == .p18. 0.331  1   0.565
```

```
parTable(fit2)
```

```
##    id       lhs op       rhs user block group free ustart exo label plabel
## 1   1        JC =~  s21_rd.4    1     1     1    0      1   0         .p1.
## 2   2        JC =~ s21_str.4    1     1     1    1     NA   0  .p2.   .p2.
## 3   3        JC =~  s21_cd.4    1     1     1    2     NA   0  .p3.   .p3.
## 4   4        JC =~ s21_soz.4    1     1     1    3     NA   0  .p4.   .p4.
## 5   5  s21_rd.4 ~~  s21_rd.4    0     1     1    4     NA   0         .p5.
## 6   6 s21_str.4 ~~ s21_str.4    0     1     1    5     NA   0         .p6.
## 7   7  s21_cd.4 ~~  s21_cd.4    0     1     1    6     NA   0         .p7.
## 8   8 s21_soz.4 ~~ s21_soz.4    0     1     1    7     NA   0         .p8.
## 9   9        JC ~~        JC    0     1     1    8     NA   0         .p9.
## 10 10  s21_rd.4 ~1              0     1     1    9     NA   0        .p10.
## 11 11 s21_str.4 ~1              0     1     1   10     NA   0        .p11.
## 12 12  s21_cd.4 ~1              0     1     1   11     NA   0        .p12.
## 13 13 s21_soz.4 ~1              0     1     1   12     NA   0        .p13.
## 14 14        JC ~1              0     1     1    0      0   0        .p14.
## 15 15        JC =~  s21_rd.4    1     2     2    0      1   0        .p15.
## 16 16        JC =~ s21_str.4    1     2     2   13     NA   0  .p2.  .p16.
## 17 17        JC =~  s21_cd.4    1     2     2   14     NA   0  .p3.  .p17.
## 18 18        JC =~ s21_soz.4    1     2     2   15     NA   0  .p4.  .p18.
## 19 19  s21_rd.4 ~~  s21_rd.4    0     2     2   16     NA   0        .p19.
## 20 20 s21_str.4 ~~ s21_str.4    0     2     2   17     NA   0        .p20.
## 21 21  s21_cd.4 ~~  s21_cd.4    0     2     2   18     NA   0        .p21.
## 22 22 s21_soz.4 ~~ s21_soz.4    0     2     2   19     NA   0        .p22.
## 23 23        JC ~~        JC    0     2     2   20     NA   0        .p23.
## 24 24  s21_rd.4 ~1              0     2     2   21     NA   0        .p24.
## 25 25 s21_str.4 ~1              0     2     2   22     NA   0        .p25.
## 26 26  s21_cd.4 ~1              0     2     2   23     NA   0        .p26.
## 27 27 s21_soz.4 ~1              0     2     2   24     NA   0        .p27.
## 28 28        JC ~1              0     2     2    0      0   0        .p28.
## 29 29      .p2. ==     .p16.    2     0     0    0     NA   0             
## 30 30      .p3. ==     .p17.    2     0     0    0     NA   0             
## 31 31      .p4. ==     .p18.    2     0     0    0     NA   0             
##    start   est    se
## 1  1.000 1.000 0.000
## 2  1.461 1.220 0.192
## 3  1.984 1.934 0.271
## 4  1.875 1.979 0.306
## 5  0.233 0.391 0.038
## 6  0.218 0.279 0.029
## 7  0.389 0.465 0.053
## 8  0.225 0.108 0.034
## 9  0.050 0.088 0.025
## 10 2.796 2.796 0.044
## 11 3.759 3.759 0.041
## 12 2.455 2.455 0.057
## 13 2.511 2.511 0.043
## 14 0.000 0.000 0.000
## 15 1.000 1.000 0.000
## 16 0.518 1.220 0.192
## 17 1.931 1.934 0.271
## 18 1.171 1.979 0.306
## 19 0.203 0.296 0.046
## 20 0.157 0.233 0.038
## 21 0.386 0.412 0.078
## 22 0.294 0.231 0.057
## 23 0.050 0.088 0.027
## 24 2.888 2.888 0.060
## 25 3.813 3.813 0.059
## 26 2.632 2.632 0.084
## 27 2.712 2.712 0.074
## 28 0.000 0.000 0.000
## 29 0.000 0.000 0.000
## 30 0.000 0.000 0.000
## 31 0.000 0.000 0.000
```

```
fit2_partial <- cfa(model = model_jc_w,
    data  = filter(data, group3_caredutieskids.4 %in% c("1+", "no kids")),
    estimator = "ML",
    missing = "ML",
    group = "group3_caredutieskids.4",
    group.equal = c("loadings"),
    group.partial = c("JC =~ s21_str.4")) 

anova(fit1, fit2_partial) # Now metric MI holds!
```

```
## 
## Chi-Squared Difference Test
## 
##              Df    AIC    BIC  Chisq Chisq diff    RMSEA Df diff Pr(>Chisq)
## fit1          4 2788.6 2881.3 14.747                                       
## fit2_partial  6 2787.3 2872.2 17.383     2.6363 0.042578       2     0.2676
```

```
## Off-Job Crafting
model_ojc_w <- '
OJC =~ s200_de.4 + s200_re.4 + s200_au.4 + s200_ma.4 + s200_me.4 + s200_af.4
s200_de.4 ~~ s200_re.4
'

### Group 3
fit1 <- cfa(model = model_ojc_w,
    data  = data,
    estimator = "ML",
    missing = "ML",
    group = "group3_caredutieskids.4")

fit2 <- cfa(model = model_ojc_w,
    data  = data,
    estimator = "ML",
    missing = "ML",
    group = "group3_caredutieskids.4",
    group.equal = c("loadings"))

fit3 <- cfa(model = model_ojc_w,
    data  = data,
    estimator = "ML",
    missing = "ML",
    group = "group3_caredutieskids.4",
    group.equal = c("loadings", "intercepts"))

anova(fit1, fit2, fit3) # Metric MI does hold for this group and time point
```

```
## 
## Chi-Squared Difference Test
## 
##      Df    AIC    BIC  Chisq Chisq diff   RMSEA Df diff Pr(>Chisq)    
## fit1 24 6678.8 6923.1 126.25                                          
## fit2 34 6666.3 6867.7 133.66     7.4139 0.00000      10  0.6858779    
## fit3 44 6675.9 6834.5 163.33    29.6691 0.10483      10  0.0009701 ***
## ---
## Signif. codes:  0 '***' 0.001 '**' 0.01 '*' 0.05 '.' 0.1 ' ' 1
```

```
summary(fit1, fit.measures = TRUE)
```

```
## lavaan 0.6.16 ended normally after 74 iterations
## 
##   Estimator                                         ML
##   Optimization method                           NLMINB
##   Number of model parameters                        57
## 
##   Number of observations per group:               Used       Total
##     no kids                                        244         246
##     none at all                                    188         189
##     1+                                             105         105
##   Number of missing patterns per group:                           
##     no kids                                          6            
##     none at all                                      4            
##     1+                                               1            
## 
## Model Test User Model:
##                                                       
##   Test statistic                               126.251
##   Degrees of freedom                                24
##   P-value (Chi-square)                           0.000
##   Test statistic for each group:
##     no kids                                     84.145
##     none at all                                 20.884
##     1+                                          21.222
## 
## Model Test Baseline Model:
## 
##   Test statistic                              1544.263
##   Degrees of freedom                                45
##   P-value                                        0.000
## 
## User Model versus Baseline Model:
## 
##   Comparative Fit Index (CFI)                    0.932
##   Tucker-Lewis Index (TLI)                       0.872
##                                                       
##   Robust Comparative Fit Index (CFI)             0.932
##   Robust Tucker-Lewis Index (TLI)                0.872
## 
## Loglikelihood and Information Criteria:
## 
##   Loglikelihood user model (H0)              -3282.424
##   Loglikelihood unrestricted model (H1)      -3219.299
##                                                       
##   Akaike (AIC)                                6678.848
##   Bayesian (BIC)                              6923.150
##   Sample-size adjusted Bayesian (SABIC)       6742.213
## 
## Root Mean Square Error of Approximation:
## 
##   RMSEA                                          0.154
##   90 Percent confidence interval - lower         0.128
##   90 Percent confidence interval - upper         0.181
##   P-value H_0: RMSEA <= 0.050                    0.000
##   P-value H_0: RMSEA >= 0.080                    1.000
##                                                       
##   Robust RMSEA                                   0.155
##   90 Percent confidence interval - lower         0.129
##   90 Percent confidence interval - upper         0.182
##   P-value H_0: Robust RMSEA <= 0.050             0.000
##   P-value H_0: Robust RMSEA >= 0.080             1.000
## 
## Standardized Root Mean Square Residual:
## 
##   SRMR                                           0.044
## 
## Parameter Estimates:
## 
##   Standard errors                             Standard
##   Information                                 Observed
##   Observed information based on                Hessian
## 
## 
## Group 1 [no kids]:
## 
## Latent Variables:
##                    Estimate  Std.Err  z-value  P(>|z|)
##   OJC =~                                              
##     s200_de.4         1.000                           
##     s200_re.4         1.069    0.112    9.575    0.000
##     s200_au.4         1.436    0.189    7.582    0.000
##     s200_ma.4         1.490    0.203    7.346    0.000
##     s200_me.4         1.434    0.201    7.123    0.000
##     s200_af.4         1.305    0.199    6.552    0.000
## 
## Covariances:
##                    Estimate  Std.Err  z-value  P(>|z|)
##  .s200_de.4 ~~                                        
##    .s200_re.4         0.316    0.045    6.999    0.000
## 
## Intercepts:
##                    Estimate  Std.Err  z-value  P(>|z|)
##    .s200_de.4         3.852    0.061   63.068    0.000
##    .s200_re.4         3.727    0.054   69.486    0.000
##    .s200_au.4         3.731    0.053   70.429    0.000
##    .s200_ma.4         3.446    0.055   62.696    0.000
##    .s200_me.4         3.612    0.053   67.737    0.000
##    .s200_af.4         3.771    0.059   64.428    0.000
##     OJC               0.000                           
## 
## Variances:
##                    Estimate  Std.Err  z-value  P(>|z|)
##    .s200_de.4         0.690    0.066   10.418    0.000
##    .s200_re.4         0.452    0.045   10.017    0.000
##    .s200_au.4         0.235    0.033    7.192    0.000
##    .s200_ma.4         0.244    0.033    7.405    0.000
##    .s200_me.4         0.245    0.035    7.061    0.000
##    .s200_af.4         0.462    0.052    8.956    0.000
##     OJC               0.217    0.058    3.770    0.000
## 
## 
## Group 2 [none at all]:
## 
## Latent Variables:
##                    Estimate  Std.Err  z-value  P(>|z|)
##   OJC =~                                              
##     s200_de.4         1.000                           
##     s200_re.4         0.984    0.146    6.734    0.000
##     s200_au.4         1.627    0.263    6.185    0.000
##     s200_ma.4         1.415    0.244    5.792    0.000
##     s200_me.4         1.438    0.248    5.802    0.000
##     s200_af.4         1.310    0.240    5.450    0.000
## 
## Covariances:
##                    Estimate  Std.Err  z-value  P(>|z|)
##  .s200_de.4 ~~                                        
##    .s200_re.4         0.263    0.048    5.500    0.000
## 
## Intercepts:
##                    Estimate  Std.Err  z-value  P(>|z|)
##    .s200_de.4         3.949    0.065   61.053    0.000
##    .s200_re.4         3.918    0.059   66.506    0.000
##    .s200_au.4         3.802    0.059   64.966    0.000
##    .s200_ma.4         3.399    0.058   58.154    0.000
##    .s200_me.4         3.502    0.057   60.998    0.000
##    .s200_af.4         3.727    0.064   58.330    0.000
##     OJC               0.000                           
## 
## Variances:
##                    Estimate  Std.Err  z-value  P(>|z|)
##    .s200_de.4         0.616    0.067    9.196    0.000
##    .s200_re.4         0.488    0.054    8.996    0.000
##    .s200_au.4         0.194    0.036    5.319    0.000
##    .s200_ma.4         0.296    0.039    7.506    0.000
##    .s200_me.4         0.266    0.037    7.112    0.000
##    .s200_af.4         0.470    0.055    8.524    0.000
##     OJC               0.170    0.055    3.110    0.002
## 
## 
## Group 3 [1+]:
## 
## Latent Variables:
##                    Estimate  Std.Err  z-value  P(>|z|)
##   OJC =~                                              
##     s200_de.4         1.000                           
##     s200_re.4         1.273    0.211    6.020    0.000
##     s200_au.4         1.448    0.252    5.754    0.000
##     s200_ma.4         1.642    0.295    5.557    0.000
##     s200_me.4         1.384    0.249    5.546    0.000
##     s200_af.4         1.288    0.241    5.335    0.000
## 
## Covariances:
##                    Estimate  Std.Err  z-value  P(>|z|)
##  .s200_de.4 ~~                                        
##    .s200_re.4         0.125    0.049    2.544    0.011
## 
## Intercepts:
##                    Estimate  Std.Err  z-value  P(>|z|)
##    .s200_de.4         3.825    0.083   46.085    0.000
##    .s200_re.4         3.632    0.082   44.334    0.000
##    .s200_au.4         3.827    0.076   50.318    0.000
##    .s200_ma.4         3.490    0.089   39.358    0.000
##    .s200_me.4         3.695    0.072   51.231    0.000
##    .s200_af.4         3.757    0.078   48.457    0.000
##     OJC               0.000                           
## 
## Variances:
##                    Estimate  Std.Err  z-value  P(>|z|)
##    .s200_de.4         0.514    0.075    6.853    0.000
##    .s200_re.4         0.365    0.057    6.436    0.000
##    .s200_au.4         0.168    0.033    5.092    0.000
##    .s200_ma.4         0.261    0.047    5.505    0.000
##    .s200_me.4         0.145    0.029    5.010    0.000
##    .s200_af.4         0.283    0.045    6.300    0.000
##     OJC               0.210    0.074    2.817    0.005
```

```
# Measurement model with partial MI for this group 

# For the GC3, the loadings of str & cd to JC need to be freed across groups
measurement_model_parcelled_jc_ojc_partialMI_GC3 <- '
# # # # # # # # # # # # # # # # # # # # #
# Specify parameters for job crafting ----
# # # # # # # # # # # # # # # # # # # # #
# Specify latent true scores
JC_W1 =~ s21_rd.3 + c(jc1_1, jc1_2, jc1_3)*s21_str.3 + c(jc2_1, jc2_2, jc2_3)*s21_cd.3 + jc3*s21_soz.3
JC_W2 =~ s21_rd.4 + c(jc1_1, jc1_2, jc1_3)*s21_str.4 + c(jc2_1, jc2_2, jc2_3)*s21_cd.4 + jc3*s21_soz.4
JC_W3 =~ s21_rd.6 + c(jc1_1, jc1_2, jc1_3)*s21_str.6 + c(jc2_1, jc2_2, jc2_3)*s21_cd.6 + jc3*s21_soz.6
JC_W4 =~ s21_rd.7 + c(jc1_1, jc1_2, jc1_3)*s21_str.7 + c(jc2_1, jc2_2, jc2_3)*s21_cd.7 + jc3*s21_soz.7


# Residual covariances between same items
s21_rd.3 ~~ s21_rd.4 + s21_rd.6 + s21_rd.7
s21_rd.4 ~~ s21_rd.6 + s21_rd.7
s21_rd.6 ~~ s21_rd.7
s21_str.3 ~~ s21_str.4 + s21_str.6 + s21_str.7
s21_str.4 ~~ s21_str.6 + s21_str.7
s21_str.6 ~~ s21_str.7
s21_cd.3 ~~ s21_cd.4 + s21_cd.6 + s21_cd.7
s21_cd.4 ~~ s21_cd.6 + s21_cd.7
s21_cd.6 ~~ s21_cd.7
s21_soz.3 ~~ s21_soz.4 + s21_soz.6 + s21_soz.7
s21_soz.4 ~~ s21_soz.6 + s21_soz.7
s21_soz.6 ~~ s21_soz.7

# Specify mean of latent true scores
JC_W1 ~ 1 # label gamma_jc1 removed
JC_W2 ~ 0 * 1
JC_W3 ~ 0 * 1
JC_W4 ~ 0 * 1

# Specify variance of latent true scores
JC_W1 ~~ JC_W1 # label sigma2_jc1 removed
JC_W2 ~~ 0 * JC_W2
JC_W3 ~~ 0 * JC_W3
JC_W4 ~~ 0 * JC_W4

# Specify autoregressions of latent variables
JC_W2 ~ 1 * JC_W1
JC_W3 ~ 1 * JC_W2
JC_W4 ~ 1 * JC_W3

# Specify latent change scores
djc2 =~ 1 * JC_W2
djc3 =~ 1 * JC_W3
djc4 =~ 1 * JC_W4

# Specify latent change scores means (from Geiser)
djc2 ~ 1 # label gamma_jc2 removed
djc3 ~ 1 # label gamma_jc3 removed
djc4 ~ 1 # label gamma_jc4 removed

# Specify latent change scores variances (from Geiser)
djc2 ~~ djc2 # label res_jc2 removed
djc3 ~~ djc3 # label res_jc3 removed
djc4 ~~ djc4 # label res_jc4 removed

# Specify change component - not proportional!
djc2 ~ JC_W1 # label beta_jc1 removed
djc3 ~ JC_W2 # label beta_jc2 removed
djc4 ~ JC_W3 # label beta_jc3 removed

# Intercepts of reference variables set to zero (from Geiser)
s21_rd.3 ~ 0 * 1
s21_rd.4 ~ 0 * 1
s21_rd.6 ~ 0 * 1
s21_rd.7 ~ 0 * 1

# Change factors are allowed to correlate (from Geiser)
djc2 ~~ djc3 + djc4
djc3 ~~ djc4

# # # # # # # # # # # # # # # # # # # # #
# Specify parameters for off-job crafting ----
# # # # # # # # # # # # # # # # # # # # #
# Specify latent true scores
OJC_W1 =~ s200_de.3 + ojc1*s200_re.3 + ojc2*s200_au.3 + ojc3*s200_ma.3 + ojc4*s200_me.3 + ojc5*s200_af.3
OJC_W2 =~ s200_de.4 + ojc1*s200_re.4 + ojc2*s200_au.4 + ojc3*s200_ma.4 + ojc4*s200_me.4 + ojc5*s200_af.4
OJC_W3 =~ s200_de.6 + ojc1*s200_re.6 + ojc2*s200_au.6 + ojc3*s200_ma.6 + ojc4*s200_me.6 + ojc5*s200_af.6
OJC_W4 =~ s200_de.7 + ojc1*s200_re.7 + ojc2*s200_au.7 + ojc3*s200_ma.7 + ojc4*s200_me.7 + ojc5*s200_af.7

# Allowing a residual covariance between detachment & relaxation
s200_de.3 ~~ s200_re.3
s200_de.4 ~~ s200_re.4
s200_de.6 ~~ s200_re.6
s200_de.7 ~~ s200_re.7

# Residual covariances for same items
s200_de.3 ~~ s200_de.4 + s200_de.6 + s200_de.7
s200_de.4 ~~ s200_de.6 + s200_de.7
s200_de.6 ~~ s200_de.7
s200_re.3 ~~ s200_re.4 + s200_re.6 + s200_re.7
s200_re.4 ~~ s200_re.6 + s200_re.7
s200_re.6 ~~ s200_re.7
s200_au.3 ~~ s200_au.4 + s200_au.6 + s200_au.7
s200_au.4 ~~ s200_au.6 + s200_au.7
s200_au.6 ~~ s200_au.7
s200_ma.3 ~~ s200_ma.4 + s200_ma.6 + s200_ma.7
s200_ma.4 ~~ s200_ma.6 + s200_ma.7
s200_ma.6 ~~ s200_ma.7
s200_me.3 ~~ s200_me.4 + s200_me.6 + s200_me.7
s200_me.4 ~~ s200_me.6 + s200_me.7
s200_me.6 ~~ s200_me.7
s200_af.3 ~~ s200_af.4 + s200_af.6 + s200_af.7
s200_af.4 ~~ s200_af.6 + s200_af.7
s200_af.6 ~~ s200_af.7

# Specify mean of latent true scores 
OJC_W1 ~ 1 # label gamma_ojc1 removed
OJC_W2 ~ 0 * 1
OJC_W3 ~ 0 * 1
OJC_W4 ~ 0 * 1

# Specify variance of latent true scores 
OJC_W1 ~~ OJC_W1 # label sigma2_ojc1 removed
OJC_W2 ~~ 0 * OJC_W2
OJC_W3 ~~ 0 * OJC_W3
OJC_W4 ~~ 0 * OJC_W4

# Specify autoregressions of latent variables 
OJC_W2 ~ 1 * OJC_W1
OJC_W3 ~ 1 * OJC_W2
OJC_W4 ~ 1 * OJC_W3

# Specify latent change scores 
dojc2 =~ 1 * OJC_W2
dojc3 =~ 1 * OJC_W3
dojc4 =~ 1 * OJC_W4

# Specify latent change scores means (from Geiser)
dojc2 ~ 1 # label gamma_ojc2 removed
dojc3 ~ 1 # label gamma_ojc3 removed
dojc4 ~ 1 # label gamma_ojc4 removed

# Specify latent change scores variances (from Geiser)
dojc2 ~~ dojc2 # label res_ojc2 removed
dojc3 ~~ dojc3 # label res_ojc3 removed
dojc4 ~~ dojc4 # label res_ojc4 removed

# Specify change component - not proportional!
dojc2 ~ OJC_W1 # label beta_ojc1 removed
dojc3 ~ OJC_W2 # label beta_ojc2 removed
dojc4 ~ OJC_W3 # label beta_ojc3 removed

# Intercepts of reference variables set to zero (from Geiser)
s200_de.3 ~ 0 * 1
s200_de.4 ~ 0 * 1
s200_de.6 ~ 0 * 1
s200_de.7 ~ 0 * 1

# Change factors are allowed to correlate (from Geiser)
dojc2 ~~ dojc3 + dojc4
dojc3 ~~ dojc4
'

measurement_model_parcelled_jc_ojc_partialMI_GC3_V2 <- '
# # # # # # # # # # # # # # # # # # # # #
# Specify parameters for job crafting ----
# # # # # # # # # # # # # # # # # # # # #
# Specify latent true scores
JC_W1 =~ s21_rd.3 + c(jc1_1, jc1_2)*s21_str.3 + c(jc2_1, jc2_2)*s21_cd.3 + jc3*s21_soz.3
JC_W2 =~ s21_rd.4 + c(jc1_1, jc1_2)*s21_str.4 + c(jc2_1, jc2_2)*s21_cd.4 + jc3*s21_soz.4
JC_W3 =~ s21_rd.6 + c(jc1_1, jc1_2)*s21_str.6 + c(jc2_1, jc2_2)*s21_cd.6 + jc3*s21_soz.6
JC_W4 =~ s21_rd.7 + c(jc1_1, jc1_2)*s21_str.7 + c(jc2_1, jc2_2)*s21_cd.7 + jc3*s21_soz.7


# Residual covariances between same items
s21_rd.3 ~~ s21_rd.4 + s21_rd.6 + s21_rd.7
s21_rd.4 ~~ s21_rd.6 + s21_rd.7
s21_rd.6 ~~ s21_rd.7
s21_str.3 ~~ s21_str.4 + s21_str.6 + s21_str.7
s21_str.4 ~~ s21_str.6 + s21_str.7
s21_str.6 ~~ s21_str.7
s21_cd.3 ~~ s21_cd.4 + s21_cd.6 + s21_cd.7
s21_cd.4 ~~ s21_cd.6 + s21_cd.7
s21_cd.6 ~~ s21_cd.7
s21_soz.3 ~~ s21_soz.4 + s21_soz.6 + s21_soz.7
s21_soz.4 ~~ s21_soz.6 + s21_soz.7
s21_soz.6 ~~ s21_soz.7

# Specify mean of latent true scores
JC_W1 ~ 1 # label gamma_jc1 removed
JC_W2 ~ 0 * 1
JC_W3 ~ 0 * 1
JC_W4 ~ 0 * 1

# Specify variance of latent true scores
JC_W1 ~~ JC_W1 # label sigma2_jc1 removed
JC_W2 ~~ 0 * JC_W2
JC_W3 ~~ 0 * JC_W3
JC_W4 ~~ 0 * JC_W4

# Specify autoregressions of latent variables
JC_W2 ~ 1 * JC_W1
JC_W3 ~ 1 * JC_W2
JC_W4 ~ 1 * JC_W3

# Specify latent change scores
djc2 =~ 1 * JC_W2
djc3 =~ 1 * JC_W3
djc4 =~ 1 * JC_W4

# Specify latent change scores means (from Geiser)
djc2 ~ 1 # label gamma_jc2 removed
djc3 ~ 1 # label gamma_jc3 removed
djc4 ~ 1 # label gamma_jc4 removed

# Specify latent change scores variances (from Geiser)
djc2 ~~ djc2 # label res_jc2 removed
djc3 ~~ djc3 # label res_jc3 removed
djc4 ~~ djc4 # label res_jc4 removed

# Specify change component - not proportional!
djc2 ~ JC_W1 # label beta_jc1 removed
djc3 ~ JC_W2 # label beta_jc2 removed
djc4 ~ JC_W3 # label beta_jc3 removed

# Intercepts of reference variables set to zero (from Geiser)
s21_rd.3 ~ 0 * 1
s21_rd.4 ~ 0 * 1
s21_rd.6 ~ 0 * 1
s21_rd.7 ~ 0 * 1

# Change factors are allowed to correlate (from Geiser)
djc2 ~~ djc3 + djc4
djc3 ~~ djc4

# # # # # # # # # # # # # # # # # # # # #
# Specify parameters for off-job crafting ----
# # # # # # # # # # # # # # # # # # # # #
# Specify latent true scores
OJC_W1 =~ s200_de.3 + ojc1*s200_re.3 + ojc2*s200_au.3 + ojc3*s200_ma.3 + ojc4*s200_me.3 + ojc5*s200_af.3
OJC_W2 =~ s200_de.4 + ojc1*s200_re.4 + ojc2*s200_au.4 + ojc3*s200_ma.4 + ojc4*s200_me.4 + ojc5*s200_af.4
OJC_W3 =~ s200_de.6 + ojc1*s200_re.6 + ojc2*s200_au.6 + ojc3*s200_ma.6 + ojc4*s200_me.6 + ojc5*s200_af.6
OJC_W4 =~ s200_de.7 + ojc1*s200_re.7 + ojc2*s200_au.7 + ojc3*s200_ma.7 + ojc4*s200_me.7 + ojc5*s200_af.7

# Allowing a residual covariance between detachment & relaxation
s200_de.3 ~~ s200_re.3
s200_de.4 ~~ s200_re.4
s200_de.6 ~~ s200_re.6
s200_de.7 ~~ s200_re.7

# Residual covariances for same items
s200_de.3 ~~ s200_de.4 + s200_de.6 + s200_de.7
s200_de.4 ~~ s200_de.6 + s200_de.7
s200_de.6 ~~ s200_de.7
s200_re.3 ~~ s200_re.4 + s200_re.6 + s200_re.7
s200_re.4 ~~ s200_re.6 + s200_re.7
s200_re.6 ~~ s200_re.7
s200_au.3 ~~ s200_au.4 + s200_au.6 + s200_au.7
s200_au.4 ~~ s200_au.6 + s200_au.7
s200_au.6 ~~ s200_au.7
s200_ma.3 ~~ s200_ma.4 + s200_ma.6 + s200_ma.7
s200_ma.4 ~~ s200_ma.6 + s200_ma.7
s200_ma.6 ~~ s200_ma.7
s200_me.3 ~~ s200_me.4 + s200_me.6 + s200_me.7
s200_me.4 ~~ s200_me.6 + s200_me.7
s200_me.6 ~~ s200_me.7
s200_af.3 ~~ s200_af.4 + s200_af.6 + s200_af.7
s200_af.4 ~~ s200_af.6 + s200_af.7
s200_af.6 ~~ s200_af.7

# Specify mean of latent true scores 
OJC_W1 ~ 1 # label gamma_ojc1 removed
OJC_W2 ~ 0 * 1
OJC_W3 ~ 0 * 1
OJC_W4 ~ 0 * 1

# Specify variance of latent true scores 
OJC_W1 ~~ OJC_W1 # label sigma2_ojc1 removed
OJC_W2 ~~ 0 * OJC_W2
OJC_W3 ~~ 0 * OJC_W3
OJC_W4 ~~ 0 * OJC_W4

# Specify autoregressions of latent variables 
OJC_W2 ~ 1 * OJC_W1
OJC_W3 ~ 1 * OJC_W2
OJC_W4 ~ 1 * OJC_W3

# Specify latent change scores 
dojc2 =~ 1 * OJC_W2
dojc3 =~ 1 * OJC_W3
dojc4 =~ 1 * OJC_W4

# Specify latent change scores means (from Geiser)
dojc2 ~ 1 # label gamma_ojc2 removed
dojc3 ~ 1 # label gamma_ojc3 removed
dojc4 ~ 1 # label gamma_ojc4 removed

# Specify latent change scores variances (from Geiser)
dojc2 ~~ dojc2 # label res_ojc2 removed
dojc3 ~~ dojc3 # label res_ojc3 removed
dojc4 ~~ dojc4 # label res_ojc4 removed

# Specify change component - not proportional!
dojc2 ~ OJC_W1 # label beta_ojc1 removed
dojc3 ~ OJC_W2 # label beta_ojc2 removed
dojc4 ~ OJC_W3 # label beta_ojc3 removed

# Intercepts of reference variables set to zero (from Geiser)
s200_de.3 ~ 0 * 1
s200_de.4 ~ 0 * 1
s200_de.6 ~ 0 * 1
s200_de.7 ~ 0 * 1

# Change factors are allowed to correlate (from Geiser)
dojc2 ~~ dojc3 + dojc4
dojc3 ~~ dojc4
'

measurement_model_srh <- '
# # # # # # # # # # # # # # # # # # # # # # # # # # #
# Add outcome self-rated health and regressions ----
# # # # # # # # # # # # # # # # # # # # # # # # # # #

# Specify latent true scores 
SRH_W1 =~ 1 * s38.3r
SRH_W2 =~ 1 * s38.4r
SRH_W3 =~ 1 * s38.6 # no reverse code needed
SRH_W4 =~ 1 * s38.7 # no reverse code needed

# Specify intercept of obseved scores
s38.3r ~ 0 * 1
s38.4r ~ 0 * 1
s38.6 ~ 0 * 1
s38.7 ~ 0 * 1

# Settng residual variances equal across time
s38.3r ~~ res_srh * s38.3r
s38.4r ~~ res_srh * s38.4r
s38.6 ~~ res_srh * s38.6
s38.7 ~~ res_srh * s38.7

# Specifying latent means
SRH_W1 ~ 1 # label gamma_srh1 removed
SRH_W2 ~ 1 # label gamma_srh2 removed
SRH_W3 ~ 1 # label gamma_srh3 removed
SRH_W4 ~ 1 # label gamma_srh4 removed

# Autoregressions
SRH_W2 ~ SRH_W1
SRH_W3 ~ SRH_W2
SRH_W4 ~ SRH_W3

'

model_latent_srh <- '
# Covariances of change scores
djc2 ~~ dojc2
djc3 ~~ dojc3
djc4 ~~ dojc4
                           
# Specify regressions of latent variables and of change scores 
SRH_W2 ~ djc2 + dojc2 + c137.6 # Controlling for high risk group 
SRH_W3 ~ djc3 + dojc3 + c137.6 # Controlling for high risk group 
SRH_W4 ~ djc4 + dojc4 + c137.6 # Controlling for high risk group

# control variable is allowed to correlate with remaining factors
c137.6 ~~ JC_W1 + OJC_W1 + SRH_W1 + djc2 + djc3 + djc4 + dojc2 + dojc3 + dojc4
                    
'

# For this group comparison, the same measurement model as for the full sample can not be used, as the factor loading from JC_SOZ needs to be released.
# Additionally, the initially set up third group needs to be discarded as this group was significantly different from the other two groups and partial metric MI could not be established.

## LCSM Job and Off-Job Crafting with self-rated health as outcome ----

### Model estimation ----
model_lcsm_parcel_SRH_GC3 <- paste(measurement_model_parcelled_jc_ojc_partialMI_GC3, measurement_model_srh, model_latent_srh, sep = "")

lcsm_parcel_SRH_GC3 <- sem(model_lcsm_parcel_SRH_GC3, data, 
                         estimator = "ML", 
                         missing = "ML",
                         group = "group3_caredutieskids.4",
                         group.partial = c("JC_W1 =~ s21_soz.3", 
                                           "JC_W2 =~ s21_soz.4",
                                           "JC_W3 =~ s21_soz.6",
                                           "JC_W4 =~ s21_soz.7"),
                         em.h1.iter.max = 1000000); beep(6)

model_lcsm_parcel_SRH_GC3 <- paste(measurement_model_parcelled_jc_ojc_partialMI_GC3_V2, measurement_model_srh, model_latent_srh, sep = "")
lcsm_parcel_SRH_GC3 <- sem(model_lcsm_parcel_SRH_GC3, filter(data, group3_caredutieskids.4 %in% c("1+", "no kids")), 
                         estimator = "ML", 
                         missing = "ML",
                         group = "group3_caredutieskids.4",
                         group.partial = c("JC_W1 =~ s21_soz.3", 
                                           "JC_W2 =~ s21_soz.4",
                                           "JC_W3 =~ s21_soz.6",
                                           "JC_W4 =~ s21_soz.7"),
                         em.h1.iter.max = 1000000); beep(6)

summary(lcsm_parcel_SRH_GC3, fit.measures = TRUE, standardized = TRUE, fm.args = list(robust = FALSE))
```

```
## lavaan 0.6.16 ended normally after 481 iterations
## 
##   Estimator                                         ML
##   Optimization method                           NLMINB
##   Number of model parameters                       474
##   Number of equality constraints                    61
## 
##   Number of observations per group:                   
##     no kids                                        246
##     1+                                             105
##   Number of missing patterns per group:               
##     no kids                                         21
##     1+                                              11
## 
## Model Test User Model:
##                                                       
##   Test statistic                              6309.252
##   Degrees of freedom                              1747
##   P-value (Chi-square)                           0.000
##   Test statistic for each group:
##     no kids                                   1513.164
##     1+                                        4796.089
## 
## Model Test Baseline Model:
## 
##   Test statistic                             13770.049
##   Degrees of freedom                              1980
##   P-value                                        0.000
## 
## User Model versus Baseline Model:
## 
##   Comparative Fit Index (CFI)                    0.613
##   Tucker-Lewis Index (TLI)                       0.561
## 
## Loglikelihood and Information Criteria:
## 
##   Loglikelihood user model (H0)              -9177.547
##   Loglikelihood unrestricted model (H1)      -6022.921
##                                                       
##   Akaike (AIC)                               19181.095
##   Bayesian (BIC)                             20775.599
##   Sample-size adjusted Bayesian (SABIC)      19465.410
## 
## Root Mean Square Error of Approximation:
## 
##   RMSEA                                          0.122
##   90 Percent confidence interval - lower         0.119
##   90 Percent confidence interval - upper         0.125
##   P-value H_0: RMSEA <= 0.050                    0.000
##   P-value H_0: RMSEA >= 0.080                    1.000
## 
## Standardized Root Mean Square Residual:
## 
##   SRMR                                           0.107
## 
## Parameter Estimates:
## 
##   Standard errors                             Standard
##   Information                                 Observed
##   Observed information based on                Hessian
## 
## 
## Group 1 [no kids]:
## 
## Latent Variables:
##                    Estimate  Std.Err  z-value  P(>|z|)   Std.lv  Std.all
##   JC_W1 =~                                                              
##     s21_r.3           1.000                               0.309    0.433
##     s21_s.3 (j1_1)    1.146    0.136    8.442    0.000    0.355    0.591
##     s21_c.3 (j2_1)    1.593    0.186    8.542    0.000    0.493    0.577
##     s21_s.3  (jc3)    1.523    0.154    9.891    0.000    0.471    0.739
##   JC_W2 =~                                                              
##     s21_r.4           1.000                               0.341    0.488
##     s21_s.4 (j1_1)    1.146    0.136    8.442    0.000    0.391    0.604
##     s21_c.4 (j2_1)    1.593    0.186    8.542    0.000    0.544    0.627
##     s21_s.4  (jc3)    1.523    0.154    9.891    0.000    0.520    0.797
##   JC_W3 =~                                                              
##     s21_r.6           1.000                               0.340    0.508
##     s21_s.6 (j1_1)    1.146    0.136    8.442    0.000    0.389    0.608
##     s21_c.6 (j2_1)    1.593    0.186    8.542    0.000    0.541    0.617
##     s21_s.6  (jc3)    1.523    0.154    9.891    0.000    0.517    0.777
##   JC_W4 =~                                                              
##     s21_r.7           1.000                               0.358    0.497
##     s21_s.7 (j1_1)    1.146    0.136    8.442    0.000    0.410    0.587
##     s21_c.7 (j2_1)    1.593    0.186    8.542    0.000    0.570    0.649
##     s21_s.7  (jc3)    1.523    0.154    9.891    0.000    0.544    0.853
##   djc2 =~                                                               
##     JC_W2             1.000                               0.509    0.509
##   djc3 =~                                                               
##     JC_W3             1.000                               0.543    0.543
##   djc4 =~                                                               
##     JC_W4             1.000                               0.560    0.560
##   OJC_W1 =~                                                             
##     s200_.3           1.000                               0.472    0.558
##     s200_.3 (ojc1)    0.996    0.059   16.816    0.000    0.471    0.575
##     s200_.3 (ojc2)    1.243    0.074   16.838    0.000    0.587    0.799
##     s200_.3 (ojc3)    1.245    0.080   15.576    0.000    0.588    0.741
##     s200_.3 (ojc4)    1.151    0.075   15.255    0.000    0.544    0.727
##     s200_.3 (ojc5)    1.107    0.077   14.346    0.000    0.523    0.632
##   OJC_W2 =~                                                             
##     s200_.4           1.000                               0.559    0.580
##     s200_.4 (ojc1)    0.996    0.059   16.816    0.000    0.557    0.664
##     s200_.4 (ojc2)    1.243    0.074   16.838    0.000    0.695    0.825
##     s200_.4 (ojc3)    1.245    0.080   15.576    0.000    0.696    0.812
##     s200_.4 (ojc4)    1.151    0.075   15.255    0.000    0.644    0.782
##     s200_.4 (ojc5)    1.107    0.077   14.346    0.000    0.619    0.670
##   OJC_W3 =~                                                             
##     s200_.6           1.000                               0.522    0.601
##     s200_.6 (ojc1)    0.996    0.059   16.816    0.000    0.520    0.675
##     s200_.6 (ojc2)    1.243    0.074   16.838    0.000    0.649    0.833
##     s200_.6 (ojc3)    1.245    0.080   15.576    0.000    0.650    0.839
##     s200_.6 (ojc4)    1.151    0.075   15.255    0.000    0.601    0.788
##     s200_.6 (ojc5)    1.107    0.077   14.346    0.000    0.578    0.711
##   OJC_W4 =~                                                             
##     s200_.7           1.000                               0.488    0.569
##     s200_.7 (ojc1)    0.996    0.059   16.816    0.000    0.486    0.651
##     s200_.7 (ojc2)    1.243    0.074   16.838    0.000    0.607    0.833
##     s200_.7 (ojc3)    1.245    0.080   15.576    0.000    0.608    0.801
##     s200_.7 (ojc4)    1.151    0.075   15.255    0.000    0.562    0.715
##     s200_.7 (ojc5)    1.107    0.077   14.346    0.000    0.540    0.685
##   dojc2 =~                                                              
##     OJC_W2            1.000                               0.704    0.704
##   dojc3 =~                                                              
##     OJC_W3            1.000                               0.804    0.804
##   dojc4 =~                                                              
##     OJC_W4            1.000                               0.691    0.691
##   SRH_W1 =~                                                             
##     s38.3r            1.000                               0.746    0.900
##   SRH_W2 =~                                                             
##     s38.4r            1.000                               0.692    0.886
##   SRH_W3 =~                                                             
##     s38.6             1.000                               0.631    0.868
##   SRH_W4 =~                                                             
##     s38.7             1.000                               0.616    0.862
## 
## Regressions:
##                    Estimate  Std.Err  z-value  P(>|z|)   Std.lv  Std.all
##   JC_W2 ~                                                               
##     JC_W1             1.000                               0.906    0.906
##   JC_W3 ~                                                               
##     JC_W2             1.000                               1.005    1.005
##   JC_W4 ~                                                               
##     JC_W3             1.000                               0.950    0.950
##   djc2 ~                                                                
##     JC_W1            -0.049    0.071   -0.690    0.490   -0.087   -0.087
##   djc3 ~                                                                
##     JC_W2            -0.044    0.078   -0.570    0.569   -0.082   -0.082
##   djc4 ~                                                                
##     JC_W3             0.070    0.097    0.721    0.471    0.119    0.119
##   OJC_W2 ~                                                              
##     OJC_W1            1.000                               0.845    0.845
##   OJC_W3 ~                                                              
##     OJC_W2            1.000                               1.072    1.072
##   OJC_W4 ~                                                              
##     OJC_W3            1.000                               1.069    1.069
##   dojc2 ~                                                               
##     OJC_W1           -0.147    0.074   -1.969    0.049   -0.176   -0.176
##   dojc3 ~                                                               
##     OJC_W2           -0.063    0.096   -0.652    0.514   -0.084   -0.084
##   dojc4 ~                                                               
##     OJC_W3           -0.054    0.101   -0.533    0.594   -0.084   -0.084
##   SRH_W2 ~                                                              
##     SRH_W1            0.833    0.067   12.353    0.000    0.899    0.899
##   SRH_W3 ~                                                              
##     SRH_W2            0.905    0.069   13.030    0.000    0.992    0.992
##   SRH_W4 ~                                                              
##     SRH_W3            1.043    0.086   12.135    0.000    1.070    1.070
##   SRH_W2 ~                                                              
##     djc2              0.352    0.306    1.154    0.249    0.089    0.089
##     dojc2             0.278    0.107    2.589    0.010    0.158    0.158
##     c137.6           -0.108    0.106   -1.022    0.307   -0.156   -0.074
##   SRH_W3 ~                                                              
##     djc3              0.419    0.278    1.509    0.131    0.122    0.122
##     dojc3             0.102    0.092    1.107    0.268    0.068    0.068
##     c137.6            0.053    0.103    0.519    0.604    0.085    0.040
##   SRH_W4 ~                                                              
##     djc4              0.337    0.302    1.115    0.265    0.110    0.110
##     dojc4             0.378    0.145    2.602    0.009    0.207    0.207
##     c137.6            0.105    0.109    0.962    0.336    0.170    0.080
## 
## Covariances:
##                    Estimate  Std.Err  z-value  P(>|z|)   Std.lv  Std.all
##  .s21_rd.3 ~~                                                           
##    .s21_rd.4          0.188    0.033    5.704    0.000    0.188    0.478
##    .s21_rd.6          0.198    0.035    5.703    0.000    0.198    0.535
##    .s21_rd.7          0.233    0.039    5.933    0.000    0.233    0.580
##  .s21_rd.4 ~~                                                           
##    .s21_rd.6          0.214    0.032    6.763    0.000    0.214    0.609
##    .s21_rd.7          0.215    0.036    5.990    0.000    0.215    0.563
##  .s21_rd.6 ~~                                                           
##    .s21_rd.7          0.211    0.037    5.749    0.000    0.211    0.587
##  .s21_str.3 ~~                                                          
##    .s21_str.4         0.151    0.023    6.463    0.000    0.151    0.605
##    .s21_str.6         0.153    0.025    6.055    0.000    0.153    0.621
##    .s21_str.7         0.188    0.029    6.513    0.000    0.188    0.688
##  .s21_str.4 ~~                                                          
##    .s21_str.6         0.172    0.025    6.875    0.000    0.172    0.657
##    .s21_str.7         0.181    0.029    6.271    0.000    0.181    0.621
##  .s21_str.6 ~~                                                          
##    .s21_str.7         0.192    0.030    6.443    0.000    0.192    0.670
##  .s21_cd.3 ~~                                                           
##    .s21_cd.4          0.300    0.045    6.596    0.000    0.300    0.635
##    .s21_cd.6          0.366    0.051    7.191    0.000    0.366    0.759
##    .s21_cd.7          0.312    0.051    6.156    0.000    0.312    0.669
##  .s21_cd.4 ~~                                                           
##    .s21_cd.6          0.320    0.047    6.805    0.000    0.320    0.686
##    .s21_cd.7          0.285    0.048    5.975    0.000    0.285    0.629
##  .s21_cd.6 ~~                                                           
##    .s21_cd.7          0.331    0.052    6.311    0.000    0.331    0.718
##  .s21_soz.3 ~~                                                          
##    .s21_soz.4         0.093    0.025    3.747    0.000    0.093    0.551
##    .s21_soz.6         0.077    0.026    2.945    0.003    0.077    0.427
##    .s21_soz.7         0.054    0.025    2.187    0.029    0.054    0.382
##  .s21_soz.4 ~~                                                          
##    .s21_soz.6         0.079    0.024    3.280    0.001    0.079    0.477
##    .s21_soz.7         0.056    0.025    2.278    0.023    0.056    0.429
##  .s21_soz.6 ~~                                                          
##    .s21_soz.7         0.055    0.025    2.196    0.028    0.055    0.392
##  .djc2 ~~                                                               
##    .djc3             -0.012    0.007   -1.704    0.088   -0.398   -0.398
##    .djc4              0.003    0.007    0.358    0.720    0.072    0.072
##  .djc3 ~~                                                               
##    .djc4             -0.024    0.009   -2.794    0.005   -0.653   -0.653
##  .s200_de.3 ~~                                                          
##    .s200_re.3         0.073    0.031    2.379    0.017    0.073    0.155
##  .s200_de.4 ~~                                                          
##    .s200_re.4         0.157    0.031    5.046    0.000    0.157    0.319
##  .s200_de.6 ~~                                                          
##    .s200_re.6         0.133    0.030    4.427    0.000    0.133    0.337
##  .s200_de.7 ~~                                                          
##    .s200_re.7         0.082    0.030    2.702    0.007    0.082    0.205
##  .s200_de.3 ~~                                                          
##    .s200_de.4         0.273    0.045    6.055    0.000    0.273    0.496
##    .s200_de.6         0.225    0.042    5.352    0.000    0.225    0.461
##    .s200_de.7         0.276    0.048    5.778    0.000    0.276    0.557
##  .s200_de.4 ~~                                                          
##    .s200_de.6         0.213    0.041    5.225    0.000    0.213    0.391
##    .s200_de.7         0.274    0.046    6.009    0.000    0.274    0.495
##  .s200_de.6 ~~                                                          
##    .s200_de.7         0.256    0.046    5.621    0.000    0.256    0.524
##  .s200_re.3 ~~                                                          
##    .s200_re.4         0.156    0.033    4.679    0.000    0.156    0.372
##    .s200_re.6         0.141    0.032    4.338    0.000    0.141    0.370
##    .s200_re.7         0.173    0.036    4.770    0.000    0.173    0.455
##  .s200_re.4 ~~                                                          
##    .s200_re.6         0.140    0.027    5.141    0.000    0.140    0.392
##    .s200_re.7         0.129    0.030    4.310    0.000    0.129    0.362
##  .s200_re.6 ~~                                                          
##    .s200_re.7         0.113    0.032    3.539    0.000    0.113    0.350
##  .s200_au.3 ~~                                                          
##    .s200_au.4         0.051    0.021    2.376    0.017    0.051    0.242
##    .s200_au.6         0.049    0.021    2.268    0.023    0.049    0.255
##    .s200_au.7         0.057    0.023    2.457    0.014    0.057    0.319
##  .s200_au.4 ~~                                                          
##    .s200_au.6         0.060    0.022    2.775    0.006    0.060    0.291
##    .s200_au.7         0.057    0.022    2.638    0.008    0.057    0.299
##  .s200_au.6 ~~                                                          
##    .s200_au.7         0.067    0.024    2.844    0.004    0.067    0.385
##  .s200_ma.3 ~~                                                          
##    .s200_ma.4         0.073    0.025    2.874    0.004    0.073    0.272
##    .s200_ma.6         0.112    0.025    4.517    0.000    0.112    0.499
##    .s200_ma.7         0.104    0.028    3.663    0.000    0.104    0.430
##  .s200_ma.4 ~~                                                          
##    .s200_ma.6         0.056    0.023    2.406    0.016    0.056    0.263
##    .s200_ma.7         0.079    0.027    2.898    0.004    0.079    0.348
##  .s200_ma.6 ~~                                                          
##    .s200_ma.7         0.068    0.025    2.792    0.005    0.068    0.357
##  .s200_me.3 ~~                                                          
##    .s200_me.4         0.076    0.025    3.049    0.002    0.076    0.287
##    .s200_me.6         0.060    0.025    2.425    0.015    0.060    0.250
##    .s200_me.7         0.104    0.030    3.420    0.001    0.104    0.369
##  .s200_me.4 ~~                                                          
##    .s200_me.6         0.066    0.023    2.854    0.004    0.066    0.274
##    .s200_me.7         0.080    0.029    2.772    0.006    0.080    0.283
##  .s200_me.6 ~~                                                          
##    .s200_me.7         0.105    0.030    3.464    0.001    0.105    0.406
##  .s200_af.3 ~~                                                          
##    .s200_af.4         0.182    0.037    4.883    0.000    0.182    0.413
##    .s200_af.6         0.127    0.036    3.481    0.000    0.127    0.346
##    .s200_af.7         0.115    0.037    3.095    0.002    0.115    0.313
##  .s200_af.4 ~~                                                          
##    .s200_af.6         0.134    0.036    3.753    0.000    0.134    0.341
##    .s200_af.7         0.178    0.039    4.537    0.000    0.178    0.451
##  .s200_af.6 ~~                                                          
##    .s200_af.7         0.180    0.038    4.783    0.000    0.180    0.549
##  .dojc2 ~~                                                              
##    .dojc3            -0.089    0.026   -3.370    0.001   -0.565   -0.565
##    .dojc4            -0.009    0.019   -0.482    0.630   -0.071   -0.071
##  .dojc3 ~~                                                              
##    .dojc4            -0.051    0.020   -2.483    0.013   -0.385   -0.385
##  .djc2 ~~                                                               
##    .dojc2             0.017    0.007    2.568    0.010    0.249    0.249
##  .djc3 ~~                                                               
##    .dojc3             0.006    0.006    0.989    0.323    0.076    0.076
##  .djc4 ~~                                                               
##    .dojc4            -0.009    0.007   -1.265    0.206   -0.132   -0.132
##   JC_W1 ~~                                                              
##     c137.6           -0.017    0.014   -1.235    0.217   -0.055   -0.116
##   OJC_W1 ~~                                                             
##     c137.6            0.026    0.019    1.388    0.165    0.055    0.117
##   SRH_W1 ~~                                                             
##     c137.6           -0.146    0.031   -4.638    0.000   -0.195   -0.413
##  .djc2 ~~                                                               
##     c137.6           -0.001    0.009   -0.084    0.933   -0.004   -0.009
##  .djc3 ~~                                                               
##     c137.6           -0.016    0.009   -1.789    0.074   -0.087   -0.184
##  .djc4 ~~                                                               
##     c137.6           -0.005    0.011   -0.402    0.688   -0.022   -0.047
##  .dojc2 ~~                                                              
##     c137.6            0.011    0.016    0.687    0.492    0.029    0.060
##  .dojc3 ~~                                                              
##     c137.6           -0.022    0.016   -1.348    0.178   -0.054   -0.114
##  .dojc4 ~~                                                              
##     c137.6            0.002    0.014    0.134    0.894    0.006    0.012
##   JC_W1 ~~                                                              
##     OJC_W1            0.069    0.015    4.443    0.000    0.470    0.470
##     SRH_W1            0.043    0.020    2.150    0.032    0.186    0.186
##   OJC_W1 ~~                                                             
##     SRH_W1            0.149    0.031    4.785    0.000    0.423    0.423
## 
## Intercepts:
##                    Estimate  Std.Err  z-value  P(>|z|)   Std.lv  Std.all
##     JC_W1             2.931    0.050   58.304    0.000    9.471    9.471
##    .JC_W2             0.000                               0.000    0.000
##    .JC_W3             0.000                               0.000    0.000
##    .JC_W4             0.000                               0.000    0.000
##    .djc2              0.008    0.212    0.039    0.969    0.048    0.048
##    .djc3              0.265    0.220    1.202    0.230    1.436    1.436
##    .djc4             -0.325    0.289   -1.124    0.261   -1.626   -1.626
##    .s21_rd.3          0.000                               0.000    0.000
##    .s21_rd.4          0.000                               0.000    0.000
##    .s21_rd.6          0.000                               0.000    0.000
##    .s21_rd.7          0.000                               0.000    0.000
##     OJC_W1            3.976    0.060   66.806    0.000    8.418    8.418
##    .OJC_W2            0.000                               0.000    0.000
##    .OJC_W3            0.000                               0.000    0.000
##    .OJC_W4            0.000                               0.000    0.000
##    .dojc2             0.455    0.302    1.509    0.131    1.155    1.155
##    .dojc3             0.200    0.376    0.532    0.595    0.477    0.477
##    .dojc4             0.274    0.389    0.705    0.481    0.814    0.814
##    .s200_de.3         0.000                               0.000    0.000
##    .s200_de.4         0.000                               0.000    0.000
##    .s200_de.6         0.000                               0.000    0.000
##    .s200_de.7         0.000                               0.000    0.000
##    .s38.3r            0.000                               0.000    0.000
##    .s38.4r            0.000                               0.000    0.000
##    .s38.6             0.000                               0.000    0.000
##    .s38.7             0.000                               0.000    0.000
##     SRH_W1            3.646    0.057   64.206    0.000    4.887    4.887
##    .SRH_W2            0.783    0.267    2.936    0.003    1.132    1.132
##    .SRH_W3            0.226    0.283    0.797    0.425    0.358    0.358
##    .SRH_W4           -0.168    0.329   -0.509    0.611   -0.272   -0.272
##    .s21_str.3         0.512    0.402    1.273    0.203    0.512    0.854
##    .s21_cd.3         -2.228    0.553   -4.027    0.000   -2.228   -2.606
##    .s21_soz.3        -1.855    0.457   -4.057    0.000   -1.855   -2.910
##    .s21_str.4         0.553    0.384    1.442    0.149    0.553    0.854
##    .s21_cd.4         -1.998    0.527   -3.793    0.000   -1.998   -2.302
##    .s21_soz.4        -1.746    0.435   -4.013    0.000   -1.746   -2.677
##    .s21_str.6         0.340    0.403    0.844    0.399    0.340    0.532
##    .s21_cd.6         -2.182    0.554   -3.940    0.000   -2.182   -2.490
##    .s21_soz.6        -1.904    0.458   -4.161    0.000   -1.904   -2.859
##    .s21_str.7         0.347    0.389    0.892    0.372    0.347    0.497
##    .s21_cd.7         -2.175    0.533   -4.079    0.000   -2.175   -2.476
##    .s21_soz.7        -1.869    0.440   -4.246    0.000   -1.869   -2.929
##    .s200_re.3        -0.110    0.244   -0.453    0.651   -0.110   -0.135
##    .s200_au.3        -1.037    0.302   -3.435    0.001   -1.037   -1.412
##    .s200_ma.3        -1.397    0.326   -4.281    0.000   -1.397   -1.760
##    .s200_me.3        -0.890    0.308   -2.889    0.004   -0.890   -1.189
##    .s200_af.3        -0.484    0.315   -1.536    0.124   -0.484   -0.585
##    .s200_re.4        -0.114    0.234   -0.486    0.627   -0.114   -0.136
##    .s200_au.4        -1.059    0.293   -3.617    0.000   -1.059   -1.256
##    .s200_ma.4        -1.351    0.316   -4.276    0.000   -1.351   -1.575
##    .s200_me.4        -0.825    0.298   -2.766    0.006   -0.825   -1.001
##    .s200_af.4        -0.497    0.306   -1.627    0.104   -0.497   -0.538
##    .s200_re.6        -0.160    0.232   -0.689    0.491   -0.160   -0.207
##    .s200_au.6        -1.085    0.290   -3.738    0.000   -1.085   -1.393
##    .s200_ma.6        -1.386    0.313   -4.431    0.000   -1.386   -1.788
##    .s200_me.6        -0.882    0.296   -2.981    0.003   -0.882   -1.157
##    .s200_af.6        -0.573    0.302   -1.896    0.058   -0.573   -0.705
##    .s200_re.7        -0.153    0.238   -0.642    0.521   -0.153   -0.205
##    .s200_au.7        -1.044    0.297   -3.516    0.000   -1.044   -1.433
##    .s200_ma.7        -1.331    0.320   -4.157    0.000   -1.331   -1.755
##    .s200_me.7        -0.871    0.303   -2.871    0.004   -0.871   -1.108
##    .s200_af.7        -0.573    0.309   -1.855    0.064   -0.573   -0.726
##     c137.6            0.330    0.036    9.259    0.000    0.330    0.697
## 
## Variances:
##                    Estimate  Std.Err  z-value  P(>|z|)   Std.lv  Std.all
##     JC_W1             0.096    0.020    4.704    0.000    1.000    1.000
##    .JC_W2             0.000                               0.000    0.000
##    .JC_W3             0.000                               0.000    0.000
##    .JC_W4             0.000                               0.000    0.000
##    .djc2              0.030    0.009    3.491    0.000    0.992    0.992
##    .djc3              0.033    0.009    3.583    0.000    0.961    0.961
##    .djc4              0.043    0.014    3.063    0.002    1.062    1.062
##     OJC_W1            0.223    0.036    6.252    0.000    1.000    1.000
##    .OJC_W2            0.000                               0.000    0.000
##    .OJC_W3            0.000                               0.000    0.000
##    .OJC_W4            0.000                               0.000    0.000
##    .dojc2             0.150    0.025    5.899    0.000    0.969    0.969
##    .dojc3             0.164    0.034    4.863    0.000    0.930    0.930
##    .dojc4             0.107    0.026    4.039    0.000    0.937    0.937
##    .s38.3r  (rs_s)    0.131    0.015    8.987    0.000    0.131    0.190
##    .s38.4r  (rs_s)    0.131    0.015    8.987    0.000    0.131    0.214
##    .s38.6   (rs_s)    0.131    0.015    8.987    0.000    0.131    0.247
##    .s38.7   (rs_s)    0.131    0.015    8.987    0.000    0.131    0.256
##    .s21_r.3           0.414    0.045    9.236    0.000    0.414    0.812
##    .s21_s.3           0.234    0.029    8.210    0.000    0.234    0.650
##    .s21_c.3           0.488    0.058    8.419    0.000    0.488    0.668
##    .s21_s.3           0.184    0.031    5.898    0.000    0.184    0.454
##    .s21_r.4           0.373    0.036   10.224    0.000    0.373    0.762
##    .s21_s.4           0.267    0.029    9.284    0.000    0.267    0.635
##    .s21_c.4           0.458    0.050    9.075    0.000    0.458    0.607
##    .s21_s.4           0.155    0.028    5.576    0.000    0.155    0.365
##    .s21_r.6           0.331    0.039    8.499    0.000    0.331    0.742
##    .s21_s.6           0.258    0.032    8.124    0.000    0.258    0.630
##    .s21_c.6           0.475    0.061    7.836    0.000    0.475    0.619
##    .s21_s.6           0.176    0.032    5.456    0.000    0.176    0.397
##    .s21_r.7           0.390    0.050    7.849    0.000    0.390    0.753
##    .s21_s.7           0.319    0.040    7.915    0.000    0.319    0.655
##    .s21_c.7           0.447    0.063    7.060    0.000    0.447    0.579
##    .s21_s.7           0.110    0.030    3.693    0.000    0.110    0.272
##    .s200_.3           0.493    0.052    9.487    0.000    0.493    0.688
##    .s200_.3           0.448    0.048    9.258    0.000    0.448    0.669
##    .s200_.3           0.195    0.028    6.963    0.000    0.195    0.361
##    .s200_.3           0.284    0.036    7.974    0.000    0.284    0.451
##    .s200_.3           0.264    0.033    8.026    0.000    0.264    0.472
##    .s200_.3           0.411    0.047    8.704    0.000    0.411    0.600
##    .s200_.4           0.618    0.056   11.112    0.000    0.618    0.664
##    .s200_.4           0.394    0.037   10.585    0.000    0.394    0.560
##    .s200_.4           0.227    0.029    7.959    0.000    0.227    0.320
##    .s200_.4           0.251    0.031    8.183    0.000    0.251    0.341
##    .s200_.4           0.263    0.031    8.517    0.000    0.263    0.388
##    .s200_.4           0.472    0.049    9.663    0.000    0.472    0.552
##    .s200_.6           0.482    0.051    9.487    0.000    0.482    0.639
##    .s200_.6           0.323    0.036    8.966    0.000    0.323    0.544
##    .s200_.6           0.186    0.028    6.737    0.000    0.186    0.307
##    .s200_.6           0.178    0.027    6.671    0.000    0.178    0.297
##    .s200_.6           0.220    0.030    7.321    0.000    0.220    0.379
##    .s200_.6           0.326    0.042    7.742    0.000    0.326    0.494
##    .s200_.7           0.496    0.060    8.333    0.000    0.496    0.676
##    .s200_.7           0.321    0.040    7.926    0.000    0.321    0.576
##    .s200_.7           0.163    0.028    5.754    0.000    0.163    0.306
##    .s200_.7           0.206    0.034    6.047    0.000    0.206    0.359
##    .s200_.7           0.302    0.044    6.897    0.000    0.302    0.489
##    .s200_.7           0.331    0.048    6.899    0.000    0.331    0.531
##     c137.6            0.224    0.025    8.877    0.000    0.224    1.000
##     SRH_W1            0.557    0.069    8.033    0.000    1.000    1.000
##    .SRH_W2            0.055    0.026    2.095    0.036    0.116    0.116
##    .SRH_W3            0.022    0.022    0.986    0.324    0.055    0.055
##    .SRH_W4           -0.033    0.027   -1.206    0.228   -0.086   -0.086
## 
## 
## Group 2 [1+]:
## 
## Latent Variables:
##                    Estimate  Std.Err  z-value  P(>|z|)   Std.lv  Std.all
##   JC_W1 =~                                                              
##     s21_r.3           1.000                               0.341    0.515
##     s21_s.3 (j1_2)    0.850    0.152    5.585    0.000    0.289    0.466
##     s21_c.3 (j2_2)    1.983    0.263    7.544    0.000    0.675    0.770
##     s21_s.3  (jc3)    1.523    0.154    9.891    0.000    0.519    0.721
##   JC_W2 =~                                                              
##     s21_r.4           1.000                               0.347    0.562
##     s21_s.4 (j1_2)    0.850    0.152    5.585    0.000    0.295    0.513
##     s21_c.4 (j2_2)    1.983    0.263    7.544    0.000    0.689    0.751
##     s21_s.4  (jc3)    1.523    0.154    9.891    0.000    0.529    0.717
##   JC_W3 =~                                                              
##     s21_r.6           1.000                               0.354    0.530
##     s21_s.6 (j1_2)    0.850    0.152    5.585    0.000    0.301    0.440
##     s21_c.6 (j2_2)    1.983    0.263    7.544    0.000    0.702    0.737
##     s21_s.6  (jc3)    1.523    0.154    9.891    0.000    0.539    0.725
##   JC_W4 =~                                                              
##     s21_r.7           1.000                               0.353    0.553
##     s21_s.7 (j1_2)    0.850    0.152    5.585    0.000    0.300    0.482
##     s21_c.7 (j2_2)    1.983    0.263    7.544    0.000    0.700    0.739
##     s21_s.7  (jc3)    1.523    0.154    9.891    0.000    0.537    0.765
##   djc2 =~                                                               
##     JC_W2             1.000                               0.610    0.610
##   djc3 =~                                                               
##     JC_W3             1.000                               0.503    0.503
##   djc4 =~                                                               
##     JC_W4             1.000                               0.930    0.930
##   OJC_W1 =~                                                             
##     s200_.3           1.000                               0.523    0.562
##     s200_.3 (ojc1)    0.996    0.059   16.816    0.000    0.521    0.623
##     s200_.3 (ojc2)    1.243    0.074   16.838    0.000    0.650    0.803
##     s200_.3 (ojc3)    1.245    0.080   15.576    0.000    0.651    0.724
##     s200_.3 (ojc4)    1.151    0.075   15.255    0.000    0.602    0.763
##     s200_.3 (ojc5)    1.107    0.077   14.346    0.000    0.579    0.733
##   OJC_W2 =~                                                             
##     s200_.4           1.000                               0.544    0.614
##     s200_.4 (ojc1)    0.996    0.059   16.816    0.000    0.542    0.672
##     s200_.4 (ojc2)    1.243    0.074   16.838    0.000    0.676    0.859
##     s200_.4 (ojc3)    1.245    0.080   15.576    0.000    0.677    0.788
##     s200_.4 (ojc4)    1.151    0.075   15.255    0.000    0.626    0.845
##     s200_.4 (ojc5)    1.107    0.077   14.346    0.000    0.602    0.753
##   OJC_W3 =~                                                             
##     s200_.6           1.000                               0.554    0.660
##     s200_.6 (ojc1)    0.996    0.059   16.816    0.000    0.552    0.725
##     s200_.6 (ojc2)    1.243    0.074   16.838    0.000    0.688    0.923
##     s200_.6 (ojc3)    1.245    0.080   15.576    0.000    0.689    0.806
##     s200_.6 (ojc4)    1.151    0.075   15.255    0.000    0.637    0.824
##     s200_.6 (ojc5)    1.107    0.077   14.346    0.000    0.613    0.762
##   OJC_W4 =~                                                             
##     s200_.7           1.000                               0.549    0.648
##     s200_.7 (ojc1)    0.996    0.059   16.816    0.000    0.547    0.694
##     s200_.7 (ojc2)    1.243    0.074   16.838    0.000    0.683    0.852
##     s200_.7 (ojc3)    1.245    0.080   15.576    0.000    0.684    0.849
##     s200_.7 (ojc4)    1.151    0.075   15.255    0.000    0.632    0.834
##     s200_.7 (ojc5)    1.107    0.077   14.346    0.000    0.608    0.743
##   dojc2 =~                                                              
##     OJC_W2            1.000                               0.670    0.670
##   dojc3 =~                                                              
##     OJC_W3            1.000                               0.922    0.922
##   dojc4 =~                                                              
##     OJC_W4            1.000                               0.701    0.701
##   SRH_W1 =~                                                             
##     s38.3r            1.000                               0.606    0.859
##   SRH_W2 =~                                                             
##     s38.4r            1.000                               0.564    0.842
##   SRH_W3 =~                                                             
##     s38.6             1.000                               0.614    0.862
##   SRH_W4 =~                                                             
##     s38.7             1.000                               0.838    0.918
## 
## Regressions:
##                    Estimate  Std.Err  z-value  P(>|z|)   Std.lv  Std.all
##   JC_W2 ~                                                               
##     JC_W1             1.000                               0.981    0.981
##   JC_W3 ~                                                               
##     JC_W2             1.000                               0.981    0.981
##   JC_W4 ~                                                               
##     JC_W3             1.000                               1.004    1.004
##   djc2 ~                                                                
##     JC_W1            -0.173    0.099   -1.750    0.080   -0.279   -0.279
##   djc3 ~                                                                
##     JC_W2             0.004    0.129    0.028    0.978    0.007    0.007
##   djc4 ~                                                                
##     JC_W3            -0.116    0.217   -0.535    0.593   -0.125   -0.125
##   OJC_W2 ~                                                              
##     OJC_W1            1.000                               0.961    0.961
##   OJC_W3 ~                                                              
##     OJC_W2            1.000                               0.982    0.982
##   OJC_W4 ~                                                              
##     OJC_W3            1.000                               1.008    1.008
##   dojc2 ~                                                               
##     OJC_W1           -0.201    0.094   -2.147    0.032   -0.289   -0.289
##   dojc3 ~                                                               
##     OJC_W2            0.034    0.153    0.221    0.825    0.036    0.036
##   dojc4 ~                                                               
##     OJC_W3            0.051    0.165    0.312    0.755    0.074    0.074
##   SRH_W2 ~                                                              
##     SRH_W1            1.009    0.132    7.672    0.000    1.084    1.084
##   SRH_W3 ~                                                              
##     SRH_W2            0.970    0.180    5.391    0.000    0.891    0.891
##   SRH_W4 ~                                                              
##     SRH_W3            1.309    0.228    5.747    0.000    0.959    0.959
##   SRH_W2 ~                                                              
##     djc2             -0.181    0.259   -0.699    0.484   -0.068   -0.068
##     dojc2             0.015    0.138    0.108    0.914    0.010    0.010
##     c137.6           -0.099    0.214   -0.460    0.646   -0.175   -0.064
##   SRH_W3 ~                                                              
##     djc3              0.920    0.694    1.326    0.185    0.267    0.267
##     dojc3             0.005    0.177    0.031    0.976    0.005    0.005
##     c137.6            0.163    0.317    0.514    0.607    0.265    0.097
##   SRH_W4 ~                                                              
##     djc4              0.516    0.324    1.594    0.111    0.202    0.202
##     dojc4             0.697    0.342    2.040    0.041    0.321    0.321
##     c137.6           -0.005    0.310   -0.018    0.986   -0.007   -0.002
## 
## Covariances:
##                    Estimate  Std.Err  z-value  P(>|z|)   Std.lv  Std.all
##  .s21_rd.3 ~~                                                           
##    .s21_rd.4          0.109    0.039    2.784    0.005    0.109    0.376
##    .s21_rd.6          0.168    0.050    3.331    0.001    0.168    0.521
##    .s21_rd.7          0.137    0.049    2.769    0.006    0.137    0.454
##  .s21_rd.4 ~~                                                           
##    .s21_rd.6          0.119    0.041    2.900    0.004    0.119    0.409
##    .s21_rd.7          0.002    0.048    0.052    0.959    0.002    0.009
##  .s21_rd.6 ~~                                                           
##    .s21_rd.7          0.151    0.052    2.920    0.003    0.151    0.501
##  .s21_str.3 ~~                                                          
##    .s21_str.4         0.162    0.037    4.398    0.000    0.162    0.595
##    .s21_str.6         0.207    0.057    3.659    0.000    0.207    0.613
##    .s21_str.7         0.201    0.047    4.287    0.000    0.201    0.671
##  .s21_str.4 ~~                                                          
##    .s21_str.6         0.171    0.044    3.891    0.000    0.171    0.566
##    .s21_str.7         0.196    0.040    4.852    0.000    0.196    0.729
##  .s21_str.6 ~~                                                          
##    .s21_str.7         0.231    0.053    4.341    0.000    0.231    0.692
##  .s21_cd.3 ~~                                                           
##    .s21_cd.4          0.235    0.069    3.431    0.001    0.235    0.695
##    .s21_cd.6          0.238    0.077    3.079    0.002    0.238    0.660
##    .s21_cd.7         -0.027    0.071   -0.380    0.704   -0.027   -0.076
##  .s21_cd.4 ~~                                                           
##    .s21_cd.6          0.234    0.078    3.006    0.003    0.234    0.598
##    .s21_cd.7          0.188    0.080    2.360    0.018    0.188    0.488
##  .s21_cd.6 ~~                                                           
##    .s21_cd.7          0.198    0.090    2.207    0.027    0.198    0.482
##  .s21_soz.3 ~~                                                          
##    .s21_soz.4         0.164    0.047    3.510    0.000    0.164    0.637
##    .s21_soz.6         0.178    0.051    3.483    0.000    0.178    0.698
##    .s21_soz.7         0.146    0.043    3.381    0.001    0.146    0.647
##  .s21_soz.4 ~~                                                          
##    .s21_soz.6         0.158    0.049    3.224    0.001    0.158    0.601
##    .s21_soz.7         0.158    0.046    3.408    0.001    0.158    0.680
##  .s21_soz.6 ~~                                                          
##    .s21_soz.7         0.219    0.051    4.293    0.000    0.219    0.946
##  .djc2 ~~                                                               
##    .djc3             -0.014    0.012   -1.142    0.254   -0.383   -0.383
##    .djc4             -0.008    0.017   -0.476    0.634   -0.126   -0.126
##  .djc3 ~~                                                               
##    .djc4             -0.032    0.017   -1.918    0.055   -0.572   -0.572
##  .s200_de.3 ~~                                                          
##    .s200_re.3         0.181    0.059    3.056    0.002    0.181    0.360
##  .s200_de.4 ~~                                                          
##    .s200_re.4         0.079    0.037    2.125    0.034    0.079    0.189
##  .s200_de.6 ~~                                                          
##    .s200_re.6         0.073    0.037    1.960    0.050    0.073    0.222
##  .s200_de.7 ~~                                                          
##    .s200_re.7         0.024    0.045    0.528    0.597    0.024    0.065
##  .s200_de.3 ~~                                                          
##    .s200_de.4         0.188    0.060    3.150    0.002    0.188    0.349
##    .s200_de.6         0.132    0.062    2.133    0.033    0.132    0.272
##    .s200_de.7         0.065    0.069    0.949    0.343    0.065    0.131
##  .s200_de.4 ~~                                                          
##    .s200_de.6         0.152    0.054    2.802    0.005    0.152    0.345
##    .s200_de.7         0.242    0.064    3.765    0.000    0.242    0.535
##  .s200_de.6 ~~                                                          
##    .s200_de.7         0.253    0.067    3.778    0.000    0.253    0.622
##  .s200_re.3 ~~                                                          
##    .s200_re.4         0.197    0.047    4.188    0.000    0.197    0.505
##    .s200_re.6         0.142    0.048    2.934    0.003    0.142    0.414
##    .s200_re.7         0.123    0.062    1.996    0.046    0.123    0.332
##  .s200_re.4 ~~                                                          
##    .s200_re.6         0.108    0.043    2.510    0.012    0.108    0.344
##    .s200_re.7         0.099    0.055    1.800    0.072    0.099    0.292
##  .s200_re.6 ~~                                                          
##    .s200_re.7         0.136    0.054    2.537    0.011    0.136    0.457
##  .s200_au.3 ~~                                                          
##    .s200_au.4         0.063    0.034    1.859    0.063    0.063    0.323
##    .s200_au.6        -0.010    0.032   -0.299    0.765   -0.010   -0.069
##    .s200_au.7        -0.084    0.048   -1.755    0.079   -0.084   -0.412
##  .s200_au.4 ~~                                                          
##    .s200_au.6         0.040    0.025    1.631    0.103    0.040    0.346
##    .s200_au.7         0.050    0.039    1.269    0.204    0.050    0.292
##  .s200_au.6 ~~                                                          
##    .s200_au.7         0.019    0.033    0.572    0.567    0.019    0.154
##  .s200_ma.3 ~~                                                          
##    .s200_ma.4         0.139    0.051    2.738    0.006    0.139    0.423
##    .s200_ma.6         0.060    0.049    1.227    0.220    0.060    0.191
##    .s200_ma.7         0.010    0.047    0.216    0.829    0.010    0.039
##  .s200_ma.4 ~~                                                          
##    .s200_ma.6         0.085    0.039    2.200    0.028    0.085    0.317
##    .s200_ma.7         0.073    0.040    1.836    0.066    0.073    0.321
##  .s200_ma.6 ~~                                                          
##    .s200_ma.7         0.135    0.049    2.722    0.006    0.135    0.625
##  .s200_me.3 ~~                                                          
##    .s200_me.4         0.060    0.029    2.038    0.042    0.060    0.296
##    .s200_me.6         0.085    0.041    2.044    0.041    0.085    0.380
##    .s200_me.7         0.128    0.045    2.815    0.005    0.128    0.600
##  .s200_me.4 ~~                                                          
##    .s200_me.6         0.047    0.033    1.433    0.152    0.047    0.272
##    .s200_me.7         0.086    0.034    2.578    0.010    0.086    0.522
##  .s200_me.6 ~~                                                          
##    .s200_me.7         0.086    0.039    2.220    0.026    0.086    0.468
##  .s200_af.3 ~~                                                          
##    .s200_af.4         0.069    0.039    1.759    0.079    0.069    0.244
##    .s200_af.6         0.092    0.050    1.856    0.063    0.092    0.330
##    .s200_af.7         0.130    0.061    2.123    0.034    0.130    0.443
##  .s200_af.4 ~~                                                          
##    .s200_af.6         0.021    0.043    0.502    0.615    0.021    0.078
##    .s200_af.7         0.090    0.051    1.744    0.081    0.090    0.311
##  .s200_af.6 ~~                                                          
##    .s200_af.7         0.148    0.054    2.749    0.006    0.148    0.520
##  .dojc2 ~~                                                              
##    .dojc3            -0.135    0.042   -3.227    0.001   -0.745   -0.745
##    .dojc4            -0.001    0.031   -0.029    0.977   -0.006   -0.006
##  .dojc3 ~~                                                              
##    .dojc4            -0.091    0.045   -2.012    0.044   -0.445   -0.445
##  .djc2 ~~                                                               
##    .dojc2             0.010    0.009    1.043    0.297    0.134    0.134
##  .djc3 ~~                                                               
##    .dojc3             0.034    0.015    2.262    0.024    0.362    0.362
##  .djc4 ~~                                                               
##    .dojc4            -0.015    0.020   -0.747    0.455   -0.119   -0.119
##   JC_W1 ~~                                                              
##     c137.6            0.028    0.019    1.528    0.126    0.084    0.229
##   OJC_W1 ~~                                                             
##     c137.6            0.023    0.026    0.866    0.386    0.043    0.118
##   SRH_W1 ~~                                                             
##     c137.6           -0.104    0.039   -2.660    0.008   -0.171   -0.468
##  .djc2 ~~                                                               
##     c137.6           -0.012    0.011   -1.164    0.245   -0.060   -0.165
##  .djc3 ~~                                                               
##     c137.6           -0.017    0.012   -1.483    0.138   -0.096   -0.262
##  .djc4 ~~                                                               
##     c137.6            0.032    0.018    1.786    0.074    0.103    0.282
##  .dojc2 ~~                                                              
##     c137.6            0.000    0.018    0.025    0.980    0.001    0.003
##  .dojc3 ~~                                                              
##     c137.6           -0.033    0.026   -1.272    0.203   -0.063   -0.173
##  .dojc4 ~~                                                              
##     c137.6            0.016    0.020    0.784    0.433    0.040    0.109
##   JC_W1 ~~                                                              
##     OJC_W1            0.069    0.027    2.584    0.010    0.387    0.387
##     SRH_W1            0.023    0.026    0.898    0.369    0.111    0.111
##   OJC_W1 ~~                                                             
##     SRH_W1            0.121    0.041    2.963    0.003    0.381    0.381
## 
## Intercepts:
##                    Estimate  Std.Err  z-value  P(>|z|)   Std.lv  Std.all
##     JC_W1             3.101    0.074   41.982    0.000    9.102    9.102
##    .JC_W2             0.000                               0.000    0.000
##    .JC_W3             0.000                               0.000    0.000
##    .JC_W4             0.000                               0.000    0.000
##    .djc2              0.325    0.313    1.036    0.300    1.534    1.534
##    .djc3              0.189    0.379    0.499    0.618    1.061    1.061
##    .djc4              0.273    0.670    0.407    0.684    0.832    0.832
##    .s21_rd.3          0.000                               0.000    0.000
##    .s21_rd.4          0.000                               0.000    0.000
##    .s21_rd.6          0.000                               0.000    0.000
##    .s21_rd.7          0.000                               0.000    0.000
##     OJC_W1            3.823    0.104   36.643    0.000    7.314    7.314
##    .OJC_W2            0.000                               0.000    0.000
##    .OJC_W3            0.000                               0.000    0.000
##    .OJC_W4            0.000                               0.000    0.000
##    .dojc2             0.772    0.370    2.089    0.037    2.121    2.121
##    .dojc3            -0.345    0.592   -0.584    0.559   -0.677   -0.677
##    .dojc4            -0.112    0.601   -0.187    0.852   -0.292   -0.292
##    .s200_de.3         0.000                               0.000    0.000
##    .s200_de.4         0.000                               0.000    0.000
##    .s200_de.6         0.000                               0.000    0.000
##    .s200_de.7         0.000                               0.000    0.000
##    .s38.3r            0.000                               0.000    0.000
##    .s38.4r            0.000                               0.000    0.000
##    .s38.6             0.000                               0.000    0.000
##    .s38.7             0.000                               0.000    0.000
##     SRH_W1            3.591    0.074   48.724    0.000    5.922    5.922
##    .SRH_W2            0.021    0.500    0.042    0.967    0.037    0.037
##    .SRH_W3            0.012    0.733    0.016    0.987    0.019    0.019
##    .SRH_W4           -1.418    0.899   -1.577    0.115   -1.692   -1.692
##    .s21_str.3         1.261    0.480    2.628    0.009    1.261    2.029
##    .s21_cd.3         -3.445    0.828   -4.159    0.000   -3.445   -3.930
##    .s21_soz.3        -1.907    0.492   -3.876    0.000   -1.907   -2.649
##    .s21_str.4         1.360    0.444    3.062    0.002    1.360    2.365
##    .s21_cd.4         -3.094    0.768   -4.030    0.000   -3.094   -3.373
##    .s21_soz.4        -1.685    0.454   -3.713    0.000   -1.685   -2.284
##    .s21_str.6         1.161    0.479    2.425    0.015    1.161    1.698
##    .s21_cd.6         -3.412    0.826   -4.133    0.000   -3.412   -3.579
##    .s21_soz.6        -1.949    0.488   -3.992    0.000   -1.949   -2.622
##    .s21_str.7         1.201    0.469    2.563    0.010    1.201    1.933
##    .s21_cd.7         -3.480    0.807   -4.311    0.000   -3.480   -3.678
##    .s21_soz.7        -2.079    0.479   -4.340    0.000   -2.079   -2.961
##    .s200_re.3        -0.191    0.244   -0.782    0.434   -0.191   -0.228
##    .s200_au.3        -1.039    0.308   -3.376    0.001   -1.039   -1.283
##    .s200_ma.3        -1.207    0.332   -3.629    0.000   -1.207   -1.342
##    .s200_me.3        -0.761    0.312   -2.443    0.015   -0.761   -0.966
##    .s200_af.3        -0.321    0.317   -1.013    0.311   -0.321   -0.407
##    .s200_re.4        -0.179    0.241   -0.743    0.458   -0.179   -0.222
##    .s200_au.4        -0.928    0.298   -3.121    0.002   -0.928   -1.179
##    .s200_ma.4        -1.273    0.322   -3.957    0.000   -1.273   -1.480
##    .s200_me.4        -0.708    0.302   -2.348    0.019   -0.708   -0.957
##    .s200_af.4        -0.479    0.309   -1.549    0.121   -0.479   -0.598
##    .s200_re.6        -0.177    0.230   -0.773    0.440   -0.177   -0.233
##    .s200_au.6        -0.904    0.284   -3.184    0.001   -0.904   -1.212
##    .s200_ma.6        -1.165    0.309   -3.771    0.000   -1.165   -1.362
##    .s200_me.6        -0.658    0.290   -2.267    0.023   -0.658   -0.851
##    .s200_af.6        -0.389    0.297   -1.308    0.191   -0.389   -0.483
##    .s200_re.7        -0.173    0.244   -0.707    0.479   -0.173   -0.219
##    .s200_au.7        -0.963    0.300   -3.214    0.001   -0.963   -1.200
##    .s200_ma.7        -1.237    0.317   -3.901    0.000   -1.237   -1.534
##    .s200_me.7        -0.731    0.300   -2.435    0.015   -0.731   -0.964
##    .s200_af.7        -0.429    0.308   -1.389    0.165   -0.429   -0.524
##     c137.6            0.174    0.045    3.908    0.000    0.174    0.476
## 
## Variances:
##                    Estimate  Std.Err  z-value  P(>|z|)   Std.lv  Std.all
##     JC_W1             0.116    0.033    3.499    0.000    1.000    1.000
##    .JC_W2             0.000                               0.000    0.000
##    .JC_W3             0.000                               0.000    0.000
##    .JC_W4             0.000                               0.000    0.000
##    .djc2              0.041    0.014    3.011    0.003    0.922    0.922
##    .djc3              0.032    0.014    2.249    0.025    1.003    1.003
##    .djc4              0.097    0.036    2.651    0.008    0.899    0.899
##     OJC_W1            0.273    0.057    4.830    0.000    1.000    1.000
##    .OJC_W2            0.000                               0.000    0.000
##    .OJC_W3            0.000                               0.000    0.000
##    .OJC_W4            0.000                               0.000    0.000
##    .dojc2             0.122    0.029    4.185    0.000    0.916    0.916
##    .dojc3             0.269    0.077    3.513    0.000    1.034    1.034
##    .dojc4             0.157    0.057    2.741    0.006    1.058    1.058
##    .s38.3r  (rs_s)    0.131    0.015    8.987    0.000    0.131    0.262
##    .s38.4r  (rs_s)    0.131    0.015    8.987    0.000    0.131    0.291
##    .s38.6   (rs_s)    0.131    0.015    8.987    0.000    0.131    0.257
##    .s38.7   (rs_s)    0.131    0.015    8.987    0.000    0.131    0.157
##    .s21_r.3           0.322    0.058    5.534    0.000    0.322    0.735
##    .s21_s.3           0.302    0.054    5.584    0.000    0.302    0.783
##    .s21_c.3           0.313    0.082    3.808    0.000    0.313    0.407
##    .s21_s.3           0.249    0.056    4.445    0.000    0.249    0.481
##    .s21_r.4           0.261    0.042    6.188    0.000    0.261    0.684
##    .s21_s.4           0.243    0.038    6.337    0.000    0.243    0.737
##    .s21_c.4           0.367    0.082    4.478    0.000    0.367    0.436
##    .s21_s.4           0.265    0.054    4.858    0.000    0.265    0.486
##    .s21_r.6           0.321    0.061    5.283    0.000    0.321    0.719
##    .s21_s.6           0.377    0.072    5.256    0.000    0.377    0.806
##    .s21_c.6           0.416    0.107    3.886    0.000    0.416    0.457
##    .s21_s.6           0.262    0.062    4.254    0.000    0.262    0.474
##    .s21_r.7           0.282    0.064    4.418    0.000    0.282    0.694
##    .s21_s.7           0.297    0.058    5.108    0.000    0.297    0.767
##    .s21_c.7           0.406    0.122    3.330    0.001    0.406    0.453
##    .s21_s.7           0.204    0.055    3.696    0.000    0.204    0.414
##    .s200_.3           0.590    0.103    5.755    0.000    0.590    0.684
##    .s200_.3           0.428    0.075    5.710    0.000    0.428    0.612
##    .s200_.3           0.233    0.054    4.348    0.000    0.233    0.356
##    .s200_.3           0.385    0.077    5.035    0.000    0.385    0.476
##    .s200_.3           0.260    0.052    4.975    0.000    0.260    0.418
##    .s200_.3           0.288    0.056    5.114    0.000    0.288    0.462
##    .s200_.4           0.490    0.071    6.891    0.000    0.490    0.623
##    .s200_.4           0.356    0.053    6.681    0.000    0.356    0.548
##    .s200_.4           0.163    0.031    5.169    0.000    0.163    0.263
##    .s200_.4           0.281    0.048    5.892    0.000    0.281    0.380
##    .s200_.4           0.156    0.029    5.359    0.000    0.156    0.285
##    .s200_.4           0.277    0.044    6.288    0.000    0.277    0.433
##    .s200_.6           0.397    0.072    5.512    0.000    0.397    0.564
##    .s200_.6           0.275    0.050    5.446    0.000    0.275    0.475
##    .s200_.6           0.083    0.028    2.998    0.003    0.083    0.148
##    .s200_.6           0.255    0.053    4.857    0.000    0.255    0.350
##    .s200_.6           0.192    0.044    4.406    0.000    0.192    0.321
##    .s200_.6           0.271    0.057    4.771    0.000    0.271    0.419
##    .s200_.7           0.417    0.091    4.608    0.000    0.417    0.580
##    .s200_.7           0.322    0.077    4.196    0.000    0.322    0.518
##    .s200_.7           0.177    0.055    3.210    0.001    0.177    0.275
##    .s200_.7           0.182    0.054    3.347    0.001    0.182    0.280
##    .s200_.7           0.175    0.047    3.714    0.000    0.175    0.305
##    .s200_.7           0.300    0.073    4.077    0.000    0.300    0.447
##     c137.6            0.134    0.024    5.655    0.000    0.134    1.000
##     SRH_W1            0.368    0.073    5.044    0.000    1.000    1.000
##    .SRH_W2           -0.080    0.033   -2.424    0.015   -0.250   -0.250
##    .SRH_W3            0.080    0.041    1.961    0.050    0.214    0.214
##    .SRH_W4           -0.008    0.088   -0.095    0.924   -0.012   -0.012
```

```
parameterEstimates(lcsm_parcel_SRH_GC3, standardized = TRUE, remove.nonfree = TRUE, output = "pretty")
```

```
## 
## 
## Group 1 []:
## 
## Latent Variables:
##                    Estimate  Std.Err  z-value  P(>|z|) ci.lower ci.upper
##   JC_W1 =~                                                              
##     s21_s.3 (j1_1)    1.146    0.136    8.442    0.000    0.880    1.413
##     s21_c.3 (j2_1)    1.593    0.186    8.542    0.000    1.227    1.958
##     s21_s.3  (jc3)    1.523    0.154    9.891    0.000    1.221    1.824
##   JC_W2 =~                                                              
##     s21_s.4 (j1_1)    1.146    0.136    8.442    0.000    0.880    1.413
##     s21_c.4 (j2_1)    1.593    0.186    8.542    0.000    1.227    1.958
##     s21_s.4  (jc3)    1.523    0.154    9.891    0.000    1.221    1.824
##   JC_W3 =~                                                              
##     s21_s.6 (j1_1)    1.146    0.136    8.442    0.000    0.880    1.413
##     s21_c.6 (j2_1)    1.593    0.186    8.542    0.000    1.227    1.958
##     s21_s.6  (jc3)    1.523    0.154    9.891    0.000    1.221    1.824
##   JC_W4 =~                                                              
##     s21_s.7 (j1_1)    1.146    0.136    8.442    0.000    0.880    1.413
##     s21_c.7 (j2_1)    1.593    0.186    8.542    0.000    1.227    1.958
##     s21_s.7  (jc3)    1.523    0.154    9.891    0.000    1.221    1.824
##   OJC_W1 =~                                                             
##     s200_.3 (ojc1)    0.996    0.059   16.816    0.000    0.880    1.112
##     s200_.3 (ojc2)    1.243    0.074   16.838    0.000    1.098    1.388
##     s200_.3 (ojc3)    1.245    0.080   15.576    0.000    1.088    1.402
##     s200_.3 (ojc4)    1.151    0.075   15.255    0.000    1.003    1.299
##     s200_.3 (ojc5)    1.107    0.077   14.346    0.000    0.956    1.259
##   OJC_W2 =~                                                             
##     s200_.4 (ojc1)    0.996    0.059   16.816    0.000    0.880    1.112
##     s200_.4 (ojc2)    1.243    0.074   16.838    0.000    1.098    1.388
##     s200_.4 (ojc3)    1.245    0.080   15.576    0.000    1.088    1.402
##     s200_.4 (ojc4)    1.151    0.075   15.255    0.000    1.003    1.299
##     s200_.4 (ojc5)    1.107    0.077   14.346    0.000    0.956    1.259
##   OJC_W3 =~                                                             
##     s200_.6 (ojc1)    0.996    0.059   16.816    0.000    0.880    1.112
##     s200_.6 (ojc2)    1.243    0.074   16.838    0.000    1.098    1.388
##     s200_.6 (ojc3)    1.245    0.080   15.576    0.000    1.088    1.402
##     s200_.6 (ojc4)    1.151    0.075   15.255    0.000    1.003    1.299
##     s200_.6 (ojc5)    1.107    0.077   14.346    0.000    0.956    1.259
##   OJC_W4 =~                                                             
##     s200_.7 (ojc1)    0.996    0.059   16.816    0.000    0.880    1.112
##     s200_.7 (ojc2)    1.243    0.074   16.838    0.000    1.098    1.388
##     s200_.7 (ojc3)    1.245    0.080   15.576    0.000    1.088    1.402
##     s200_.7 (ojc4)    1.151    0.075   15.255    0.000    1.003    1.299
##     s200_.7 (ojc5)    1.107    0.077   14.346    0.000    0.956    1.259
##    Std.lv  Std.all
##                   
##     0.355    0.591
##     0.493    0.577
##     0.471    0.739
##                   
##     0.391    0.604
##     0.544    0.627
##     0.520    0.797
##                   
##     0.389    0.608
##     0.541    0.617
##     0.517    0.777
##                   
##     0.410    0.587
##     0.570    0.649
##     0.544    0.853
##                   
##     0.471    0.575
##     0.587    0.799
##     0.588    0.741
##     0.544    0.727
##     0.523    0.632
##                   
##     0.557    0.664
##     0.695    0.825
##     0.696    0.812
##     0.644    0.782
##     0.619    0.670
##                   
##     0.520    0.675
##     0.649    0.833
##     0.650    0.839
##     0.601    0.788
##     0.578    0.711
##                   
##     0.486    0.651
##     0.607    0.833
##     0.608    0.801
##     0.562    0.715
##     0.540    0.685
## 
## Regressions:
##                    Estimate  Std.Err  z-value  P(>|z|) ci.lower ci.upper
##   djc2 ~                                                                
##     JC_W1            -0.049    0.071   -0.690    0.490   -0.188    0.090
##   djc3 ~                                                                
##     JC_W2            -0.044    0.078   -0.570    0.569   -0.196    0.108
##   djc4 ~                                                                
##     JC_W3             0.070    0.097    0.721    0.471   -0.121    0.261
##   dojc2 ~                                                               
##     OJC_W1           -0.147    0.074   -1.969    0.049   -0.293   -0.001
##   dojc3 ~                                                               
##     OJC_W2           -0.063    0.096   -0.652    0.514   -0.251    0.126
##   dojc4 ~                                                               
##     OJC_W3           -0.054    0.101   -0.533    0.594   -0.252    0.144
##   SRH_W2 ~                                                              
##     SRH_W1            0.833    0.067   12.353    0.000    0.701    0.966
##   SRH_W3 ~                                                              
##     SRH_W2            0.905    0.069   13.030    0.000    0.769    1.041
##   SRH_W4 ~                                                              
##     SRH_W3            1.043    0.086   12.135    0.000    0.875    1.212
##   SRH_W2 ~                                                              
##     djc2              0.352    0.306    1.154    0.249   -0.246    0.951
##     dojc2             0.278    0.107    2.589    0.010    0.067    0.488
##     c137.6           -0.108    0.106   -1.022    0.307   -0.315    0.099
##   SRH_W3 ~                                                              
##     djc3              0.419    0.278    1.509    0.131   -0.125    0.964
##     dojc3             0.102    0.092    1.107    0.268   -0.078    0.282
##     c137.6            0.053    0.103    0.519    0.604   -0.148    0.255
##   SRH_W4 ~                                                              
##     djc4              0.337    0.302    1.115    0.265   -0.255    0.929
##     dojc4             0.378    0.145    2.602    0.009    0.093    0.662
##     c137.6            0.105    0.109    0.962    0.336   -0.109    0.318
##    Std.lv  Std.all
##                   
##    -0.087   -0.087
##                   
##    -0.082   -0.082
##                   
##     0.119    0.119
##                   
##    -0.176   -0.176
##                   
##    -0.084   -0.084
##                   
##    -0.084   -0.084
##                   
##     0.899    0.899
##                   
##     0.992    0.992
##                   
##     1.070    1.070
##                   
##     0.089    0.089
##     0.158    0.158
##    -0.156   -0.074
##                   
##     0.122    0.122
##     0.068    0.068
##     0.085    0.040
##                   
##     0.110    0.110
##     0.207    0.207
##     0.170    0.080
## 
## Covariances:
##                    Estimate  Std.Err  z-value  P(>|z|) ci.lower ci.upper
##   s21_rd.3 ~~                                                           
##     s21_rd.4          0.188    0.033    5.704    0.000    0.123    0.252
##     s21_rd.6          0.198    0.035    5.703    0.000    0.130    0.266
##     s21_rd.7          0.233    0.039    5.933    0.000    0.156    0.310
##   s21_rd.4 ~~                                                           
##     s21_rd.6          0.214    0.032    6.763    0.000    0.152    0.276
##     s21_rd.7          0.215    0.036    5.990    0.000    0.144    0.285
##   s21_rd.6 ~~                                                           
##     s21_rd.7          0.211    0.037    5.749    0.000    0.139    0.283
##  .s21_str.3 ~~                                                          
##    .s21_str.4         0.151    0.023    6.463    0.000    0.105    0.197
##    .s21_str.6         0.153    0.025    6.055    0.000    0.103    0.202
##    .s21_str.7         0.188    0.029    6.513    0.000    0.132    0.245
##  .s21_str.4 ~~                                                          
##    .s21_str.6         0.172    0.025    6.875    0.000    0.123    0.222
##    .s21_str.7         0.181    0.029    6.271    0.000    0.125    0.238
##  .s21_str.6 ~~                                                          
##    .s21_str.7         0.192    0.030    6.443    0.000    0.134    0.251
##  .s21_cd.3 ~~                                                           
##    .s21_cd.4          0.300    0.045    6.596    0.000    0.211    0.389
##    .s21_cd.6          0.366    0.051    7.191    0.000    0.266    0.465
##    .s21_cd.7          0.312    0.051    6.156    0.000    0.213    0.412
##  .s21_cd.4 ~~                                                           
##    .s21_cd.6          0.320    0.047    6.805    0.000    0.228    0.412
##    .s21_cd.7          0.285    0.048    5.975    0.000    0.191    0.378
##  .s21_cd.6 ~~                                                           
##    .s21_cd.7          0.331    0.052    6.311    0.000    0.228    0.434
##  .s21_soz.3 ~~                                                          
##    .s21_soz.4         0.093    0.025    3.747    0.000    0.044    0.142
##    .s21_soz.6         0.077    0.026    2.945    0.003    0.026    0.128
##    .s21_soz.7         0.054    0.025    2.187    0.029    0.006    0.103
##  .s21_soz.4 ~~                                                          
##    .s21_soz.6         0.079    0.024    3.280    0.001    0.032    0.126
##    .s21_soz.7         0.056    0.025    2.278    0.023    0.008    0.105
##  .s21_soz.6 ~~                                                          
##    .s21_soz.7         0.055    0.025    2.196    0.028    0.006    0.103
##  .djc2 ~~                                                               
##    .djc3             -0.012    0.007   -1.704    0.088   -0.027    0.002
##    .djc4              0.003    0.007    0.358    0.720   -0.011    0.017
##  .djc3 ~~                                                               
##    .djc4             -0.024    0.009   -2.794    0.005   -0.041   -0.007
##   s200_de.3 ~~                                                          
##    .s200_re.3         0.073    0.031    2.379    0.017    0.013    0.133
##   s200_de.4 ~~                                                          
##    .s200_re.4         0.157    0.031    5.046    0.000    0.096    0.218
##   s200_de.6 ~~                                                          
##    .s200_re.6         0.133    0.030    4.427    0.000    0.074    0.192
##   s200_de.7 ~~                                                          
##    .s200_re.7         0.082    0.030    2.702    0.007    0.022    0.141
##   s200_de.3 ~~                                                          
##     s200_de.4         0.273    0.045    6.055    0.000    0.185    0.362
##     s200_de.6         0.225    0.042    5.352    0.000    0.142    0.307
##     s200_de.7         0.276    0.048    5.778    0.000    0.182    0.369
##   s200_de.4 ~~                                                          
##     s200_de.6         0.213    0.041    5.225    0.000    0.133    0.294
##     s200_de.7         0.274    0.046    6.009    0.000    0.185    0.363
##   s200_de.6 ~~                                                          
##     s200_de.7         0.256    0.046    5.621    0.000    0.167    0.345
##  .s200_re.3 ~~                                                          
##    .s200_re.4         0.156    0.033    4.679    0.000    0.091    0.222
##    .s200_re.6         0.141    0.032    4.338    0.000    0.077    0.204
##    .s200_re.7         0.173    0.036    4.770    0.000    0.102    0.244
##  .s200_re.4 ~~                                                          
##    .s200_re.6         0.140    0.027    5.141    0.000    0.086    0.193
##    .s200_re.7         0.129    0.030    4.310    0.000    0.070    0.187
##  .s200_re.6 ~~                                                          
##    .s200_re.7         0.113    0.032    3.539    0.000    0.050    0.175
##  .s200_au.3 ~~                                                          
##    .s200_au.4         0.051    0.021    2.376    0.017    0.009    0.093
##    .s200_au.6         0.049    0.021    2.268    0.023    0.007    0.090
##    .s200_au.7         0.057    0.023    2.457    0.014    0.011    0.102
##  .s200_au.4 ~~                                                          
##    .s200_au.6         0.060    0.022    2.775    0.006    0.018    0.102
##    .s200_au.7         0.057    0.022    2.638    0.008    0.015    0.100
##  .s200_au.6 ~~                                                          
##    .s200_au.7         0.067    0.024    2.844    0.004    0.021    0.113
##  .s200_ma.3 ~~                                                          
##    .s200_ma.4         0.073    0.025    2.874    0.004    0.023    0.122
##    .s200_ma.6         0.112    0.025    4.517    0.000    0.064    0.161
##    .s200_ma.7         0.104    0.028    3.663    0.000    0.048    0.160
##  .s200_ma.4 ~~                                                          
##    .s200_ma.6         0.056    0.023    2.406    0.016    0.010    0.101
##    .s200_ma.7         0.079    0.027    2.898    0.004    0.026    0.133
##  .s200_ma.6 ~~                                                          
##    .s200_ma.7         0.068    0.025    2.792    0.005    0.020    0.117
##  .s200_me.3 ~~                                                          
##    .s200_me.4         0.076    0.025    3.049    0.002    0.027    0.124
##    .s200_me.6         0.060    0.025    2.425    0.015    0.012    0.109
##    .s200_me.7         0.104    0.030    3.420    0.001    0.044    0.164
##  .s200_me.4 ~~                                                          
##    .s200_me.6         0.066    0.023    2.854    0.004    0.021    0.111
##    .s200_me.7         0.080    0.029    2.772    0.006    0.023    0.136
##  .s200_me.6 ~~                                                          
##    .s200_me.7         0.105    0.030    3.464    0.001    0.045    0.164
##  .s200_af.3 ~~                                                          
##    .s200_af.4         0.182    0.037    4.883    0.000    0.109    0.255
##    .s200_af.6         0.127    0.036    3.481    0.000    0.055    0.198
##    .s200_af.7         0.115    0.037    3.095    0.002    0.042    0.189
##  .s200_af.4 ~~                                                          
##    .s200_af.6         0.134    0.036    3.753    0.000    0.064    0.203
##    .s200_af.7         0.178    0.039    4.537    0.000    0.101    0.255
##  .s200_af.6 ~~                                                          
##    .s200_af.7         0.180    0.038    4.783    0.000    0.106    0.254
##  .dojc2 ~~                                                              
##    .dojc3            -0.089    0.026   -3.370    0.001   -0.140   -0.037
##    .dojc4            -0.009    0.019   -0.482    0.630   -0.045    0.027
##  .dojc3 ~~                                                              
##    .dojc4            -0.051    0.020   -2.483    0.013   -0.091   -0.011
##  .djc2 ~~                                                               
##    .dojc2             0.017    0.007    2.568    0.010    0.004    0.030
##  .djc3 ~~                                                               
##    .dojc3             0.006    0.006    0.989    0.323   -0.005    0.017
##  .djc4 ~~                                                               
##    .dojc4            -0.009    0.007   -1.265    0.206   -0.023    0.005
##   JC_W1 ~~                                                              
##     c137.6           -0.017    0.014   -1.235    0.217   -0.044    0.010
##   OJC_W1 ~~                                                             
##     c137.6            0.026    0.019    1.388    0.165   -0.011    0.063
##   SRH_W1 ~~                                                             
##     c137.6           -0.146    0.031   -4.638    0.000   -0.207   -0.084
##  .djc2 ~~                                                               
##     c137.6           -0.001    0.009   -0.084    0.933   -0.019    0.017
##  .djc3 ~~                                                               
##     c137.6           -0.016    0.009   -1.789    0.074   -0.033    0.002
##  .djc4 ~~                                                               
##     c137.6           -0.005    0.011   -0.402    0.688   -0.027    0.018
##  .dojc2 ~~                                                              
##     c137.6            0.011    0.016    0.687    0.492   -0.021    0.043
##  .dojc3 ~~                                                              
##     c137.6           -0.022    0.016   -1.348    0.178   -0.053    0.010
##  .dojc4 ~~                                                              
##     c137.6            0.002    0.014    0.134    0.894   -0.026    0.030
##   JC_W1 ~~                                                              
##     OJC_W1            0.069    0.015    4.443    0.000    0.038    0.099
##     SRH_W1            0.043    0.020    2.150    0.032    0.004    0.082
##   OJC_W1 ~~                                                             
##     SRH_W1            0.149    0.031    4.785    0.000    0.088    0.210
##    Std.lv  Std.all
##                   
##     0.188    0.478
##     0.198    0.535
##     0.233    0.580
##                   
##     0.214    0.609
##     0.215    0.563
##                   
##     0.211    0.587
##                   
##     0.151    0.605
##     0.153    0.621
##     0.188    0.688
##                   
##     0.172    0.657
##     0.181    0.621
##                   
##     0.192    0.670
##                   
##     0.300    0.635
##     0.366    0.759
##     0.312    0.669
##                   
##     0.320    0.686
##     0.285    0.629
##                   
##     0.331    0.718
##                   
##     0.093    0.551
##     0.077    0.427
##     0.054    0.382
##                   
##     0.079    0.477
##     0.056    0.429
##                   
##     0.055    0.392
##                   
##    -0.398   -0.398
##     0.072    0.072
##                   
##    -0.653   -0.653
##                   
##     0.073    0.155
##                   
##     0.157    0.319
##                   
##     0.133    0.337
##                   
##     0.082    0.205
##                   
##     0.273    0.496
##     0.225    0.461
##     0.276    0.557
##                   
##     0.213    0.391
##     0.274    0.495
##                   
##     0.256    0.524
##                   
##     0.156    0.372
##     0.141    0.370
##     0.173    0.455
##                   
##     0.140    0.392
##     0.129    0.362
##                   
##     0.113    0.350
##                   
##     0.051    0.242
##     0.049    0.255
##     0.057    0.319
##                   
##     0.060    0.291
##     0.057    0.299
##                   
##     0.067    0.385
##                   
##     0.073    0.272
##     0.112    0.499
##     0.104    0.430
##                   
##     0.056    0.263
##     0.079    0.348
##                   
##     0.068    0.357
##                   
##     0.076    0.287
##     0.060    0.250
##     0.104    0.369
##                   
##     0.066    0.274
##     0.080    0.283
##                   
##     0.105    0.406
##                   
##     0.182    0.413
##     0.127    0.346
##     0.115    0.313
##                   
##     0.134    0.341
##     0.178    0.451
##                   
##     0.180    0.549
##                   
##    -0.565   -0.565
##    -0.071   -0.071
##                   
##    -0.385   -0.385
##                   
##     0.249    0.249
##                   
##     0.076    0.076
##                   
##    -0.132   -0.132
##                   
##    -0.055   -0.116
##                   
##     0.055    0.117
##                   
##    -0.195   -0.413
##                   
##    -0.004   -0.009
##                   
##    -0.087   -0.184
##                   
##    -0.022   -0.047
##                   
##     0.029    0.060
##                   
##    -0.054   -0.114
##                   
##     0.006    0.012
##                   
##     0.470    0.470
##     0.186    0.186
##                   
##     0.423    0.423
## 
## Intercepts:
##                    Estimate  Std.Err  z-value  P(>|z|) ci.lower ci.upper
##     JC_W1             2.931    0.050   58.304    0.000    2.832    3.029
##    .djc2              0.008    0.212    0.039    0.969   -0.407    0.424
##    .djc3              0.265    0.220    1.202    0.230   -0.167    0.696
##    .djc4             -0.325    0.289   -1.124    0.261   -0.893    0.242
##     OJC_W1            3.976    0.060   66.806    0.000    3.860    4.093
##    .dojc2             0.455    0.302    1.509    0.131   -0.136    1.046
##    .dojc3             0.200    0.376    0.532    0.595   -0.537    0.937
##    .dojc4             0.274    0.389    0.705    0.481   -0.489    1.037
##     SRH_W1            3.646    0.057   64.206    0.000    3.535    3.758
##    .SRH_W2            0.783    0.267    2.936    0.003    0.260    1.306
##    .SRH_W3            0.226    0.283    0.797    0.425   -0.329    0.781
##    .SRH_W4           -0.168    0.329   -0.509    0.611   -0.813    0.478
##    .s21_str.3         0.512    0.402    1.273    0.203   -0.276    1.301
##    .s21_cd.3         -2.228    0.553   -4.027    0.000   -3.312   -1.144
##    .s21_soz.3        -1.855    0.457   -4.057    0.000   -2.751   -0.959
##    .s21_str.4         0.553    0.384    1.442    0.149   -0.199    1.305
##    .s21_cd.4         -1.998    0.527   -3.793    0.000   -3.031   -0.966
##    .s21_soz.4        -1.746    0.435   -4.013    0.000   -2.599   -0.893
##    .s21_str.6         0.340    0.403    0.844    0.399   -0.450    1.130
##    .s21_cd.6         -2.182    0.554   -3.940    0.000   -3.267   -1.096
##    .s21_soz.6        -1.904    0.458   -4.161    0.000   -2.801   -1.007
##    .s21_str.7         0.347    0.389    0.892    0.372   -0.415    1.109
##    .s21_cd.7         -2.175    0.533   -4.079    0.000   -3.220   -1.130
##    .s21_soz.7        -1.869    0.440   -4.246    0.000   -2.731   -1.006
##    .s200_re.3        -0.110    0.244   -0.453    0.651   -0.588    0.367
##    .s200_au.3        -1.037    0.302   -3.435    0.001   -1.629   -0.445
##    .s200_ma.3        -1.397    0.326   -4.281    0.000   -2.036   -0.757
##    .s200_me.3        -0.890    0.308   -2.889    0.004   -1.493   -0.286
##    .s200_af.3        -0.484    0.315   -1.536    0.124   -1.102    0.134
##    .s200_re.4        -0.114    0.234   -0.486    0.627   -0.573    0.345
##    .s200_au.4        -1.059    0.293   -3.617    0.000   -1.632   -0.485
##    .s200_ma.4        -1.351    0.316   -4.276    0.000   -1.971   -0.732
##    .s200_me.4        -0.825    0.298   -2.766    0.006   -1.409   -0.240
##    .s200_af.4        -0.497    0.306   -1.627    0.104   -1.096    0.102
##    .s200_re.6        -0.160    0.232   -0.689    0.491   -0.614    0.295
##    .s200_au.6        -1.085    0.290   -3.738    0.000   -1.654   -0.516
##    .s200_ma.6        -1.386    0.313   -4.431    0.000   -1.999   -0.773
##    .s200_me.6        -0.882    0.296   -2.981    0.003   -1.461   -0.302
##    .s200_af.6        -0.573    0.302   -1.896    0.058   -1.165    0.019
##    .s200_re.7        -0.153    0.238   -0.642    0.521   -0.620    0.314
##    .s200_au.7        -1.044    0.297   -3.516    0.000   -1.625   -0.462
##    .s200_ma.7        -1.331    0.320   -4.157    0.000   -1.959   -0.704
##    .s200_me.7        -0.871    0.303   -2.871    0.004   -1.466   -0.276
##    .s200_af.7        -0.573    0.309   -1.855    0.064   -1.178    0.032
##     c137.6            0.330    0.036    9.259    0.000    0.260    0.399
##    Std.lv  Std.all
##     9.471    9.471
##     0.048    0.048
##     1.436    1.436
##    -1.626   -1.626
##     8.418    8.418
##     1.155    1.155
##     0.477    0.477
##     0.814    0.814
##     4.887    4.887
##     1.132    1.132
##     0.358    0.358
##    -0.272   -0.272
##     0.512    0.854
##    -2.228   -2.606
##    -1.855   -2.910
##     0.553    0.854
##    -1.998   -2.302
##    -1.746   -2.677
##     0.340    0.532
##    -2.182   -2.490
##    -1.904   -2.859
##     0.347    0.497
##    -2.175   -2.476
##    -1.869   -2.929
##    -0.110   -0.135
##    -1.037   -1.412
##    -1.397   -1.760
##    -0.890   -1.189
##    -0.484   -0.585
##    -0.114   -0.136
##    -1.059   -1.256
##    -1.351   -1.575
##    -0.825   -1.001
##    -0.497   -0.538
##    -0.160   -0.207
##    -1.085   -1.393
##    -1.386   -1.788
##    -0.882   -1.157
##    -0.573   -0.705
##    -0.153   -0.205
##    -1.044   -1.433
##    -1.331   -1.755
##    -0.871   -1.108
##    -0.573   -0.726
##     0.330    0.697
## 
## Variances:
##                    Estimate  Std.Err  z-value  P(>|z|) ci.lower ci.upper
##     JC_W1             0.096    0.020    4.704    0.000    0.056    0.136
##    .djc2              0.030    0.009    3.491    0.000    0.013    0.047
##    .djc3              0.033    0.009    3.583    0.000    0.015    0.050
##    .djc4              0.043    0.014    3.063    0.002    0.015    0.070
##     OJC_W1            0.223    0.036    6.252    0.000    0.153    0.293
##    .dojc2             0.150    0.025    5.899    0.000    0.100    0.200
##    .dojc3             0.164    0.034    4.863    0.000    0.098    0.230
##    .dojc4             0.107    0.026    4.039    0.000    0.055    0.158
##     s38.3r  (rs_s)    0.131    0.015    8.987    0.000    0.102    0.159
##     s38.4r  (rs_s)    0.131    0.015    8.987    0.000    0.102    0.159
##     s38.6   (rs_s)    0.131    0.015    8.987    0.000    0.102    0.159
##     s38.7   (rs_s)    0.131    0.015    8.987    0.000    0.102    0.159
##     s21_r.3           0.414    0.045    9.236    0.000    0.326    0.502
##    .s21_s.3           0.234    0.029    8.210    0.000    0.178    0.290
##    .s21_c.3           0.488    0.058    8.419    0.000    0.374    0.602
##    .s21_s.3           0.184    0.031    5.898    0.000    0.123    0.246
##     s21_r.4           0.373    0.036   10.224    0.000    0.302    0.445
##    .s21_s.4           0.267    0.029    9.284    0.000    0.211    0.323
##    .s21_c.4           0.458    0.050    9.075    0.000    0.359    0.556
##    .s21_s.4           0.155    0.028    5.576    0.000    0.101    0.210
##     s21_r.6           0.331    0.039    8.499    0.000    0.255    0.407
##    .s21_s.6           0.258    0.032    8.124    0.000    0.196    0.320
##    .s21_c.6           0.475    0.061    7.836    0.000    0.356    0.594
##    .s21_s.6           0.176    0.032    5.456    0.000    0.113    0.239
##     s21_r.7           0.390    0.050    7.849    0.000    0.292    0.487
##    .s21_s.7           0.319    0.040    7.915    0.000    0.240    0.399
##    .s21_c.7           0.447    0.063    7.060    0.000    0.323    0.571
##    .s21_s.7           0.110    0.030    3.693    0.000    0.052    0.169
##     s200_.3           0.493    0.052    9.487    0.000    0.391    0.595
##    .s200_.3           0.448    0.048    9.258    0.000    0.353    0.543
##    .s200_.3           0.195    0.028    6.963    0.000    0.140    0.250
##    .s200_.3           0.284    0.036    7.974    0.000    0.214    0.354
##    .s200_.3           0.264    0.033    8.026    0.000    0.200    0.329
##    .s200_.3           0.411    0.047    8.704    0.000    0.318    0.503
##     s200_.4           0.618    0.056   11.112    0.000    0.509    0.727
##    .s200_.4           0.394    0.037   10.585    0.000    0.321    0.467
##    .s200_.4           0.227    0.029    7.959    0.000    0.171    0.283
##    .s200_.4           0.251    0.031    8.183    0.000    0.191    0.311
##    .s200_.4           0.263    0.031    8.517    0.000    0.203    0.324
##    .s200_.4           0.472    0.049    9.663    0.000    0.376    0.567
##     s200_.6           0.482    0.051    9.487    0.000    0.382    0.581
##    .s200_.6           0.323    0.036    8.966    0.000    0.252    0.393
##    .s200_.6           0.186    0.028    6.737    0.000    0.132    0.240
##    .s200_.6           0.178    0.027    6.671    0.000    0.126    0.231
##    .s200_.6           0.220    0.030    7.321    0.000    0.161    0.279
##    .s200_.6           0.326    0.042    7.742    0.000    0.244    0.409
##     s200_.7           0.496    0.060    8.333    0.000    0.380    0.613
##    .s200_.7           0.321    0.040    7.926    0.000    0.241    0.400
##    .s200_.7           0.163    0.028    5.754    0.000    0.107    0.218
##    .s200_.7           0.206    0.034    6.047    0.000    0.140    0.273
##    .s200_.7           0.302    0.044    6.897    0.000    0.216    0.388
##    .s200_.7           0.331    0.048    6.899    0.000    0.237    0.425
##     c137.6            0.224    0.025    8.877    0.000    0.174    0.273
##     SRH_W1            0.557    0.069    8.033    0.000    0.421    0.693
##    .SRH_W2            0.055    0.026    2.095    0.036    0.004    0.107
##    .SRH_W3            0.022    0.022    0.986    0.324   -0.022    0.065
##    .SRH_W4           -0.033    0.027   -1.206    0.228   -0.085    0.020
##    Std.lv  Std.all
##     1.000    1.000
##     0.992    0.992
##     0.961    0.961
##     1.062    1.062
##     1.000    1.000
##     0.969    0.969
##     0.930    0.930
##     0.937    0.937
##     0.131    0.190
##     0.131    0.214
##     0.131    0.247
##     0.131    0.256
##     0.414    0.812
##     0.234    0.650
##     0.488    0.668
##     0.184    0.454
##     0.373    0.762
##     0.267    0.635
##     0.458    0.607
##     0.155    0.365
##     0.331    0.742
##     0.258    0.630
##     0.475    0.619
##     0.176    0.397
##     0.390    0.753
##     0.319    0.655
##     0.447    0.579
##     0.110    0.272
##     0.493    0.688
##     0.448    0.669
##     0.195    0.361
##     0.284    0.451
##     0.264    0.472
##     0.411    0.600
##     0.618    0.664
##     0.394    0.560
##     0.227    0.320
##     0.251    0.341
##     0.263    0.388
##     0.472    0.552
##     0.482    0.639
##     0.323    0.544
##     0.186    0.307
##     0.178    0.297
##     0.220    0.379
##     0.326    0.494
##     0.496    0.676
##     0.321    0.576
##     0.163    0.306
##     0.206    0.359
##     0.302    0.489
##     0.331    0.531
##     0.224    1.000
##     1.000    1.000
##     0.116    0.116
##     0.055    0.055
##    -0.086   -0.086
## 
## 
## Group 2 []:
## 
## Latent Variables:
##                    Estimate  Std.Err  z-value  P(>|z|) ci.lower ci.upper
##   JC_W1 =~                                                              
##     s21_s.3 (j1_2)    0.850    0.152    5.585    0.000    0.551    1.148
##     s21_c.3 (j2_2)    1.983    0.263    7.544    0.000    1.467    2.498
##     s21_s.3  (jc3)    1.523    0.154    9.891    0.000    1.221    1.824
##   JC_W2 =~                                                              
##     s21_s.4 (j1_2)    0.850    0.152    5.585    0.000    0.551    1.148
##     s21_c.4 (j2_2)    1.983    0.263    7.544    0.000    1.467    2.498
##     s21_s.4  (jc3)    1.523    0.154    9.891    0.000    1.221    1.824
##   JC_W3 =~                                                              
##     s21_s.6 (j1_2)    0.850    0.152    5.585    0.000    0.551    1.148
##     s21_c.6 (j2_2)    1.983    0.263    7.544    0.000    1.467    2.498
##     s21_s.6  (jc3)    1.523    0.154    9.891    0.000    1.221    1.824
##   JC_W4 =~                                                              
##     s21_s.7 (j1_2)    0.850    0.152    5.585    0.000    0.551    1.148
##     s21_c.7 (j2_2)    1.983    0.263    7.544    0.000    1.467    2.498
##     s21_s.7  (jc3)    1.523    0.154    9.891    0.000    1.221    1.824
##   OJC_W1 =~                                                             
##     s200_.3 (ojc1)    0.996    0.059   16.816    0.000    0.880    1.112
##     s200_.3 (ojc2)    1.243    0.074   16.838    0.000    1.098    1.388
##     s200_.3 (ojc3)    1.245    0.080   15.576    0.000    1.088    1.402
##     s200_.3 (ojc4)    1.151    0.075   15.255    0.000    1.003    1.299
##     s200_.3 (ojc5)    1.107    0.077   14.346    0.000    0.956    1.259
##   OJC_W2 =~                                                             
##     s200_.4 (ojc1)    0.996    0.059   16.816    0.000    0.880    1.112
##     s200_.4 (ojc2)    1.243    0.074   16.838    0.000    1.098    1.388
##     s200_.4 (ojc3)    1.245    0.080   15.576    0.000    1.088    1.402
##     s200_.4 (ojc4)    1.151    0.075   15.255    0.000    1.003    1.299
##     s200_.4 (ojc5)    1.107    0.077   14.346    0.000    0.956    1.259
##   OJC_W3 =~                                                             
##     s200_.6 (ojc1)    0.996    0.059   16.816    0.000    0.880    1.112
##     s200_.6 (ojc2)    1.243    0.074   16.838    0.000    1.098    1.388
##     s200_.6 (ojc3)    1.245    0.080   15.576    0.000    1.088    1.402
##     s200_.6 (ojc4)    1.151    0.075   15.255    0.000    1.003    1.299
##     s200_.6 (ojc5)    1.107    0.077   14.346    0.000    0.956    1.259
##   OJC_W4 =~                                                             
##     s200_.7 (ojc1)    0.996    0.059   16.816    0.000    0.880    1.112
##     s200_.7 (ojc2)    1.243    0.074   16.838    0.000    1.098    1.388
##     s200_.7 (ojc3)    1.245    0.080   15.576    0.000    1.088    1.402
##     s200_.7 (ojc4)    1.151    0.075   15.255    0.000    1.003    1.299
##     s200_.7 (ojc5)    1.107    0.077   14.346    0.000    0.956    1.259
##    Std.lv  Std.all
##                   
##     0.289    0.466
##     0.675    0.770
##     0.519    0.721
##                   
##     0.295    0.513
##     0.689    0.751
##     0.529    0.717
##                   
##     0.301    0.440
##     0.702    0.737
##     0.539    0.725
##                   
##     0.300    0.482
##     0.700    0.739
##     0.537    0.765
##                   
##     0.521    0.623
##     0.650    0.803
##     0.651    0.724
##     0.602    0.763
##     0.579    0.733
##                   
##     0.542    0.672
##     0.676    0.859
##     0.677    0.788
##     0.626    0.845
##     0.602    0.753
##                   
##     0.552    0.725
##     0.688    0.923
##     0.689    0.806
##     0.637    0.824
##     0.613    0.762
##                   
##     0.547    0.694
##     0.683    0.852
##     0.684    0.849
##     0.632    0.834
##     0.608    0.743
## 
## Regressions:
##                    Estimate  Std.Err  z-value  P(>|z|) ci.lower ci.upper
##   djc2 ~                                                                
##     JC_W1            -0.173    0.099   -1.750    0.080   -0.367    0.021
##   djc3 ~                                                                
##     JC_W2             0.004    0.129    0.028    0.978   -0.249    0.257
##   djc4 ~                                                                
##     JC_W3            -0.116    0.217   -0.535    0.593   -0.541    0.309
##   dojc2 ~                                                               
##     OJC_W1           -0.201    0.094   -2.147    0.032   -0.385   -0.018
##   dojc3 ~                                                               
##     OJC_W2            0.034    0.153    0.221    0.825   -0.265    0.333
##   dojc4 ~                                                               
##     OJC_W3            0.051    0.165    0.312    0.755   -0.272    0.374
##   SRH_W2 ~                                                              
##     SRH_W1            1.009    0.132    7.672    0.000    0.752    1.267
##   SRH_W3 ~                                                              
##     SRH_W2            0.970    0.180    5.391    0.000    0.617    1.322
##   SRH_W4 ~                                                              
##     SRH_W3            1.309    0.228    5.747    0.000    0.863    1.756
##   SRH_W2 ~                                                              
##     djc2             -0.181    0.259   -0.699    0.484   -0.688    0.326
##     dojc2             0.015    0.138    0.108    0.914   -0.257    0.286
##     c137.6           -0.099    0.214   -0.460    0.646   -0.519    0.322
##   SRH_W3 ~                                                              
##     djc3              0.920    0.694    1.326    0.185   -0.440    2.280
##     dojc3             0.005    0.177    0.031    0.976   -0.341    0.352
##     c137.6            0.163    0.317    0.514    0.607   -0.458    0.784
##   SRH_W4 ~                                                              
##     djc4              0.516    0.324    1.594    0.111   -0.119    1.151
##     dojc4             0.697    0.342    2.040    0.041    0.027    1.368
##     c137.6           -0.005    0.310   -0.018    0.986   -0.612    0.601
##    Std.lv  Std.all
##                   
##    -0.279   -0.279
##                   
##     0.007    0.007
##                   
##    -0.125   -0.125
##                   
##    -0.289   -0.289
##                   
##     0.036    0.036
##                   
##     0.074    0.074
##                   
##     1.084    1.084
##                   
##     0.891    0.891
##                   
##     0.959    0.959
##                   
##    -0.068   -0.068
##     0.010    0.010
##    -0.175   -0.064
##                   
##     0.267    0.267
##     0.005    0.005
##     0.265    0.097
##                   
##     0.202    0.202
##     0.321    0.321
##    -0.007   -0.002
## 
## Covariances:
##                    Estimate  Std.Err  z-value  P(>|z|) ci.lower ci.upper
##   s21_rd.3 ~~                                                           
##     s21_rd.4          0.109    0.039    2.784    0.005    0.032    0.186
##     s21_rd.6          0.168    0.050    3.331    0.001    0.069    0.266
##     s21_rd.7          0.137    0.049    2.769    0.006    0.040    0.234
##   s21_rd.4 ~~                                                           
##     s21_rd.6          0.119    0.041    2.900    0.004    0.038    0.199
##     s21_rd.7          0.002    0.048    0.052    0.959   -0.091    0.096
##   s21_rd.6 ~~                                                           
##     s21_rd.7          0.151    0.052    2.920    0.003    0.050    0.252
##  .s21_str.3 ~~                                                          
##    .s21_str.4         0.162    0.037    4.398    0.000    0.090    0.234
##    .s21_str.6         0.207    0.057    3.659    0.000    0.096    0.318
##    .s21_str.7         0.201    0.047    4.287    0.000    0.109    0.293
##  .s21_str.4 ~~                                                          
##    .s21_str.6         0.171    0.044    3.891    0.000    0.085    0.258
##    .s21_str.7         0.196    0.040    4.852    0.000    0.117    0.275
##  .s21_str.6 ~~                                                          
##    .s21_str.7         0.231    0.053    4.341    0.000    0.127    0.336
##  .s21_cd.3 ~~                                                           
##    .s21_cd.4          0.235    0.069    3.431    0.001    0.101    0.370
##    .s21_cd.6          0.238    0.077    3.079    0.002    0.086    0.389
##    .s21_cd.7         -0.027    0.071   -0.380    0.704   -0.167    0.113
##  .s21_cd.4 ~~                                                           
##    .s21_cd.6          0.234    0.078    3.006    0.003    0.081    0.386
##    .s21_cd.7          0.188    0.080    2.360    0.018    0.032    0.344
##  .s21_cd.6 ~~                                                           
##    .s21_cd.7          0.198    0.090    2.207    0.027    0.022    0.374
##  .s21_soz.3 ~~                                                          
##    .s21_soz.4         0.164    0.047    3.510    0.000    0.072    0.255
##    .s21_soz.6         0.178    0.051    3.483    0.000    0.078    0.279
##    .s21_soz.7         0.146    0.043    3.381    0.001    0.061    0.231
##  .s21_soz.4 ~~                                                          
##    .s21_soz.6         0.158    0.049    3.224    0.001    0.062    0.254
##    .s21_soz.7         0.158    0.046    3.408    0.001    0.067    0.249
##  .s21_soz.6 ~~                                                          
##    .s21_soz.7         0.219    0.051    4.293    0.000    0.119    0.319
##  .djc2 ~~                                                               
##    .djc3             -0.014    0.012   -1.142    0.254   -0.038    0.010
##    .djc4             -0.008    0.017   -0.476    0.634   -0.041    0.025
##  .djc3 ~~                                                               
##    .djc4             -0.032    0.017   -1.918    0.055   -0.064    0.001
##   s200_de.3 ~~                                                          
##    .s200_re.3         0.181    0.059    3.056    0.002    0.065    0.297
##   s200_de.4 ~~                                                          
##    .s200_re.4         0.079    0.037    2.125    0.034    0.006    0.152
##   s200_de.6 ~~                                                          
##    .s200_re.6         0.073    0.037    1.960    0.050   -0.000    0.147
##   s200_de.7 ~~                                                          
##    .s200_re.7         0.024    0.045    0.528    0.597   -0.065    0.113
##   s200_de.3 ~~                                                          
##     s200_de.4         0.188    0.060    3.150    0.002    0.071    0.305
##     s200_de.6         0.132    0.062    2.133    0.033    0.011    0.253
##     s200_de.7         0.065    0.069    0.949    0.343   -0.069    0.199
##   s200_de.4 ~~                                                          
##     s200_de.6         0.152    0.054    2.802    0.005    0.046    0.258
##     s200_de.7         0.242    0.064    3.765    0.000    0.116    0.368
##   s200_de.6 ~~                                                          
##     s200_de.7         0.253    0.067    3.778    0.000    0.122    0.384
##  .s200_re.3 ~~                                                          
##    .s200_re.4         0.197    0.047    4.188    0.000    0.105    0.289
##    .s200_re.6         0.142    0.048    2.934    0.003    0.047    0.237
##    .s200_re.7         0.123    0.062    1.996    0.046    0.002    0.244
##  .s200_re.4 ~~                                                          
##    .s200_re.6         0.108    0.043    2.510    0.012    0.024    0.192
##    .s200_re.7         0.099    0.055    1.800    0.072   -0.009    0.207
##  .s200_re.6 ~~                                                          
##    .s200_re.7         0.136    0.054    2.537    0.011    0.031    0.241
##  .s200_au.3 ~~                                                          
##    .s200_au.4         0.063    0.034    1.859    0.063   -0.003    0.129
##    .s200_au.6        -0.010    0.032   -0.299    0.765   -0.073    0.054
##    .s200_au.7        -0.084    0.048   -1.755    0.079   -0.177    0.010
##  .s200_au.4 ~~                                                          
##    .s200_au.6         0.040    0.025    1.631    0.103   -0.008    0.088
##    .s200_au.7         0.050    0.039    1.269    0.204   -0.027    0.126
##  .s200_au.6 ~~                                                          
##    .s200_au.7         0.019    0.033    0.572    0.567   -0.045    0.082
##  .s200_ma.3 ~~                                                          
##    .s200_ma.4         0.139    0.051    2.738    0.006    0.040    0.239
##    .s200_ma.6         0.060    0.049    1.227    0.220   -0.036    0.155
##    .s200_ma.7         0.010    0.047    0.216    0.829   -0.083    0.103
##  .s200_ma.4 ~~                                                          
##    .s200_ma.6         0.085    0.039    2.200    0.028    0.009    0.160
##    .s200_ma.7         0.073    0.040    1.836    0.066   -0.005    0.150
##  .s200_ma.6 ~~                                                          
##    .s200_ma.7         0.135    0.049    2.722    0.006    0.038    0.232
##  .s200_me.3 ~~                                                          
##    .s200_me.4         0.060    0.029    2.038    0.042    0.002    0.117
##    .s200_me.6         0.085    0.041    2.044    0.041    0.003    0.166
##    .s200_me.7         0.128    0.045    2.815    0.005    0.039    0.217
##  .s200_me.4 ~~                                                          
##    .s200_me.6         0.047    0.033    1.433    0.152   -0.017    0.112
##    .s200_me.7         0.086    0.034    2.578    0.010    0.021    0.152
##  .s200_me.6 ~~                                                          
##    .s200_me.7         0.086    0.039    2.220    0.026    0.010    0.162
##  .s200_af.3 ~~                                                          
##    .s200_af.4         0.069    0.039    1.759    0.079   -0.008    0.146
##    .s200_af.6         0.092    0.050    1.856    0.063   -0.005    0.190
##    .s200_af.7         0.130    0.061    2.123    0.034    0.010    0.250
##  .s200_af.4 ~~                                                          
##    .s200_af.6         0.021    0.043    0.502    0.615   -0.062    0.105
##    .s200_af.7         0.090    0.051    1.744    0.081   -0.011    0.190
##  .s200_af.6 ~~                                                          
##    .s200_af.7         0.148    0.054    2.749    0.006    0.043    0.254
##  .dojc2 ~~                                                              
##    .dojc3            -0.135    0.042   -3.227    0.001   -0.217   -0.053
##    .dojc4            -0.001    0.031   -0.029    0.977   -0.062    0.060
##  .dojc3 ~~                                                              
##    .dojc4            -0.091    0.045   -2.012    0.044   -0.181   -0.002
##  .djc2 ~~                                                               
##    .dojc2             0.010    0.009    1.043    0.297   -0.008    0.027
##  .djc3 ~~                                                               
##    .dojc3             0.034    0.015    2.262    0.024    0.004    0.063
##  .djc4 ~~                                                               
##    .dojc4            -0.015    0.020   -0.747    0.455   -0.053    0.024
##   JC_W1 ~~                                                              
##     c137.6            0.028    0.019    1.528    0.126   -0.008    0.065
##   OJC_W1 ~~                                                             
##     c137.6            0.023    0.026    0.866    0.386   -0.029    0.074
##   SRH_W1 ~~                                                             
##     c137.6           -0.104    0.039   -2.660    0.008   -0.180   -0.027
##  .djc2 ~~                                                               
##     c137.6           -0.012    0.011   -1.164    0.245   -0.033    0.008
##  .djc3 ~~                                                               
##     c137.6           -0.017    0.012   -1.483    0.138   -0.040    0.005
##  .djc4 ~~                                                               
##     c137.6            0.032    0.018    1.786    0.074   -0.003    0.067
##  .dojc2 ~~                                                              
##     c137.6            0.000    0.018    0.025    0.980   -0.034    0.035
##  .dojc3 ~~                                                              
##     c137.6           -0.033    0.026   -1.272    0.203   -0.084    0.018
##  .dojc4 ~~                                                              
##     c137.6            0.016    0.020    0.784    0.433   -0.024    0.056
##   JC_W1 ~~                                                              
##     OJC_W1            0.069    0.027    2.584    0.010    0.017    0.121
##     SRH_W1            0.023    0.026    0.898    0.369   -0.027    0.073
##   OJC_W1 ~~                                                             
##     SRH_W1            0.121    0.041    2.963    0.003    0.041    0.200
##    Std.lv  Std.all
##                   
##     0.109    0.376
##     0.168    0.521
##     0.137    0.454
##                   
##     0.119    0.409
##     0.002    0.009
##                   
##     0.151    0.501
##                   
##     0.162    0.595
##     0.207    0.613
##     0.201    0.671
##                   
##     0.171    0.566
##     0.196    0.729
##                   
##     0.231    0.692
##                   
##     0.235    0.695
##     0.238    0.660
##    -0.027   -0.076
##                   
##     0.234    0.598
##     0.188    0.488
##                   
##     0.198    0.482
##                   
##     0.164    0.637
##     0.178    0.698
##     0.146    0.647
##                   
##     0.158    0.601
##     0.158    0.680
##                   
##     0.219    0.946
##                   
##    -0.383   -0.383
##    -0.126   -0.126
##                   
##    -0.572   -0.572
##                   
##     0.181    0.360
##                   
##     0.079    0.189
##                   
##     0.073    0.222
##                   
##     0.024    0.065
##                   
##     0.188    0.349
##     0.132    0.272
##     0.065    0.131
##                   
##     0.152    0.345
##     0.242    0.535
##                   
##     0.253    0.622
##                   
##     0.197    0.505
##     0.142    0.414
##     0.123    0.332
##                   
##     0.108    0.344
##     0.099    0.292
##                   
##     0.136    0.457
##                   
##     0.063    0.323
##    -0.010   -0.069
##    -0.084   -0.412
##                   
##     0.040    0.346
##     0.050    0.292
##                   
##     0.019    0.154
##                   
##     0.139    0.423
##     0.060    0.191
##     0.010    0.039
##                   
##     0.085    0.317
##     0.073    0.321
##                   
##     0.135    0.625
##                   
##     0.060    0.296
##     0.085    0.380
##     0.128    0.600
##                   
##     0.047    0.272
##     0.086    0.522
##                   
##     0.086    0.468
##                   
##     0.069    0.244
##     0.092    0.330
##     0.130    0.443
##                   
##     0.021    0.078
##     0.090    0.311
##                   
##     0.148    0.520
##                   
##    -0.745   -0.745
##    -0.006   -0.006
##                   
##    -0.445   -0.445
##                   
##     0.134    0.134
##                   
##     0.362    0.362
##                   
##    -0.119   -0.119
##                   
##     0.084    0.229
##                   
##     0.043    0.118
##                   
##    -0.171   -0.468
##                   
##    -0.060   -0.165
##                   
##    -0.096   -0.262
##                   
##     0.103    0.282
##                   
##     0.001    0.003
##                   
##    -0.063   -0.173
##                   
##     0.040    0.109
##                   
##     0.387    0.387
##     0.111    0.111
##                   
##     0.381    0.381
## 
## Intercepts:
##                    Estimate  Std.Err  z-value  P(>|z|) ci.lower ci.upper
##     JC_W1             3.101    0.074   41.982    0.000    2.956    3.246
##    .djc2              0.325    0.313    1.036    0.300   -0.289    0.939
##    .djc3              0.189    0.379    0.499    0.618   -0.554    0.932
##    .djc4              0.273    0.670    0.407    0.684   -1.040    1.587
##     OJC_W1            3.823    0.104   36.643    0.000    3.619    4.027
##    .dojc2             0.772    0.370    2.089    0.037    0.048    1.497
##    .dojc3            -0.345    0.592   -0.584    0.559   -1.505    0.814
##    .dojc4            -0.112    0.601   -0.187    0.852   -1.291    1.066
##     SRH_W1            3.591    0.074   48.724    0.000    3.446    3.735
##    .SRH_W2            0.021    0.500    0.042    0.967   -0.959    1.001
##    .SRH_W3            0.012    0.733    0.016    0.987   -1.425    1.449
##    .SRH_W4           -1.418    0.899   -1.577    0.115   -3.180    0.345
##    .s21_str.3         1.261    0.480    2.628    0.009    0.320    2.201
##    .s21_cd.3         -3.445    0.828   -4.159    0.000   -5.069   -1.822
##    .s21_soz.3        -1.907    0.492   -3.876    0.000   -2.871   -0.943
##    .s21_str.4         1.360    0.444    3.062    0.002    0.489    2.230
##    .s21_cd.4         -3.094    0.768   -4.030    0.000   -4.599   -1.589
##    .s21_soz.4        -1.685    0.454   -3.713    0.000   -2.575   -0.796
##    .s21_str.6         1.161    0.479    2.425    0.015    0.223    2.099
##    .s21_cd.6         -3.412    0.826   -4.133    0.000   -5.029   -1.794
##    .s21_soz.6        -1.949    0.488   -3.992    0.000   -2.907   -0.992
##    .s21_str.7         1.201    0.469    2.563    0.010    0.283    2.120
##    .s21_cd.7         -3.480    0.807   -4.311    0.000   -5.063   -1.898
##    .s21_soz.7        -2.079    0.479   -4.340    0.000   -3.018   -1.140
##    .s200_re.3        -0.191    0.244   -0.782    0.434   -0.668    0.287
##    .s200_au.3        -1.039    0.308   -3.376    0.001   -1.642   -0.436
##    .s200_ma.3        -1.207    0.332   -3.629    0.000   -1.858   -0.555
##    .s200_me.3        -0.761    0.312   -2.443    0.015   -1.372   -0.150
##    .s200_af.3        -0.321    0.317   -1.013    0.311   -0.943    0.300
##    .s200_re.4        -0.179    0.241   -0.743    0.458   -0.650    0.293
##    .s200_au.4        -0.928    0.298   -3.121    0.002   -1.512   -0.345
##    .s200_ma.4        -1.273    0.322   -3.957    0.000   -1.903   -0.642
##    .s200_me.4        -0.708    0.302   -2.348    0.019   -1.300   -0.117
##    .s200_af.4        -0.479    0.309   -1.549    0.121   -1.084    0.127
##    .s200_re.6        -0.177    0.230   -0.773    0.440   -0.628    0.273
##    .s200_au.6        -0.904    0.284   -3.184    0.001   -1.460   -0.347
##    .s200_ma.6        -1.165    0.309   -3.771    0.000   -1.770   -0.559
##    .s200_me.6        -0.658    0.290   -2.267    0.023   -1.227   -0.089
##    .s200_af.6        -0.389    0.297   -1.308    0.191   -0.971    0.194
##    .s200_re.7        -0.173    0.244   -0.707    0.479   -0.651    0.306
##    .s200_au.7        -0.963    0.300   -3.214    0.001   -1.550   -0.375
##    .s200_ma.7        -1.237    0.317   -3.901    0.000   -1.858   -0.615
##    .s200_me.7        -0.731    0.300   -2.435    0.015   -1.319   -0.143
##    .s200_af.7        -0.429    0.308   -1.389    0.165   -1.033    0.176
##     c137.6            0.174    0.045    3.908    0.000    0.087    0.262
##    Std.lv  Std.all
##     9.102    9.102
##     1.534    1.534
##     1.061    1.061
##     0.832    0.832
##     7.314    7.314
##     2.121    2.121
##    -0.677   -0.677
##    -0.292   -0.292
##     5.922    5.922
##     0.037    0.037
##     0.019    0.019
##    -1.692   -1.692
##     1.261    2.029
##    -3.445   -3.930
##    -1.907   -2.649
##     1.360    2.365
##    -3.094   -3.373
##    -1.685   -2.284
##     1.161    1.698
##    -3.412   -3.579
##    -1.949   -2.622
##     1.201    1.933
##    -3.480   -3.678
##    -2.079   -2.961
##    -0.191   -0.228
##    -1.039   -1.283
##    -1.207   -1.342
##    -0.761   -0.966
##    -0.321   -0.407
##    -0.179   -0.222
##    -0.928   -1.179
##    -1.273   -1.480
##    -0.708   -0.957
##    -0.479   -0.598
##    -0.177   -0.233
##    -0.904   -1.212
##    -1.165   -1.362
##    -0.658   -0.851
##    -0.389   -0.483
##    -0.173   -0.219
##    -0.963   -1.200
##    -1.237   -1.534
##    -0.731   -0.964
##    -0.429   -0.524
##     0.174    0.476
## 
## Variances:
##                    Estimate  Std.Err  z-value  P(>|z|) ci.lower ci.upper
##     JC_W1             0.116    0.033    3.499    0.000    0.051    0.181
##    .djc2              0.041    0.014    3.011    0.003    0.014    0.068
##    .djc3              0.032    0.014    2.249    0.025    0.004    0.060
##    .djc4              0.097    0.036    2.651    0.008    0.025    0.168
##     OJC_W1            0.273    0.057    4.830    0.000    0.162    0.384
##    .dojc2             0.122    0.029    4.185    0.000    0.065    0.178
##    .dojc3             0.269    0.077    3.513    0.000    0.119    0.419
##    .dojc4             0.157    0.057    2.741    0.006    0.045    0.270
##     s38.3r  (rs_s)    0.131    0.015    8.987    0.000    0.102    0.159
##     s38.4r  (rs_s)    0.131    0.015    8.987    0.000    0.102    0.159
##     s38.6   (rs_s)    0.131    0.015    8.987    0.000    0.102    0.159
##     s38.7   (rs_s)    0.131    0.015    8.987    0.000    0.102    0.159
##     s21_r.3           0.322    0.058    5.534    0.000    0.208    0.436
##    .s21_s.3           0.302    0.054    5.584    0.000    0.196    0.409
##    .s21_c.3           0.313    0.082    3.808    0.000    0.152    0.473
##    .s21_s.3           0.249    0.056    4.445    0.000    0.139    0.359
##     s21_r.4           0.261    0.042    6.188    0.000    0.179    0.344
##    .s21_s.4           0.243    0.038    6.337    0.000    0.168    0.319
##    .s21_c.4           0.367    0.082    4.478    0.000    0.206    0.528
##    .s21_s.4           0.265    0.054    4.858    0.000    0.158    0.372
##     s21_r.6           0.321    0.061    5.283    0.000    0.202    0.440
##    .s21_s.6           0.377    0.072    5.256    0.000    0.236    0.517
##    .s21_c.6           0.416    0.107    3.886    0.000    0.206    0.625
##    .s21_s.6           0.262    0.062    4.254    0.000    0.141    0.383
##     s21_r.7           0.282    0.064    4.418    0.000    0.157    0.407
##    .s21_s.7           0.297    0.058    5.108    0.000    0.183    0.410
##    .s21_c.7           0.406    0.122    3.330    0.001    0.167    0.645
##    .s21_s.7           0.204    0.055    3.696    0.000    0.096    0.313
##     s200_.3           0.590    0.103    5.755    0.000    0.389    0.791
##    .s200_.3           0.428    0.075    5.710    0.000    0.281    0.575
##    .s200_.3           0.233    0.054    4.348    0.000    0.128    0.338
##    .s200_.3           0.385    0.077    5.035    0.000    0.235    0.535
##    .s200_.3           0.260    0.052    4.975    0.000    0.157    0.362
##    .s200_.3           0.288    0.056    5.114    0.000    0.178    0.399
##     s200_.4           0.490    0.071    6.891    0.000    0.350    0.629
##    .s200_.4           0.356    0.053    6.681    0.000    0.252    0.461
##    .s200_.4           0.163    0.031    5.169    0.000    0.101    0.224
##    .s200_.4           0.281    0.048    5.892    0.000    0.187    0.374
##    .s200_.4           0.156    0.029    5.359    0.000    0.099    0.214
##    .s200_.4           0.277    0.044    6.288    0.000    0.191    0.363
##     s200_.6           0.397    0.072    5.512    0.000    0.256    0.538
##    .s200_.6           0.275    0.050    5.446    0.000    0.176    0.374
##    .s200_.6           0.083    0.028    2.998    0.003    0.029    0.137
##    .s200_.6           0.255    0.053    4.857    0.000    0.152    0.359
##    .s200_.6           0.192    0.044    4.406    0.000    0.107    0.277
##    .s200_.6           0.271    0.057    4.771    0.000    0.160    0.383
##     s200_.7           0.417    0.091    4.608    0.000    0.240    0.595
##    .s200_.7           0.322    0.077    4.196    0.000    0.171    0.472
##    .s200_.7           0.177    0.055    3.210    0.001    0.069    0.284
##    .s200_.7           0.182    0.054    3.347    0.001    0.075    0.288
##    .s200_.7           0.175    0.047    3.714    0.000    0.083    0.268
##    .s200_.7           0.300    0.073    4.077    0.000    0.156    0.444
##     c137.6            0.134    0.024    5.655    0.000    0.087    0.180
##     SRH_W1            0.368    0.073    5.044    0.000    0.225    0.510
##    .SRH_W2           -0.080    0.033   -2.424    0.015   -0.144   -0.015
##    .SRH_W3            0.080    0.041    1.961    0.050    0.000    0.161
##    .SRH_W4           -0.008    0.088   -0.095    0.924   -0.181    0.164
##    Std.lv  Std.all
##     1.000    1.000
##     0.922    0.922
##     1.003    1.003
##     0.899    0.899
##     1.000    1.000
##     0.916    0.916
##     1.034    1.034
##     1.058    1.058
##     0.131    0.262
##     0.131    0.291
##     0.131    0.257
##     0.131    0.157
##     0.322    0.735
##     0.302    0.783
##     0.313    0.407
##     0.249    0.481
##     0.261    0.684
##     0.243    0.737
##     0.367    0.436
##     0.265    0.486
##     0.321    0.719
##     0.377    0.806
##     0.416    0.457
##     0.262    0.474
##     0.282    0.694
##     0.297    0.767
##     0.406    0.453
##     0.204    0.414
##     0.590    0.684
##     0.428    0.612
##     0.233    0.356
##     0.385    0.476
##     0.260    0.418
##     0.288    0.462
##     0.490    0.623
##     0.356    0.548
##     0.163    0.263
##     0.281    0.380
##     0.156    0.285
##     0.277    0.433
##     0.397    0.564
##     0.275    0.475
##     0.083    0.148
##     0.255    0.350
##     0.192    0.321
##     0.271    0.419
##     0.417    0.580
##     0.322    0.518
##     0.177    0.275
##     0.182    0.280
##     0.175    0.305
##     0.300    0.447
##     0.134    1.000
##     1.000    1.000
##    -0.250   -0.250
##     0.214    0.214
##    -0.012   -0.012
```

```
fitMeasures(lcsm_parcel_SRH_GC3, c("chisq", "df", "pvalue", "cfi", "tli", "rmsea", "srmr"), fm.args = list(robust = FALSE))
```

```
##    chisq       df   pvalue      cfi      tli    rmsea     srmr 
## 6309.252 1747.000    0.000    0.613    0.561    0.122    0.107
```

```
# Unfortunately, both models do not converge properly, therefore we only include these analyses for full transparency. 
# We suggest that job crafting research should investigate measurement differences for similar subgroups and potential explanations.
```
